# Direct Photochemical Synthesis of Substituted Benzo[*b*]fluorenes

**DOI:** 10.1021/acs.orglett.4c03978

**Published:** 2024-11-25

**Authors:** Ruairi Crawford, Yannick Ortin, Brendan Twamley, Marcus Baumann

**Affiliations:** † School of Chemistry, University College Dublin, Dublin 4, Ireland; ‡ School of Chemistry, Trinity College Dublin, Dublin 2, Ireland

## Abstract

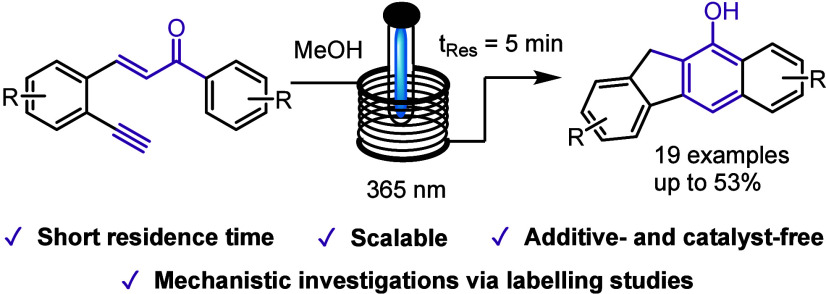

We report a new and straightforward route toward substituted benzo[*b*]fluorenes via the direct photochemical conversion of alkynylated chalcones. This transformation exploits a high-power light-emitting diode emitting ultraviolet A light to enable the rapid formation of the target products (*t*_res_ = 5 min). A continuous flow approach thereby facilitates reproducibility and scalability, granting streamlined access to these important scaffolds and their derivatives. A mechanistic proposal based on a biradical species is presented and supported by deuteration studies.

Fluorenes represent an important and structurally diverse class of aromatic hydrocarbons with many applications as fluorescent dyes, polymer precursors [e.g., for polyfluorene in organic light-emitting diodes (OLEDs)], and synthetic building blocks (i.e., Fmoc-protecting group).^1^ The addition of benzene rings gives rise to different benzofluorene species that have been studied extensively as DNA adducts.^2^ Access to these important scaffolds typically relies on multistep syntheses as reported previously. A limitation of these routes is their linear nature, the use of stoichiometric reagents, and the generation of large amounts of chemical waste.^3^

Continuous flow chemistry has emerged as a powerful technology that enables safe and efficient chemical reactions by exploiting reactor miniaturization in combination with improved heat and mass transfer.^4^ Integration of inline analysis and purification tools furthermore allows for reaction telescoping, and extended operation of flow reactors provides for an attractive scale-up option using small footprint equipment.^5^ Moreover, the area of photochemistry benefits from continuous flow processing due to short pathlengths of light, uniform irradiation profiles, and high spatiotemporal control.^6^ In addition to simple scalability, flow photochemistry oftentimes facilitates faster and cleaner photochemical reactions due to the constant removal of the reaction product from the irradiated reactor zone.^7^ As demonstrated by many recent studies, this can lead to improved reaction outcomes, yet it can also enable the discovery of new reactions and reactivities.^8^ For instance, our group exploited continuous flow processing for the discovery of a novel skeletal rearrangement of simple alkynylated chalcones. Irradiating solutions of these substrates thereby generates complex pentacyclic scaffolds containing cyclobutenes, cyclopentenes, and cycloheptatrienes as versatile substructures (top of Scheme 1).^8a,9^

**Scheme 1:**
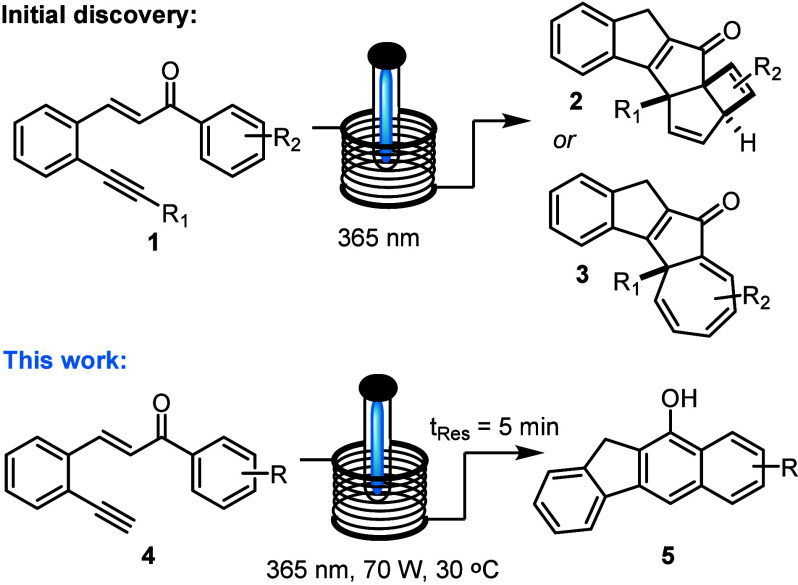
Photochemical Transformation of Alkynylated Chalcones in Flow Mode

Crucially, this new reactivity is based on the alkyne moiety intercepting the isomerizing enone species, which triggers several rearrangements in a complex sequence. Various substituents can be incorporated into these scaffolds based on the specific alkyne and benzene species employed, thus providing rapid access to new chemical space. Subsequent efforts from our group investigated the use of terminal alkynes in an analogous photochemical cascade process, which led to the discovery of an unprecedented route to diverse benzo[*b*]fluorene products in a simple and streamlined manner (bottom of Scheme 1). Here, we communicate our findings, including mechanistic investigations, exploiting deuterium-labeling studies.

Our efforts commenced with alkyne substrate **4a**, which was subjected to photochemical conditions using a commercial Vapourtec E-Series flow reactor and its photomodule with high-power light-emitting diodes (LEDs) emitting at 365 nm. Using this setup, it was quickly established that short exposure of solutions of compound **4a** (7 min, 80 mM MeCN) afforded a major new product. Detailed NMR studies showed that both the alkyne and the ketone had been converted into a new aromatic ring system which, based on ^1^H nuclear magnetic resonance (NMR) and infrared (IR) analyses, contained a hydroxy group. Moreover, the methoxy-substituted benzene ring had become part of this fused ring system. The presence of a benzylic methylene group indicated that a conjugated tetracyclic scaffold had been formed via a formal photoannulation process, rendering a new aromatic ring. Finally, single crystal X-ray diffraction analysis confirmed the molecular structure of compound **5a** as benzo[*b*]fluorene (Scheme 2).

**Scheme 2:**
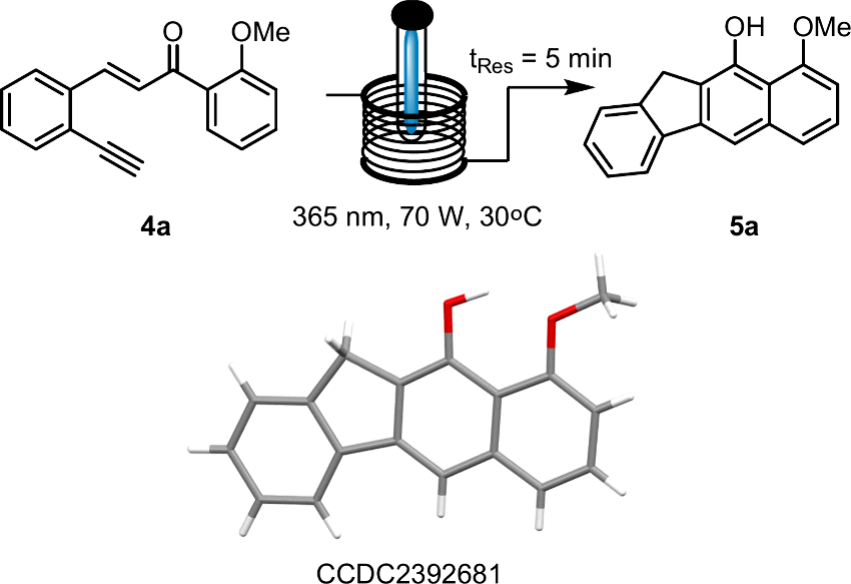
Photochemical Generation of Benzo[*b*]fluorene **5a** and Its X-ray Structure

Although several studies report the synthesis of related benzo[*b*]fluorene scaffolds, their preparation is typically based on multistep sequences requiring stoichiometric reagents under harsh reaction conditions.^10^ For instance, a report by Li and co-workers accessed the benzo[*b*]fluorene scaffold via an interesting high-temperature radical process starting from chalcones, tosyl hydrazines, and alkynes in the presence of a copper catalyst.^3a^ Tu and co-workers developed a related thermal route affording either benzo[*b*]fluorenones or benzo[*b*]fluorenols via a base-controlled 1,6-enyne bicyclization strategy.^11^ In view of the apparent lack of mild and atom-efficient entries into these versatile benzo[*b*]fluorenes, we exploit our initial discovery to realize a mild and fast photochemical approach.

Due to the low initial yield of compound **5a** (ca. 15%) when using acetonitrile as a solvent, further common organic solvents were screened for this photochemical process. As shown in Table 1 various aprotic solvents [e.g., acetonitrile (MeCN), tetrahydrofuran (THF), and ethyl acetate (EtOAc)] gave equally poor yields, whereas polar solvents, such as methanol, gave a significantly increased yield of 45%, along with 29% unreacted starting material, which could be recovered easily. The remaining mass balance (ca. 26%) indicates product loss due to photodecomposition. Going forward, it was decided that short residence times would be preferred over longer residence times that would give full substrate conversion as this would minimize side-product formation due to the competing absorbance of the reaction product. Moreover, this strategy would allow recovery of the substrate that can be reused, and it simplifies product isolation due to fewer side products requiring separation via chromatography. In addition, fluorinated alcohols, such as trifluoroethanol (TFE) and hexafluoroisopropanol (HFIP), were tested as they can stabilize radical intermediates in related processes;^12^ however, in this case, no further improvements were obtained. Trialing other light sources, light intensities, and residence times (see the Supporting Information for full details) did not yield further improvements on the best conditions (entry 4 in Table 1).

**Table 1:**
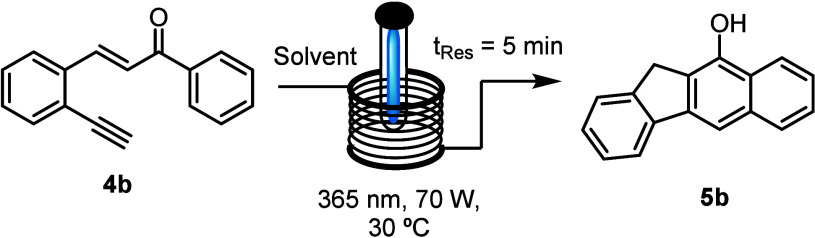
Solvent Optimization toward Benzo[*b*]fluorene **5b**^a^

entry	solvent	yield of compound **5b** (%)
**1**	MeCN	15
**2**	THF	14
**3**	EtOAc	3
**4**	MeOH	45 (29)
**5**	TFE	38
**6**	HFIP	49 (18)

a Reactions were performed at 0.14 mmol scale in the corresponding solvent at 50 mM. Reported yields are ^1^H NMR yields of compound **5b** (% starting material) using trichloroethylene as the internal standard.

Next, we studied the scope of this photochemical process for which we first modified the right-hand aryl ring with different substituents (Scheme 3). This showed that the addition of various electron-withdrawing groups in the *para* position (**5c**–**5e**) resulted in yields similar to those of compound **5a**. A nitrile group was well-tolerated under the reaction conditions, indicating opportunities for further product derivatization. Products **5f** and **5g** bearing methoxy and methyl substituents gave similar yields of 31–46%. A small drop in the yield was seen when two methyl groups were placed on the phenyl ring resulting in regioisomers (**5h**). Similarly, fluorine in the *meta* positions resulted in a set of regioisomers and a yield comparable to that of compound **5i** with 22% of starting material remaining. The most significant drop in yield was observed for compounds **5k**–**5m** (18–34%), indicating that *ortho* substituents may favor alternative reaction pathways, including product decomposition. Pleasingly, scaling up the model substrate to process 2.3 g of substrate **4b** resulted in a product yield of 41%, which is nearly identical to the small-scale reaction, thus highlighting the ease of scaling this continuous flow approach. It is interesting to note that starting material could be recovered for nearly all cases from the reaction mixture as either the *E* or *Z* isomer of the chalcone substrate, indicating that breaking of this π bond is part of the reaction mechanism (*vide infra*). Moreover, further increasing the residence time from 5 to 15 min did not significantly alter the product yield (i.e., for compounds **5a**, **5f**, and **5l**), indicating that recovery of unreacted starting material is preferred over enforcing full substrate conversion at the expense of more side products.

**Scheme 3:**
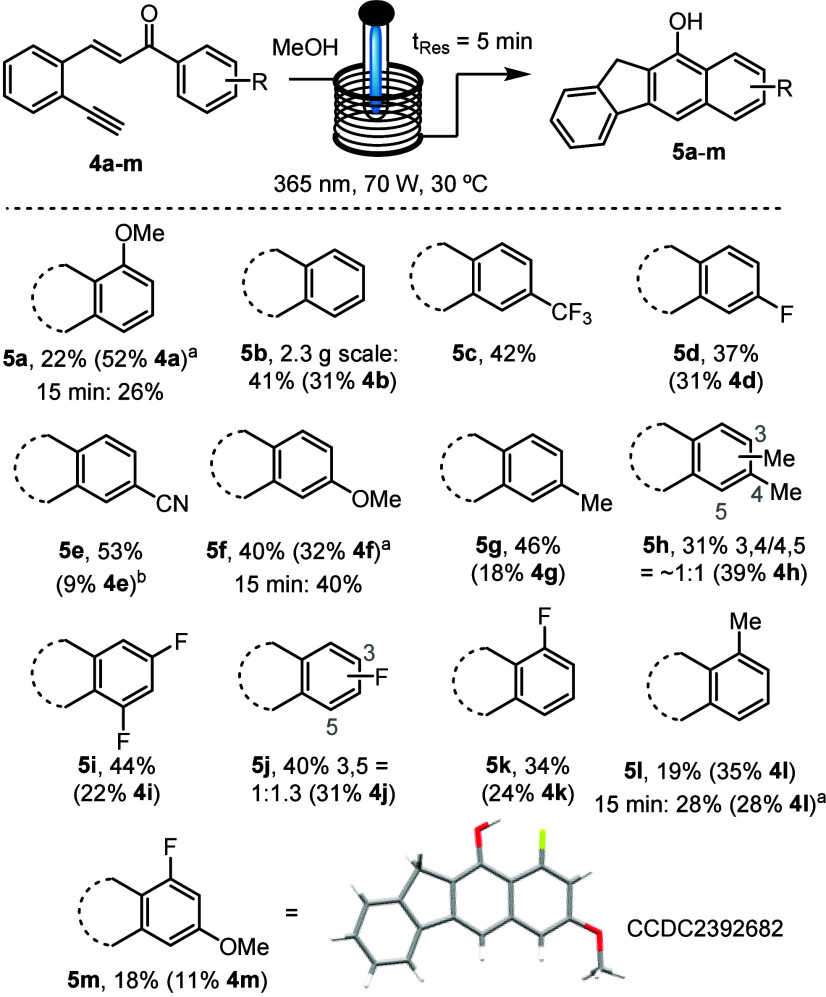
Substrate Scope of Benzo[*b*]fluorenes ^1^H NMR yield using trichloroethylene as the internal standard. HFIP was used as the solvent. Reactions were performed at 50 mM. Reported yields are isolated yields with amounts of recovered starting material in parentheses.

To further expand the scope of the accessible benzo[*b*]fluorenes, we switched our attention to the left-hand aryl-ring-bearing electronically differentiated groups (Scheme 4). A trend emerged showing slightly lower yields of 28–35%, whereby no or very little starting material was recovered.

**Scheme 4:**
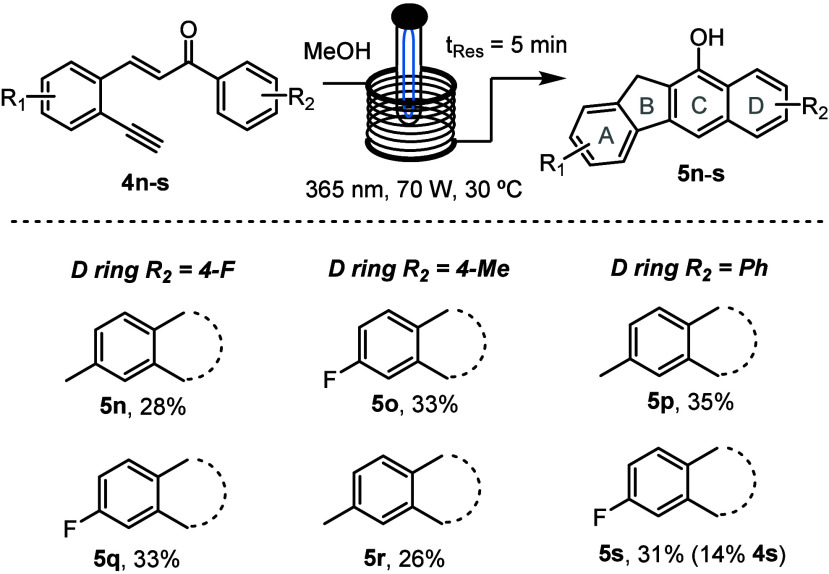
Substrate Scope of Benzo[*b*]fluorenes with Modified A and D Rings Reactions were performed at 50 mM. Reported yields are isolated yields (% starting material recovered).

To gain mechanistic insights into the formation of the benzo[*b*]fluorene products, a set of control experiments were conducted as summarized in Scheme 5. Using the standard conditions for the synthesis of compound **5b** in the presence of (2,2,6,6-tetramethylpiperidin-1-yl)oxyl (TEMPO, 1 equiv) as a radical scavenger fully prevented product formation, and only isomerization of the starting material was observed by ^1^H NMR. Analyzing a reaction sample by high-resolution mass spectrometry (HR-MS) indicated that an adduct with TEMPO had formed, which agrees with radicals being formed under the reaction conditions. When the photochemical reaction was carried out using *d*_4_-methanol as the solvent, the expected benzo[*b*]fluorene product **5b** was isolated in 40% yield along with 20% of the *Z* isomer of the substrate; however, deuterium was not incorporated in these structures. Using a deuterated alkyne derivative (**4b**-**D**_**1**_) generated the expected product (5b-D_1_) bearing the deuterium label *para* to the phenolic group, indicating that no proton transfer occurs at the alkyne position. Lastly, when a fully deuterated benzoyl variant of the substrate (**4b**-**D**_**5**_) was subjected to the standard conditions, the corresponding penta-deuterated benzo[*b*]fluorene product (5b-D_5_) was obtained having four deuterium atoms remaining on this aryl ring, whereas one deuterium is observed in the benzylic position. This key finding, which indicates fragmentation of an *ortho* C–D bond on the arene, was supported by various NMR techniques, including ^13^C and heteronuclear single-quantum correlation (HSQC) data, that show a triplet with a coupling constant of 20 Hz at δ = 32.5 ppm (see the Supporting Information for full details).

**Scheme 5:**
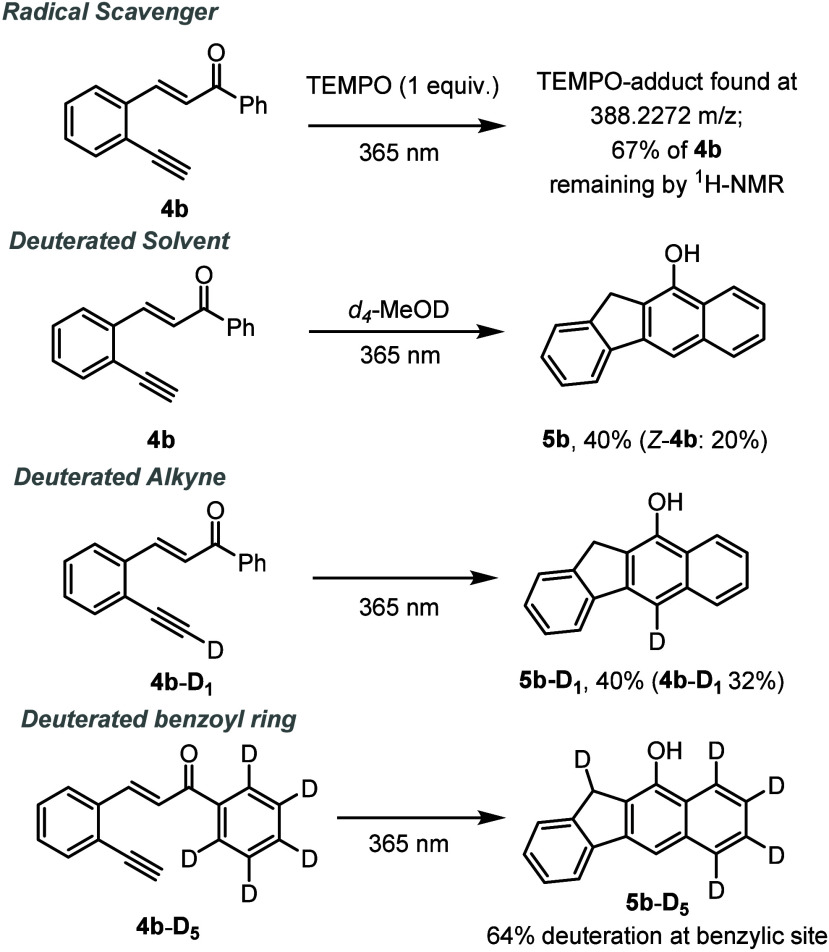
Control Experiments and Deuterium-Labeling Studies under Standard Conditions (70 W and *t*_res_ of 5 min)

On the basis of these experimental data, we propose the mechanism shown in Scheme 6. Initially, upon irradiation of the substrate, the π bond of the alkene undergoes homolysis to form a diradical species (**Int1**), which is intercepted by the alkyne through a 5-*exo*-*dig* cyclization. Rotation across the σ bond of the benzoyl moiety in the resulting intermediate (**Int2**) positions the arene ring next to the benzylic radical, which, in turn, allows for a 1,5-hydrogen atom transfer to occur.^13^ The formation of intermediate **Int3** is supported by our deuteration studies (see part 4 of Scheme 5). Back-rotation of the benzoyl group allows for facile carbon–carbon bond formation by radical recombination, generating the tetracyclic ring system (**Int4**), which subsequently tautomerizes to give the final benzo[*b*]fluorene scaffold.

**Scheme 6:**
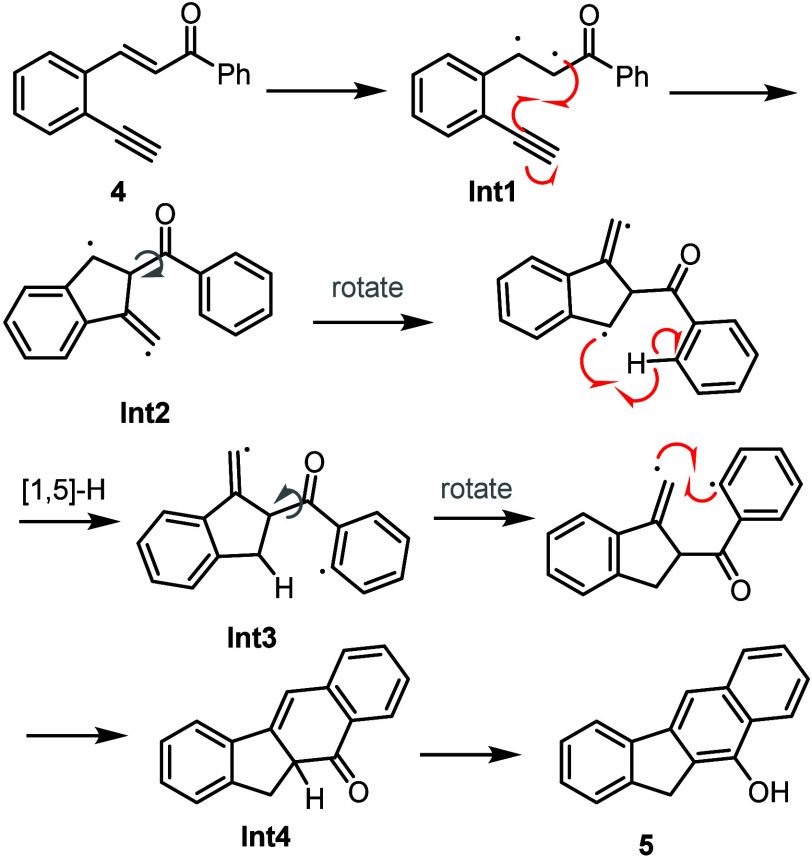
Proposed Reaction Mechanism

Lastly, we evaluated the potential value of benzo[*b*]fluorenes obtained toward further functionalization reactions. As shown in Scheme 7, this demonstrated that common bromination reactions are effective in generating derivatives **6a**, **6d**, and **6e** in a high yield. Moreover, it was found that alkylation of the phenolic position under basic conditions gives compound **6b**, which is accompanied by aerobic oxidation at the benzylic site rendering ketone product **6c**, both of which are competent products for further functionalization giving derivatives **6d**–**6f**.

**Scheme 7:**
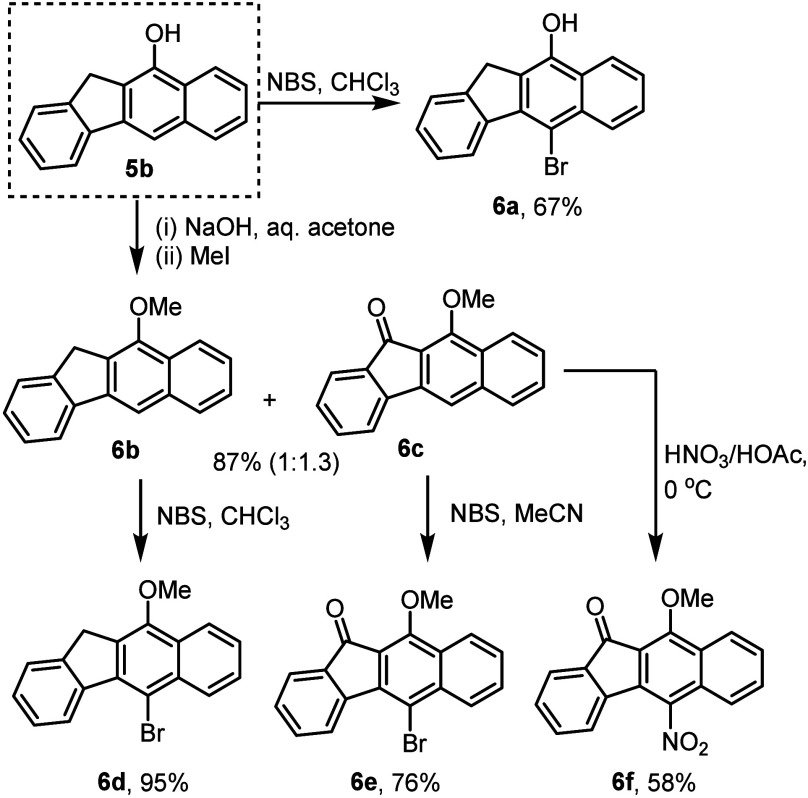
Benzo[*b*]fluorene Derivatization Studies

In conclusion, we report the discovery of a new route to substituted benzo[*b*]fluorenes from simple alkyne-bearing chalcones via a unique photochemical transformation. This process is triggered by irradiation of alcoholic solutions of the chalcone substrates using ultraviolet A (UV-A) light (ca. 365 nm, 70 W), affording the desired products in only 5 min. The scalability of this reaction was demonstrated using a continuous flow reactor setup rendering the desired products in good yields, with small amounts of substrates recoverable. Various substitution patterns are tolerated providing valuable benzo[*b*]fluorene products that can be derivatized by bromination, nitration, oxidation, and alkylation reactions. Overall, this new method not only affords an attractive reagent-free route yielding diverse sets of benzo[*b*]fluorene products but also showcases the value of photochemical flow processes for the discovery of new reactions and their mechanistic investigation.

## Data Availability

The data underlying this study are available in the published article and its Supporting Information.

## Supporting Information Available

Complete experimental procedures, compound characterization, and pictures of the flow equipment (PDF)
FAIR data, including the primary NMR FID files, for compounds **S1**–**S3**, **4b**-**D**_**1**_, **4b**-**D**_**5**_, **5b**-**D**_**1**_, **5b**-**D**_**5**_, **4a**–**4s**, **5a**–**5s**, and **6a**–**6f** (ZIP)
