# Supplementary material for: Direct Photochemical Synthesis of Substituted Benzo[b]fluorenes
Source: Org Lett. 2024 Nov 25;26(48):10364–8. doi: 10.1021/acs.orglett.4c03978 (PMC11629382; doi:10.1021/acs.orglett.4c03978)

Direct Photochemical Synthesis of Substituted Benzo[*b*]fluorenes

Ruairi Crawford,[a] Yannick Ortin,[a] Brendan Twamley,[b] and Marcus Baumann[a,*]

Email: marcus.baumann@ucd.ie

[a] University College Dublin, School of Chemistry, Science Centre South, D04N2E5, Dublin, Ireland; [b] School of Chemistry, Trinity College Dublin, Dublin 2, Ireland

Contents

General Materials and Methods 3

Experimental Procedures 4

Experimental procedures for control experiments 7

Optimisation Table for the Synthesis of *11*H-Benzo[*b*]fluoren-10-ol (5b) 10

Set-up for photochemical reactions 11

Characterisation of Synthesised Aldehydes 13

Characterisation of Deuterated Products 14

Characterisation of Aldol Products 16

Characterisation of 11*H*-Benzo[*b*]fluorenes 26

Functionalisation of 11*H*-Benzo[*b*]Benzofluoren-10-ol 37

X-Ray data 40

Structure Tables 41

Hydrogen bonding for 5a 42

Hydrogen bonding for 5m 43

References 44

Copies of NMR Spectra 45

Copy of TEMPO Control HRMS Data 109

Copy of Deuterated Products HRMS Data 109

General Materials and Methods

Substrates, reagents, and solvents were used as purchased without further purification. ^{1}H-NMR spectra were recorded at 25 °C using Varian VNMRS 400, 500 and Agilent DD2 500 MHz spectrometers. Deuterated solvents acquired from Sigma-Aldrich and Apollo Scientific were used as supplied. Spectra recorded in ppm using the chosen solvent peak as a reference ($CHCl_3$ δ_H = 7.27 ppm) or DMSO-*d6* (δ_H = 2.50 ppm) or acetone-*d6* (δ_H = 2.05 ppm) or MeCN-*d3* (1.94 ppm). Data for ^{1}H-NMR are reported as follows: chemical shift (δ/ppm) (multiplicity, coupling constant (Hz), integration).^{13}C[^{1}H] NMR spectra were recorded on the same instruments (100 and 125 MHz) and are reported relative to $CHCl_3$ (77.0 ppm) or DMSO-*d6* (39.5 ppm), acetone-*d6* (206.3 ppm), MeCN-*d3* (118.3 ppm). ^{19}F-NMR were recorded at 376 MHz. ^{2}H-NMR were recorded at 77 MHz. Multiplicities are reported as follows: s = singlet, d = doublet, t = triplet, q = quartet, m = multiplicity (C, CH, CH_2, CH_3). Experiments were used in the structural assignment.

IR spectra were obtained by use of a Platinum spectrometer (near, ATR sampling, Bruker, Billercia, MA, USA) with intensities of the characteristic signals as reported as weak (w, <20% of tallest signal), medium (m, 21-70% of tallest signal) or strong (s, >71% of tallest signal).

High-resolution mass spectrometry was performed using the indicated techniques on a micromass LCT orthogonal time of flight mass spectrometer and quadropole time-of-flight mass spectrometer with leucine-enkephalin (Tyr-Gly-Phe-Leu) as internal lock mass.

Melting points were recorded on Stuart SMP10 melting point apparatus.

Flow reactions were performed using a Vapourtec E-Series UV-150 photoflow reactor, containing a high-power LED (365 nm, 400 nm, 420 and 450 nm) and a reactor coil of 10 mL volume (FEP tubing).

TLC was performed on Merck pre-coated Silica gel 60 F254 aluminium plates with realisation by UV irradiation at 254nm, $KMnO_4$. Flash chromatography was performed using Macherey-Nagel silica gel 60 M, with a particle range of 0.04 - 0.063 mm.

Experimental Procedures

General procedure A - synthesis of 2-((trimethylsilyl)ethynyl)benzaldehydes (S1-2)

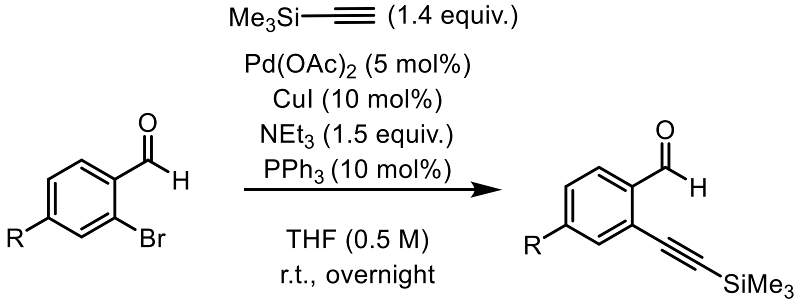

To the appropriate bromobenzaldehyde (1 equiv.) dissolved in dry THF (0.5 M) was added in quick succession trimethylsilylacetylene (1.4 equiv.), Et_3N (1.5 equiv.), $Pd(OAc)_2$ (5 mol%), CuI (10 mol%) and PPh_3 (10 mol%). The reaction was allowed to stir overnight. The crude product was filtered through a pad of silica before all volatiles were evaporated. The resulting crude product was purified by SiO_2 column chromatography using EtOAc/cyclohexane (1-10%).

General procedure B - synthesis of aldol products (4a-s)

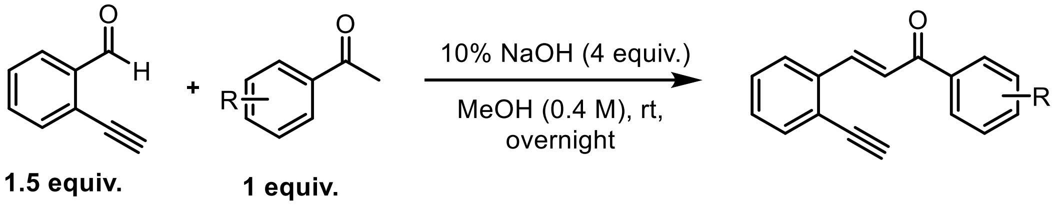

Following a modified previously reported procedure.[1] A mixture of 2-ethynylbenzaldehyde or 2-((trimethylsilyl)ethynyl)benzaldehyde (1.5 equiv.) and the appropriate acetophenone (1 equiv.) in MeOH (0.5 M) was cooled to 0 °C. 10% NaOH solution (4 equiv.) was added dropwise to the mixture. The mixture was then allowed to warm to room temperature and stir overnight. The mixture was then diluted with deionised water and the organic layer was extracted with EtOAc. The combined organic layers were washed with brine and dried with Na_2SO_4. The solvent was evaporated in vacuo to afford the crude product. The crude material was purified by SiO_2 column chromatography using EtOAc/cyclohexane (1-10%).

General procedure C - synthesis of Benzo[*b*]fluorenes (5a-s)

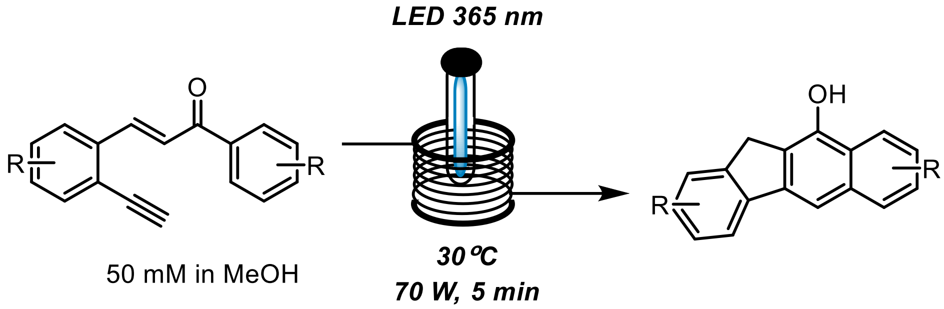

A solution of (*E*)-3-(2-ethynylphenyl)-1-phenylprop-2-en-1-one in MeOH (0.5 M) was prepared, the solvent was degassed and purged with N_2. The solution was passed through the UV-150 Vapourtec photoreactor equipped with 365 nm LED (70 W) with a residence time of 5 min. The reaction mixture was collected, the solvent was evaporated in vacuo to afford the crude product. The crude material was analysed by ^{1}H-NMR using trichloroethylene as internal standard.

General procedure D - bromination of Benzo[*b*]fluorenes (6a, 6d-e)

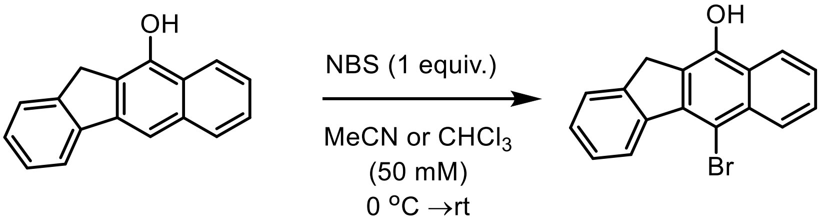

A solution of the appropriate *11*H-benzo[*b*]fluoren-10-ol (**5b**) or 10-methoxy-11*H*-benzo[*b*]fluorene (**6b**) or 10-methoxy-11*H*-benzo[b]fluoren-11-one (**6c**, 1 equiv.) was dissolved in $CHCl_3$ or MeCN (50 mM) and cooled to 0 °C. NBS (1 equiv.) was added in small portions and the reaction was allowed to warm to room temperature and stir for 2 hours. The mixture was then diluted with deionised water and the organic layer was extracted with EtOAc. The combined organic layers were washed with brine and dried with Na_2SO_4. The solvent was evaporated in vacuo to afford the crude product. The crude material was purified by SiO_2 column chromatography using EtOAc/cyclohexane (1-10%).

Procedure for methylation of *11*H-Benzo[*b*]fluoren-10-ol (6b-c)

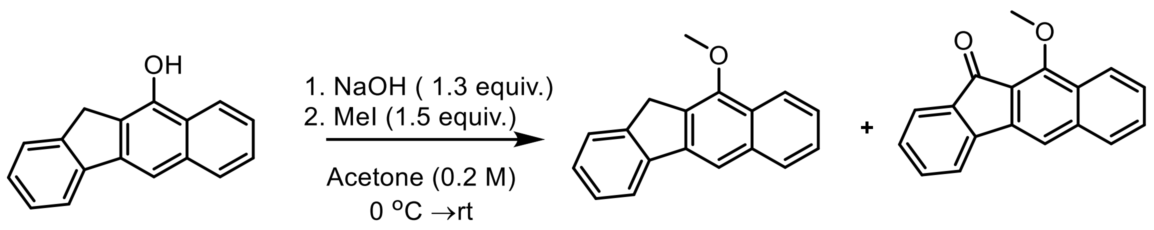

To a solution of *11*H-benzo[*b*]fluoren-10-ol (**5b**) in acetone (0.2 M) was cooled to 0 °C and NaOH (1.3 equiv.) was added. The reaction was allowed to warm to room temperature and stir for 30 minutes. Iodomethane (1.5 equiv.) was added dropwise to the reaction mixture and was stirred for 3 hours. The mixture was then diluted with deionised water and the organic layer was extracted with EtOAc. The combined organic layers were washed with brine and dried with Na_2SO_4. The solvent was evaporated in vacuo to afford the crude product. The crude material was purified by SiO_2 column chromatography using EtOAc/cyclohexane (1-10%).

Procedure for nitration of 10-Methoxy-11*H*-benzo[b]fluoren-11-one (6f)

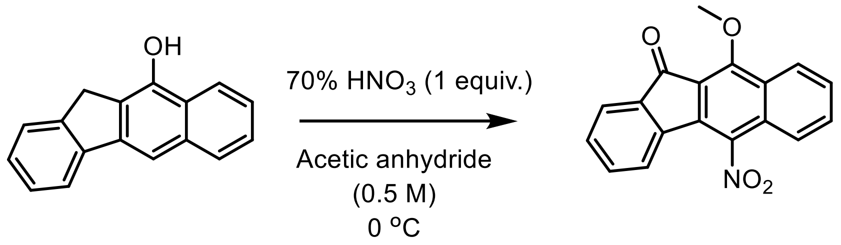

Following a modified previously reported procedure.[2] To a solution of 10-methoxy-11*H*-benzo[*b*]fluoren-11-one (**6c**, 1 equiv.) and acetic anhydride (0.5 M) was cooled to 0 °C. 70% Nitric acid (1 equiv.) was added dropwise to the reaction mixture. The reaction was allowed to stir for 2 hours at 0 °C. Then was poured onto ice-water and the organic layer was extracted with EtOAc. The combined organic layers were washed with brine and dried with Na_2SO_4. The solvent was evaporated in vacuo to afford the crude product. The crude material was purified by SiO_2 column chromatography using EtOAc/cyclohexane (1-10%).

Experimental procedures for control experiments

Procedure for synthesis of (*E*)-3-(2-ethynylphenyl)-1-(phenyl-*d_5*)prop-2-en-1-one (4b-D_5)

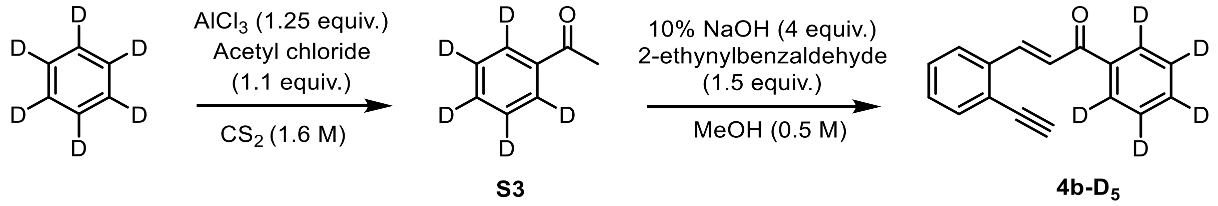

1-(Phenyl-d_5)ethan-1-one (**S3**) was synthesised following a previously reported method.[3] A mixture of 2-ethynylbenzaldehyde (1.5 equiv.) and 1-(phenyl-d_5)ethan-1-one (**S3**, 1 equiv.) in MeOH (0.5 M) was cooled to 0 °C. 10% NaOH solution (4 equiv.) was added dropwise to the mixture. The mixture was then allowed to warm to room temperature and stir overnight. The mixture was then diluted with deionised water and the organic layer was extracted with EtOAc. The combined organic layers were washed with brine and dried with Na_2SO_4. The solvent was evaporated in vacuo to afford the crude product. The crude material was purified by SiO_2 column chromatography using EtOAc/cyclohexane (1-10%).

Procedure for synthesis of (*E*)-3-(2-(ethynyl-d_1)phenyl)-1-phenylprop-2-en-1-one (4b-D_1)

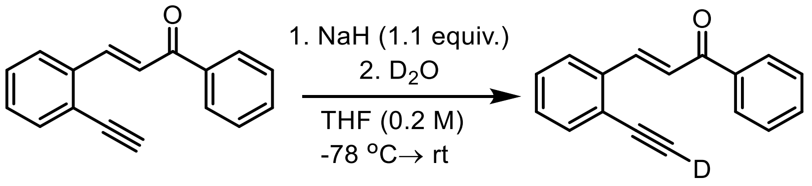

A solution of (*E*)-3-(2-ethynylphenyl)-1-phenylprop-2-en-1-one (**4b**) was dissolved in THF (0.2 M) and cooled to -78 °C. NaH (1.1 equiv.) was added and the reaction mixture was allowed to for 30 minutes. The reaction was allowed to warm to room temperature and was quenched with D_2O. The organic layer was extracted with Et_2O. The solvent was evaporated in vacuo to afford the crude product.

Control procedure for radical scavenger experiment for synthesis of 11*H*-benzo[*b*]fluoren-10-ol (5b)

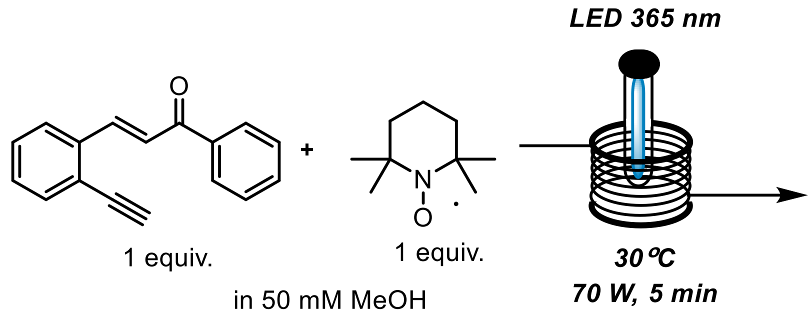

A solution of (*E*)-3-(2-ethynylphenyl)-1-phenylprop-2-en-1-one (**4b**) in MeOH (0.5 M) was prepared, TEMPO (1 equiv.) was added, the solvent was degassed and purged with N_2. The solution was passed through the UV-150 Vapourtec photoreactor equipped with 365 nm LED (70 W) with a residence time of 5 min. The reaction mixture was collected, the solvent was evaporated in vacuo to afford the crude product. The crude material was analysed by ^{1}H-NMR and submitted for HR-MS analysis.

Control procedure for synthesis of 11*H*-benzo[*b*]fluoren-10-ol (5b) in *d*-MeOH

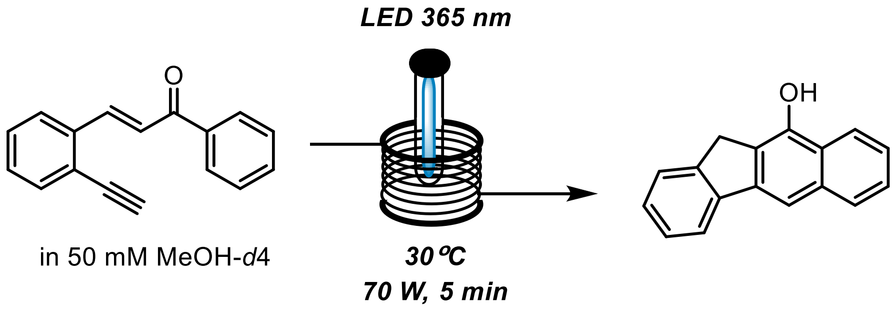

A solution of (*E*)-3-(2-ethynylphenyl)-1-phenylprop-2-en-1-one (**4b**) in *d*-MeOH (0.5 M) was prepared, the solvent was degassed and purged with N_2. The solution was passed through the UV-150 Vapourtec photoreactor equipped with 365 nm LED (70 W) with a residence time of 5 min. The reaction mixture was collected, the solvent was evaporated in vacuo to afford the crude product. The crude material was purified by SiO_2 column chromatography using EtOAc/cyclohexane (1-10%).

Control procedure for synthesis of 11*H*-benzo[*b*]fluoren-5-d-10-ol (5b-D$_1$)

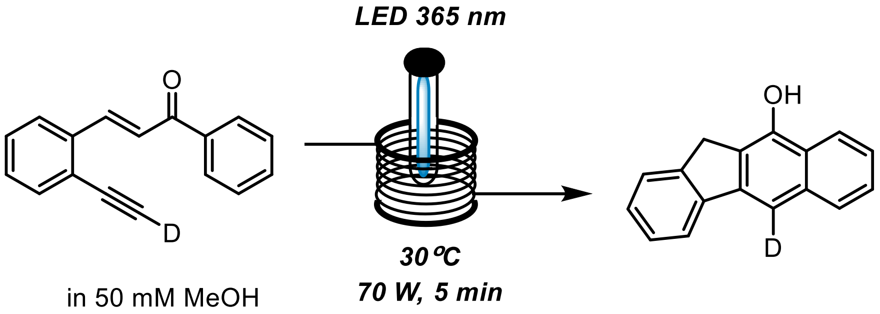

A solution of (*E*)-3-(2-(ethynyl-d)phenyl)-1-phenylprop-2-en-1-one (**4b-D$_1$**) in MeOH (0.5 M) was prepared, the solvent was degassed and purged with N_2. The solution was passed through the UV-150 Vapourtec photoreactor equipped with 365 nm LED (70 W) with a residence time of 5 min. The reaction mixture was collected, the solvent was evaporated in vacuo to afford the crude product. The crude material was purified by SiO_2 column chromatography using EtOAc/cyclohexane (1-10%).

Control procedure for synthesis of 11*H*-benzo[*b*]fluoren-6,7,8,9,11-d5-10-ol (5b-D$_5$)

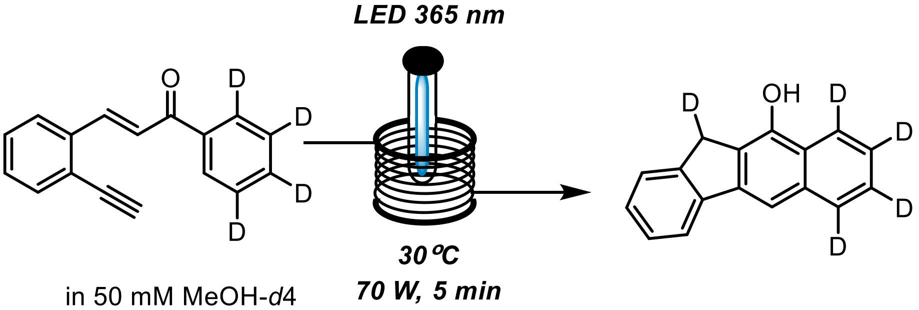

A solution of (*E*)-3-(2-Ethynylphenyl)-1-(phenyl-d5)prop-2-en-1-one (**4b-D$_5$**) in *d$_4$*-MeOH (0.5 M) was prepared, the solvent was degassed and purged with N_2. The solution was passed through the UV-150 Vapourtec photoreactor equipped with 365 nm LED (70 W) with a residence time of 5 min. The reaction mixture was collected, the solvent was evaporated in vacuo to afford the crude product. The crude material was purified by SiO_2 column chromatography using EtOAc/cyclohexane (1-10%).

Optimisation Table for the Synthesis of *11*H-Benzo[*b*]fluoren-10-ol (5b)

An optimisation study was completed to optimise the synthesis of **5b**, only 3 variables were focussed on which were Time (min), Power (Watt) and Concentration (mM). (Note - HFIP was used for optimisation study).

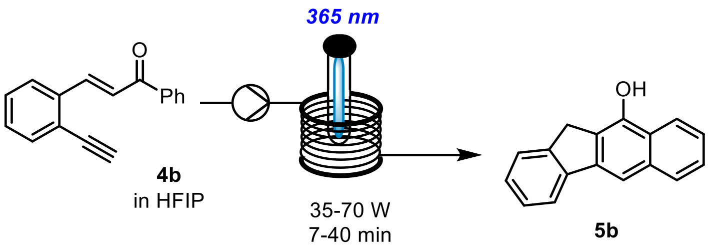

Entry	Concentration (mM)	Power (Watt)	Time (min)	Yield of **5b** (%)
**1**	30	52.5	23.5	33
**2**	50	35	7	35% (SM 33%)
**3**	50	70	7	47%
**4**	50	70	40	28%
**5**	10	35	7	48%
**6**	50	35	40	40%
**7**	10	70	40	0%
**8**	10	70	7	37%
**9**	10	35	40	25%
**10**	50	35	15	47%
**11**	**50**	**70**	**5**	**49% (SM 16%)**
**12**	50	70	2.5	36% (SM 39%)

Set-up for photochemical reactions

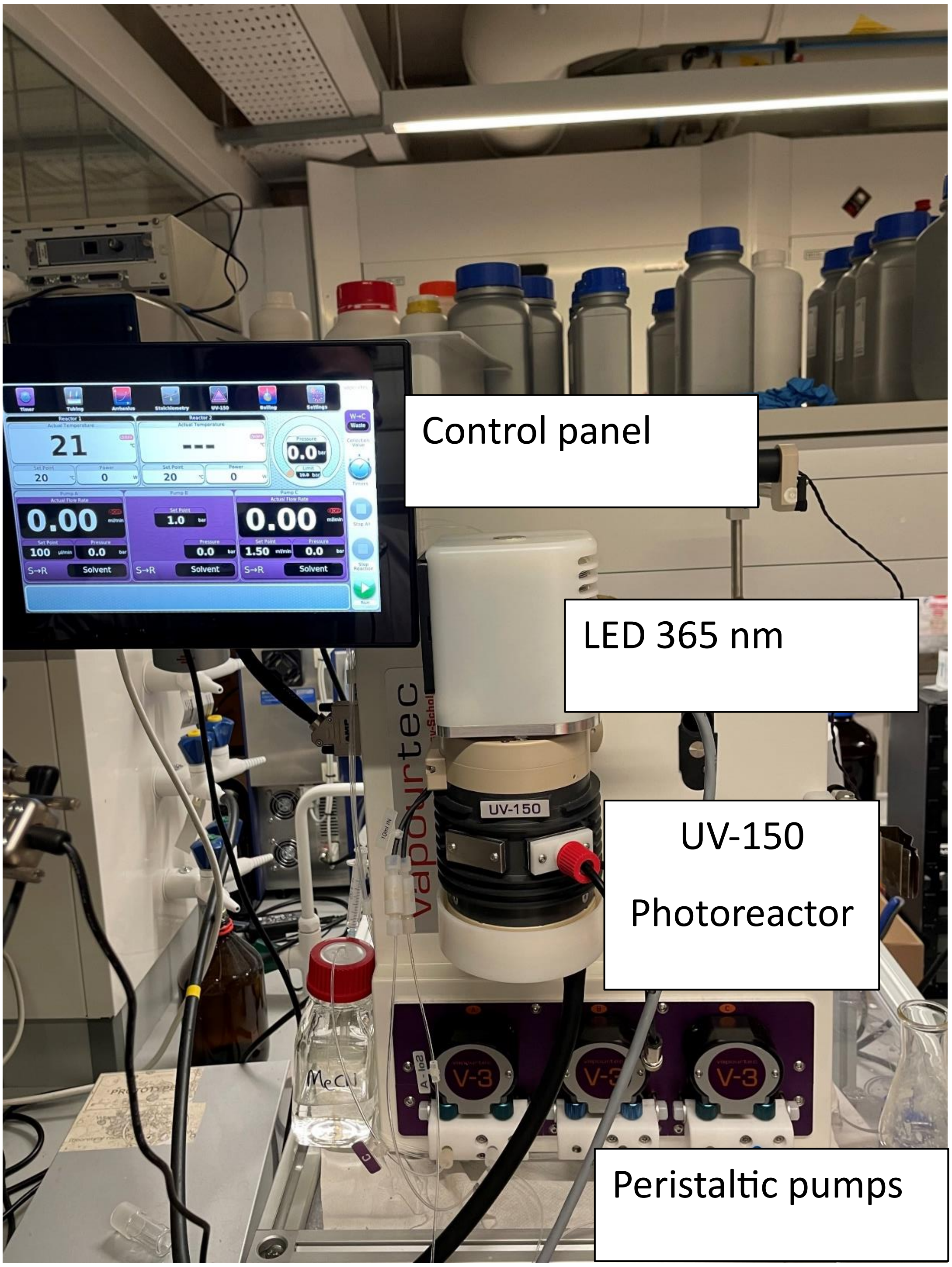

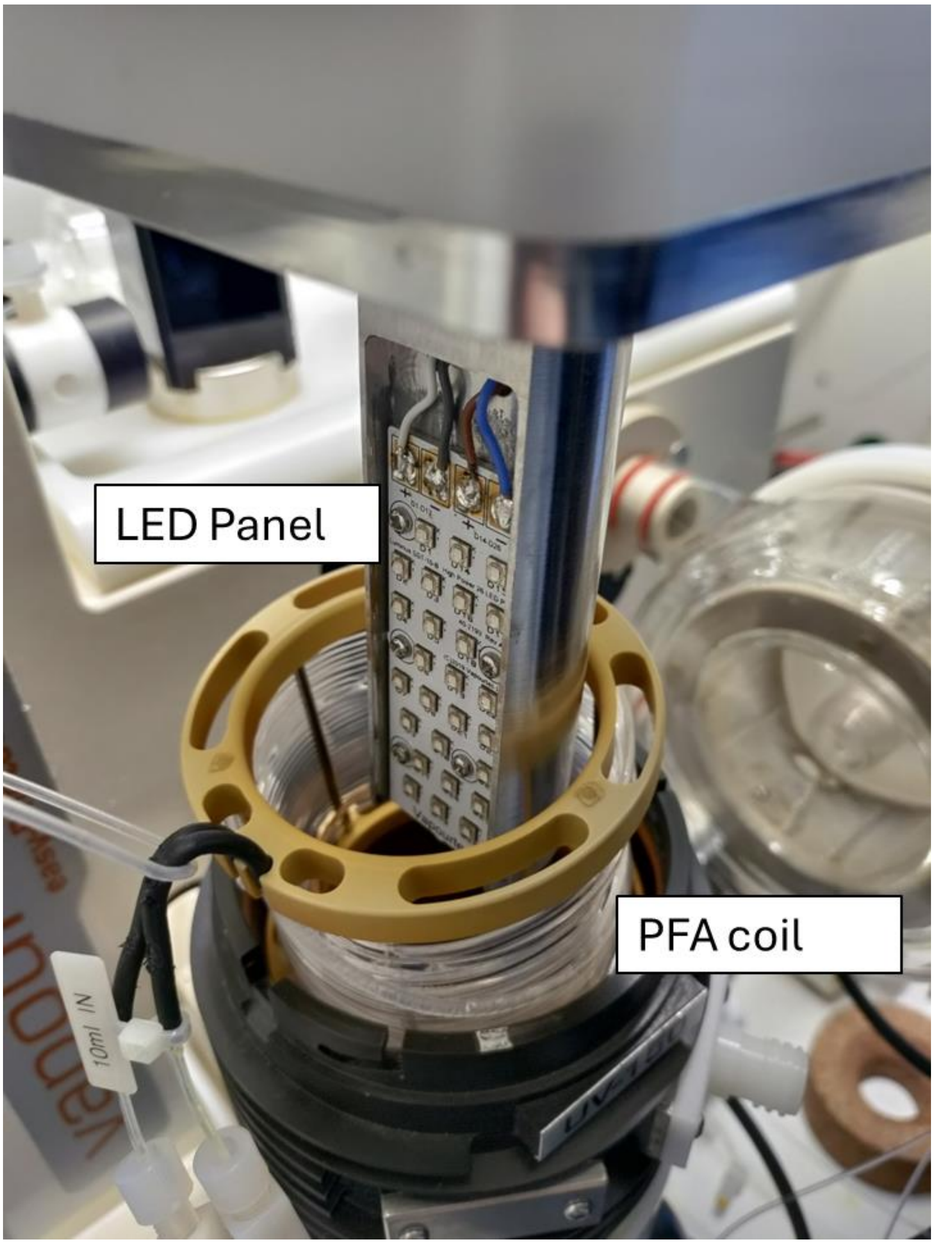
LED Panel
PFA coil
10ml IN

Characterisation of Synthesised Aldehydes

4-Methyl-2-((trimethylsilyl)ethynyl)benzaldehyde (S1)

Synthesised via Procedure A

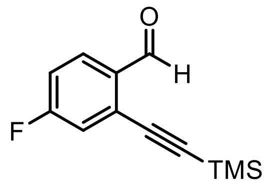

Chemical Formula: $C_{12}H_{13}FOSi$
Exact Mass: 220.0720

Yield: 86% (2.8 g, 12.7 mmol)

Appearance: Brown solid

^{1}H-NMR (400 MHz, CDCl$_3$) δ/ppm 10.46 (d, *J* = 1 Hz, 1H), 7.93 (dd, *J* = 8.7, 5.8 Hz, 1H), 7.24 (dd, *J* = 8.9, 2.5 Hz, 1H), 7.12 (dddd, *J* = 8.8, 8.0, 2.5, 1 Hz, 1H), 0.28 (s, 9H). **^{13}C-NMR (100 MHz, CDCl$_3$)** δ/ppm 190.1 (C), 165.5 (d, *J* = 250 Hz, C), 132.8 (d, *J* = 3 Hz, C), 129.6 (d, *J* = 10 Hz, CH), 129.1 (d, *J* = 11 Hz, C), 119.9 (d, *J* = 24 Hz, CH), 116.7 (d, *J* = 22 Hz, CH), 103.8 (C), 98.6 (d, *J* = 3 Hz, C), -0.42 (3CH$_3$). **^{19}F-NMR (376 MHz, CDCl$_3$) δ/ppm -**103.3 (td, *J* = 8.5, 5.8 Hz). In agreement with previously reported.[4]

4-methyl-2-((trimethylsilyl)ethynyl)benzaldehyde (S2)

Synthesised via Procedure A

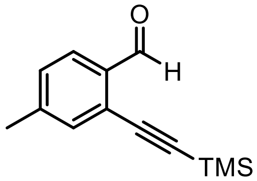

Chemical Formula: $C_{13}H_{16}OSi$
Exact Mass: 216.0970

Yield: 75% (1.6 g, 7.40 mmol)

Appearance: Brown solid

^{1}H-NMR (400 MHz, CDCl$_3$) δ/ppm 10.49 (s, 1H), 7.81 (d, *J* = 8.0 Hz, 1H), 7.39 (s, 1H), 7.24 (ddt, *J* = 8.1, 1.8, 1 Hz, 1H), 2.39 (s, 3H), 0.27 (s, 9H). **^{13}C-NMR (100 MHz, CDCl$_3$)** δ/ppm 191.5 (C), 144.7 (C), 134.0 (C), 133.8 (CH), 129.8 (CH), 126.9 (CH), 126.7 (C), 101.8 (C), 100.2 (C), 21.5 (CH$_3$), -0.3 (3CH$_3$).

Characterisation of Deuterated Products

1-(Phenyl-d_5)ethan-1-one (S3)

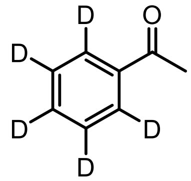

Chemical Formula: $C_8H_3D_5O$
Exact Mass: 125.0889

Yield: 75% (1.6 g, 7.40 mmol)

Appearance: Orange liquid

^{1}H- NMR (500 MHz, $CDCl_3$) δ/ppm 2.61 (s, 3H). **^{13}C-NMR (125 MHz, $CDCl_3$)** δ/ppm 198.1, 136.9 (C), 133.4 – 131.4 (m, 2C), 128.5 – 127.2 (m, 3C), 26.5 (CH_3).

(*E*)-3-(2-(Ethynyl-d)phenyl)-1-phenylprop-2-en-1-one (4b-D_1)

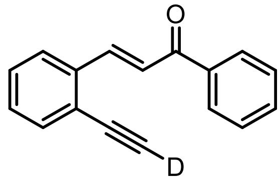

Chemical Formula: $C_{17}H_{11}DO$
Exact Mass: 233.0951

Yield: 88% (140 mg, 0.60 mmol)

Appearance: Orange waxy solid

^{1}H-NMR (400 MHz, $CDCl_3$) δ/ppm 8.27 (d, *J* = 15.9 Hz, 1H), 8.07 – 8.00 (m, 2H), 7.76 (dd, *J* = 7.7, 1.5 Hz, 1H), 7.67 – 7.57 (m, 3H), 7.55 – 7.49 (m, 2H), 7.44 – 7.33 (m, 2H). **^{13}C-NMR (125 MHz, $CDCl_3$)** δ/ppm 194.4 (C), 137.9 (CH), 136.9 (C), 133.0 (CH), 132.7 (CH), 129.1 (C), 128.9 (CH), 128.4 (2CH), 128.4 (2CH), 128.3 (CH), 128.2 (CH), 128.0 (CH), 121.5 (C), two carbon resonances not observed. **IR (neat)** v/cm^{-1}: 3061 (w), 2581 (w), 1664 (m), 1579 (w), 1225 (m), 1004 (w), 902 (s), 727 (s), 649 (w). **HR-MS (QTOF ES+):** calcd for $C_{17}H_{12}DO$ 234.1024 found 234.1025 (M+H$^+$).

(*E*)-3-(2-Ethynylphenyl)-1-(phenyl-d5)prop-2-en-1-one (4b-D_5)

Synthesised via Procedure B

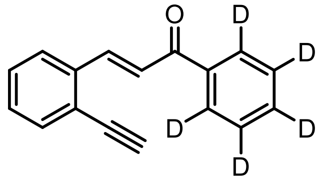

Chemical Formula: $C_{17}H_7D_5O$
Exact Mass: 237.1202

Yield: 75% (216 mg, 0.91 mmol)

Appearance: Orange solid

^{1}H-NMR (400 MHz, $CDCl_3$) δ/ppm 8.27 (d, *J* = 15.9 Hz, 1H), 7.79 – 7.70 (m, 1H), 7.62 (d, *J* = 15.9 Hz, 1H), 7.59 – 7.56 (m, 1H), 7.43 – 7.38 (m, 1H), 7.36 (td, *J* = 7.5, 1.5 Hz, 1H), 3.44 (s, 1H). **^{13}C-NMR (100 MHz, $CDCl_3$)** δ/ppm 190.6 (C), 142.2 (CH), 133.6 (CH), 137.9 (C), 136.9 (C), 129.7 (CH), 129.0 (CH), 128.55 – 127.43 (m, 5C), 126.4 (CH), 124.1 (CH), 123.2 (C), 83.5 (CH), 81.2 (C). **IR (neat)** ν/cm^{-1}: 3233 (m), 1656 (m), 1588 (s), 11474 (m), 1310 (m), 1176 (s), 1045 (w), 821 (w), 757 (s), 586 (w). **HR-MS (QTOF ES+):** calcd for $C_{17}H_8D_5O$ 238.1275 found 238.1275 ($M+H^+$).

11*H*-Benzo[*b*]fluoren-5-d-10-ol (5b-D_1)

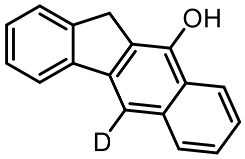

Chemical Formula: $C_{17}H_{11}DO$
Exact Mass: 233.0951

Yield: 24% (35 mg, 0.15 mmol)

Appearance: Brown solid

^{1}H-NMR (500 MHz, MeCN-*d3*) δ/ppm 8.29 – 8.19 (m, 1H), 7.96 – 7.87 (m, 2H), 7.65 – 7.59 (m, 1H), 7.52 – 7.44 (m, 2H), 7.43 – 7.35 (m, 2H), 7.28 (s, 1H), 4.02 (s, 2H). **^{13}C-NMR (125 MHz, MeCN-*d3*)** δ/ppm 148.8 (C), 144.6 (C), 142.2 (C), 142.1 (C), 135.7 (C), 128.8 (CH), 128.6 (CH), 127.9 (CH), 126.7 (CH), 126.4 (CH), 125.4 (CH), 125.3 (C), 123.1 (C), 122.5 (CH), 121.6 (CH), 111.4 (t, *J* = 25 Hz, C), 34.6 (CH_2). **^{2}H-NMR (77 MHz, MeCN)** δ/ppm 7.93. **IR (neat)** ν/cm^{-1}: 3380 (m), 1664 (w), 1573 (w), 1500 (w), 1262 (m), 1084 (m), 985 (m), 760 (s), 721 (s), 652 (w). **HR-MS (QTOF ES+):** calcd for $C_{17}H_{12}DO$ 234.1024 found 234.1024 ($M+H^+$).

11*H*-Benzo[*b*]fluoren-6,7,8,9,11-d5-10-ol (5b-D$_5$)

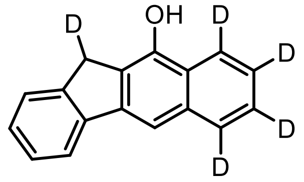

Chemical Formula: $C_{17}H_7D_5O$
Exact Mass: 237.1202

Yield: 23% (29 mg, 0.12 mmol)

Appearance: Orange solid

^{1}H-NMR (500 MHz, CDCl$_3$) δ/ppm 7.90 (dt, *J* = 7.7, 1.0 Hz, 1H), 7.85 (s, 1H), 7.57 (dq, *J* = 7.4, 1.0 Hz, 1H), 7.43 (tt, *J* = 7.5, 1.0 Hz, 1H), 7.36 (td, *J* = 7.4, 1.2 Hz, 1H), 3.93 (t, *J* = 2.8 Hz, 1H). **^{13}C-NMR (125 MHz, CDCl$_3$)** δ/ppm 146.8 (m, C), 142.8 (m, C), 141.4 (C), 141.2 (C),134.6 (C), 127.5 (CH), 127.0 (m, CH), 125.3 (m, CH), 123.7 (C), 120.7 (m, CH), 111.1 (CH), 32.5 (t, *J* = 20 Hz, CH), 4 carbon resonances not observed. **^{2}H-NMR (77 MHz, CHCl$_3$)** δ/ppm 8.24, 7.96, 7.54, 3.94. **IR (neat)** v/cm^{-1}: 3286 (w), 2921 (w), 1631 (m), 1554 m), 1741 (w), 1460 (w), 1243 (m), 1139 9m), 860 (w), 765 (s), 565 (m). **HR-MS (QTOF ES+):** calcd for $C_{17}H_8D_5O$ 238.1275 found 238.1273 (M+H$^+$).

Characterisation of Aldol Products

(*E*)-3-(2-Ethynylphenyl)-1-(2-methoxyphenyl)prop-2-en-1-one (4a)

Synthesised via Procedure B

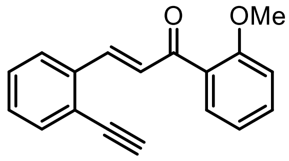

Chemical Formula: $C_{18}H_{14}O_2$
Exact Mass: 262.0994

Yield: 73% (317 mg, 1.21 mmol)

Appearance: Light-yellow solid

^{1}H-NMR (400 MHz, CDCl$_3$) δ/ppm 8.11 (d, *J* = 16.0 Hz, 1H), 7.72 (dd, *J* = 7.7, 1.5 Hz, 1H), 7.64 (dd, *J* = 7.6, 1.8 Hz, 1H), 7.57 – 7.51 (m, 1H), 7.51 – 7.46 (m, 1H), 7.43 (d, *J* = 16.0 Hz, 1H), 7.38 (td, *J* = 7.6, 1.7 Hz, 1H), 7.33 (td, *J* = 7.4, 1.5 Hz, 1H), 7.04 (td, *J* = 7.5, 1.0 Hz, 1H), 7.00 (d, *J* = 8.4 Hz, 1H), 3.90 (s, 3H), 3.37 (s, 1H). **^{13}C-NMR (100 MHz, CDCl$_3$)** δ/ppm 192.9 (C), 158.1 (C), 140.7 (CH), 137.1 (C), 133.4 (CH), 132.9 (CH), 130.4 (CH), 129.5 (CH), 129.1 (C), 129.0 (CH), 128.7 (CH), 126.1 (CH), 123.2 (C), 120.7 (CH), 111.5 (CH), 83.2 (CH), 81.1 (C), 55.7 (CH$_3$). **IR (neat)** v/cm^{-1}: 3216 (w), 1656 (m), 1585 (s), 1477 (m), 1329 (m), 1239 (m), 1114 (w), 1060 (m), 990 (w), 754 (s). **HR-MS (QTOF ES+):** calcd for $C_{18}H_{15}O_2$ 263.1067 found 263.1069 (M+H$^+$).

(*E*)-3-(2-Ethynylphenyl)-1-phenylprop-2-en-1-one (4b)

Synthesised via Procedure B

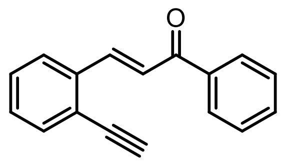

Chemical Formula: $C_{17}H_{12}O$
Exact Mass: 232.0888

Yield: 77% (2.3 g, 9.90 mmol)

Appearance: Light-yellow solid

^{1}H-NMR (400 MHz, $CDCl_3$) δ/ppm 8.27 (d, *J* = 15.8 Hz, 1H), 8.10 – 7.97 (m, 2H), 7.82 – 7.73 (m, 1H), 7.66 – 7.56 (m, 3H), 7.54 – 7.48 (m, 2H), 7.44 – 7.34 (m, 2H), 3.44 (s, 1H). **^{13}C-NMR (125 MHz, $CDCl_3$)** δ/ppm 190.6 (C), 142.3 (CH), 138.0 (C), 136.9 (C), 133.6 (CH), 132.8 (CH), 129.8 (CH), 129.0 (CH), 128.6 (4CH), 126.4 (CH), 124.2 (1CH), 123.2 (C), 83.5 (CH), 81.2 (C). **IR (neat)** v/cm^{-1}: 3234 (m), 2981 (w), 1658 (m), 1589 (m), 1333 (m), 1272 (m), 1046 (m), 979 (m), 736 (s), 687 (m). **HR-MS (QTOF ES+):** calcd for $C_{17}H_{13}O$ 233.0961 found 233.0963 (M+H^{+}).[5]

(*E*)-3-(2-Ethynylphenyl)-1-(4-(trifluoromethyl)phenyl)prop-2-en-1-one (4c)

Synthesised via Procedure B

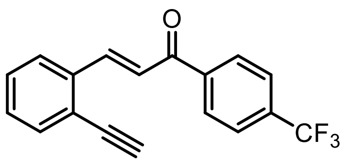

Chemical Formula: $C_{18}H_{11}F_3O$
Exact Mass: 300.0762

Yield: 73% (291 mg, 0.96 mmol)

Appearance: Light-yellow solid

^{1}H-NMR (400 MHz, $CDCl_3$) δ/ppm 8.28 (d, *J* = 15.8 Hz, 1H), 8.17 – 8.04 (m, 2H), 7.81 – 7.75 (m, 3H), 7.64 – 7.51 (m, 2H), 7.40 (pd, *J* = 7.4, 1.6 Hz, 2H), 3.45 (s, 1H). **^{13}C-NMR (100 MHz, $CDCl_3$)** δ/ppm 189.8 (C), 143.5 (CH), 140.9 (CH), 136.4 (C), 134.0 (q, *J* = 32.8 Hz, C), 133.8 (CH), 130.2 (CH), 129.1 (CH), 128.8 (2CH), 126.5 (CH), 125.61 (q, *J* = 4 Hz, CF), 125.0 (C), 123.6 (2CH), 123.4 (C), 83.7 (CH), 81.1 (C). **IR (neat)** v/cm^{-1}: 3255 (w), 2980 (w), 1663 (m), 1590 (m), 1475 (m), 1316 (s), 1158 (s), 1064 (s), 994 (m), 843 (m), 672 (m). **^{19}F-NMR (376 MHz, $CDCl_3$) δ/ppm** -62.9. **HR-MS (QTOF ES+):** calcd for $C_{18}H_{12}F_3O$ 301.0835 found 301.0835 (M+H^{+}).

(*E*)-3-(2-Ethynylphenyl)-1-(4-fluorophenyl)prop-2-en-1-one (4d)

Synthesised via Procedure B

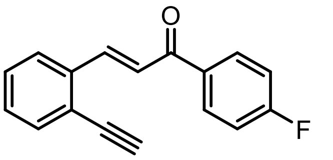

Chemical Formula: $C_{17}H_{11}FO$
Exact Mass: 250.0794

Yield: 82% (447 mg, 1.79 mmol)

Appearance: Light-yellow solid

^{1}H-NMR (400 MHz, CDCl$_3$) δ/ppm 8.25 (d, *J* = 15.8 Hz, 1H), 8.05 (dd, *J* = 8.9, 5.4 Hz, 2H), 7.77 – 7.70 (m, 1H), 7.62 – 7.54 (m, 2H), 7.43 – 7.32 (m, 2H), 7.23 – 7.13 (m, 2H), 3.44 (s, 1H). **^{13}C-NMR (100 MHz, CDCl$_3$)** δ/ppm 188.9 (C), 165.5 (d, *J* = 250 Hz, C), 142.5 (CH), 136.7 (C), 134.3 (d, *J* = 3.0 Hz, C), 133.6 (CH), 131.1 (d, *J* = 9.3 Hz, 2CH), 129.8 (CH), 129.0 (CH), 126.4 (CH), 123.7 (CH), 123.2 (C), 115.6 (d, *J* = 22 Hz, 2CH), 83.5 (CH), 81.2 (C). **^{19}F-NMR (376 MHz, CDCl$_3$) δ/ppm** -105.3 (tt, *J* = 8.5, 5.5 Hz). **IR (neat)** ν/cm^{-1}: 3234 (w), 2980 (m), 1659 (m), 1585 (m), 1474 (w), 1333 (w), 1156 (m), 1021 (w), 837 (m), 761 (s). **HR-MS (QTOF ES+):** calcd for $C_{17}H_{12}FO$ 251.0872 found 251.0867 (M+H$^+$).[6]

(*E*)-4-(3-(2-Ethynylphenyl)acryloyl)benzonitrile (4e)

Synthesised via Procedure B

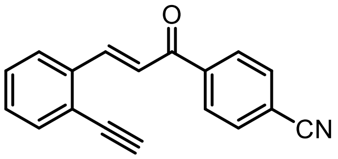

Chemical Formula: $C_{18}H_{11}NO$
Exact Mass: 257.0841

Yield: 57% (189 mg, 0.73 mmol)

Appearance: Light-yellow solid

^{1}H-NMR (400 MHz, CDCl$_3$) δ/ppm 8.28 (d, *J* = 15.9 Hz, 1H), 8.10 – 8.06 (m, 2H), 7.86 – 7.80 (m, 2H), 7.79 – 7.72 (m, 1H), 7.59 (dd, *J* = 7.2, 1.9 Hz, 1H), 7.54 (d, *J* = 15.9 Hz, 1H), 7.47 – 7.36 (m, 2H), 3.45 (s, 1H). **^{13}C-NMR (100 MHz, CDCl$_3$)** δ/ppm 189.4 (C), 144.0 (CH), 141.3 (C), 136.2 (C), 133.8 (CH), 132.4 (2CH), 130.4 (CH), 129.2 (CH) 128.9 (2CH), 126.5 (CH), 123.5 (C), 123.3 (CH), 118.0 (C), 116.0 (C), 83.8 (CH), 81.0 (C). **IR (neat)** ν/cm^{-1}: 3268 (m), 2980 (w), 1641 (s), 1590 (m), 1470 (m), 1277 (s), 1075 (m), 994 (w), 761 (s), 643 (w). **HR-MS (QTOF ES+):** calcd for $C_{18}H_{12}NO$ 258.0913 found 258.0913 (M+H$^+$).

(*E*)-3-(2-Ethynylphenyl)-1-(4-methoxyphenyl)prop-2-en-1-one (4f)

Synthesised via Procedure B

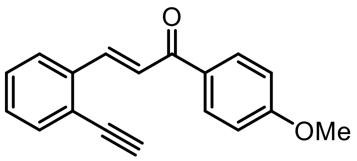

Chemical Formula: $C_{18}H_{14}O_2$
Exact Mass: 262.0994

Yield: 67% (401 mg, 1.53 mmol)

Appearance: Light-yellow solid

^{1}H-NMR (400 MHz, $CDCl_3$) δ/ppm 8.24 (d, *J* = 15.7 Hz, 1H), 8.06 – 8.00 (m, 2H), 7.78 – 7.73 (m, 1H), 7.63 (d, *J* = 15.8 Hz, 1H), 7.59 – 7.53 (m, 1H), 7.46 – 7.30 (m, 2H), 7.03 – 6.93 (m, 2H), 3.88 (s, 3H), 3.44 (s, 1H). **^{13}C-NMR (100 MHz, $CDCl_3$)** δ/ppm 188.7 (C), 163.3 (C), 141.3 (CH), 137.0 (C), 133.6 (CH), 130.9 (C), 130.8 (2CH), 129.5 (CH), 128.9 (CH), 126.3 (2CH), 123.9 (CH), 123.0 (C), 113.7 (CH), 83.3 (CH), 81.2 (C), 55.4 (CH_3). **IR (neat)** ν/cm^{-1}: 3222 (m), 1651 (w), 1588 (s), 1474 (w), 1332 (w), 1220 (m), 980 (m), 833 (m), 760 (s), 585 (w). **HR-MS (QTOF ES+):** calcd for $C_{18}H_{15}O_2$ 263.1067 found 263.1069 ($M+H^+$).[6]

(*E*)-3-(2-Ethynylphenyl)-1-(p-tolyl)prop-2-en-1-one (4g)

Synthesised via Procedure B

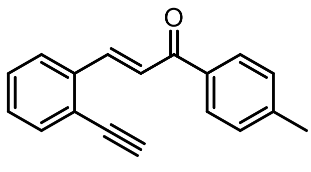

Chemical Formula: $C_{18}H_{14}O$
Exact Mass: 246.1045

Yield: 71% (134 mg, 0.54 mmol)

Appearance: Light-yellow solid

^{1}H-NMR (500 MHz, $CDCl_3$) δ/ppm 8.26 (d, *J* = 15.8 Hz, 1H), 7.94 (d, *J* = 8.1 Hz, 2H), 7.77 – 7.73 (m, 1H), 7.61 (dd, *J* = 15.9, 0.7 Hz, 1H), 7.57 – 7.54 (m, 1H), 7.38 (tt, *J* = 7.9, 1.7 Hz, 1H), 7.36 – 7.31 (m, 1H), 7.30 – 7.27 (m, 2H), 3.44 (s, 1H), 2.42 (s, 3H).**^{13}C-NMR (125 MHz, $CDCl_3$)** 189.9 (C), 143.6 (C), 141.7 (CH), 136.9 (C), 135.4 (C), 133.6 (CH), 129.6 (CH), 129.2 (2CH), 1289.0 (CH), 128.6 (2CH), 126.3 (CH), 124.1 (CH), 123.1 (C), 83.4 (CH), 81.2 (C). **IR (neat)** ν/cm^{-1}: 3279 (w), 1673 (m), 1608 (m), 1334 (w), 1315n (m), 1184 (m), 976 (m), 822 (m), 788 (s), 660 (m). **HR-MS (QTOF ES+):** calcd for $C_{18}H_{15}O$ 247.1117 found 247.1119 ($M+H^+$).

(*E*)-1-(3,4-Dimethylphenyl)-3-(2-ethynylphenyl)prop-2-en-1-one (4h)

Synthesised via Procedure B

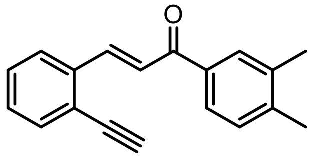

Chemical Formula: $C_{19}H_{16}O$
Exact Mass: 260.1201

Yield: 67% (306 mg, 1.18 mmol)

Appearance: Light-yellow solid

^{1}H-NMR (400 MHz, CDCl$_3$) δ/ppm 8.26 (d, *J* = 15.8 Hz, 1H), 7.83 – 7.72 (m, 3H), 7.61 (d, *J* = 15.9 Hz, 1H), 7.55 (dd, *J* = 7.5, 1.6 Hz, 1H), 7.38 (td, *J* = 7.6, 1.6 Hz, 1H), 7.33 (td, *J* = 7.5, 1.4 Hz, 1H), 7.24 (d, *J* = 7.8 Hz, 1H), 3.45 (s, 1H), 2.33 (s, 3H), 2.32 (s, 3H). **^{13}C-NMR (100 MHz, CDCl$_3$)** δ/ppm 190.1 (C), 142.4 (C), 141.5 (CH), 137.0 (C), 136.9 (C), 135.8 (C), 133.5 (CH), 129.8 (CH), 129.7 (CH), 129.6 (CH), 129.0 (CH), 126.3 (CH), 126.3 (CH), 124.2 (CH), 123.1 (C), 83.4 (CH), 81.3 (C), 20.0 (CH_3), 19.7 (CH_3). **IR (neat)** v/cm^{-1}: 3222 (w), 1660 (s), 1594 (s), 1475 (m), 1333 (w), 1254 (s), 1016 (w), 943 (w), 814 (w). **HR-MS (QTOF ES+):** calcd for $C_{19}H_{17}O$ 261.1274 found 261.1275 (M+H$^+$).

(*E*)-1-(3,5-Difluorophenyl)-3-(2-ethynylphenyl)prop-2-en-1-one (4i)

Synthesised via Procedure B

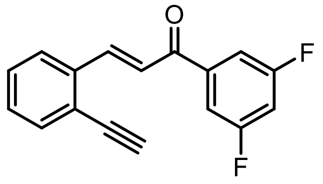

Chemical Formula: $C_{17}H_{10}F_2O$
Exact Mass: 268.0700

Yield: 54% (247 mg, 0.99 mmol)

Appearance: Light-yellow solid

^{1}H-NMR (400 MHz, CDCl$_3$) δ/ppm 8.28 (d, *J* = 15.8 Hz, 1H), 7.74 (dd, *J* = 7.4, 1.7 Hz, 1H), 7.59 (dd, *J* = 7.4, 1.7 Hz, 1H), 7.54 – 7.44 (m, 3H), 7.46 – 7.33 (m, 2H), 7.07 – 6.99 (m, 1H), 3.47 (s, 1H). **^{13}C-NMR (100 MHz, CDCl$_3$)** δ/ppm 188.2 (C), 163.1 (d, *J* = 250 Hz, C), 163.0 (d, *J* = 250 Hz, C), 144.0 (CH), 141.1 (t, *J* = 8 Hz, C), 136.5 (C), 133.9 (CH), 130.3 (CH), 129.2 (CH), 126.7 (CH), 123.5 (C), 123.1 (CH), 111.7 (d, *J* = 7 Hz, CH), 111.5 (d, *J* = 7 Hz, CH), 108.1 (t, *J* = 25 Hz, CH), 83.9 (CH), 81.2 (C). **^{19}F-NMR (376 MHz, CDCl$_3$) δ/ppm** -104.2 (m). **IR (neat)** v/cm^{-1}: 3248 (w), 1702 (m), 1586 (s), 1432 (m), 1300 (m), 1164 (w), 980 (m), 857 (m), 651 (s). **HR-MS (QTOF ES+):** calcd for $C_{17}H_{11}F_2O$ 269.0772 found 269.0772 (M+H$^+$).

(*E*)-3-(2-Ethynylphenyl)-1-(3-fluorophenyl)prop-2-en-1-one (4j)

Synthesised via Procedure B

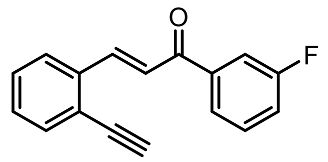

Chemical Formula: $C_{17}H_{11}FO$
Exact Mass: 250.0794

Yield: 54% (247 mg, 0.99 mmol)

Appearance: Light-yellow solid

^{1}H-NMR (400 MHz, CDCl$_3$) δ/ppm 8.24 (dd, *J* = 15.9, 1.7 Hz, 1H), 7.83 (td, *J* = 7.5, 1.8 Hz, 1H), 7.77 – 7.72 (m, 1H), 7.58 – 7.50 (m, 2H), 7.48 (dd, *J* = 15.9, 2.7 Hz, 1H), 7.42 – 7.33 (m, 2H), 7.29 – 7.24 (m, 1H), 7.17 (ddd, *J* = 10.8, 8.3, 1.1 Hz, 1H), 3.42 (s, 1H). **^{13}C-NMR (100 MHz, CDCl$_3$)** δ/ppm 189.4 (d, *J* = 2 Hz, C), 162.8 (d, *J* = 248 Hz, C), 143.1 (CH), 140.2 (d, *J* = 6 Hz, C), 136.7 (C), 133.7 (CH), 130.3 (d, *J* = 8 Hz, CH), 130.1 (CH), 129.1 (CH), 126.5 (CH), 124.2 (d, *J* = 3 Hz, C), 123.7 (CH), 123.3 (CH), 119.8 (d, *J* = 22 Hz, CH), 115.4 (d, *J* = 22 Hz, CH), 83.7 (CH), 81.2 (C).**^{19}F-NMR (376 MHz, CDCl$_3$) δ/ppm** -111.6 (td, *J* = 8.8, 5.5 Hz). **IR (neat)** v/cm^{-1}: 3246 (w), 1678 (m), 1660 (m), 1583 (s), 1432 (w), 1267 (m), 1166 (m), 985 (m), 787 (w), 751 (s). **HR-MS (QTOF ES+):** calcd for $C_{17}H_{12}FO$ 251.0867 found 251.0867 (M+H$^+$).

(E)-3-(2-ethynylphenyl)-1-(2-fluorophenyl)prop-2-en-1-one (4k)

Synthesised via Procedure B

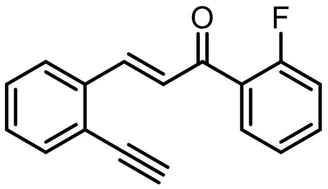

Chemical Formula: $C_{17}H_{11}FO$
Exact Mass: 250.08

Yield: 64% (289 mg, 1.15 mmol)

Appearance: Light-yellow solid

^{1}H-NMR (400 MHz, CDCl$_3$) δ/ppm 8.28 (d, *J* = 15.8 Hz, 1H), 7.80 (ddd, *J* = 7.7, 1.6, 1.0 Hz, 1H), 7.75 (dd, *J* = 7.7, 1.5 Hz, 1H), 7.70 (ddd, *J* = 9.4, 2.6, 1.6 Hz, 1H), 7.61 – 7.58 (m, 1H), 7.56 (d, *J* = 16.0 Hz, 1H), 7.49 (td, *J* = 8.0, 5.5 Hz, 1H), 7.41 (td, *J* = 7.6, 1.6 Hz, 1H), 7.39 – 7.36 (m, 1H), 7.29 (tdd, *J* = 8.2, 2.6, 1.0 Hz, 1H), 3.45 (s, 1H).**^{13}C-NMR (100 MHz, CDCl$_3$)** δ/ppm 189.0 (C), 161.2 (d, *J* = 250 Hz, C), 142.2 (d, *J* = 1 Hz, CH), 136.6 (C), 133.9 (d, *J* = 9 Hz, CH), 133.6 (CH), 131.0 (d, *J* = 3 Hz, CH), 129.9 (CH), 129.0 (CH), 127.2 (d, *J* = 7 Hz, C), 126.9 (d, *J* = 13 Hz, C), 126.4 (CH), 124.4 (d, *J* = 4 Hz, CH), 123.4 (CH), 116.4 (d, *J* = 23 Hz, CH), 83.5 (CH), 81.0 (C). **IR (neat)** v/cm^{-1}: 3253 (w), 1732 (w), 1584 (w), 1452 (w), 1369 (m), 1202

(m), 1057 (m), 872 (w), 709 (s), 692 (s), 547 (m). **^{19}F-NMR (376 MHz, $CDCl_3$) δ/ppm** -107.8 (m). **HR-MS (QTOF ES+):** calcd for $C_{17}H_{12}FO$ 251.0867 found 251.0868 ($M+H^+$).

(*E*)-3-(2-Ethynylphenyl)-1-(o-tolyl)prop-2-en-1-one (4l)

Synthesised via Procedure B

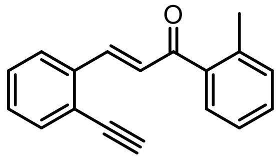

Chemical Formula: $C_{18}H_{14}O$
Exact Mass: 246.1045

Yield: 72% (329 mg, 1.34 mmol)

Appearance: Light-yellow solid

^{1}H-NMR (400 MHz, $CDCl_3$) δ/ppm 7.99 (d, *J* = 17.3 Hz, 1H), 7.75 – 7.68 (m, 1H), 7.60 – 7.51 (m, 2H), 7.43 – 7.33 (m, 3H), 7.31 – 7.26 (m, 2H), 7.20 (d, *J* = 16.1 Hz, 1H), 3.33 (s, 1H), 2.47 (s, 3H). **^{13}C-NMR (100 MHz, $CDCl_3$)** δ/ppm 196.4 (C), 143.4 (CH), 138.8 (C), 137.1 (C), 136.7 (C), 133.5 (CH), 131.3 (CH), 130.5 (CH), 129.9 (CH), 129.1 (CH), 128.3 (CH), 128.3 (CH), 126.2 (CH), 125.4 (CH), 123.2 (C), 83.4 (CH), 80.9 (C), 20.3 (CH_3). **IR (neat)** v/cm^{-1}: 3283 (w), 1642 (m), 1601 (m), 1454 (w), 1269 (m), 1131 (w), 1013 (m), 883 (w), 756 (s), 470 (w). **HR-MS (QTOF ES+):** calcd for $C_{18}H_{15}O$ 247.1117 found 247.1119 ($M+H^+$).

(*E*)-3-(2-Ethynylphenyl)-1-(2-fluoro-4-methoxyphenyl)prop-2-en-1-one (4m)

Synthesised via Procedure B

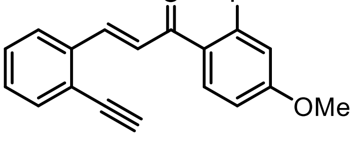

Chemical Formula: $C_{18}H_{13}FO_2$
Exact Mass: 280.0900

Yield: 70% (291 mg, 1.04 mmol)

Appearance: Light-yellow solid

^{1}H-NMR (400 MHz, $CDCl_3$) δ/ppm 8.29 – 8.21 (m, 1H), 7.90 (t, *J* = 8.7 Hz, 1H), 7.78 – 7.70 (m, 1H), 7.59 – 7.52 (m, 2H), 7.41 – 7.36 (m, 1H), 7.34 (td, *J* = 7.5, 1.5 Hz, 1H), 6.79 (ddd, *J* = 8.8, 2.5, 0.5 Hz, 1H), 6.65 (dd, *J* = 13.0, 2.4 Hz, 1H), 3.87 (s, 3H), 3.43 (s, 1H). **^{13}C-NMR (100 MHz, $CDCl_3$)** δ/ppm 186.9 (d, *J* = 4 Hz, C), 164.5 (d, *J* = 11 Hz, C), 163.08 (d, *J* = 250 Hz, C), 141.2 (d, *J* = 1 Hz, CH), 136.9 (C), 133.5 (CH), 132.70 (d, *J* = 5 Hz, CH), 129.7 (CH), 129.0 (CH), 127.2 (d, *J* = 8 Hz, CH), 126.4 (CH), 123.3 (C), 119.5 (d, *J* = 13 Hz, C), 110.7 (d, *J* = 3 Hz, CH), 101.7 (d, *J* = 28 Hz,

CH), 83.45 (CH), 81.1 (C), 55.8 (CH_3). **^{19}F-NMR (376 MHz, $CDCl_3$) δ/ppm** -104.6 (ddd, *J* = 10.8, 7.9, 6.8 Hz). **IR (neat)** ν/cm^{-1}: 3215 (w), 1651 (m), 1586 (s), 1410 (w), 1270 (m), 1156 (w), 1024 (w), 957 (w), 753 (s), 590 (w). **HR-MS (QTOF ES+):** calcd for $C_{18}H_{14}FO_2$ 281.0972 found 281.0975 (M+H$^+$).

(*E*)-3-(2-Ethynyl-4-methylphenyl)-1-(4-fluorophenyl)prop-2-en-1-one (4n)

Synthesised via Procedure B

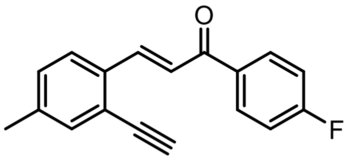

Chemical Formula: $C_{18}H_{13}FO$
Exact Mass: 264.0950

Yield: 50% (122 mg, 0.46 mmol)

Appearance: Light-yellow solid

^{1}H-NMR (400 MHz, $CDCl_3$) δ/ppm 8.23 (d, *J* = 15.8 Hz, 1H), 8.15 – 7.99 (m, 2H), 7.65 (d, *J* = 8.1 Hz, 1H), 7.55 (d, *J* = 15.8 Hz, 1H), 7.40 (s, 1H), 7.23 – 7.14 (m, 3H), 3.41 (s, 1H), 2.37 (s, 3H). **^{13}C-NMR (100 MHz, $CDCl_3$)** δ/ppm 189.1 (C), 165.5 (d, *J* = 250 Hz, C), 142.6 (CH), 140.4 (C), 134.5 (d, *J* = 3 Hz, C), 134.2 (CH), 133.9 (C), 131.0 (d, *J* = 9 Hz, 2CH), 130.1 (CH), 126.4 (CH), 123.2 (C), 122.7 (CH), 115.63 (d, *J* = 22 Hz, 2CH), 83.1 (CH), 81.3 (C), 21.1 (CH_3). **^{19}F-NMR (376 MHz, $CDCl_3$) δ/ppm** -105.7 (tt, *J* = 8.4, 5.4 Hz). **IR (neat)** ν/cm^{-1}: 3226 (m), 1653 (m), 1586 (s), 1446 (w), 1268 (s), 1031 (s), 985 (s), 841 (w), 779 (w), 680 (s). **HR-MS (QTOF ES+):** calcd for $C_{18}H_{14}FO$ 265.1023 found 265.1023 (M+H$^+$).

(*E*)-3-(2-Ethynyl-4-fluorophenyl)-1-(p-tolyl)prop-2-en-1-one (4o)

Synthesised via Procedure B

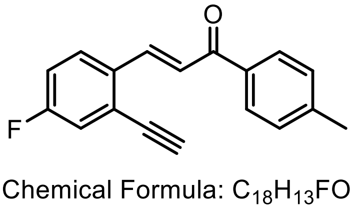

Chemical Formula: $C_{18}H_{13}FO$
Exact Mass: 264.0950

Yield: 49% (156 mg, 0.59 mmol)

Appearance: Light-yellow solid

^{1}H-NMR (400 MHz, $CDCl_3$) δ/ppm 8.20 (d, *J* = 15.8 Hz, 1H), 7.93 (d, *J* = 8.2 Hz, 2H), 7.74 (dd, *J* = 8.8, 5.6 Hz, 1H), 7.55 (dd, *J* = 15.8, 0.6 Hz, 1H), 7.30 (dt, *J* = 8.0, 0.7 Hz, 2H), 7.28 – 7.21 (m, 1H), 7.17 – 7.07 (m, 1H), 3.48 (s, 1H), 2.44 (s, 3H). **^{13}C-NMR (100 MHz, $CDCl_3$)** δ/ppm 193.7 (C), 161.8 (d, *J* = 250 Hz, C), 144.2 (C), 136.3 (CH), 134.5 (C), 134.2 (d, *J* = 4 Hz, C), 131.1 (d, *J* = 9 Hz, C), 129.1 (2CH), 129.0 (2CH), 127.9 (d, *J* = 2 Hz, C), 123.5 (d, *J* = 10 Hz, C), 119.2 (d, *J* = 23 Hz,

CH), 116.0 (d, *J* = 22 Hz, CH), 83.5 (CH), 80.7 (d, *J* = 3 Hz, C), 21.6 (CH_3). **^{19}F-NMR (376 MHz, $CDCl_3$) δ/ppm** -112.4 (td, *J* = 8.5, 5.6 Hz). **IR (neat)** ν/cm^{-1}: 3235 (m), 2980 (m), 1658 (m), 1589 (s), 1333 (w), 1255 (s), 943 (w), 810 (m), 711 (w). **HR-MS (QTOF ES+):** calcd for $C_{18}H_{14}FO$ 265.1023 found 265.1025 ($M+H^+$).

(*E*)-3-(2-Ethynylphenyl)-1-phenylprop-2-en-1-one (4p)

Synthesised via Procedure B

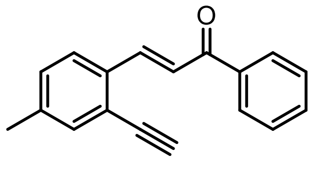

Chemical Formula: $C_{18}H_{14}O$
Exact Mass: 246.1045

Yield: 56% (127 mg, 0.52 mmol)

Appearance: Light-yellow solid

^{1}H-NMR (400 MHz, $CDCl_3$) δ/ppm 8.24 (d, *J* = 15.8 Hz, 1H), 8.04 – 7.99 (m, 2H), 7.66 (d, *J* = 8.1 Hz, 1H), 7.62 – 7.55 (m, 2H), 7.53 – 7.47 (m, 2H), 7.39 (s, 1H), 7.21 (d, *J* = 9.3 Hz, 1H), 3.41 (s, 1H), 2.36 (s, 3H). **^{13}C-NMR (100 MHz, $CDCl_3$)** δ/ppm 190.7 (C), 142.3 (CH), 140.3 (C), 138.2 (C), 134.1 (CH), 134.1 (C), 132.6 (CH), 130.0 (CH), 128.5 (4CH), 128.2 (C), 126.4 (CH), 123.1 (CH), 83.1 (CH), 81.3 (C), 21.1 (CH_3). **IR (neat)** ν/cm^{-1}: 3228 (m), 1637 (m), 1593 (s), 1488 (m), 1406 (m), 1270 (m), 1073 (m), 987 (m), 818 (s), 607 (w). **HR-MS (QTOF ES+):** calcd for $C_{18}H_{15}O$ 247.1117 found 247.1117 ($M+H^+$).

(*E*)-3-(2-Ethynyl-4-fluorophenyl)-1-(4-fluorophenyl)prop-2-en-1-one (4q)

Synthesised via Procedure B

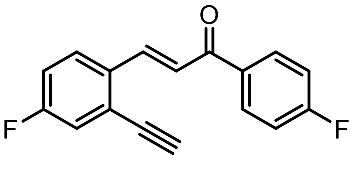

Chemical Formula: $C_{17}H_{10}F_2O$
Exact Mass: 268.0700

Yield: 65% (209 mg, 0.78 mmol)

Appearance: Light-yellow solid

^{1}H-NMR (400 MHz, $CDCl_3$) δ/ppm 8.20 (d, *J* = 15.8 Hz, 1H), 8.05 (dd, *J* = 8.8, 5.4 Hz, 2H), 7.74 (dd, *J* = 8.8, 5.6 Hz, 1H), 7.52 (d, *J* = 15.8 Hz, 1H), 7.28 – 7.25 (m, 1H), 7.23 – 7.08 (m, 3H), 3.49 (s, 1H). **^{13}C-NMR (100 MHz, $CDCl_3$)** δ/ppm 188.8 (C), 165.6 (d, *J* = 250 Hz, C), 163.2 (d, *J* = 250 Hz, C), 141.3 (CH) 134.3 (d, *J* = 3 Hz, C), 133.1 (d, *J* = 4 Hz, C), 131.1 (d, *J* = 9 Hz, 2CH), 128.4 (d, *J* = 9 Hz, CH), 125.2 (d, *J* = 10 Hz, C), 123.4 (d, *J* = 2 Hz, CH), 120.2 (d, *J* = 23 Hz, CH),

116.9 (d, *J* = 22.0 Hz, CH), 115.7 (d, *J* = 22 Hz, 2CH), 84.6 (CH), 80.0 (C). **^{19}F-NMR (376 MHz, $CDCl_3$) δ/ppm** -105.3 (tt, *J* = 8.4, 5.4 Hz), -109.6 (td, *J* = 8.3, 5.6 Hz). **IR (neat)** v/cm^{-1}: 3288 (m), 1644 (m), 1593 (s), 1405 (w), 1259 (s), 1072 (m), 843 (m), 696 (s), 513 (s). **HR-MS (QTOF ES+):** calcd for $C_{17}H_{11}F_2O$ 269.0772 found 269.0774 (M+H$^+$).

(*E*)-3-(2-Ethynyl-4-methylphenyl)-1-(p-tolyl)prop-2-en-1-one (4r)

Synthesised via Procedure B

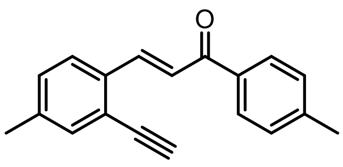

Chemical Formula: $C_{19}H_{16}O$
Exact Mass: 260.1201

Yield: 68% (96 mg, 0.37 mmol)

Appearance: Light-yellow solid

^{1}H-NMR (400 MHz, $CDCl_3$) δ/ppm 8.23 (d, *J* = 15.8 Hz, 1H), 7.95 – 7.91 (m, 2H), 7.65 (d, *J* = 8.1 Hz, 1H), 7.59 (d, *J* = 15.8 Hz, 1H), 7.39 (s, 1H), 7.29 (dt, *J* = 8.0, 0.7 Hz, 2H), 7.20 (ddd, *J* = 8.1, 1.8, 0.9 Hz, 1H), 3.41 (s, 1H), 2.43 (s, 3H), 2.36 (s, 3H). **^{13}C-NMR (100 MHz, $CDCl_3$)** δ/ppm 190.1 (C), 143.5 (C), 141.8 (CH), 140.1 (C), 135.5 (C), 134.2 (C), 134.0 (CH), 130.0 (CH), 129.2 (2CH), 128.6 (2CH), 126.3 (CH), 123.1 (CH), 123.1 (C) 83.0 (CH), 81.4 (C), 21.6 (CH_3), 21.0 (CH_3). **IR (neat)** v/cm^{-1}: 3222 (s), 1660 (m), 1594 (s), 1475 (m), 1380 (w), 1254 (s), 1180 (m), 943 (w), 814 (w). **HR-MS (QTOF ES+):** calcd for $C_{19}H_{17}O$ 261.1274 found 261.1276 (M+H$^+$).

(*E*)-3-(2-Ethynyl-4-fluorophenyl)-1-phenylprop-2-en-1-one (4s)

Synthesised via Procedure B

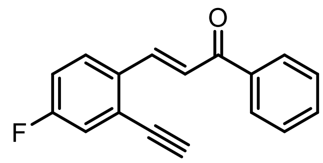

Chemical Formula: $C_{17}H_{11}FO$
Exact Mass: 250.0794

Yield: 65% (195 mg, 0.78 mmol)

Appearance: Light-yellow solid

^{1}H-NMR (400 MHz, $CDCl_3$) δ/ppm 8.21 (d, *J* = 15.8 Hz, 1H), 8.04 – 7.99 (m, 2H), 7.74 (dd, *J* = 8.8, 5.6 Hz, 1H), 7.61 – 7.56 (m, 2H), 7.53 – 7.48 (m, 2H), 7.25 (dd, *J* = 8.8, 2.7 Hz, 1H), 7.11 (td, *J* = 8.3, 2.7 Hz, 1H), 3.48 (s, 1H). **^{13}C-NMR (100 MHz, $CDCl_3$)** δ/ppm 190.3 (C), 162.9 (d, *J* = 250 Hz, C), 141.1 (CH), 137.9 (C), 133.3 (d, *J* = 3 Hz, C), 132.8 (CH), 128.6 (2CH), 128.5 (2CH), 128.4 (d, *J* = 9 Hz, C) 125.1 (d, *J* = 10 Hz, CH), 123.8 (d, *J* = 2 Hz, CH),

120.1 (d, J = 23 Hz, CH), 116.8 (d, J = 22 Hz, CH), 84.5 (CH), 80.0 (d, J = 3 Hz, C). **^{19}F-NMR (376 MHz, $CDCl_3$) δ/ppm**-109.6 (td, J = 8.4, 5.6 Hz). **IR (neat)** ν/cm^{-1}: 3220 (m), 1659 (s), 1591 (s), 1449 (m), 1332 (w), 1283 (s), 1212 (s), 1015 (s), 869 (m), 744 (s), 597 (w). **HR-MS (QTOF ES+):** calcd for $C_{17}H_{12}FO$ 251.0867 found 251.0867 (M+H$^+$).

Characterisation of 11*H*-Benzo[*b*]fluorenes

9-Methoxy-11*H*-benzo[*b*]fluoren-10-ol (5a)

Synthesised via Procedure C

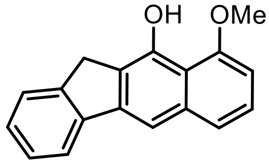

Chemical Formula: $C_{18}H_{14}O_2$
Exact Mass: 262.0994

Yield: 26% (27 mg, 0.10 mmol)

Appearance: Light-brown solid

Melting point: 176-178 °C

Off-brown crystals suitable for X-ray crystallography were obtained by slow evaporation of a DCM solution.

^{1}H-NMR (400 MHz, $CDCl_3$) δ/ppm 9.57 (s, 1H), 7.89 (m, 1H), 7.74 (s, 1H), 7.64 – 7.59 (m, 1H), 7.52 (dt, J = 8.4, 1 Hz, 1H), 7.45 – 7.33 (m, 2H), 7.30 (t, J = 8.0 Hz, 1H), 6.77 (dd, J = 7.7, 1.0 Hz, 1H), 4.08 (s, 3H), 4.01 (s, 2H). **^{13}C-NMR (100 MHz, $CDCl_3$)** δ/ppm 156.4 (C), 149.9 (C), 144.3 (C), 142.4 (C), 141.1 (C), 136.9 (C), 127.5 (CH), 126.6 (CH), 125.3 (CH), 125.0 (CH), 123.6 (C), 122.2 (CH), 120.7 (CH), 114.1 (C), 109.4 (CH), 103.4 (CH), 56.0 (CH_3), 33.7 (CH_2). **IR (neat)** ν/cm^{-1}: 3272 (m), 2920 (w), 1645 (w), 1614 (w), 1217 (m), 1066 (m), 1021 (s), 827 (w), 745 (m). **HR-MS (QTOF ES+):** calcd for $C_{18}H_{15}O_2$ 263.1067 found 263.1066 (M+H$^+$).

11*H*-Benzo[*b*]fluoren-10-ol (5b)

Synthesised via Procedure C

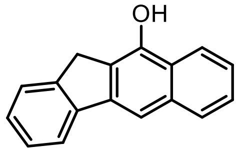

Chemical Formula: $C_{17}H_{12}O$
Exact Mass: 232.0888

Yield: 41% (942 mg, 4.05 mmol)

Appearance: Light-brown solid

Melting point: 109-110 °C

^{1}H-NMR (400 MHz, DMSO) δ/ppm 9.79 (s, 1H), 8.26 (td, *J* = 7.9, 1.0 Hz, 1H), 7.98 (m, 1H), 7.92 (m, 2H), 7.63 (td, *J* = 7.3, 1 Hz, 1H), 7.49-7.38 (m, 3H), 7.36 (td, *J* = 7.3, 1.4 Hz, 1H), 4.02 (s, 2H). **^{13}C-NMR (100 MHz, DMSO)** δ/ppm 148.9 (C), 144.0 (C), 141.6 (C), 141.6 (C), 135.0 (C), 128.4 (CH), 128.2 (CH), 127.5 (CH), 126.2 (CH), 126.1 (CH), 125.2 (C), 124.8 (CH), 122.7 (CH), 122.5 (C), 121.3 (CH), 101.3 (CH), 34.5 (CH_2). **IR (neat)** ν/cm^{-1}: 3428 (w), 1645 (w), 1585 (m), 1504 (m), 1417 (m), 1256 (m), 942 (w), 843 (w), 763 (s), 723 (s). **HR-MS (QTOF ES+):** calcd for $C_{17}H_{13}O$ 233.0961 found 233.0963 ($M+H^+$).

7-(Trifluoromethyl)-11*H*-benzo[*b*]fluoren-10-ol (5c)

Synthesised via Procedure C

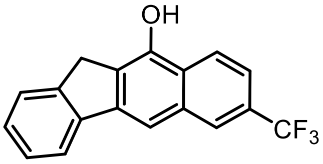

Chemical Formula: $C_{18}H_{11}F_3O$
Exact Mass: 300.0762

Yield: 42% (52 mg, 0.17 mmol)

Appearance: Light-brown solid

Melting point: 134-135 °C

^{1}H-NMR (400 MHz, $CDCl_3$) δ/ppm 8.29 (d, *J* = 8.8 Hz, 1H), 8.17 (s, 1H), 7.93 – 7.87 (m, 1H), 7.87 (s, 1H), 7.61 (dd, *J* = 8.8, 1.8 Hz, 1H), 7.57 (dt, *J* = 7.3, 1 Hz, 1H), 7.44 (td, *J* = 7.4, 1.3 Hz, 1H), 7.39 (td, *J* = 7.4, 1.4 Hz, 1H), 5.24 (s, 1H), 3.93 (s, 2H). **^{13}C-NMR (100 MHz, $CDCl_3$)** δ/ppm 146.9 (C), 142.7 (C) 142.6 (C), 140.7 (C), 133.5 (C), 128.1 (CH), 127.3 (CH), 125.8 (C), 125.6 (q, *J* = 4.5 Hz, C), 125.4 (CH), 124.9 (CH), 124.0 (CH), 123.1 (C), 122.4 (CH), 120.9 (CH), 120.1 (q, *J* = 3 Hz, C), 111.9 (CH), 32.8 (CH_2). **IR (neat)** ν/cm^{-1}: 3274 (w), 1632 (m), 1562 (m), 1416 (m), 1379 (m), 1259 (s), 1032 (m), 945 (m), 863 (w), 759 (s), 595 (w). **^{19}F NMR (376 MHz, $CDCl_3$)** δ/ppm -62.1 (m). **HR-MS (QTOF ES+):** calcd for $C_{18}H_{12}F_3O$ 301.0835 found 301.0834 ($M+H^+$).

7-Fluoro-11*H*-benzo[*b*]fluoren-10-ol (5d)

Synthesised via Procedure C

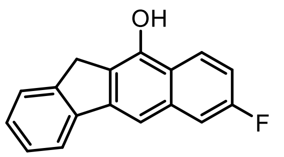

Chemical Formula: $C_{17}H_{11}FO$
Exact Mass: 250.0794

Yield: 37% (74 mg, 0.30 mmol)

Appearance: Light-brown solid

Melting point: 160-161 °C

^{1}H-NMR (500 MHz, DMSO) δ/ppm 9.97 (s, 1H), 8.28 (m, 1H), 7.95 (m, 1H), 7.90 (s, 1H), 7.64 (m, 2H), 7.42 (td, *J* = 7.7, 1.4 Hz, 1H), 7.38 (td, *J* = 7.4, 1.4 Hz, 1H), 7.31 (td, *J* = 8.8, 2.4 Hz, 1H), 4.01 (s, 2H). **^{13}C-NMR (125 MHz, DMSO)** δ/ppm 160.7 (d, *J* = 250 Hz, CF), 149.1 (C), 144.1 (C), 143.0 (C), 141.1 (C), 135.8 (d, *J* = 10 Hz, C), 128.4 (CH), 127.4 (CH), 126.0 (CH), 125.4 (d, *J* = 10 Hz, CH), 122.2 (d, *J* = 2 Hz, C), 122.0 (d, J = 2 Hz, C), 121.3 (CH), 114.4 (d, *J* = 20 Hz, CH), 111.0 (d, *J* = 20 Hz, CH), 109.6 (d, *J* = 6 Hz, CH), 34.4 (CH_2). **^{19}F-NMR (376 MHz, DMSO) δ/ppm** -116.1 (m). **IR (neat)** v/cm^{-1}: 3222 (s), 1633 (m), 1584 (m), 1415 (m), 1240 (s), 1175 (s), 1073 (s), 944 (s), 813 (w), 760 (s). **HR-MS (QTOF ES+):** calcd for $C_{17}H_{12}FO$ 251.0867 found 251.0866 ($M+H^+$).

10-Hydroxy-11*H*-benzo[*b*]fluorene-7-carbonitrile (5e)

Synthesised via Procedure C

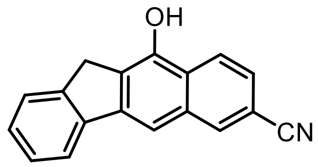

Chemical Formula: $C_{18}H_{11}NO$
Exact Mass: 257.0841

Yield: 53% (49 mg, 0.19 mmol)

Appearance: Light-brown solid

Melting point: 160-161 °C

^{1}H-NMR (500 MHz, DMSO) δ/ppm 10.25 (s, 1H), 8.51 (d, *J* = 2 Hz, 1H), 8.36 (d, *J* = 8.7 Hz, 1H), 8.05 (d, *J* = 2.2 Hz, 1H), 8.00 (d, *J* = 7.3 Hz, 1H), 7.71 – 7.61 (m, 2H), 7.51 – 7.37 (m, 2H), 4.07 (s, 2H). **^{13}C-NMR (125 MHz, DMSO)** δ/ppm 148.5 (C), 143.5 (C), 142.8 (C), 140.1 (C), 134.0 (CH), 133.2 (C), 128.3 (CH), 127.1 (CH), 125.7 (CH), 125.6 (CH), 125.6 (C), 124.5 (CH), 123.6 (C), 121.0 (CH), 119.5 (C), 110.2 (CH), 108.1 (C), 34.2 (CH_2). **IR (neat)** v/cm^{-1}: 3321 (w), 1640 (w), 1502 (w), 1416 (m), 1277 (m), 1139 (m), 944 (s), 881 (s), 760 (s). **HR-MS (QTOF ES+):** calcd for $C_{18}H_{12}NO$ 258.0913 found 258.0913 ($M+H^+$).

7-Methoxy-11*H*-benzo[*b*]fluoren-10-ol (5f)

Synthesised via Procedure C

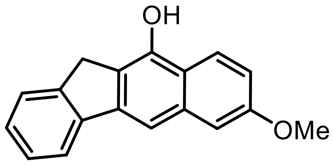

Chemical Formula: $C_{18}H_{14}O_2$
Exact Mass: 262.0994

Yield: 40% (56 mg, 0.21 mmol)

Appearance: Light-brown solid

Melting point: 200-202 °C

^{1}H-NMR (400 MHz, DMSO) δ/ppm 9.71 (s, 1H), 8.14 (d, *J* = 9.2 Hz, 1H), 7.94 (m, 1H), 7.82 (s, 1H), 7.61 (dt, *J* = 7.2, 1 Hz, 1H), 7.41 (td, *J* = 7.4, 1 Hz, 1H), 7.35 (td, *J* = 7.3, 1 Hz, 1H), 7.31 (d, *J* = 3 Hz, 1H), 7.08 (dd, *J* = 9.1, 3 Hz, 1H), 3.97 (s, 2H), 3.88 (3H). **^{13}C-NMR (100 MHz, DMSO)** δ/ppm 157.2 (C), 148.5 (C), 143.5 (C), 141.6 (C), 141.0 (C), 135.8 (C), 127.4 (CH), 126.8 (CH), 125.4 (CH), 123.5 (CH), 120.5 (CH), 120.0 (C), 119.9 (C), 116.3 (CH), 108.8 (C), 106.2 (C), 55.0 (CH_3), 33.8 (CH_2). **IR (neat)** v/cm^{-1}: 3312 (w), 1633 (w), 1503 (w), 1416 (m), 1239 (s), 1172 (s), 1081 (s), 944 (s), 759 (s), 595 (w). **HR-MS (QTOF ES+):** calcd for $C_{18}H_{15}O_2$ 263.1067 found 263.1067 (M+H$^+$).

7-Methyl-11*H*-benzo[*b*]fluoren-10-ol (5g)

Synthesised via Procedure C

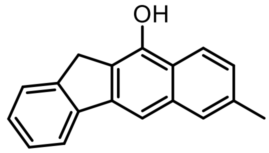

Chemical Formula: $C_{18}H_{14}O$
Exact Mass: 246.1045

Yield: 46% (40 mg, 0.16 mmol)

Appearance: Light-brown solid

Melting point: 170-171 °C

^{1}H-NMR (500 MHz, DMSO) δ/ppm 9.70 (s, 1H), 8.14 (d, *J* = 8.5 Hz, 1H), 7.96 (d, *J* = 7.4 Hz, 1H), 7.81 (s, 1H), 7.67 (s, 1H), 7.62 (d, *J* = 7.4 Hz, 1H), 7.41 (t, *J* = 7.3 Hz, 1H), 7.35 (t, *J* = 7.4 Hz, 1H), 7.27 (dd, *J* = 8.4, 2 Hz, 1H), 3.99 (s, 2H), 2.47 (s, 3H). **^{13}C-NMR (125 MHz, DMSO)** δ/ppm 148.8 (C), 143.9 (C), 141.5 (C), 135.1 (C), 135.1 (C), 127.9 (CH), 127.3 (CH), 127.2 (C), 127.2 (CH), 126.8 (CH), 125.9 (CH), 123.4 (C), 122.4 (CH), 121.7 (C), 121.1 (CH), 109.6 (CH), 34.3 (CH_2), 21.7 (CH_3). **IR (neat)** v/cm^{-1}: 3385 (w), 1642 (w), 1578 (w), 1475 (m), 1210 (m), 1075 (s), 944 (w), 882 (w). **HR-MS (QTOF ES+):** calcd for $C_{18}H_{15}O$ 247.1117 found 247.1117 (M+H$^+$).

7,8-Dimethyl-11*H*-benzo[*b*]fluoren-10-ol / 6,7-dimethyl-11H-benzo[b]fluoren-10-ol (5h)

Note - mixture of 3,4- and 4,5-isomers.

Synthesised via Procedure C

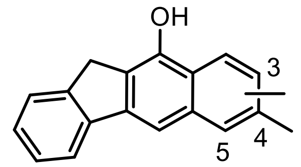

Chemical Formula: $C_{19}H_{16}O$
Exact Mass: 260.1201

Yield: 31% (40 mg, 0.15 mmol)

Appearance: Light-brown solid

Melting point: 110 °C

^{1}H-NMR (500 MHz, $CDCl_3$) δ/ppm 8.04 (s, 1H), 7.98 – 7.93 (m, 2H), 7.90 – 7.86 (m, 2H), 7.75 (s, 1H), 7.65 (s, 1H), 7.59 – 7.53 (m, 2H), 7.41 (dtt, *J* = 10, 7.5, 1 Hz, 2H), 7.35 (ddd, *J* = 8.8, 7.4, 1 Hz, 2H), 7.30 (d, *J* = 8.5 Hz, 1H), 5.19 (s, 1H), 5.15 (s, 1H), 3.94 (s, 2H), 3.94 (s, 2H), 2.68 (s, 3H), 2.51 (s, 3H), 2.47 (s, 3H), 2.45 (s, 3H). **^{13}C-NMR (125 MHz, $CDCl_3$)** δ/ppm 147.1 (C), 146.2 (C), 142.9 (C), 142.8 (C), 141.8 (C), 141.6 (C), 140.9 (C), 140.3 (C), 135.4 (C), 134.4 (C), 134.2 (C), 133.6 (C), 133.0 (C), 131.4 (C), 127.8 (CH), 127.7 (CH), 127.3 (CH), 127.2 (CH), 126.9 (CH), 126.9 (CH), 125.2 (CH), 125.2 (CH), 122.5 (C), 122.4 (C), 121.0 (C), 120.5 (CH), 120.5 (CH), 120.2 (CH), 118.2 (CH), 110.3 (CH), 107.2 (CH), 32.7 (CH_2), 32.7 (CH_2), 20.8 (CH_3), 20.3 (CH_3), 20.0 (CH_3), 15.1 (CH_3), one carbon not observed. **IR (neat)** ν/cm^{-1} 3276 (w), 1632 (w), 1563 (w), 1462 (w), 1379 (m), 1240 (m), 1083 (m), 864 (m), 759 (s), 715 (m). **HR-MS (QTOF ES+):** calcd for $C_{19}H_{17}O$ 261.1274 found 261.1273 ($M+H^+$).

6,8-Difluoro-11*H*-benzo[*b*]fluoren-10-ol (5i)

Synthesised via Procedure C

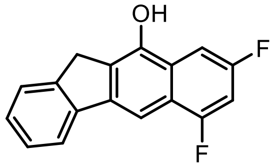

Chemical Formula: $C_{17}H_{10}F_2O$
Exact Mass: 268.0700

Yield: 44% (43 mg, 0.16 mmol)

Appearance: Light-brown solid

Melting point: 174-175 °C

^{1}H-NMR (400 MHz, DMSO) δ/ppm 10.18 (s, 1H), 8.08 (m, 1H), 8.03 (s, 1H), 7.74 (dd, *J* = 10.5, 3 Hz, 1H), 7.63 (m, 1H), 7.43-7.36 (m, 3H), 4.03 (s, 2H). **^{13}C-NMR (100 MHz, DMSO)** δ/ppm 159.5 (dd, *J* = 250, 14 Hz, CF), 158.7 (dd, *J* = 250, 14 Hz, CF), 148.8 (m, C), 143.9 (C), 141.9 (C), 141.0 (C), 128.6 (CH), 127.6 (CH), 126.1 (CH), 126.2 (d, *J* = 7.6 Hz, C), 125.2 (C), 122.1 (d, *J*

= 17 Hz, C), 121.7 (CH), 102.6 (m, CH), 102.5 (m, CH), 101.8 (dd, *J* = 29, 24 Hz, CH), 34.6 (CH_2). **IR (neat)** ν/cm^{-1}: 3278 (w), 1628 (m), 1585 (m), 1431 (s), 1271 (s), 1145 (m), 1090 (m), 991 (w), 761 (s), 697 (s), 594 (w). **^{19}F-NMR (376 MHz, DMSO)** δ/ppm -113.9 (m), -117.4 (m). **HR-MS (QTOF ES+):** calcd for $C_{17}H_{11}F_2O$ 269.0772 found 269.0772 ($M+H^+$).

6-Fluoro-11*H*-benzo[*b*]fluoren-10-ol (5j)

Synthesised via Procedure C

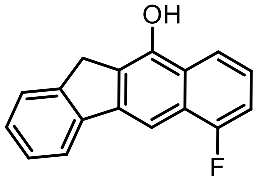

Chemical Formula: $C_{17}H_{11}FO$
Exact Mass: 250.0794

Yield: 8% (10 mg, 0.04 mmol)

Note - Purification yield

Appearance: Light-brown solid

Melting point: 120-121 °C

^{1}H-NMR (500 MHz, $CDCl_3$) δ/ppm 7.90 – 7.85 (m, 2H), 7.84 – 7.80 (m, 2H), 7.57 (dt, *J* = 7.4, 1 Hz, 1H), 7.42 (td, *J* = 7.5, 1 Hz, 1H), 7.36 (td, *J* = 7.4, 1 Hz, 1H), 7.30 – 7.22 (m, 1H), 5.19 (s, 1H), 3.96 (s, 2H). **^{13}C-NMR (125 MHz, $CDCl_3$)** δ/ppm 160.2 (d, *J* = 250 Hz, C), 146.5 (d, *J* = 6 Hz, C), 142.5 (C), 141.1 (C), 140.6 (d, *J* = 2 Hz, C), 131.7 (C), 130.2 (d, *J* = 8.7 Hz, CH), 127.7 (CH), 127.1 (CH), 125.3 (CH), 124.5 (d, *J* = 6 Hz, C), 122.7 (C), 120.7 (CH), 116.1 (d, *J* = 25 Hz, CH), 111.1 (CH), 105.2 (d, *J* = 23 Hz, CH), 32.8 (CH_2). **^{19}F NMR (376 MHz, $CDCl_3$)** δ/ppm -115.3 (ddd, *J* = 10.6, 8.2, 5.5 Hz). **IR (neat)** ν/cm^{-1}: 3221 (m), 1631 (m), 1502 (w), 1416 (w), 1238 (m), 1160 (s), 1082 (s), 856 (m), 756 (s). **HR-MS (QTOF ES+):** calcd for $C_{17}H_{12}FO$ 251.0867 found 251.0866 ($M+H^+$).

8-Fluoro-11*H*-benzo[*b*]fluoren-10-ol (5j')

Synthesised via Procedure C

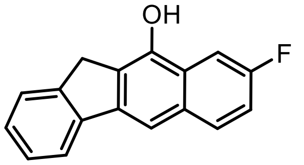

Chemical Formula: $C_{17}H_{11}FO$
Exact Mass: 250.0794

Yield: 8% (10 mg, 0.04 mmol)

Note - Purification yield

Appearance: Light-brown solid

Melting point: 120-121 °C

^{1}H-NMR (500 MHz, $CDCl_3$) δ/ppm 8.09 (s, 1H), 7.95 (ddt, *J* = 7.7, 5.6, 1 Hz, 2H), 7.59 (d, *J* = 1 Hz, 1H), 7.44 (tt, *J* = 7.5, 1 Hz, 1H), 7.40 – 7.35 (m, 2H), 7.17 (ddd, *J* = 11, 7.6, 1 Hz, 1H), 5.33 (s, 1H), 3.98 (s,

2H). **^{13}C-NMR (125 MHz, $CDCl_3$)** δ/ppm 159.2 (d, *J* = 250 Hz, C), 146.9 (d, *J* = 3 Hz, C), 142.8 (C), 141.6 (d, *J* = 2 Hz, C), 141.1 (C), 127.9 (CH), 127.2 (CH), 125.4 (d, *J* = 6 Hz, C), 125.3 (CH), 125.0 (d, *J* = 18 Hz, C), 124.2 (d, *J* = 9 Hz, CH), 122.8 (d, *J* = 1 Hz, C), 121.0 (CH), 116.86 (d, *J* = 4 Hz, CH), 109.5 (d, *J* = 20 Hz, CH), 103.9 (d, *J* = 6 Hz, CH), 32.9 (CH_2). **IR (neat)** ν/cm^{-1}: 3221 (m), 1631 (m), 1502 (w), 1416 (w), 1238 (m), 1160 (s), 1082 (s), 856 (m), 756 (s). **HR-MS (QTOF ES+):** calcd for $C_{17}H_{12}FO$ 251.0867 found 251.0866 ($M+H^+$).

9-Fluoro-11*H*-benzo[*b*]fluoren-10-ol (5k)

Synthesised via Procedure C

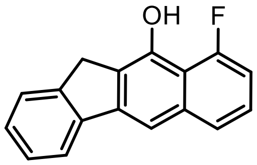

Chemical Formula: $C_{17}H_{11}FO$
Exact Mass: 250.0794

Yield: 34% (60 mg, 0.24 mmol)

Appearance: Light-yellow oil

^{1}H-NMR (400 MHz, DMSO) δ/ppm 9.53 (s, 1H), 8.05 – 7.92 (m, 2H), 7.72 (dd, *J* = 8.5, 1.1 Hz, 1H), 7.68 – 7.58 (m, 1H), 7.49 – 7.33 (m, 3H), 7.12 (ddd, *J* = 13.6, 7.6, 1 Hz, 1H), 4.01 (s, 2H). **^{13}C-NMR (100 MHz, DMSO)** δ/ppm 158.9 (d, *J* = 250 Hz, C), 147.9 (d, *J* = 3 Hz, C), 143.5 (C), 141.9 (d, *J* = 2 Hz, C), 140.4 (C), 137.3 (d, *J* = 4 Hz, C), 128.0 (CH), 127.0 (CH), 125.6 (d, *J* = 9 Hz, CH), 125.5 (CH), 124.2 (d, *J* = 2 Hz, CH), 124.1 (d, *J* = 4 Hz, CH), 120.9 (CH), 114.3 (d, *J* = 10 Hz, C), 109.8 (d, *J* = 3 Hz, C), 109.6 (d, *J* = 22 Hz, CH), 34.0 (CH_2)**. ^{19}F NMR (376 MHz, DMSO)** δ/ppm -113.4 (ddd, *J* = 14, 5, 2 Hz). **IR (neat)** ν/cm^{-1}: 3365 (w), 1670 (m), 1582 (m), 1352 (m), 1258 (m), 1221 (m), 1161 (m), 1030 (m), 850 (w), 761 (s), 723 (s). **HR-MS (QTOF ES+):** calcd for $C_{17}H_{12}FO$ 251.0867 found 251.0867 ($M+H^+$).

9-Methyl-11*H*-benzo[*b*]fluoren-10-ol (5l)

Synthesised via Procedure C

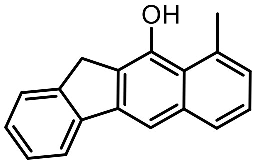

Chemical Formula: $C_{18}H_{14}O$
Exact Mass: 246.1045

Yield: 19% (25 mg, 0.10 mmol)

Appearance: Light-brown solid

Melting point: 145-146 °C

^{1}H-NMR (400 MHz, $CDCl_3$) δ/ppm 7.88 (d, *J* = 7.5 Hz, 1H), 7.81 (s, 1H), 7.76 – 7.71 (m, 1H), 7.57 (dd, *J* = 7.4, 1 Hz, 1H), 7.45 – 7.39 (m, 1H), 7.39 – 7.33 (m, 1H), 7.33 – 7.28

(m, 1H), 7.22 – 7.15 (m, 1H), 5.23 (s, 1H), 3.91 (s, 2H), 3.02 (s, 3H). **^{13}C-NMR (100 MHz, $CDCl_3$)** δ/ppm 149.5 (C), 142.7 (C), 141.3 (C), 140.4 (C), 136.6 (C), 134.2 (C), 127.8 (CH), 127.5 (CH), 127.0 (CH), 126.9 (CH), 125.4 (CH), 125.2 (CH), 123.1 (C), 122.9 (C), 120.8 (CH), 112.0 (CH), 32.9 (CH_2), 24.6 (CH_3). **IR (neat)** ν/cm^{-1}: 3223 (w), 1632 (s), 1583 (w), 1416 (w), 1238 (m), 1160 (m), 1073 (m), 944 (m), 856 (m), 759 (s), 595 (w). **HR-MS (QTOF ES+):** calcd for $C_{18}H_{15}O$ 247.1117 found 247.1116 (M+H$^+$).

9-Fluoro-7-methoxy-11*H*-benzo[*b*]fluoren-10-ol (5m)

Synthesised via Procedure C

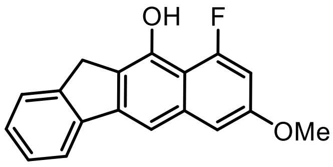

Chemical Formula: $C_{18}H_{13}FO_2$
Exact Mass: 280.0900

Yield: 18% (21 mg, 0.08 mmol)

Appearance: Light-brown solid

Melting point: 146-147 °C

Off-brown crystals suitable for X-ray crystallography were obtained by slow evaporation of the DCM solvent.

^{1}H-NMR (400 MHz, $CDCl_3$) δ/ppm 7.90 – 7.82 (m, 1H), 7.68 (d, *J* = 2.6 Hz, 1H), 7.62 – 7.55 (m, 1H), 7.46 – 7.32 (m, 2H), 7.11 – 6.92 (m, 2H), 6.77 (dd, *J* = 17, 2.4 Hz, 1H), 3.98 (s, 2H), 3.92 (s, 3H). **^{13}C-NMR (100 MHz, $CDCl_3$)** δ/ppm 159.9 (d, *J* = 250 Hz, C), 156.7 (d, *J* = 14 Hz), 146.9 (C), 144.2 (C), 143.5 (d, *J* = 1 Hz, C), 140.7 (C), 137.6 (d, *J* = 5 Hz, C), 127.9 (CH), 126.7 (CH), 125.3 (CH), 122.6 (d, *J* = 2 Hz, C), 120.7 (CH), 109.2 (d, *J* = 4 Hz, CH), 108.3 (d, *J* = 8 Hz, C), 103.2 (d, *J* = 3 Hz, CH), 102.05 (d, *J* = 26 Hz, CH), 55.5 (CH_3), 33.5 (CH_2). **^{19}F NMR (376 MHz, $CDCl_3$)** δ/ppm -120.9 (ddd, *J* = 27, 16, 3 Hz). **IR (neat)** ν/cm^{-1}: 3322 (w), 1632 (m), 1583 (m), 1503 (w), 1415 (m), 1238 (s), 1159 (s), 1082 (s), 944(s), 813 (w), 758 (s). **HR-MS (QTOF ES+):** calcd for $C_{18}H_{14}FO_2$ 281.0972 found 281.0971 (M+H$^+$).

7-Fluoro-3-methyl-11*H*-benzo[*b*]fluoren-10-ol (5n)

Synthesised via Procedure C

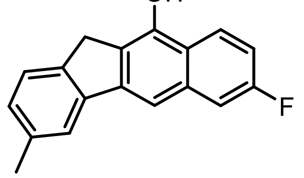

Chemical Formula: $C_{18}H_{13}FO$
Exact Mass: 264.0950

Yield: 28% (31 mg, 0.12 mmol)

Appearance: Light-brown solid

Melting point: 137-139 °C

^{1}H-NMR (400 MHz, acetone-d$_6$) δ/ppm 8.86 (s, 1H), 8.34 (ddt, *J* = 9.2, 6, 1 Hz, 1H), 7.88 (s, 1H), 7.78 (s, 1H), 7.58 (dd, *J* = 11, 2.6 Hz, 1H), 7.47

(dd, J = 7.7, 1 Hz, 1H), 7.25 (ddd, J = 9.2, 8.5, 2.6 Hz, 1H), 7.19 (ddd, J = 7.7, 1.7, 1 Hz, 1H), 4.00 (s, 2H), 2.44 (s, 3H). **^{13}C-NMR (100 MHz, acetone-d$_6$)** δ/ppm 161.8 (d, J = 250 Hz, C), 144.1 (C), 142.2 (C), 141.9 (C), 137.4 (C), 136.9 (d, J = 9 Hz, C), 129.8 (CH), 126.2 (CH), 125.6 (d, J = 10 Hz, CH), 122.9 (C), 122.8 (C), 122.2 (CH), 122.0 (C), 114.8 (d, J = 25 Hz, CH), 111.4 (d, J = 21 Hz, CH), 110.3 (d, J = 5 Hz, C), 34.0 (CH_2), 21.5 (CH_3). **^{19}F-NMR (376 MHz, acetone-d$_6$)** δ/ppm -117.9 (td, J = 10, 6.3 Hz). **IR (neat)** ν/cm^{-1}: 3299 (w), 1621 (w), 1566 (s), 1398 (w), 1197 (s), 1133 (s), 1081 (s), 869 (m), 802 (s), 615 (w). **HR-MS (QTOF ES+):** calcd for $C_{18}H_{14}FO$ 265.1023 found 265.1024 (M+H$^+$).

3-Fluoro-7-methyl-11*H*-benzo[*b*]fluoren-10-ol (5o)

Synthesised via Procedure C

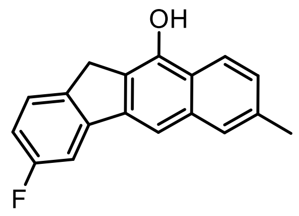

Chemical Formula: $C_{18}H_{13}FO$
Molecular Weight: 264.30

Yield: 33% (37 mg, 0.14 mmol)

Appearance: Light-brown solid

Melting point: 140-142 °C

^{1}H-NMR (500 MHz, acetone-d$_6$) δ/ppm 8.21 (d, J = 8.6 Hz, 1H), 7.86 (s, 1H), 7.74 – 7.67 (m, 2H), 7.58 (dd, J = 8.3, 5.1 Hz, 1H), 7.30 (dd, J = 8.6, 1.7 Hz, 1H), 7.09 (ddd, J = 9.4, 8.3, 2.5 Hz, 1H), 4.01 (s, 2H), 2.49 (d, J = 1.0 Hz, 3H). **^{13}C-NMR (125 MHz, acetone-d$_6$)** δ/ppm 163.5 (d, J = 250 Hz, C), 149.3 (C), 144.6 (d, J = 9 Hz, C), 141.5 (d, J = 3 Hz, C), 140.2 (d, J = 2 Hz, C), 136.1 (d, J = 4 Hz, C), 127.9 (CH), 127.6 (CH), 127.5 (CH), 127.4 (CH), 124.2 (C), 122.9 (C), 122.7 (CH), 114.9 (d, J = 23 Hz, CH), 111.1 (CH), 108.1 (d, J = 23 Hz, CH), 33.8 (CH_2), 21.6 (CH_3). **^{19}F-NMR (376 MHz, acetone-d$_6$)** δ/ppm -117.9 (m). **IR (neat)** ν/cm^{-1}: 3386 (w), 1638 (w), 1617 (m), 1464 (m)m 1258 (s), 1082 (m), 864 (w), 808 (s), 619 (w). **HR-MS (QTOF ES+):** calcd for $C_{18}H_{14}FO$ 265.1023 found 265.1024.

3-Methyl-11*H*-benzo[*b*]fluoren-10-ol (5p)

Synthesised via Procedure C

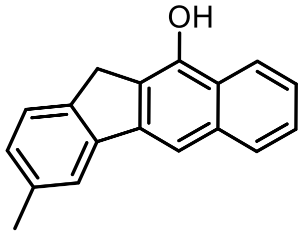

Chemical Formula: $C_{18}H_{14}O$
Exact Mass: 246.1045

Yield: 35% (38 mg, 0.15 mmol)

Appearance: Light-brown solid

Melting point: 132-134 °C

^{1}H-NMR (400 MHz, acetone-d$_6$) δ/ppm 8.70 (s, 1H), 8.32 (dt, *J* = 7.7, 1 Hz, 1H), 7.91 (dd, *J* = 8.0, 1.6 Hz, 1H), 7.89 (s, 1H), 7.87 – 7.69 (s, 1H), 7.50 – 7.40 (m, 3H), 7.17 (ddd, *J* = 7.6, 1.7, 1 Hz, 1H), 4.01 (s, 2H), 2.44 (s, 3H). **^{13}C-NMR (100 MHz, acetone-d$_6$)** δ/ppm 149.1 (C), 142.4 (C), 142.4 (C), 141.6 (C), 137.2 (C), 135.9 (C), 129.4 (CH), 128.7 (CH), 126.4 (CH), 125.9 (CH), 125.6 (C), 125.0 (CH), 123.3 (C), 122.7 (CH), 122.0 (CH), 110.9 (CH), 34.0 (CH_2), 21.5 (CH_3). **IR (neat)** ν/cm^{-1}: 3319 (m), 1638 (m), 1577 (m), 1372 (w), 1235 (s), 1085 (w), 846 (s), 734 (m). 610 (w). **HR-MS (QTOF ES+):** calcd for $C_{18}H_{15}O$ 247.1117 found 247.1118.

3,7-Difluoro-11*H*-benzo[*b*]fluoren-10-ol (5q)

Synthesised via Procedure C

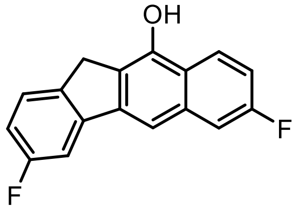

Chemical Formula: $C_{17}H_{10}F_2O$
Exact Mass: 268.0700

Yield: 33% (42 mg, 0.16 mmol)

Appearance: Light-brown solid

Melting point: 134-135 °C

^{1}H-NMR (400 MHz, acetone-d$_6$) δ/ppm 8.94 (s, 1H), 8.35 (dd, *J* = 9.3, 5.8 Hz, 1H), 7.92 (s, 1H), 7.70 (dd, *J* = 9.2, 2.5 Hz, 1H), 7.63 – 7.55 (m, 2H), 7.28 (ddd, *J* = 9.3, 8.5, 2.6 Hz, 1H), 7.12 (ddd, *J* = 9.4, 8.3, 2.5 Hz, 1H), 4.02 (s, 2H). **^{13}C-NMR (100 MHz, acetone-d$_6$)** δ/ppm 163.5 (d, *J* = 250 Hz, C), 161.8 (d, *J* = 250 Hz, C), 149.6 (C), 144.1 (d, *J* = 9 Hz, C), 143.0 (d, *J* = 3 Hz, C), 140.3 (d, *J* = 2 Hz, C), 136.8 (d, *J* = 9 Hz, C), 127.5 (d, *J* = 9 Hz, CH), 125.7 (d, *J* = 9 Hz, CH), 123.2 (C), 123.0 (C), 115.5 (d, *J* = 23 Hz, CH), 115.1 (d, *J* = 25 Hz, CH), 111.6 (d, *J* = 21 Hz, CH), 111.1 (d, *J* = 5 Hz, CH), 108.3 (d, *J* = 23 Hz, CH), 33.8 (CH_2). **^{19}F-NMR (376 MHz, acetone-d$_6$)** δ/ppm -117.48 (tt, *J* = 10, 6 Hz), -117.65 (td, *J* = 9, 5 Hz). **IR (neat)** ν/cm^{-1}: 3293 (w), 1643 (w), 1482 (m), 1259 (s), 1155 (s), 981 (w), 875 (m), 713 (w), 616 (m). **HR-MS (QTOF ES+):** calcd for $C_{17}H_{11}F_2O$ 269.0772 found 269.0773 ($M+H^+$).

3,7-Dimethyl-11*H*-benzo[*b*]fluoren-10-ol (5r)

Synthesised via Procedure C

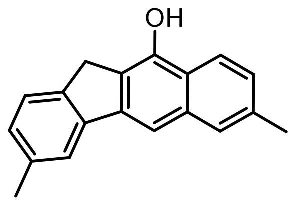

Chemical Formula: $C_{19}H_{16}O$
Exact Mass: 260.1201

Yield: 26% (19 mg, 0.07 mmol)

Appearance: Light-brown solid

Melting point: 167-168 °C

^{1}H-NMR (500 MHz, acetone-d*6*) δ/ppm 8.19 (d, *J* = 8.5 Hz, 1H), 7.80 (s, 1H), 7.77 (s, 1H), 7.68 (s, 1H), 7.46 (d, *J* = 7.9 Hz, 1H), 7.27 (dd, *J* = 8.6, 1.8 Hz, 1H), 7.17 (ddd, *J* = 7.6, 1.6, 1Hz, 1H), 3.98 (s, 2H), 2.49 (s, 3H), 2.44 (s, 3H). **^{13}C-NMR (125 MHz, acetone-d*6*)** δ/ppm 142.6 (C), 142.5 (C), 141.7 (C), 137.3 (C), 136.3 (C), 135.9 (C), 129.3 (CH), 127.8 (CH), 127.6 (C), 127.2 (CH), 126.0 (CH), 124.0 (C), 122.7 (CH), 122.5 (C), 122.0 (CH), 110.4 (CH), 34.0 (CH_2), 21.7 (CH_3), 21.6 (CH_3). **IR (neat)** v/cm^{-1}: 3388 (w), 1638 (w), 1580 (m), 1412 (w), 1258 (m), 1193 (m), 1082 (m), 884 (w), 810 (s), 819 (w). **HR-MS (QTOF ES+):** calcd for $C_{19}H_{17}O$ 261.1274 found 261.1275 (M+H^{+}).

6-Fluoro-11*H*-benzo[*b*]fluoren-10-ol (5s)

Synthesised via Procedure C

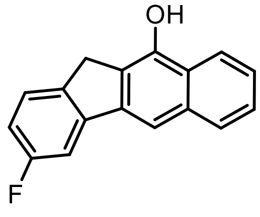

Chemical Formula: $C_{17}H_{11}FO$
Exact Mass: 250.0794

Yield: 31% (43 mg, 0.17 mmol)

Appearance: Light-brown solid

Melting point: 134 °C

^{1}H-NMR (500 MHz, acetone-d$_6$) δ/ppm 8.35 – 8.29 (m, 1H), 7.96 (s, 1H), 7.95 – 7.89 (m, 1H), 7.73 (dd, *J* = 9.2, 2.5 Hz, 1H), 7.63 – 7.57 (m, 1H), 7.52 – 7.44 (m, 2H), 7.11 (ddd, *J* = 9.4, 8.3, 2.5 Hz, 1H), 4.05 (s, 2H). **^{13}C-NMR (125 MHz, acetone-d$_6$)** δ/ppm 163.5 (d, *J* = 250 Hz, C), 149.3 (C), 144.5 (d, *J* = 9 Hz, C), 141.4 (d, *J* = 3 Hz, C), 140.1 (d, *J* = 2 Hz, C), 135.8 (C), 128.8 (CH), 127.50 (d, *J* = 9 Hz, CH), 126.6 (CH), 125.9 (C), 125.4 (CH), 123.6 (C), 122.7 (CH), 115.0 (d, *J* = 23.0 Hz, CH), 111.7 (CH), 108.1 (d, *J* = 23 Hz, CH), 33.9 (CH_2). **^{19}F-NMR** (**376 MHz, acetone-d$_6$)** δ/ppm -117.78 – -117.91 (m). **IR (neat)** v/cm^{-1}: 3292 (w), 1643 (w), 1580 (m), 1412 (m), 1260 (s), 1074 (w), 904 (w), 807 (m), 617 (m). **HR-MS (QTOF ES+):** calcd for $C_{17}H_{12}FO$ 251.0867 found 251.0867 (M+H^{+}).

Functionalisation of *11*H-Benzo[*b*]Benzofluoren-10-ol

5-Bromo-11*H*-benzo[b]fluoren-10-ol (6a)

Synthesised via Procedure D

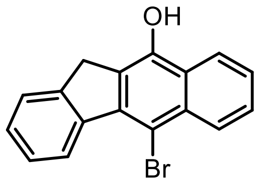

Chemical Formula: $C_{17}H_{11}BrO$
Exact Mass: 309.9993

Yield: 67% (57 mg, 0.18 mmol)

Appearance: Light-brown solid

Melting point: 132-133 °C

^{1}H-NMR (500 MHz, acetone-d$_6$) δ/ppm 8.93 (s, 1H), 8.89 (dd, *J* = 7.7, 1.3 Hz, 1H), 8.41 – 8.30 (m, 2H), 7.69 – 7.60 (m, 2H), 7.55 (ddd, *J* = 8.2, 6.8, 1.1 Hz, 1H), 7.50 – 7.46 (m, 1H), 7.43 (td, *J* = 7.4, 1.3 Hz, 1H), 4.13 (s, 2H). **^{13}C-NMR (125 MHz, acetone-d$_6$)** δ/ppm 148.8 (C), 145.4 (C), 142.0 (C), 140.4 (C), 133.9 (C), 129.1 (CH), 128.0 (CH), 127.3 (2CH), 126.6 (C), 126.1 (CH), 126.1 (CH), 125.6 (CH), 125.0 (C), 123.0 (CH), 107.0 (C), 34.7 (CH_2). **IR (neat)** v/cm^{-1}: 3228 (m), 1657 (m), 1594 (m), 1462 (w), 1331 (w), 1211 (s), 1152 (s), 1084 (m), 819 (w), 751 (s), 717 (s). **HR-MS (QTOF ES+):** calcd for $C_{17}H_{12}{}^{81}BrO_2$ 313.0047 found 313.0046 (M+H$^+$).

10-Methoxy-11*H*-benzo[*b*]fluorene (6b)

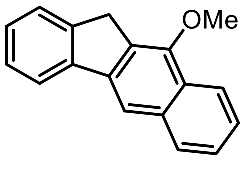

Chemical Formula: $C_{18}H_{14}O$
Exact Mass: 246.1045

Yield: 33% (37 mg, 0.15 mmol)

Note - Purification yield

Appearance: Off-brown liquid

^{1}H-NMR (400 MHz, CDCl$_3$) δ/ppm 8.27 – 8.17 (m, 1H), 7.99 (s, 1H), 7.96 – 7.89 (m, 2H), 7.59 (ddd, *J* = 7.3, 1.4, 0.7 Hz, 1H), 7.52 – 7.47 (m, 2H), 7.44 – 7.40 (m, 1H), 7.37 (td, *J* = 7.4, 1.3 Hz, 1H), 4.18 (s, 2H), 4.15 (s, 3H). **^{13}C-NMR (100 MHz, CDCl$_3$)** δ/ppm 151.8 (C), 143.3 (C), 141.7 (C), 140.9 (C), 134.8 (C), 128.5 (C), 128.1 (CH), 127.6 (CH), 127.2 (C), 126.9 (CH), 125.8 (CH), 125.2 (CH), 125.1 (CH), 121.9 (CH), 120.5 (CH), 113.7 (CH), 60.4 (CH_3), 34.4 (CH_2). **IR (neat)** v/cm^{-1}: 2981 (w), 1636 (w), 1500 (w), 1408 (w), 1337 (m), 1086 (m), 950 (w), 846 (w), 781 (s), 762 (s). **HR-MS (QTOF ES+):** calcd for $C_{18}H_{15}O$ 247.1117 found 247.1118 (M+H$^+$).

10-Methoxy-11H-benzo[b]fluoren-11-one (6c)

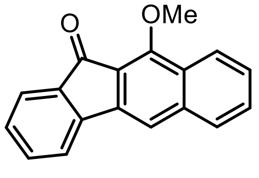

Chemical Formula: $C_{18}H_{12}O_2$
Exact Mass: 260.0837

Yield: 33% (37 mg, 0.14 mmol)

Note - Purification yield

Appearance: Bright-yellow solid

Melting point: 135 °C

^{1}H-NMR (500 MHz, $CDCl_3$) δ/ppm 8.33 – 8.28 (m, 1H), 7.77 – 7.75 (m, 1H), 7.74 – 7.72 (m, 1H), 7.70 (dt, J = 7.5, 0.9 Hz, 1H), 7.59 (s, 1H), 7.55 (qd, J = 7.2, 1.3 Hz, 2H), 7.46 (ddd, J = 8.2, 6.9, 1.2 Hz, 1H), 7.34 (td, J = 7.4, 0.9 Hz, 1H), 4.38 (s, 3H). **^{13}C-NMR (125 MHz, $CDCl_3$)** δ/ppm 190.5 (C), 157.1 (C), 144.0 (C), 139.2 (C), 138.0 (C), 136.2 (C), 134.3 (CH), 129.6 (CH), 129.5 (C), 129.0 (CH), 128.3 (CH), 126.5 (CH), 125.2 (CH), 124.0 (CH), 120.5 (CH), 118.4 (C), 114.3 (CH), 63.3 (CH_3). **IR (neat)** v/cm^{-1} **:** 3272 (w), 1695 (m), 1625 (m), 1508 (w), 1329 (m), 1163 (m), 1001 (m), 758 (s), 718 (s). **HR-MS (QTOF ES+):** calcd for $C_{18}H_{12}O_2$ 261.0912 found 261.0910 (M+H$^+$). In agreement with previously reported.[7]

5-Bromo-10-methoxy-11H-benzo[b]fluorene (6d)

Synthesised via Procedure D

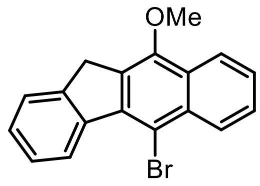

Chemical Formula: $C_{18}H_{13}BrO$
Exact Mass: 324.0150

Yield: 95% (50 mg, 0.15 mmol)

Appearance: Light-brown solid

Melting point: 117 °C

^{1}H-NMR (400 MHz, $CDCl_3$) δ/ppm 8.97 – 8.90 (m, 1H), 8.46 (ddd, J = 8.6, 1.2, 0.7 Hz, 1H), 8.19 (ddd, J = 8.2, 1.4, 0.6 Hz, 1H), 7.65 – 7.54 (m, 3H), 7.52 – 7.45 (m, 1H), 7.43 (td, J = 7.3, 1.2 Hz, 1H), 4.17 (s, 2H), 4.09 (s, 3H. **^{13}C-NMR (125 MHz, $CDCl_3$)** δ/ppm 151.2 (C), 144.0 (C), 141.0 (C), 139.9 (C), 133.4 (C), 131.4 (C), 128.2 (CH), 128.0 (C), 127.3 (CH), 127.0 (CH), 126.6 (CH), 126.0 (CH), 124.9 (CH), 124.9 (CH), 121.9 (CH), 111.4 (C), 60.8 (CH_3), 34.2 (CH_2). **IR (neat)** v/cm^{-1} **:** 2981 (w), 1708 (w), 1606 (w), 1498 (w), 1330 (m), 1178 (w), 1090 (m), 909 (m), 854 (w), 753 (s), 719 (s). **HR-MS (QTOF ES+):** calcd for $C_{18}H_{14}{}^{81}BrO$ 327.0204 found 327.017 (M+H$^+$).

5-Bromo-10-methoxy-11*H*-benzo[*b*]fluoren-11-one (6e)

Synthesised via Procedure D

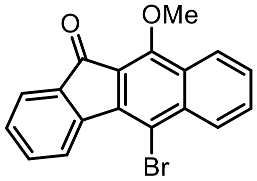

Chemical Formula: $C_{18}H_{11}BrO_2$
Exact Mass: 337.9942

Yield: 76% (33 mg, 0.10 mmol)

Appearance: Bright-yellow solid

Melting point: 172 °C

^{1}H-NMR (400 MHz, CDCl$_3$) δ/ppm 8.76 (dt, *J* = 7.8, 0.8 Hz, 1H), 8.38 – 8.30 (m, 2H), 7.81 (ddd, *J* = 7.4, 1.3, 0.7 Hz, 1H), 7.70 (ddd, *J* = 8.5, 6.9, 1.4 Hz, 1H), 7.63 (td, *J* = 7.7, 1.3 Hz, 1H), 7.56 (ddd, *J* = 8.2, 7.0, 1.2 Hz, 1H), 7.43 (td, *J* = 7.4, 0.9 Hz, 1H), 4.30 (s, 3H). **^{13}C-NMR (100 MHz, CDCl$_3$)** δ/ppm 189.6 (C), 156.6 (C), 143.7 (C), 137.4 (C), 136.9 (C), 136.7 (C), 134.3 (CH), 130.7 (CH), 130.5 (C), 129.6 (CH), 127.8 (CH), 127.5 (CH), 125.2 (CH), 124.8 (CH), 124.0 (CH), 121.2 (C), 112.6 (C), 63.5 (CH$_3$). **IR (neat)** v/cm^{-1} **:** 2981 (m), 1694 (s), 1508 (m), 1470 (m), 1369 (m), 1106 (w), 962 (m), 961 (w), 754 (s), 718 (s), 546 (w). **HR-MS (QTOF ES+):** calcd for $C_{18}H_{12}{}^{81}BrO_2$ 340.9997 found 340.9995 (M+H$^+$).

10-Methoxy-5-nitro-11H-benzo[*b*]fluoren-11-one (6f)

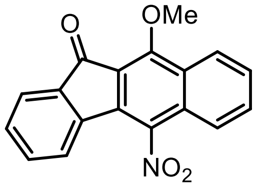

Chemical Formula: $C_{18}H_{11}NO_4$
Exact Mass: 305.0688

Yield: 58% (20 mg, 0.07 mmol)

Appearance: Bright-yellow solid

Melting point: 206 °C

^{1}H-NMR (400 MHz, CDCl$_3$) δ/ppm 8.40 (dt, *J* = 8.3, 0.9 Hz, 1H), 7.81 (d, *J* = 7.4 Hz, 1H), 7.75 – 7.70 (m, 1H), 7.66 (dt, *J* = 8.2, 1.1 Hz, 1H), 7.61 (ddd, *J* = 8.2, 6.7, 1.4 Hz, 2H), 7.57 (dd, *J* = 3.0, 0.9 Hz, 1H), 7.47 (ddd, *J* = 7.5, 5.3, 3.0 Hz, 1H), 4.43 (s, 3H). **^{13}C-NMR (100 MHz, CDCl$_3$)** δ/ppm 188.2 (C), 157.9 (C), 138.7 (C), 137.4 (C), 136.3 (C), 135.0 (CH), 131.6 (CH), 130.8 (CH), 130.2 (C), 129.7 (C), 128.9 (C), 128.1 (CH), 125.6 (CH), 124.5 (CH), 122.9 (CH), 122.2 (CH), 117.3 (C), 64.2 (CH$_3$). **IR (neat)** v/cm^{-1} **:** 2981 (s), 2899 (w), 1699 (m), 1575 (m), 1463 (w), 1373 (w), 1292 (w), 11144 (w), 952 (m), 774 (w), 753 (s), 715 (s). **HR-MS (QTOF ES+):** calcd for $C_{18}H_{12}NO_4$ 306.0761 found 306.0761 (M+H$^+$).

X-Ray data

Experimental

Crystals of **5a** and **5m** were mounted on a MiTeGen micromount with NVH immersion oil. Data were collected from a shock-cooled single crystal at 100(2) K on a Bruker D8 Quest ECO three-circle diffractometer with a sealed X-ray tube using a graphite monochromator and a Bruker PHOTON III C7 detector. The diffractometer was equipped with an Oxford Cryostream 800 low temperature device and used Mo K_α radiation (λ = 0.71073 Å). All data were integrated with SAINT and a multi-scan absorption correction using SADABS was applied.[1,2]

Structures were solved by dual methods using SHELXT and refined by full-matrix least-squares methods against F^2 by SHELXL using Olex2.[3-5] All non-hydrogen atoms were refined with anisotropic displacement parameters. All hydrogen atoms were refined with isotropic displacement parameters. Some of their coordinates were refined freely and some on calculated positions using a riding model with their U_{iso} values constrained to 1.5 times the U_{eq} of their pivot atoms for terminal sp^3 carbon atoms and 1.2 times for all other carbon atoms.

In **5a**, donor O-H hydrogens were located on the difference map and refined with restraints.

In **5m**, the absolute structure cannot be determined reliably. Donor O-H hydrogens atom were located on the difference map and refined with restraints (DFIX).

Crystallographic data for the structures reported here have been deposited with the Cambridge Crystallographic Data Centre.[6] CCDC 2392681-2392682 contain the supplementary crystallographic data for this paper. These data can be obtained free of charge from The Cambridge Crystallographic Data Centre via www.ccdc.cam.ac.uk/structures.

[1] Bruker, *SAINT*, Bruker AXS Inc., Madison, Wisconsin, USA.
[2] L. Krause, R. Herbst-Irmer, G. M. Sheldrick, D. Stalke, *J. Appl. Cryst.* **2015**, *48*, 3–10, doi:10.1107/S1600576714022985.
[3] G. M. Sheldrick, *Acta Cryst.* **2015**, *A71*, 3–8, doi:10.1107/S2053273314026370.
[4] G. M. Sheldrick, *Acta Cryst.* **2015**, *C71*, 3–8, doi:10.1107/S2053229614024218.
[5] O. V. Dolomanov, L. J. Bourhis, R. J. Gildea, J. A. K. Howard, H. Puschmann, *J. Appl. Cryst.* **2009**, *42*, 339-341, doi:10.1107/S0021889808042726.
[6] C. R. Groom, I. J. Bruno, M. P. Lightfoot, S. C. Ward, *Acta Cryst.* **2016**, *B72*, 171–179, doi:10.1107/S2052520616003954.

Structure Tables

	5a	5m
CCDC number	2392681	2392682
Empirical formula	$C_{18}H_{14}O_2$	$C_{18}H_{13}FO_2$
Formula weight	262.29	280.28
Temperature [K]	100(2)	100(2)
Crystal system	monoclinic	monoclinic
Space group (number)	$P2_1/n$ (14)	$P2_1$ (4)
a [Å]	14.1101(4)	11.7047(11)
b [Å]	4.79870(10)	3.9372(4)
c [Å]	19.2308(6)	14.0827(13)
α [°]	90	90
β [°]	110.2074(8)	99.725(2)
γ [°]	90	90
Volume [$Å^3$]	1221.97(6)	639.66(11)
Z	4	2
ρ_{calc} [gcm^{-3}]	1.426	1.455
μ [mm^{-1}]	0.092	0.104
F(000)	552	292
Crystal size [mm^3]	0.531×0.156×0.098	0.506×0.085×0.067
Crystal colour	yellow	colourless
Crystal shape	block	block
Radiation	Mo K_α (λ=0.71073 Å)	Mo K_α (λ=0.71073 Å)
2Θ range [°]	6.15 to 62.08 (0.69 Å)	5.87 to 62.14 (0.69 Å)
Index ranges	-20 ≤ h ≤ 20<br>-6 ≤ k ≤ 6<br>-27 ≤ l ≤ 27	-16 ≤ h ≤ 16<br>-5 ≤ k ≤ 5<br>-20 ≤ l ≤ 20
Reflections collected	37255	24446
Independent reflections	3894<br>R_{int} = 0.0369<br>R_{sigma} = 0.0237	4080<br>R_{int} = 0.0569<br>R_{sigma} = 0.0386
Completeness	99.9 %	99.7 %
Data / Restraints / Parameters	3894/1/186	4080/2/195
Goodness-of-fit on F^2	1.034	1.041
Final *R* indexes [$I \geq 2\sigma(I)$]	R_1 = 0.0431<br>wR_2 = 0.1062	R_1 = 0.0440<br>wR_2 = 0.1054
Final *R* indexes [all data]	R_1 = 0.0593<br>wR_2 = 0.1172	R_1 = 0.0567<br>wR_2 = 0.1143
Largest peak/hole [$eÅ^{-3}$]	0.42/-0.32	0.30/-0.22
Flack X parameter	-	0.3(5)

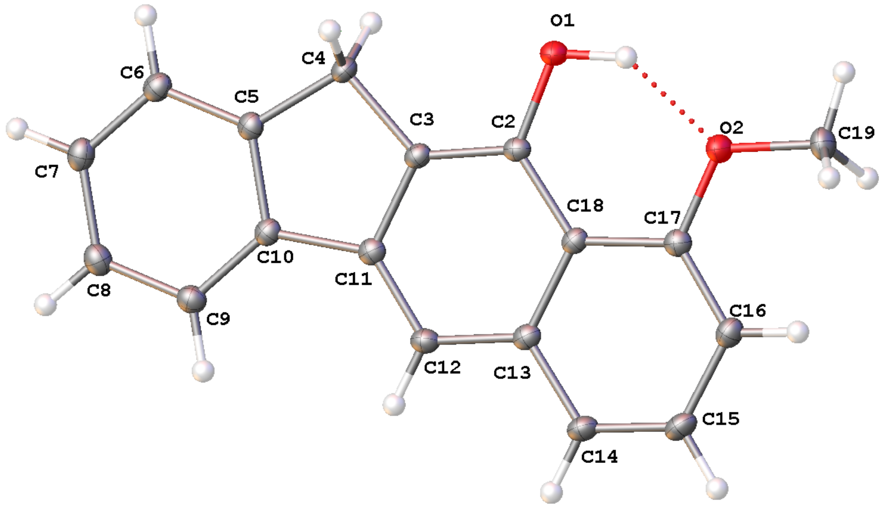

Fig. S1. Fully labelled molecular structure of **5a** with intramolecular hydrogen bonding. Displacement parameters shown at 50% probability.

Hydrogen bonding for 5a

D–H···A [Å]	d(D–H) [Å]	d(H···A) [Å]	d(D···A) [Å]	<(DHA) [°]
O1–H1···O1[#1]	0.968(9)	2.447(17)	3.1088(10)	125.4(14)
O1–H1···O2	0.968(9)	1.784(14)	2.6018(11)	140.1(17)

Symmetry transformations used to generate equivalent atoms:
#1: 0.5-X, -0.5+Y, 1.5-Z;

In **5a**, an intramolecular hydrogen bond is seen between O1 and O2, and also an intermolecular hydrogen bond between O1 and the symmetry generated O1 with an almost perpendicular arrangement (ca. 91.9 ° between the molecular planes). The graph set descriptor for these are $\mathrm{S}_1^1(6)$ and $\mathrm{C}_1^1(2)$. These hydrogen bonding interactions form a twisted infinite ribbon along the b-axis direction.

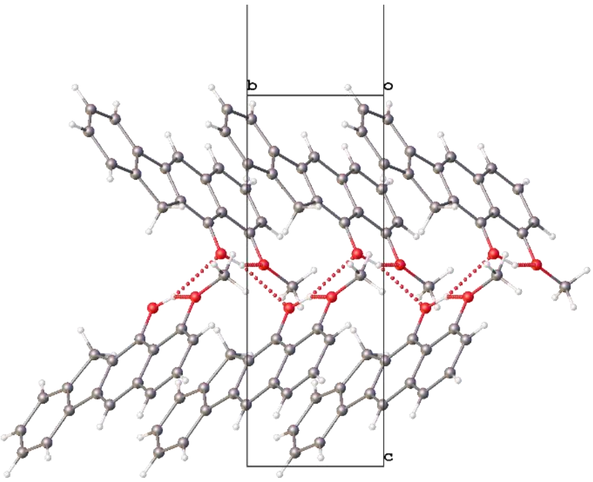

Fig. S2. Hydrogen bonding network in **5a** (dotted lines) viewed down the (101) direction.

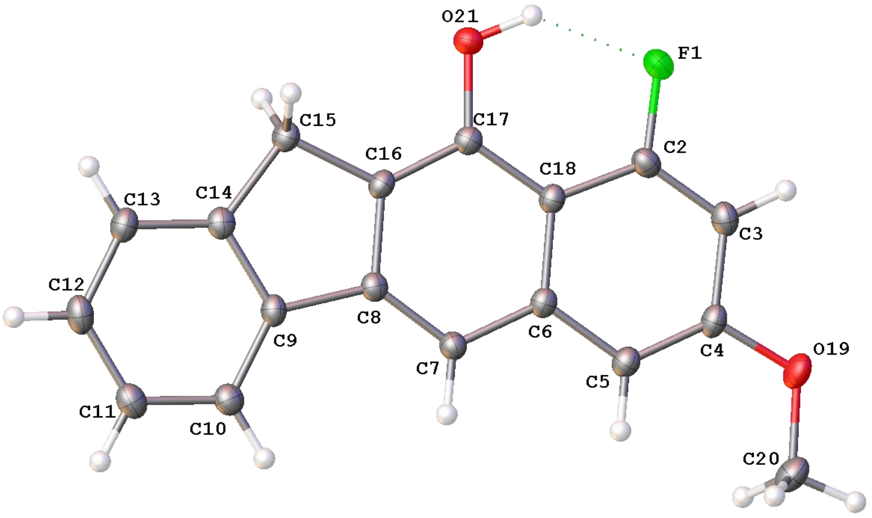

Fig. S3. Fully labelled molecular structure of **5m** with intramolecular hydrogen bonding. Displacement parameters shown at 50% probability.

Hydrogen bonding for 5m

D–H···A [Å]	d(D–H) [Å]	d(H···A) [Å]	d(D···A) [Å]	<(DHA) [°]
O21–H21···F1	0.943(13)	1.94(2)	2.6925(19)	135(3)
O21–H21···O21#1	0.943(13)	2.22(3)	2.818(2)	121(3)

Symmetry transformations used to generate equivalent atoms:
#1: 1-X, -0.5+Y, 2-Z;

There is intramolecular hydrogen bonding in **5m** between the hydroxyl OH and the fluorine forming a $S_1^1(6)$ graph set motif and there is a similar intermolecular interaction between the hydroxyl groups seen in **5a**, forming a $C_1^1(2)$ infinite chain. In this case however, the angle between the planes defined by the non-hydrogen atoms in the molecules are approx. 56°. This ruffled ribbon also extends along the b-axis.

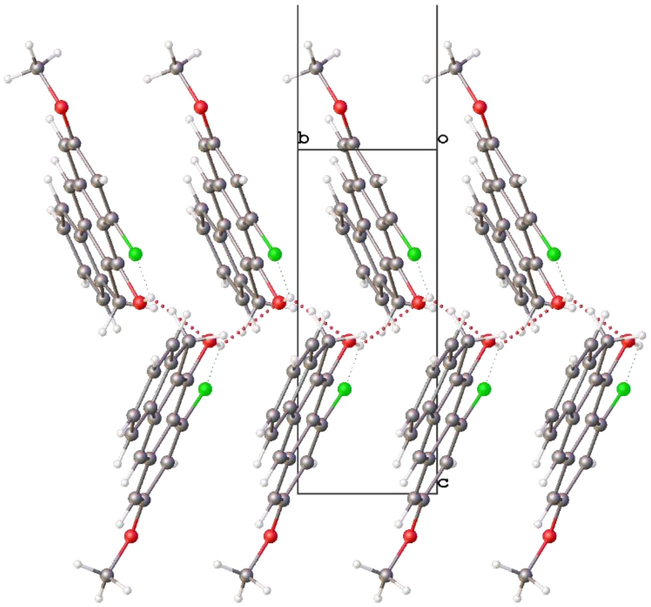

Fig. S4. Hydrogen bonding network in **5m** (dotted lines) viewed down the (101) direction.

References

(1) Chen, Z. Z.; Liu, S.; Hao, W. J.; Xu, G.; Wu, S.; Miao, J. N.; Jiang, B.; Wang, S. L.; Tu, S. J.; Li, G. Catalytic arylsulfonyl radical-triggered 1,5-enyne-bicyclizations and hydrosulfonylation of alpha,beta-conjugates. *Chem. Sci.* **2015**, *6* (11), 6654-6658. DOI: 10.1039/c5sc02343b.

(2) Antoniak, D.; Barbasiewicz, M. Corey–Chaykovsky Cyclopropanation of Nitronaphthalenes: Access to Benzonorcaradienes and Related Systems. *Org. Lett.* **2019**, *21* (23), 9320-9325. DOI: 10.1021/acs.orglett.9b03375.

(3) Gao, B.; Liu, S.; Lan, Y.; Huang, H. Rhodium-Catalyzed Cyclocarbonylation of Ketimines via C–H Bond Activation. *Organometallics* **2016**, *35* (10), 1480-1487. DOI: 10.1021/acs.organomet.6b00072.

(4) Ye, F.; Tran, C.; Jullien, L.; Le Saux, T.; Haddad, M.; Michelet, V.; Ratovelomanana-Vidal, V. Synthesis of Fluorescent Azafluorenones and Derivatives via a Ruthenium-Catalyzed [2 + 2 + 2] Cycloaddition. *Org. Lett.* **2018**, *20* (16), 4950-4953. DOI: 10.1021/acs.orglett.8b02085.

(5) Ji, K.; Zheng, Z.; Wang, Z.; Zhang, L. Enantioselective Oxidative Gold Catalysis Enabled by a Designed Chiral P,N-Bidentate Ligand. *Angew. Chem. Int. Ed.* **2015**, *54* (4), 1245-1249. DOI: https://doi.org/10.1002/anie.201409300.

(6) Sateesh, R.; Prudhviraj, J.; Priyanka, C.; Punna, N. Access to CF3-benzofulvenes via palladium-catalyzed cascade arylation/Trost–Oppolzer cyclization/double-bond isomerization. *Chem. Commun.* **2024**, *60* (26), 3551-3554. DOI: 10.1039/D3CC06082A.

(7) Patra, A.; Ghorai, S. K.; De, S. R.; Mal, D. Regiospecific Synthesis of Benzo[*b*]fluorenones via Ring Contraction by Benzil-Benzilic Acid Rearrangement of Benz[*a*]anthracene-5,6-diones. *Synthesis* **2006**, *2006* (15), 2556-2562. DOI: 10.1055/s-2006-942468.

Copies of NMR Spectra

4-Methyl-2-((trimethylsilyl)ethynyl)benzaldehyde (S1)

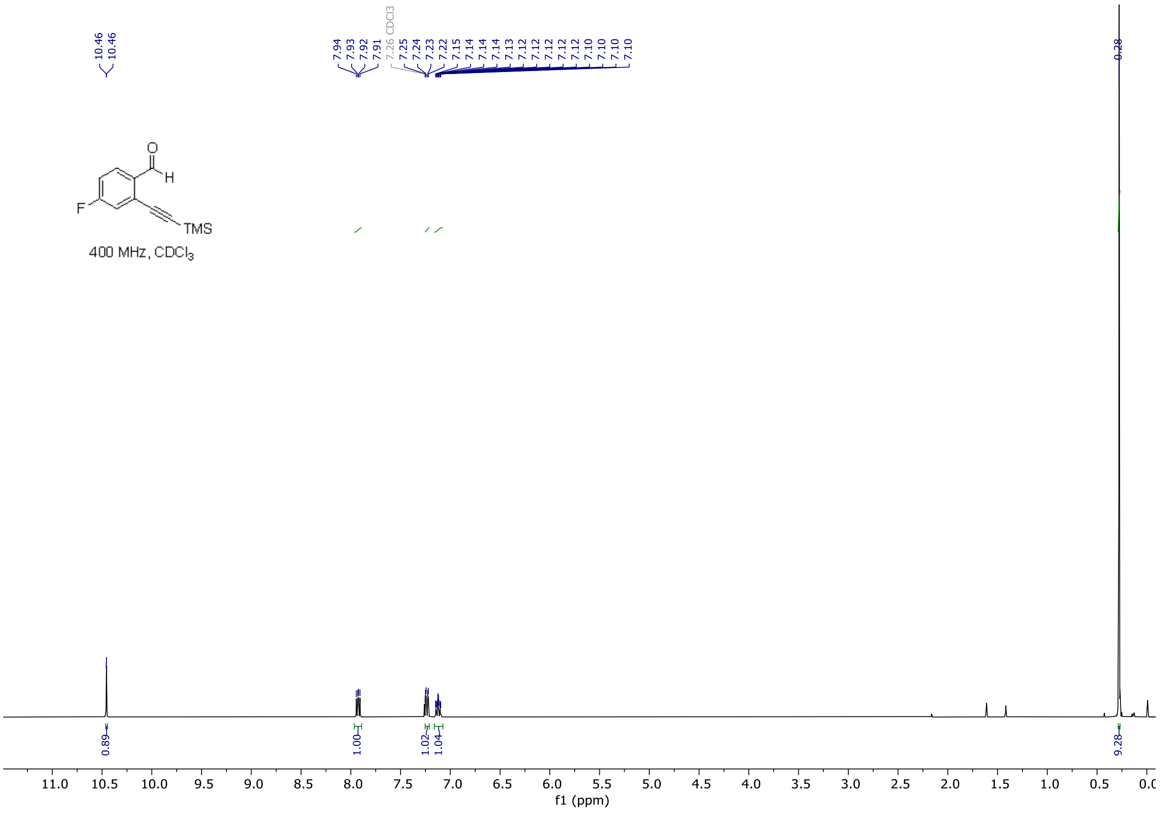

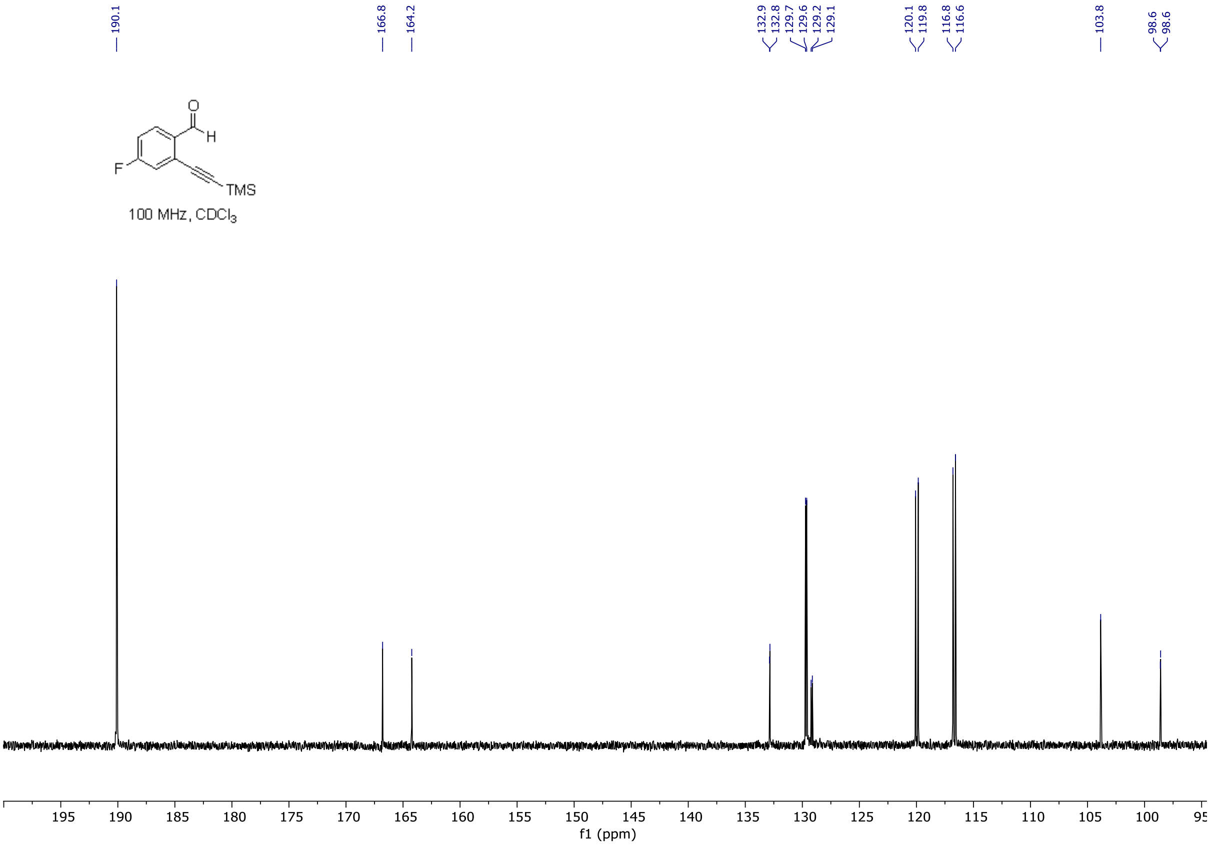

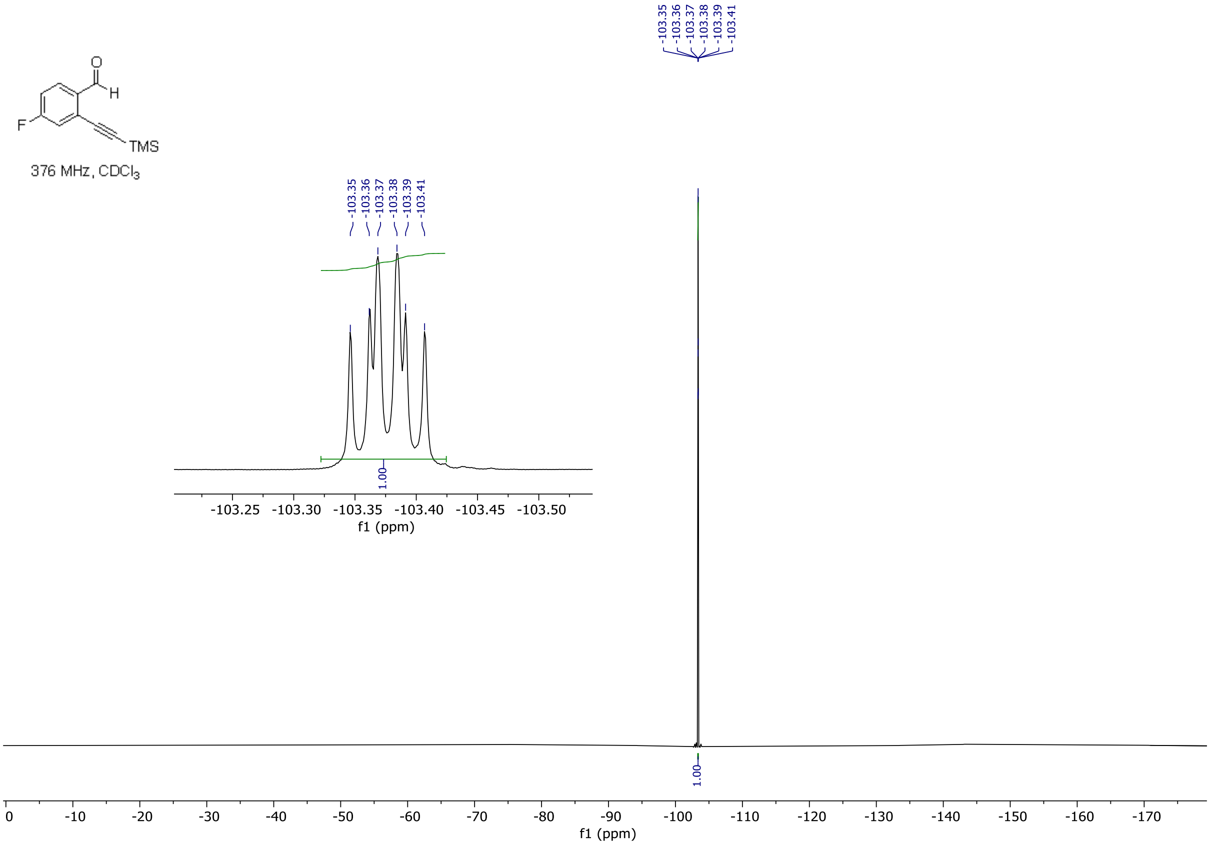

4-methyl-2-((trimethylsilyl)ethynyl)benzaldehyde (S2)

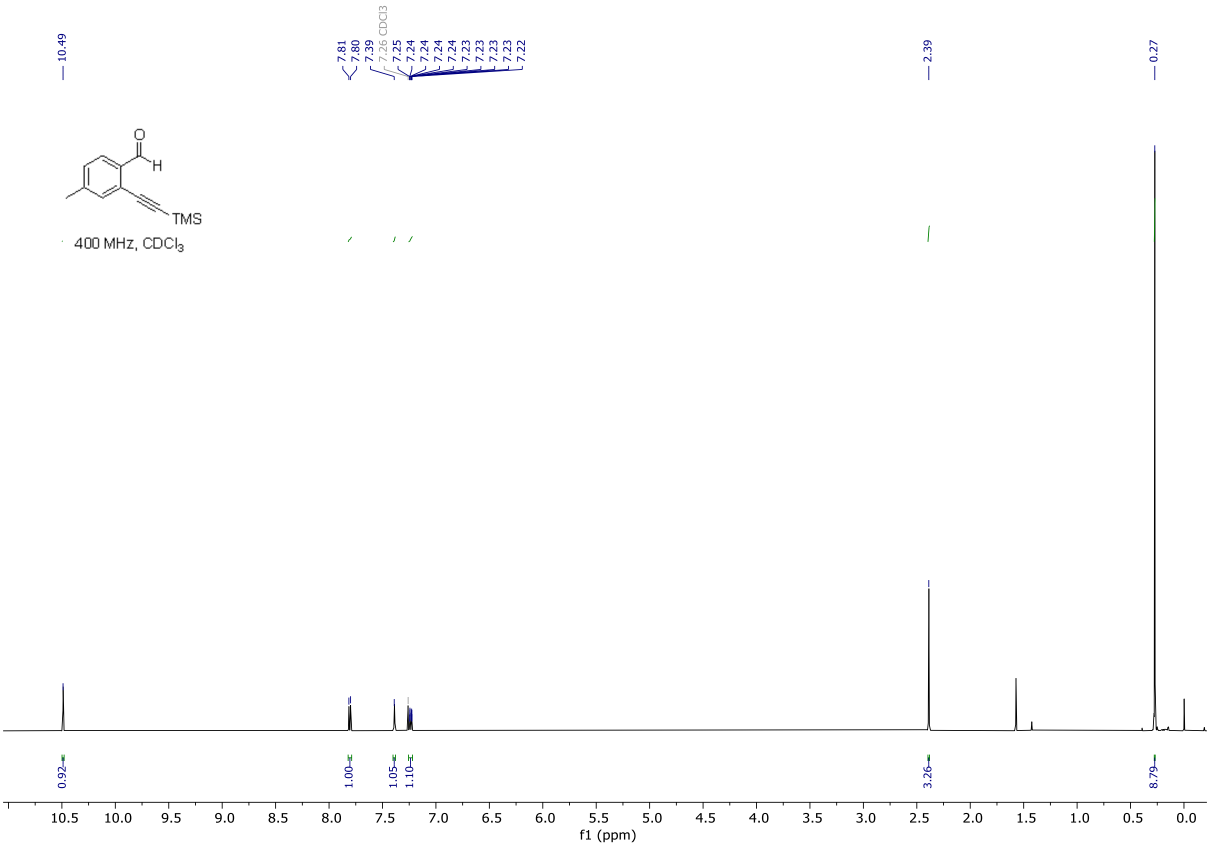

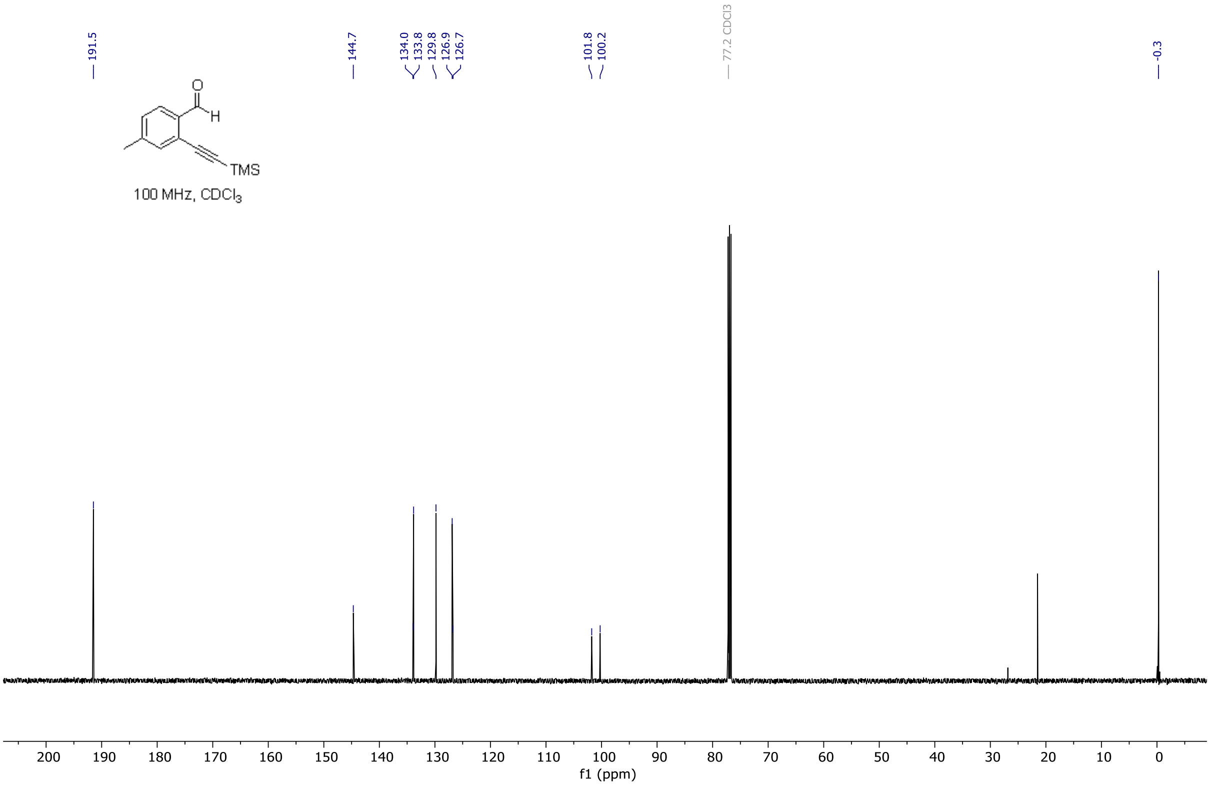

1-(Phenyl-d_5)ethan-1-one (S3)

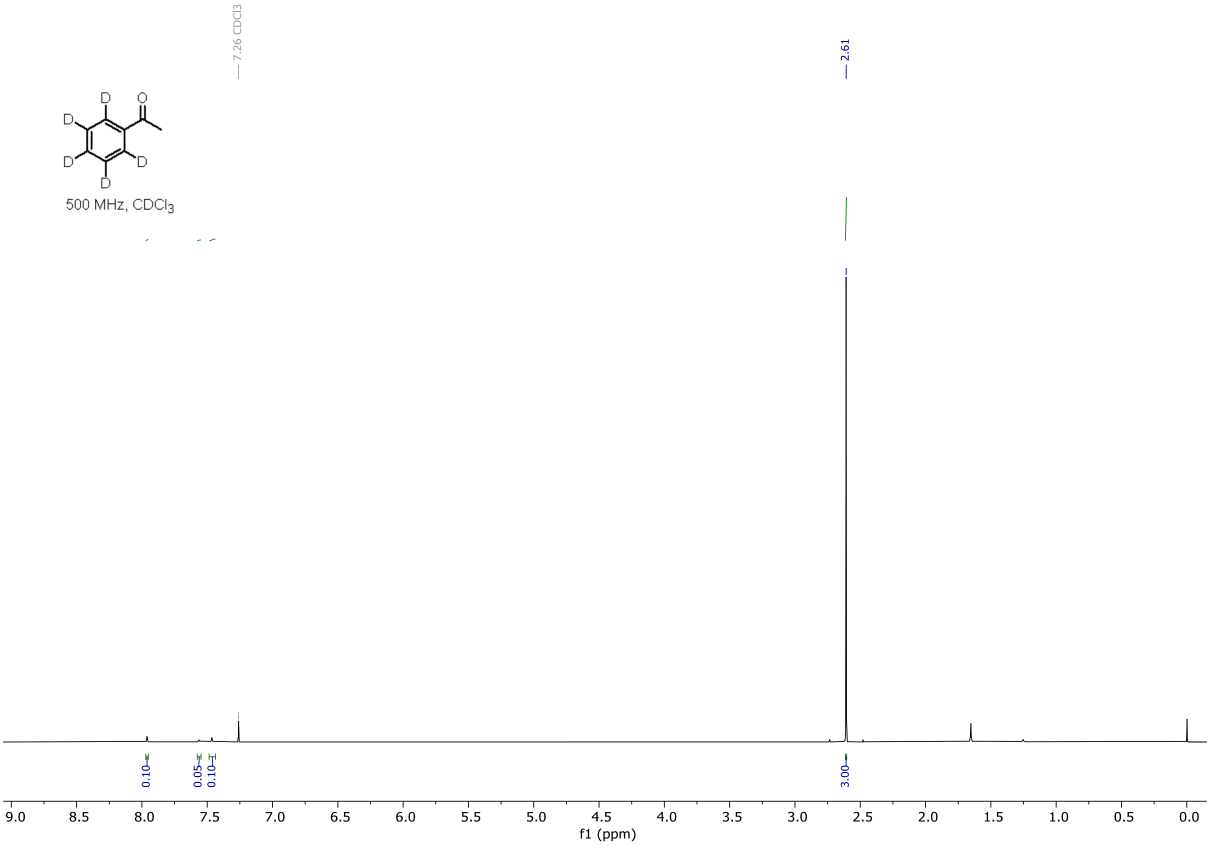

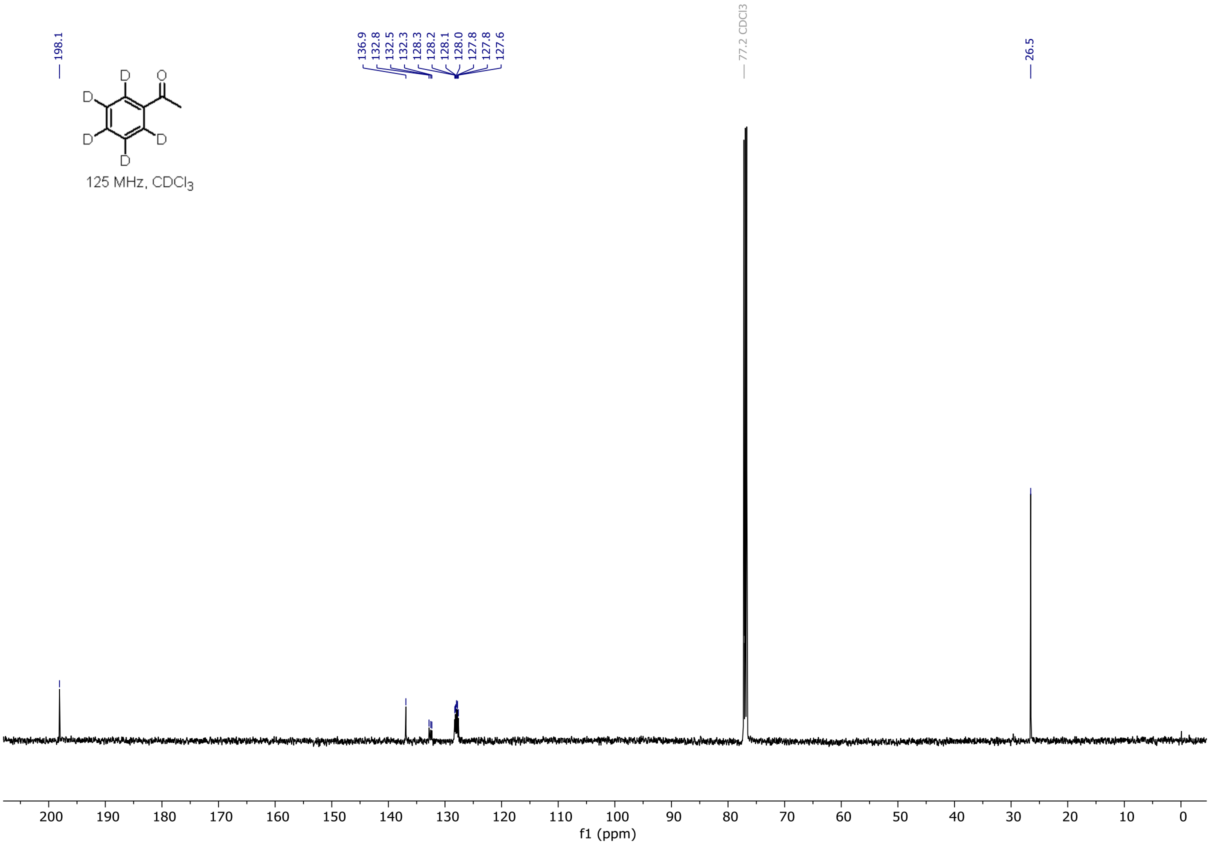

(*E*)-3-(2-(Ethynyl-d)phenyl)-1-phenylprop-2-en-1-one (4b-D$_1$)

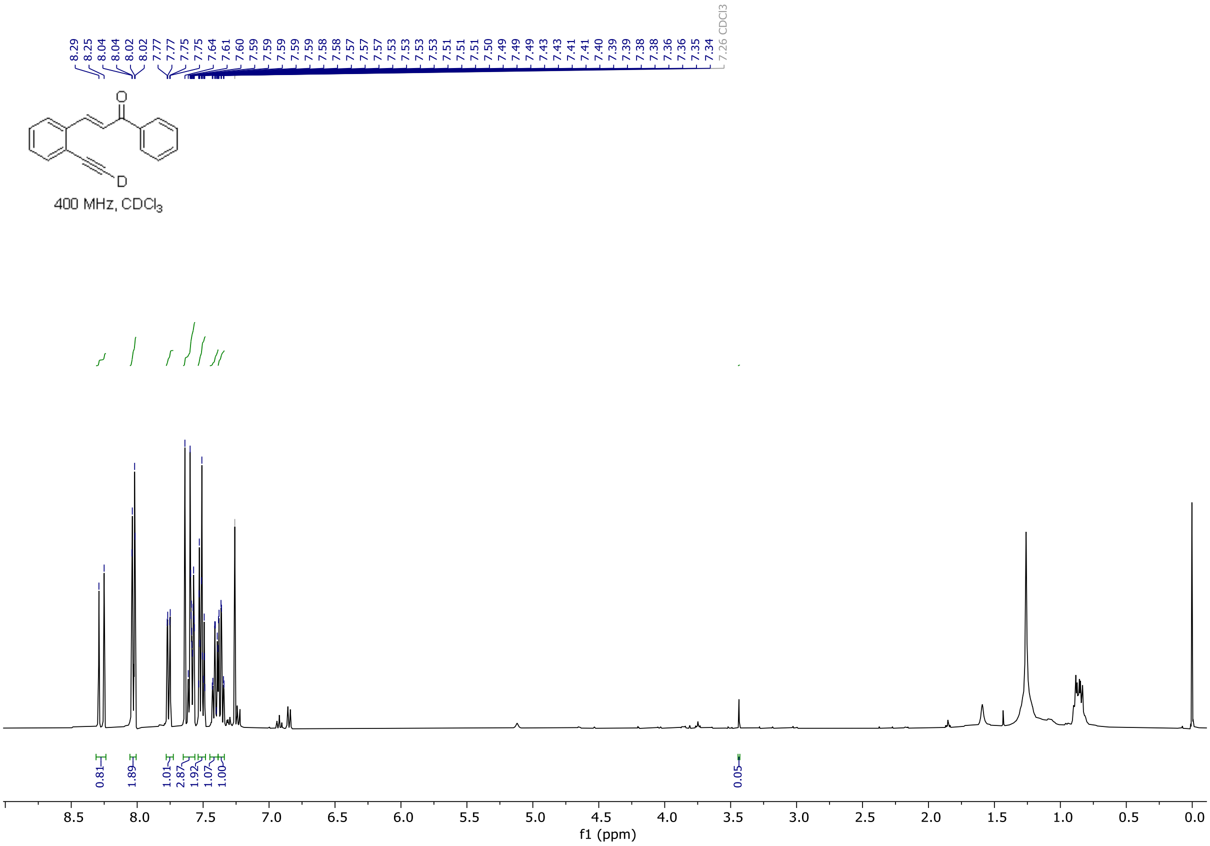

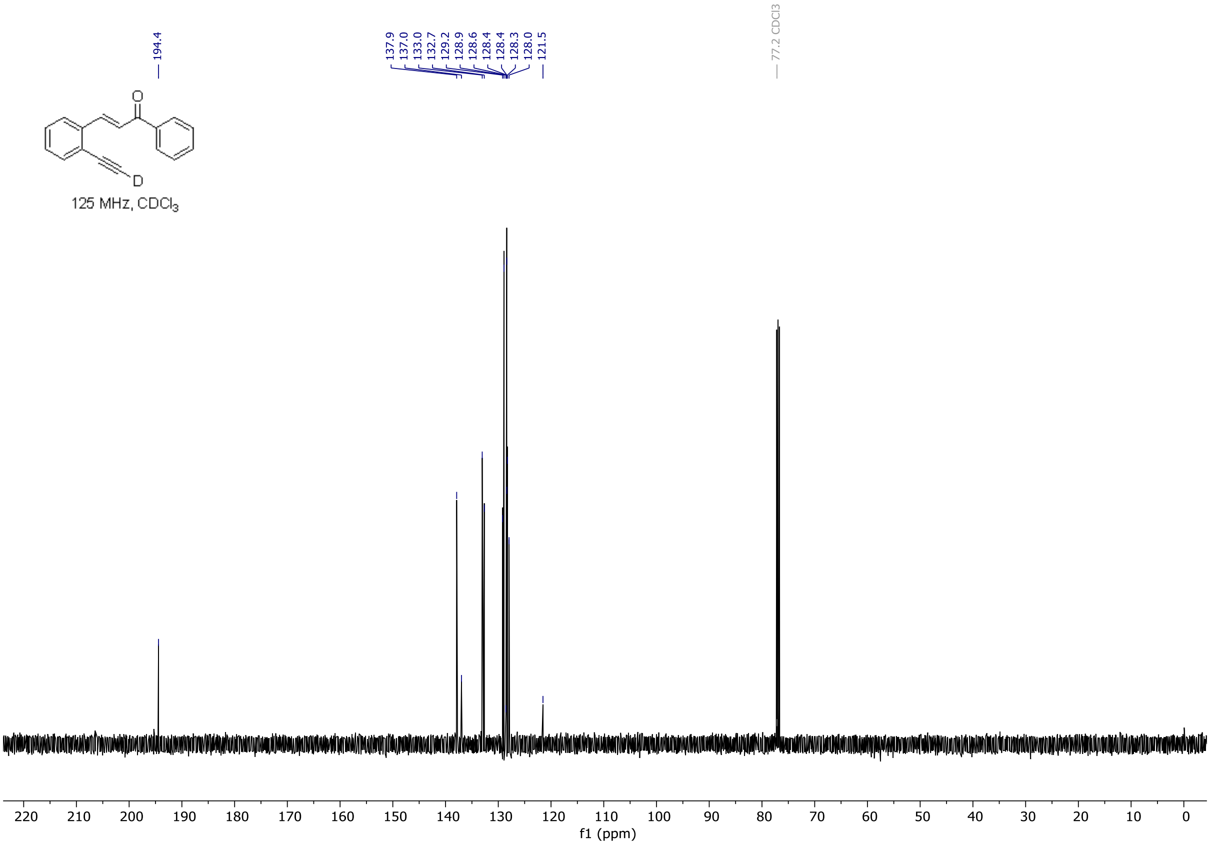

(*E*)-3-(2-Ethynylphenyl)-1-(phenyl-d5)prop-2-en-1-one (4b-D_5)

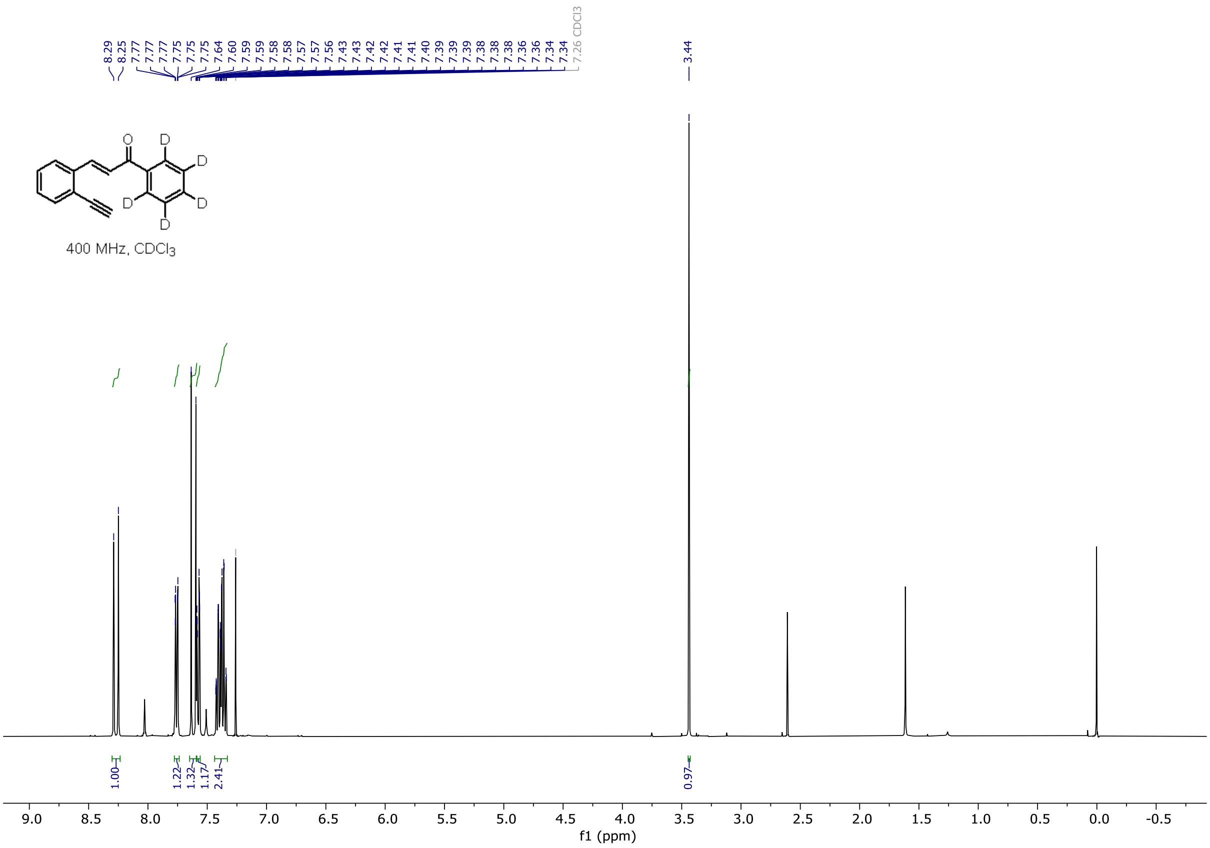

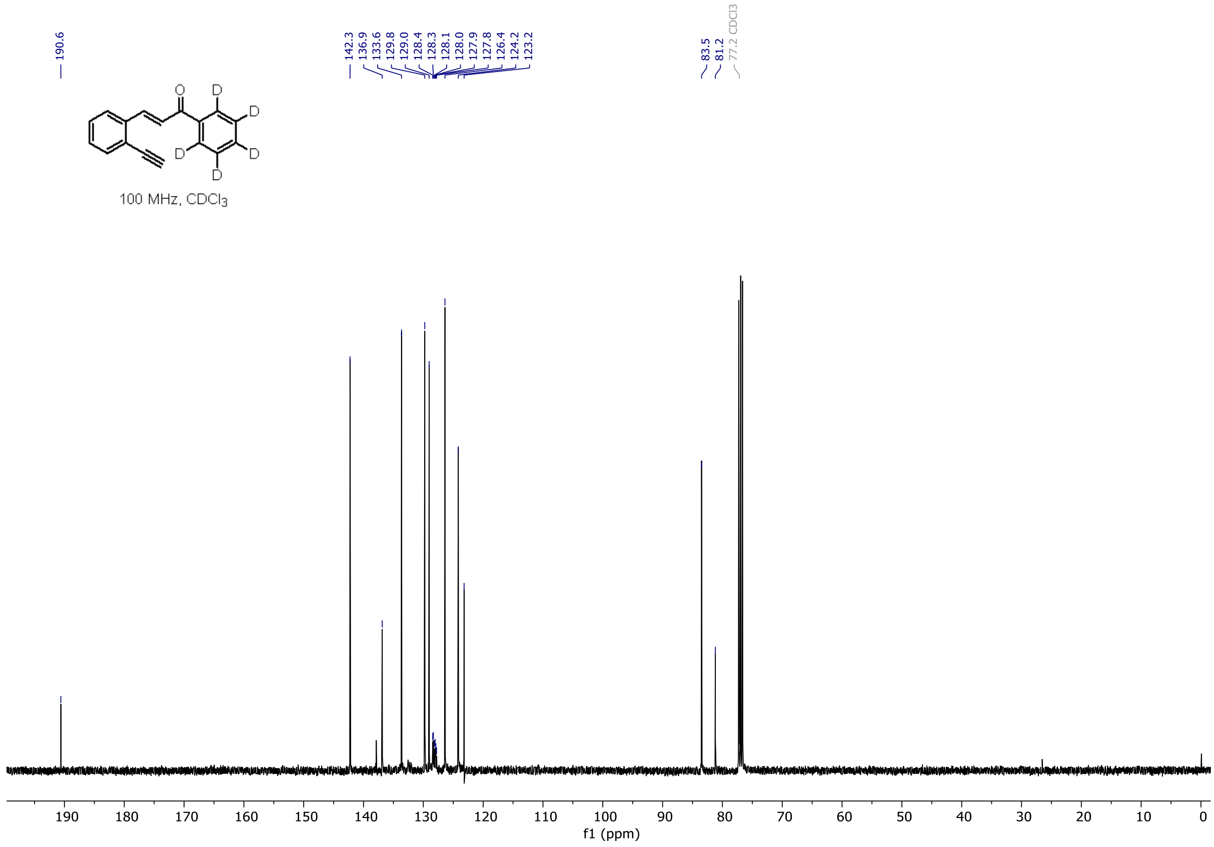

11*H*-Benzo[*b*]fluoren-6,7,8,9,11-d5-10-ol (5b-D_5)

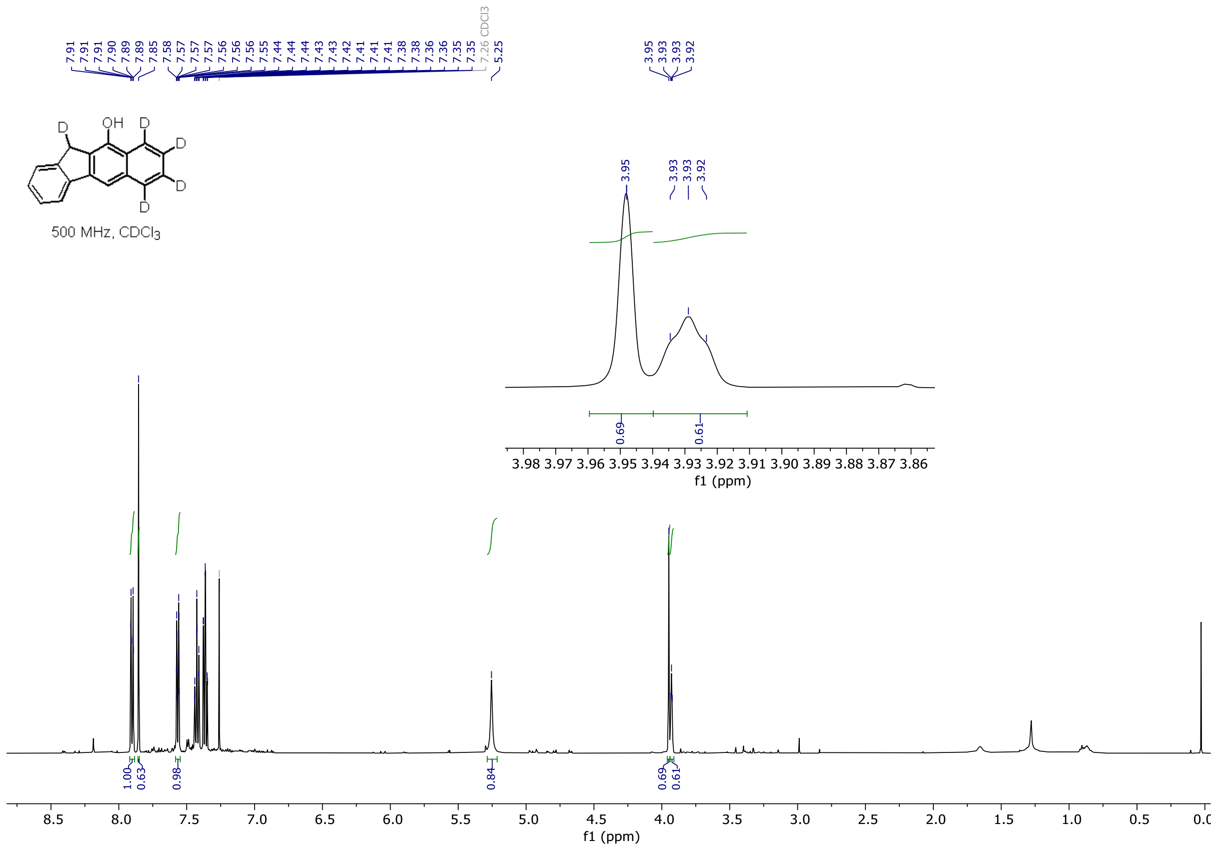

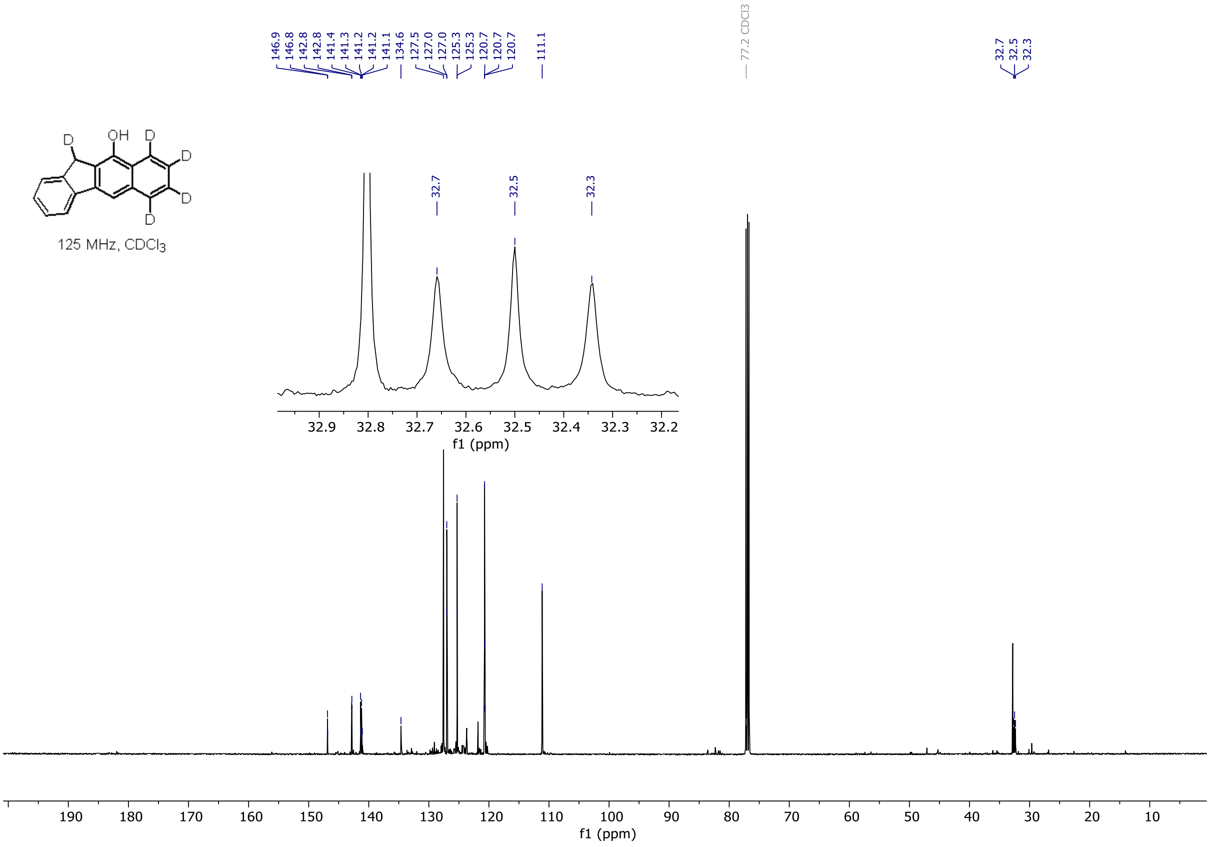

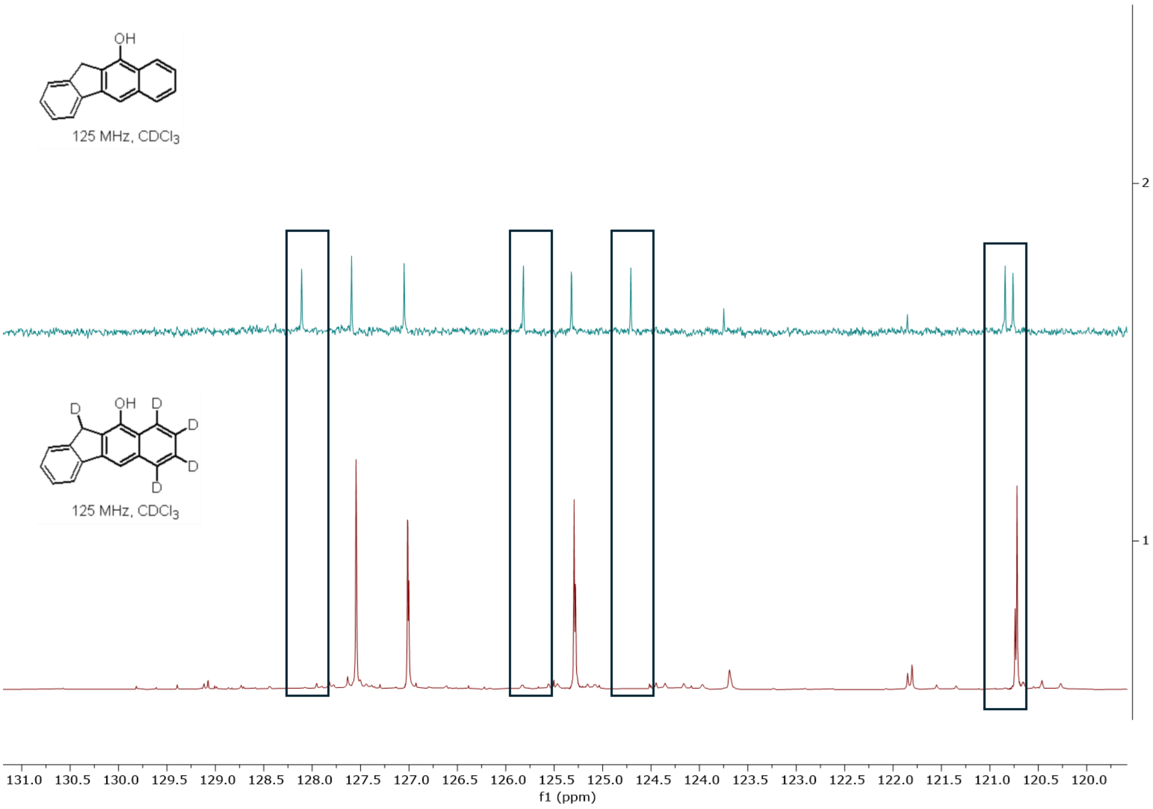

Stacked image of **5b** and **5b-D₅**. Highlighting where four C-H bonds of **5b** resonate in the ^{13}C-NMR which have been replaced by C-D bonds in **5b-D₅**.

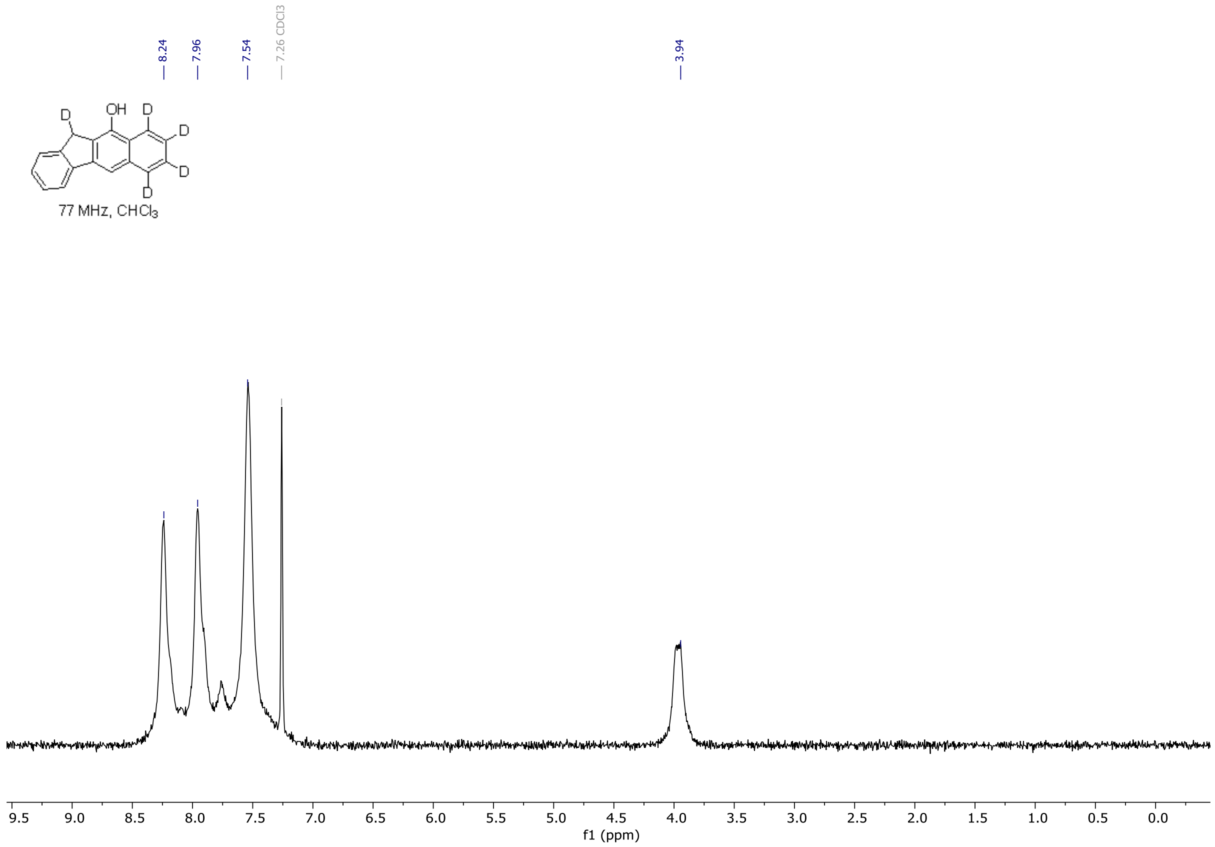

11*H*-Benzo[*b*]fluoren-5-d-10-ol (5b-D_1)

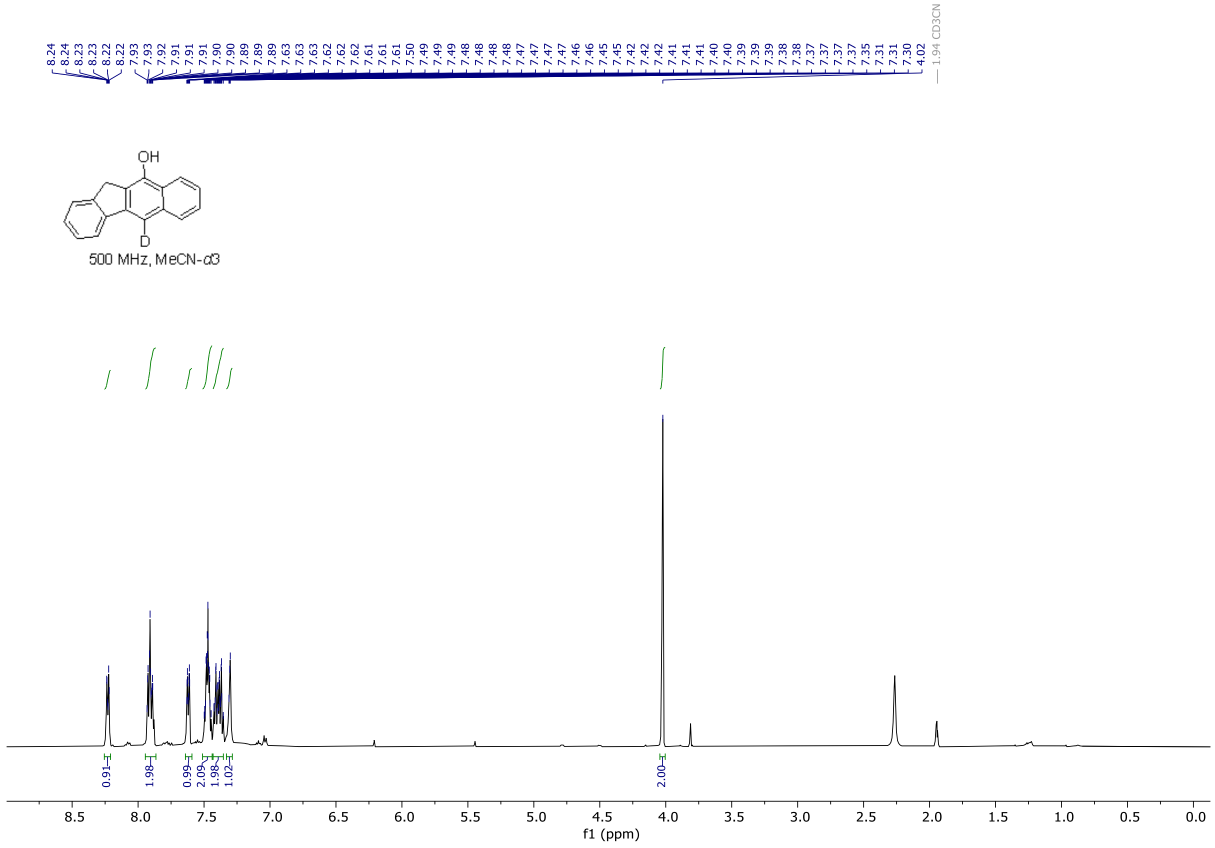

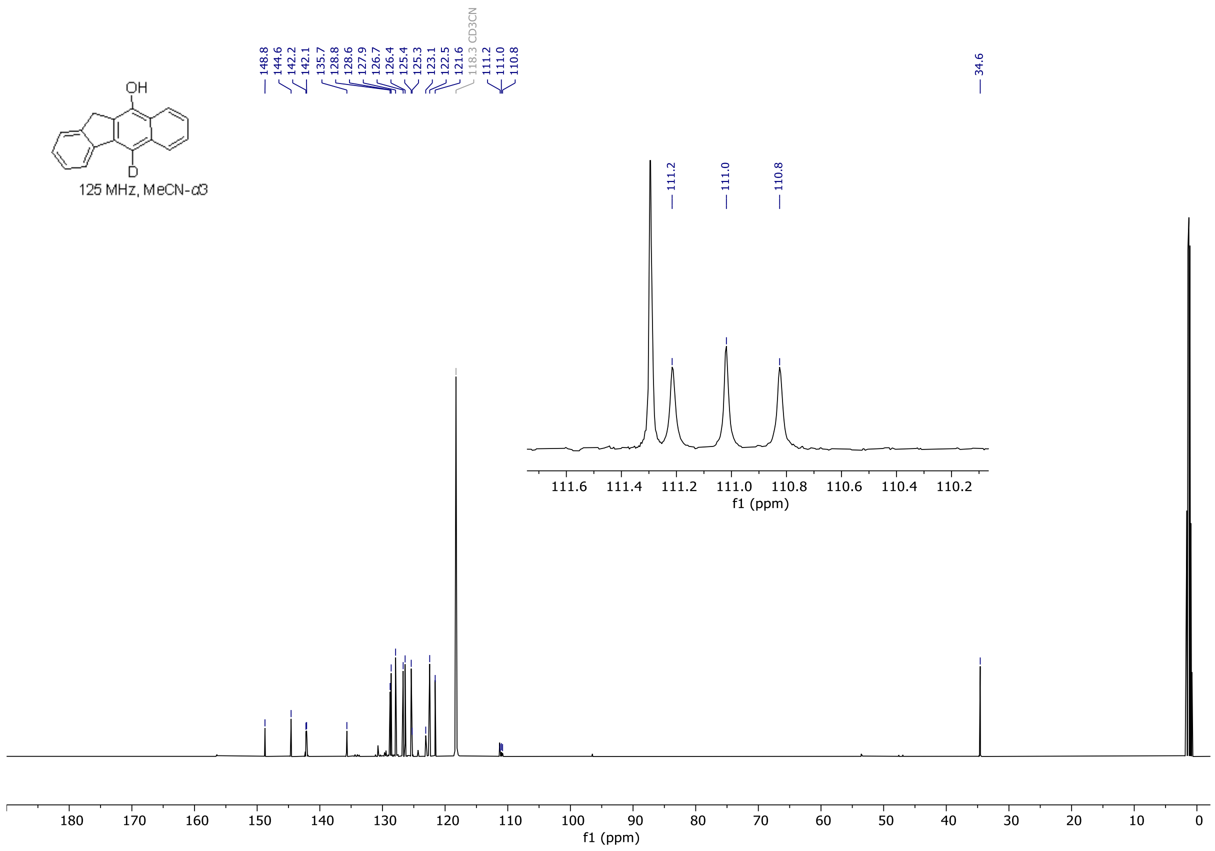
OH
D
125 MHz, MeCN-d3
148.8
144.6
142.2
142.1
135.7
128.8
128.6
127.9
126.7
126.4
125.4
125.3
123.1
122.5
121.6
118.3 CD3CN
111.2
111.0
110.8
34.6
f1 (ppm)

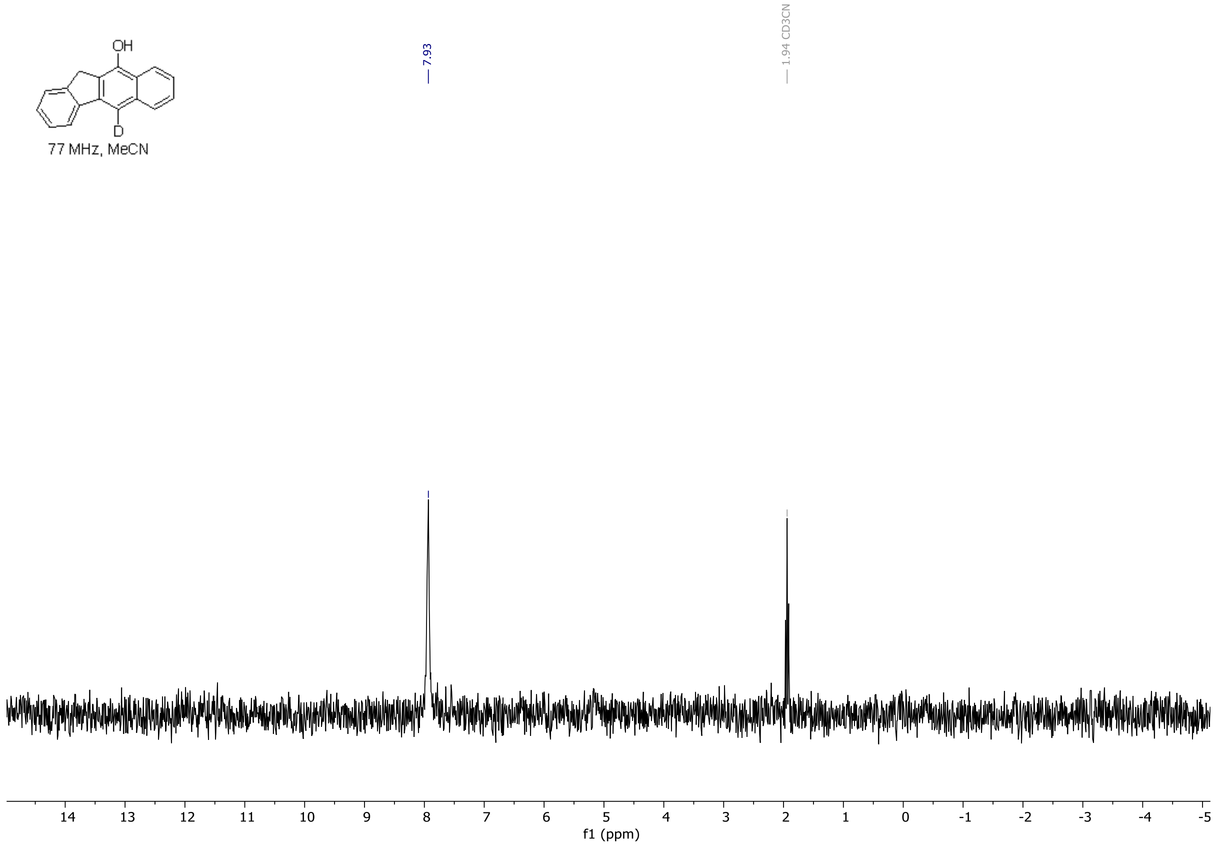
OH
D
77 MHz, MeCN
7.93
1.94 CD3CN
f1 (ppm)

(*E*)-3-(2-Ethynylphenyl)-1-(2-methoxyphenyl)prop-2-en-1-one (4a)

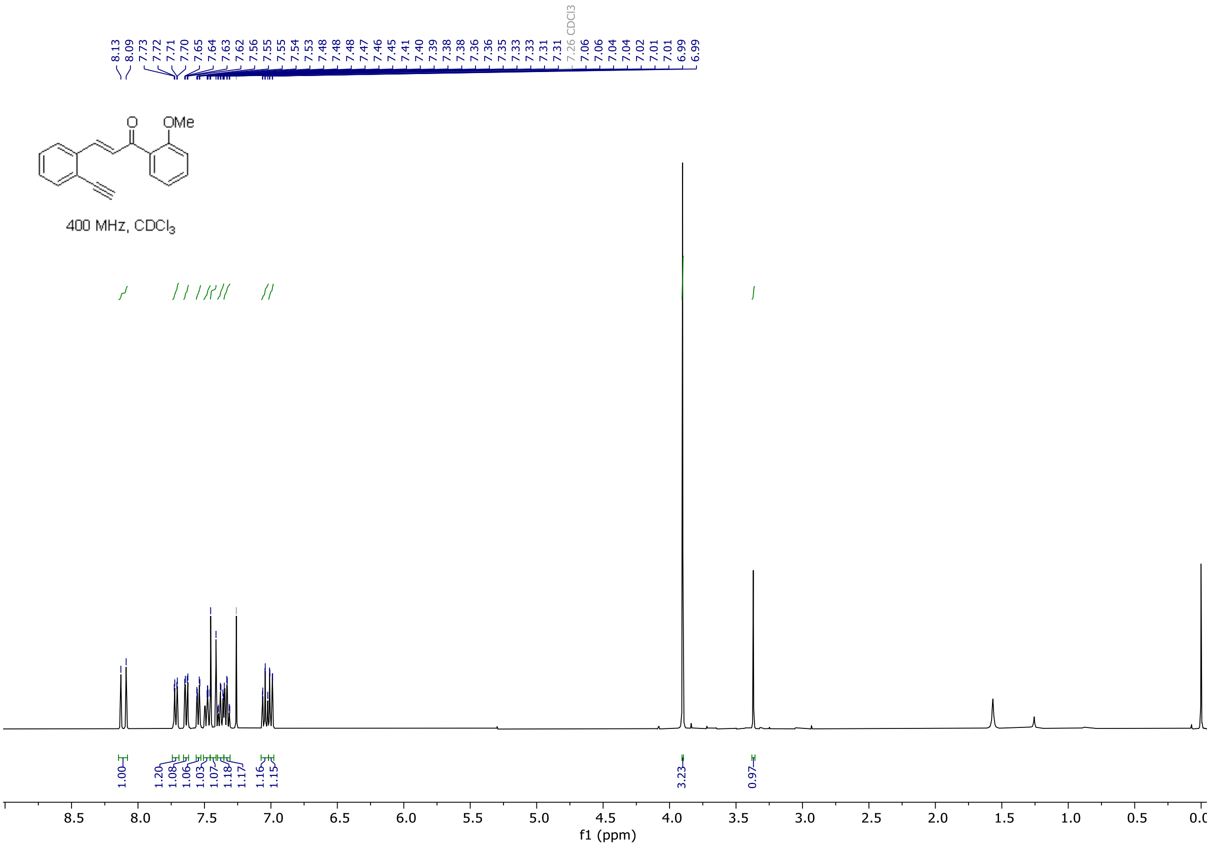

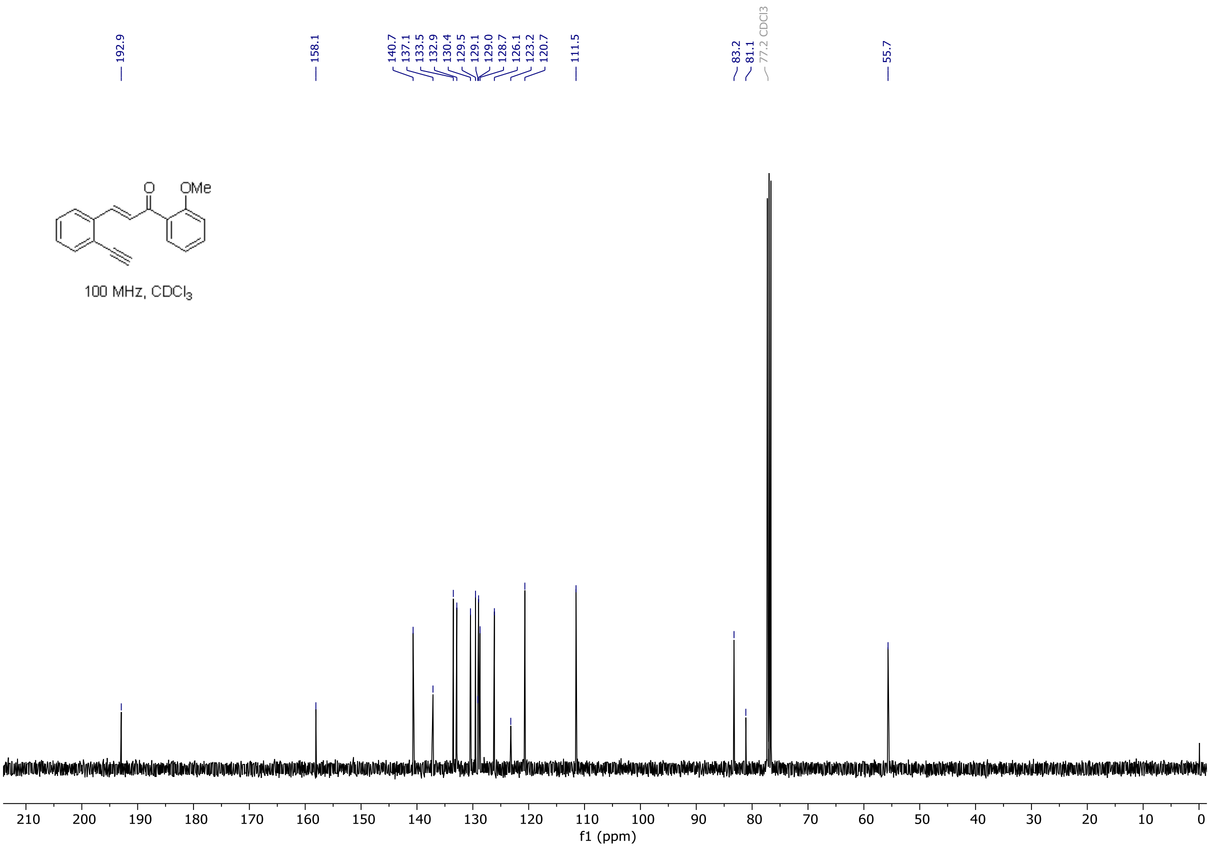

(*E*)-3-(2-Ethynylphenyl)-1-phenylprop-2-en-1-one (4b)

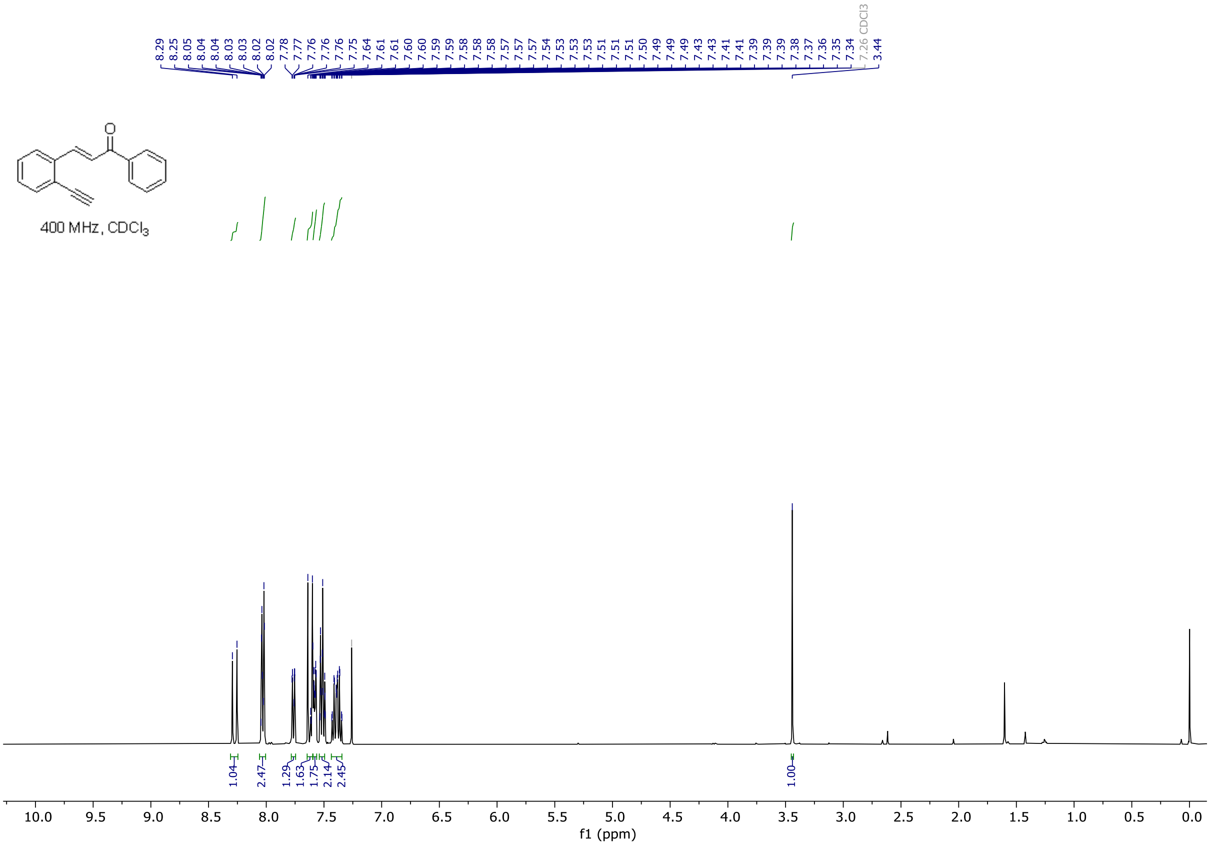

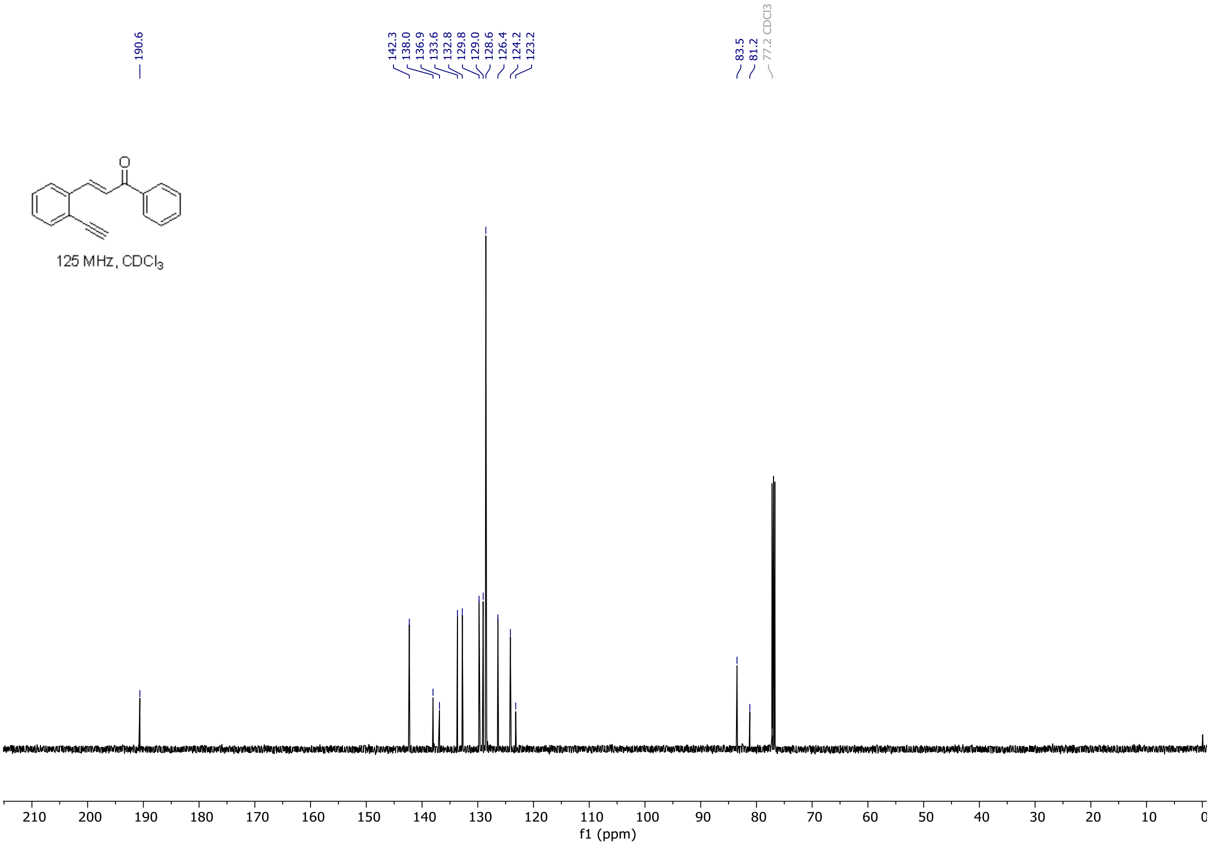

(*E*)-3-(2-Ethynylphenyl)-1-(4-(trifluoromethyl)phenyl)prop-2-en-1-one (4c)

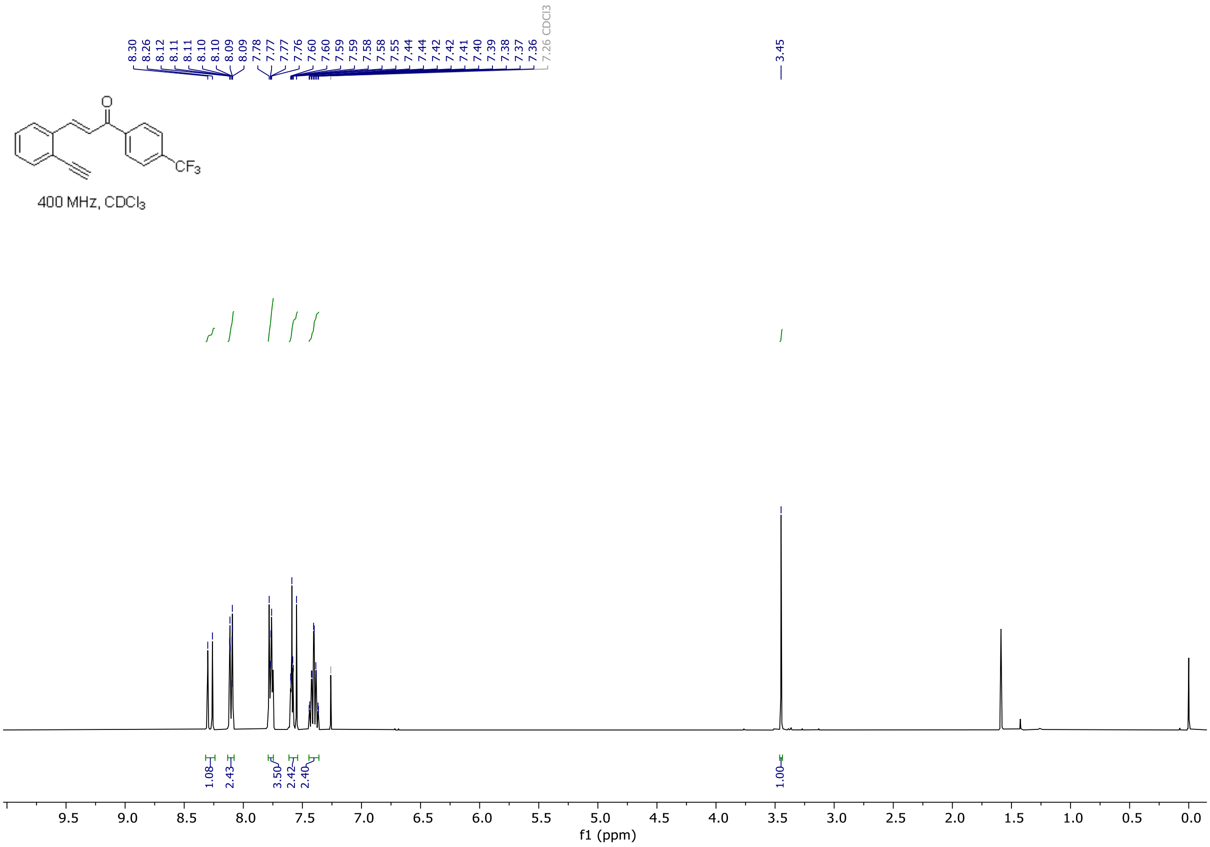

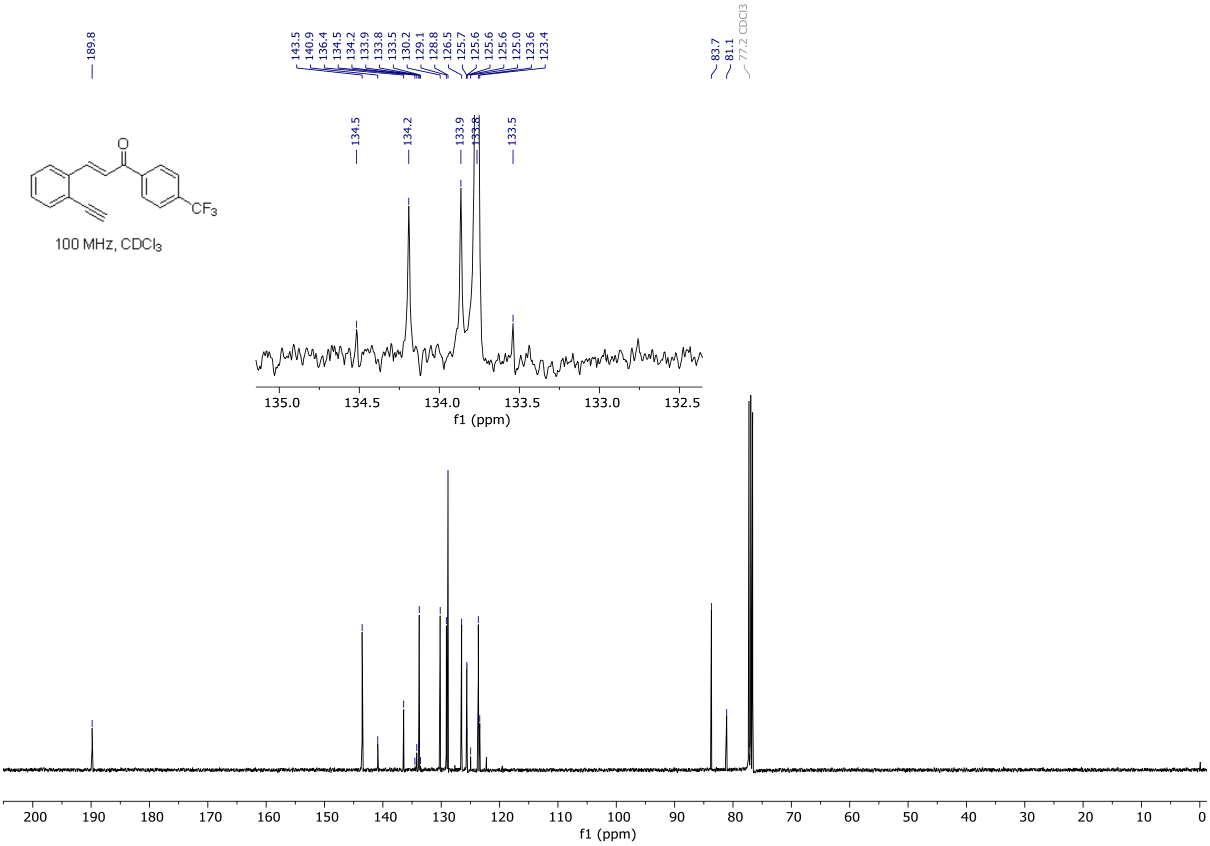

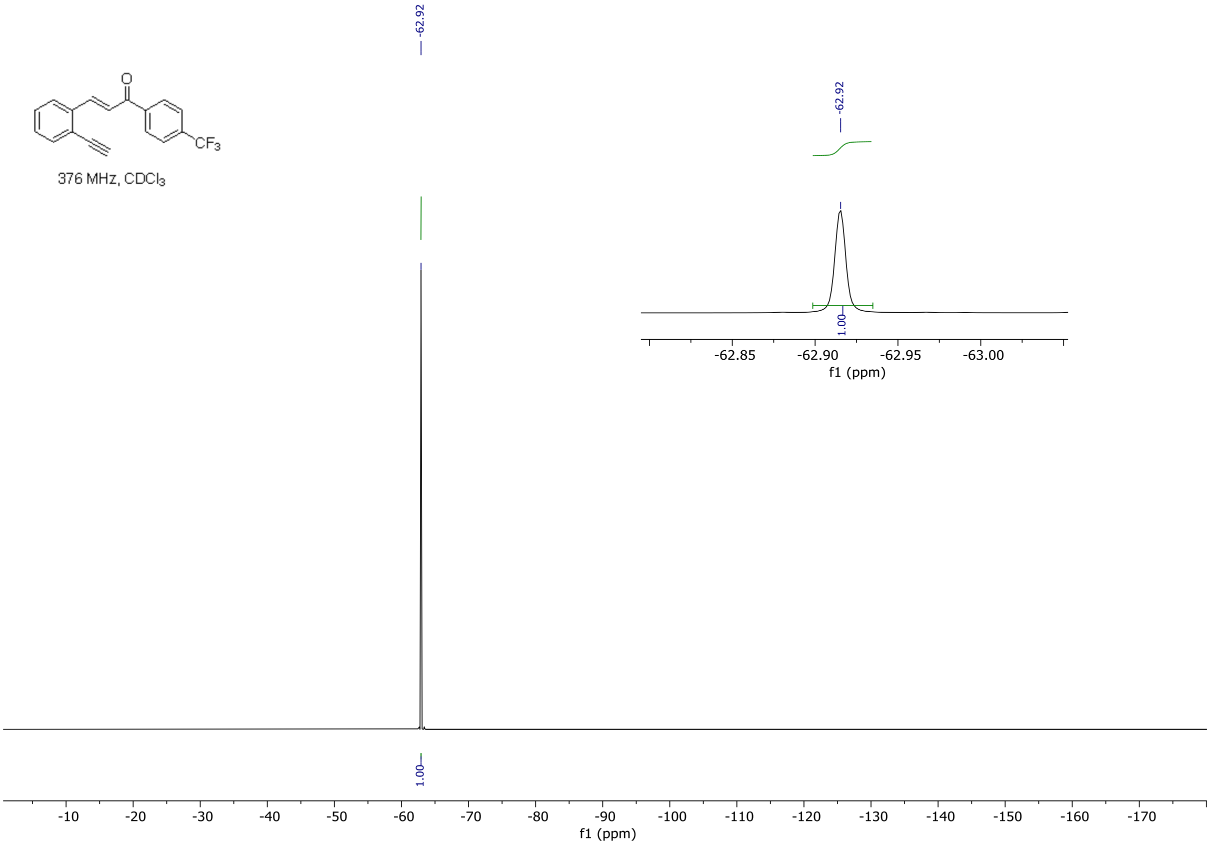

(*E*)-3-(2-Ethynylphenyl)-1-(4-fluorophenyl)prop-2-en-1-one (4d)

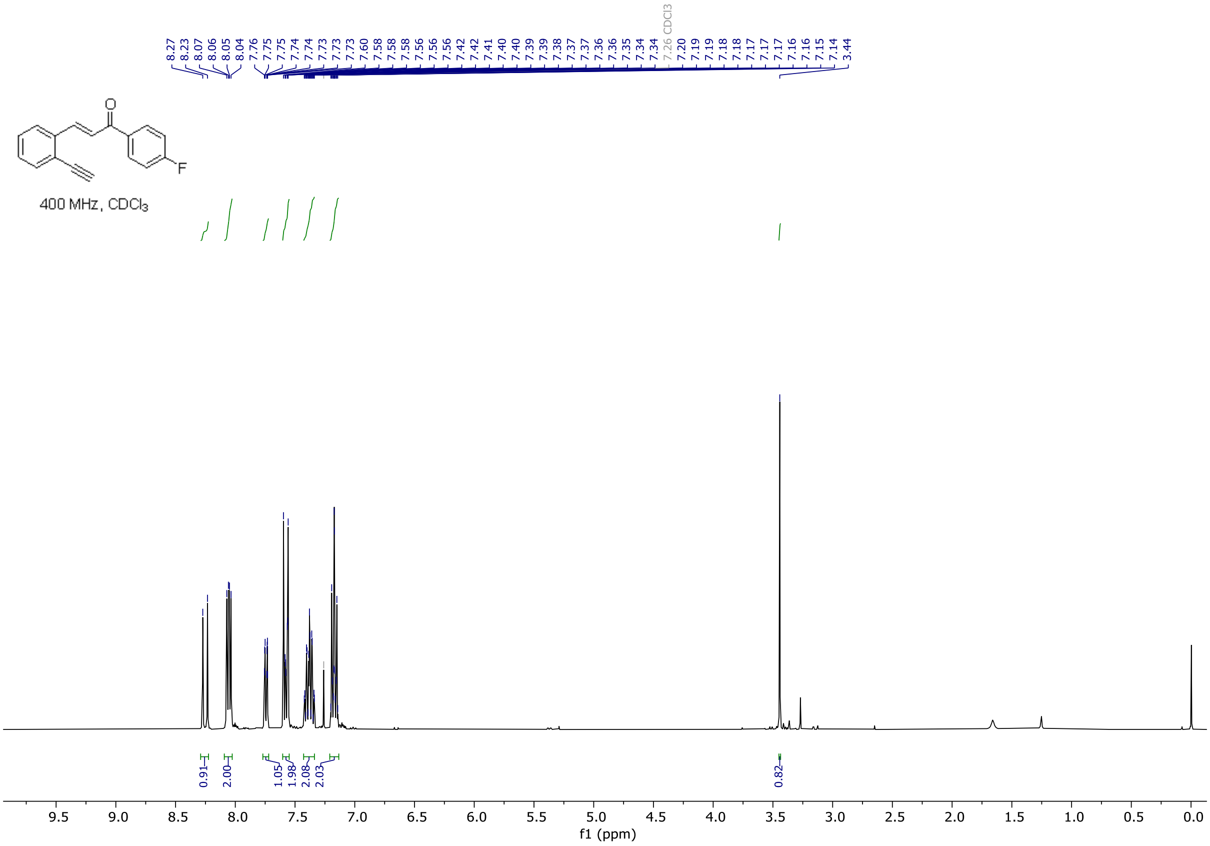

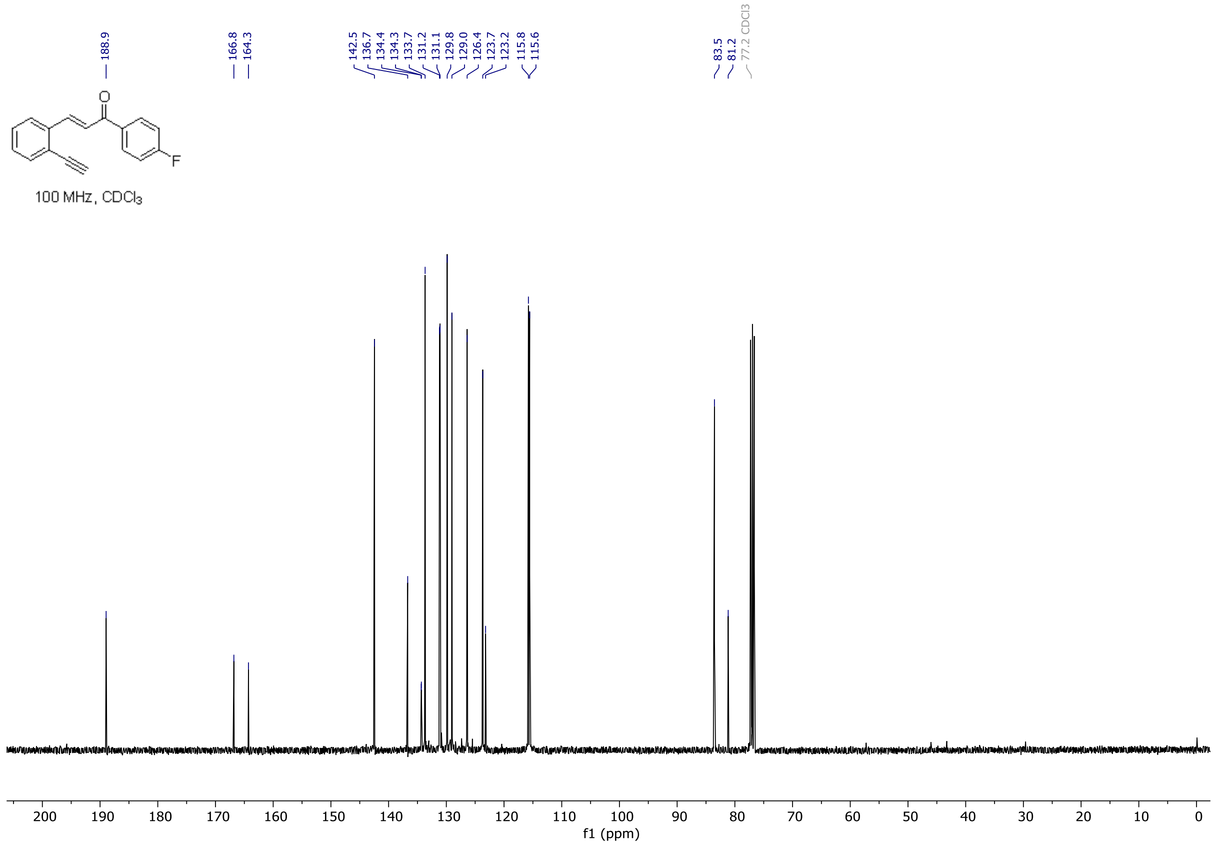
188.9
166.8
164.3
142.5
136.7
134.4
134.3
133.7
131.2
131.1
129.8
129.0
126.4
123.7
123.2
115.8
115.6
83.5
81.2
77.2 CDCl3
F
100 MHz, CDCl3
f1 (ppm)

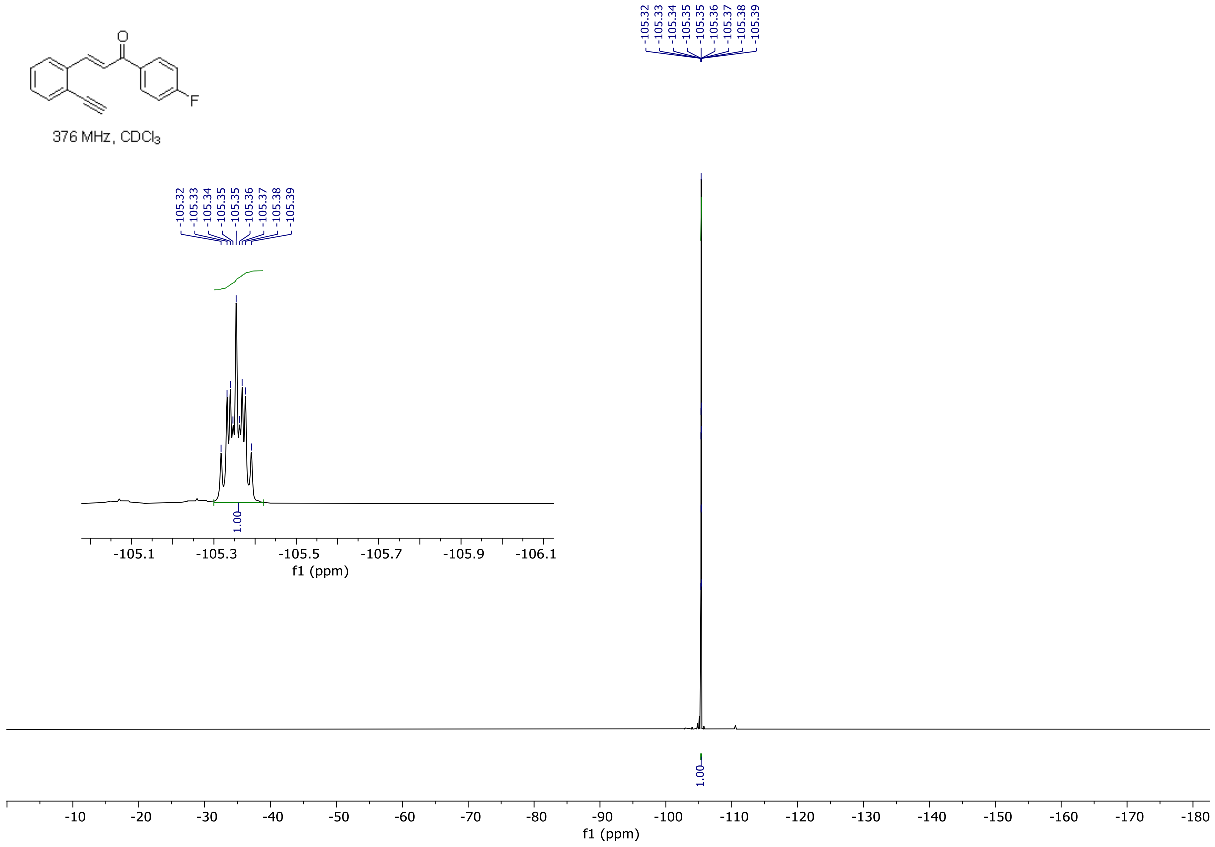
-105.32
-105.33
-105.34
-105.35
-105.35
-105.36
-105.37
-105.38
-105.39
F
376 MHz, CDCl3
1.00
f1 (ppm)

(*E*)-4-(3-(2-Ethynylphenyl)acryloyl)benzonitrile (4e)

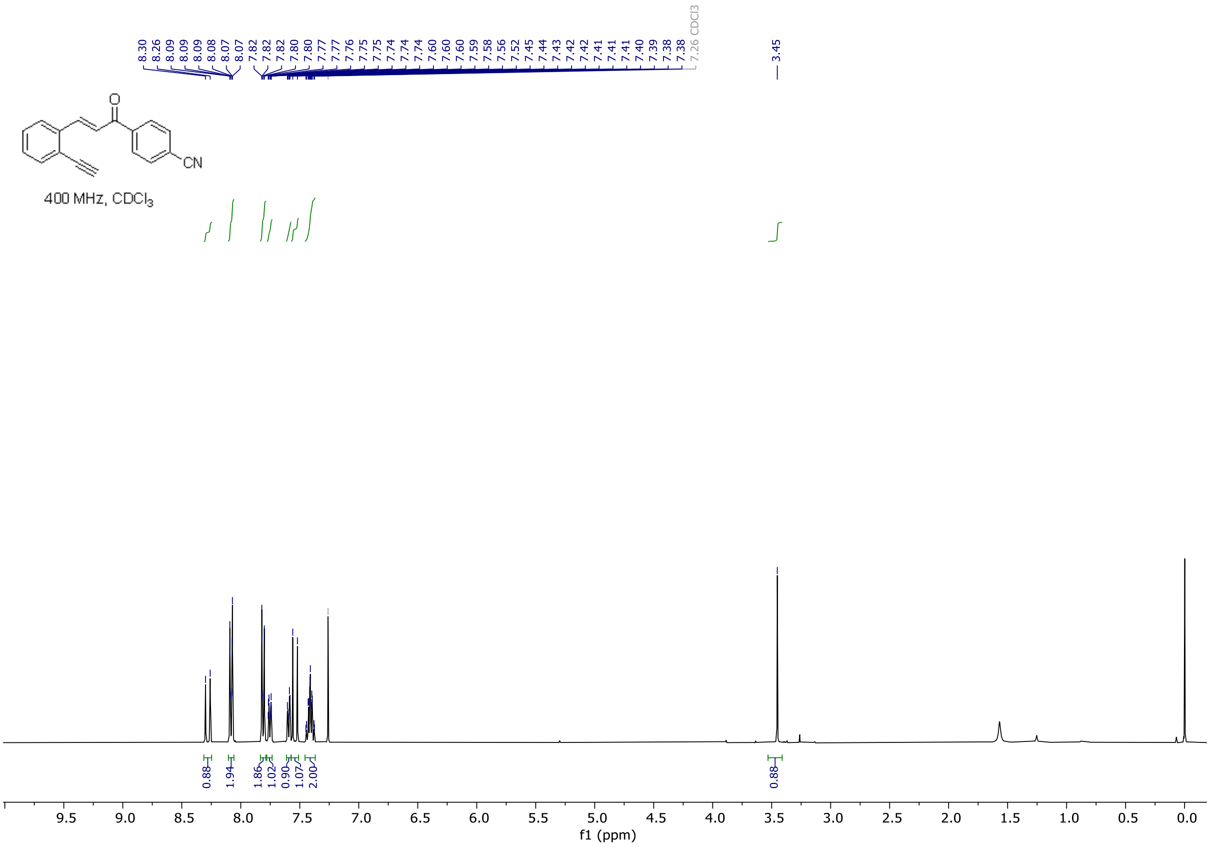

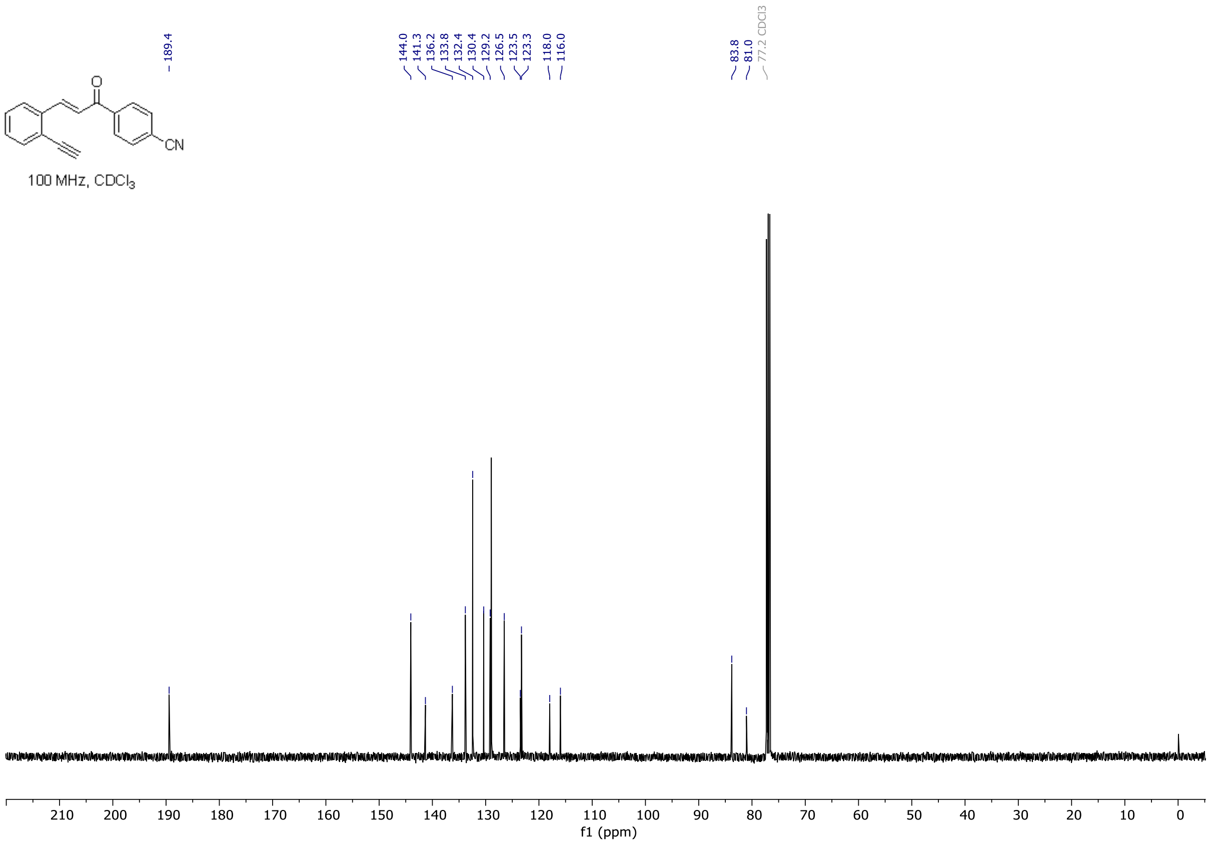

(*E*)-3-(2-Ethynylphenyl)-1-(4-methoxyphenyl)prop-2-en-1-one (4f)

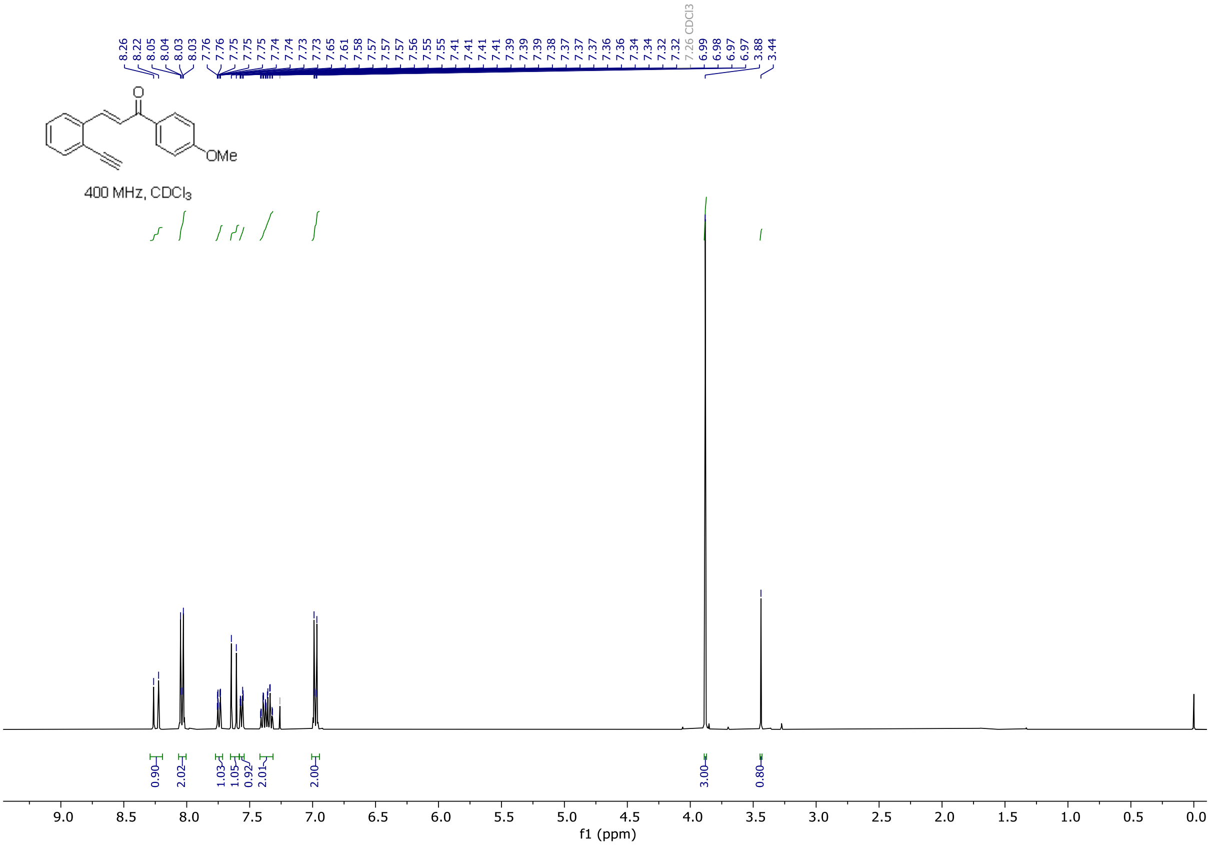

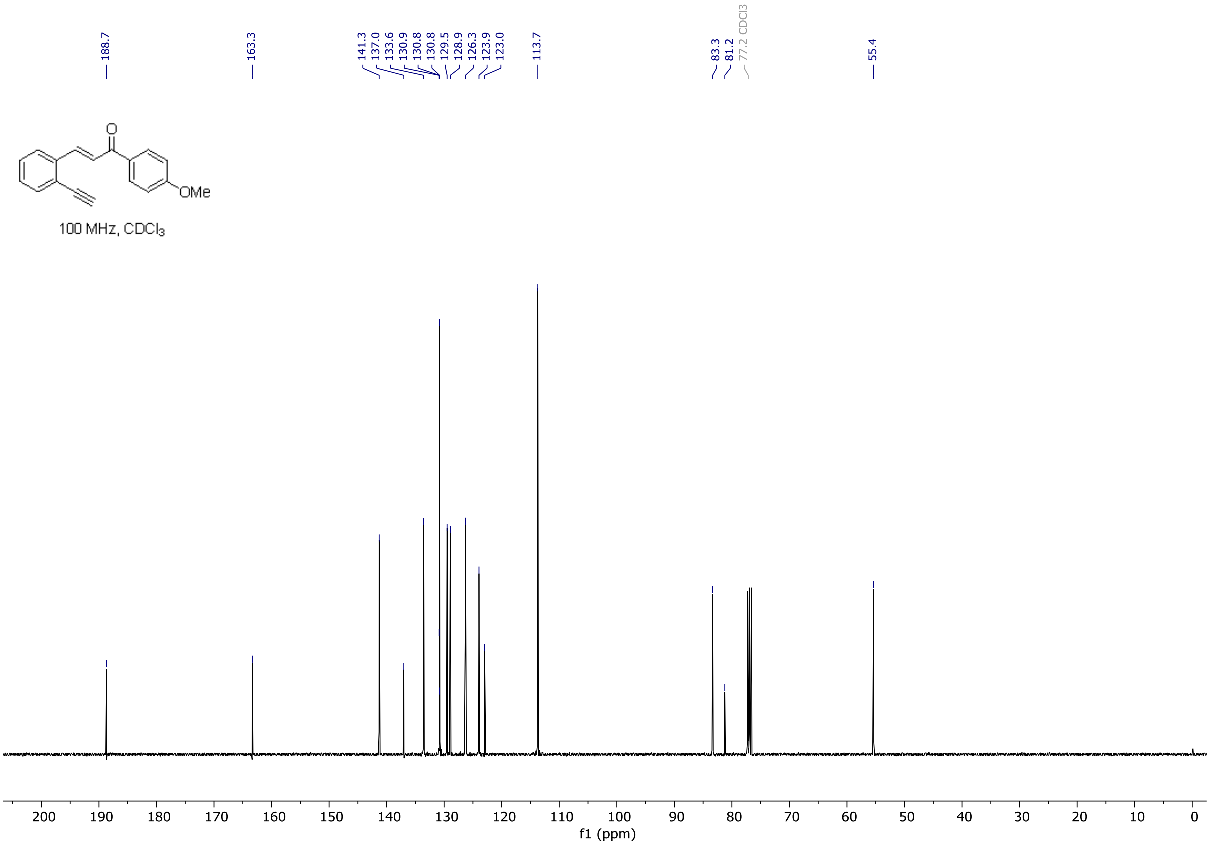

(*E*)-3-(2-Ethynylphenyl)-1-(p-tolyl)prop-2-en-1-one (4g)

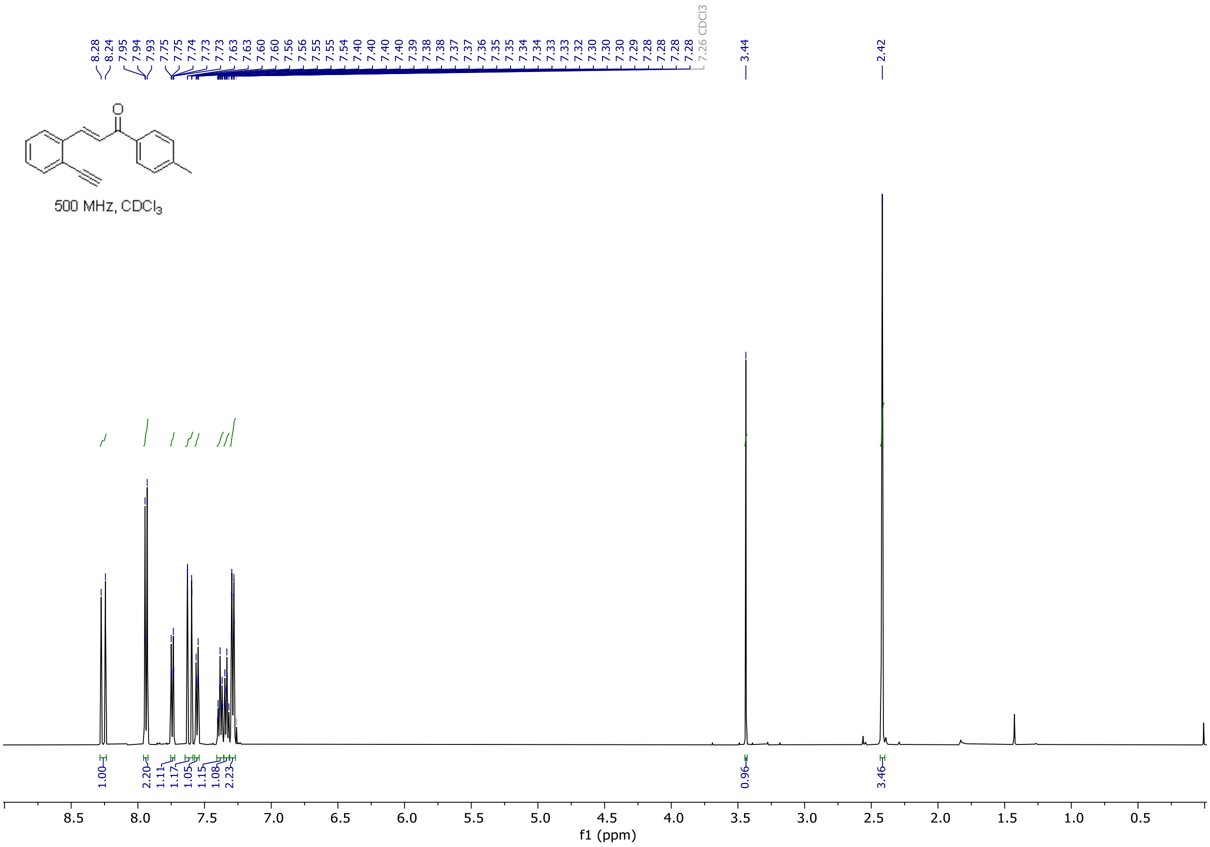

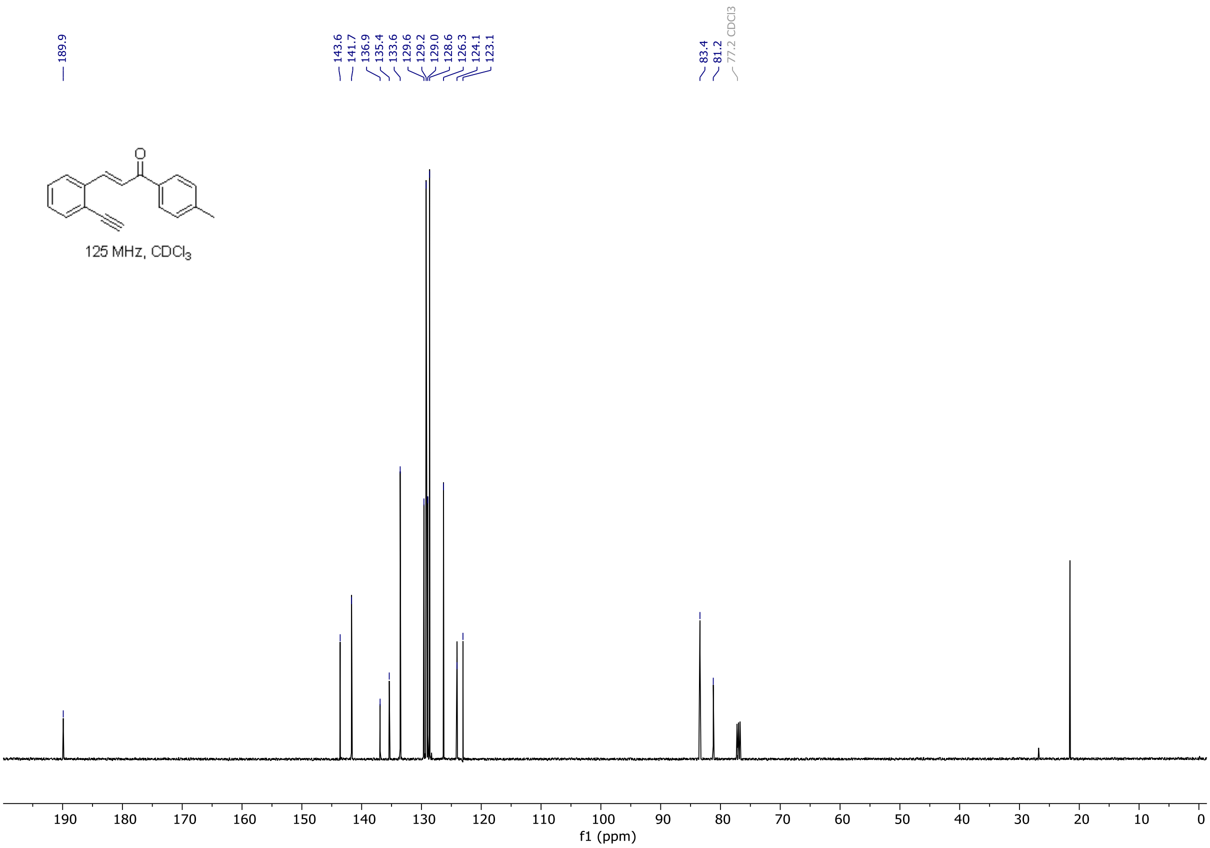

(*E*)-1-(3,4-Dimethylphenyl)-3-(2-ethynylphenyl)prop-2-en-1-one (4h)

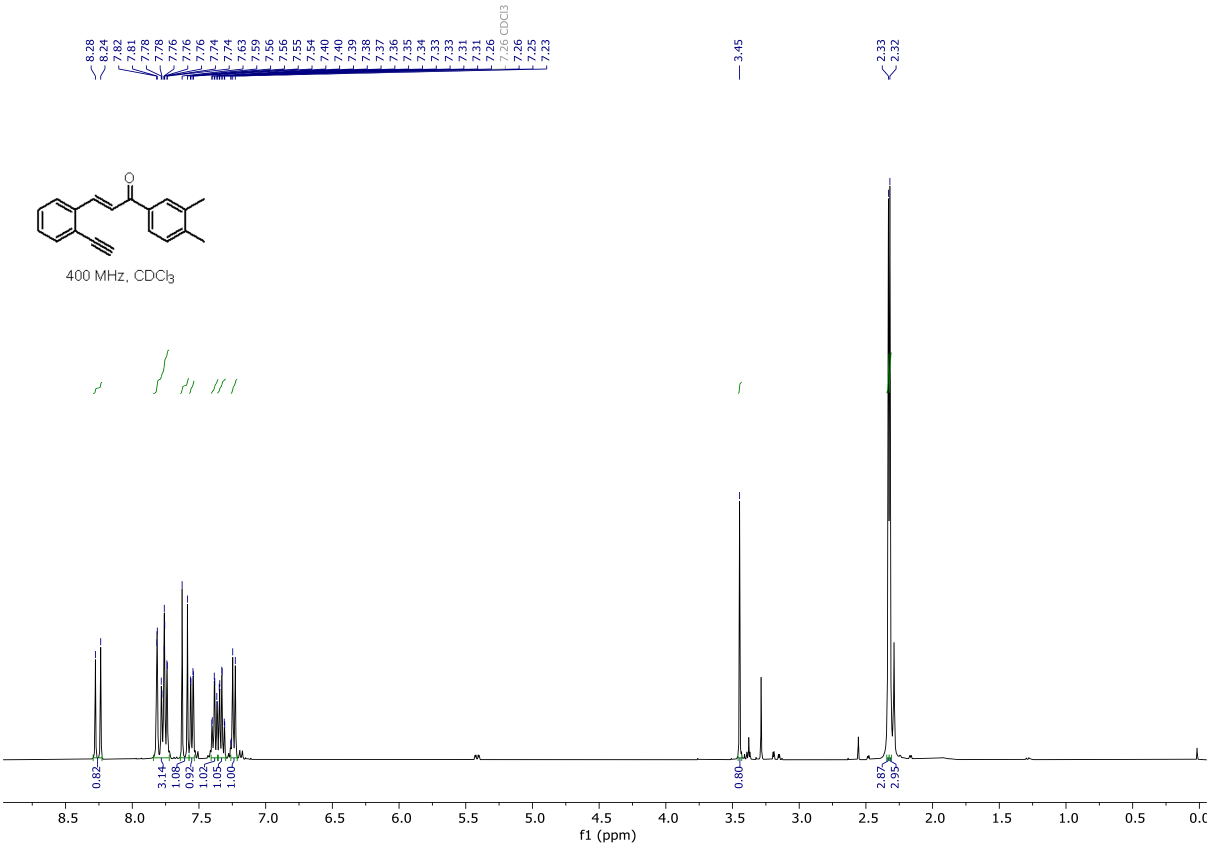

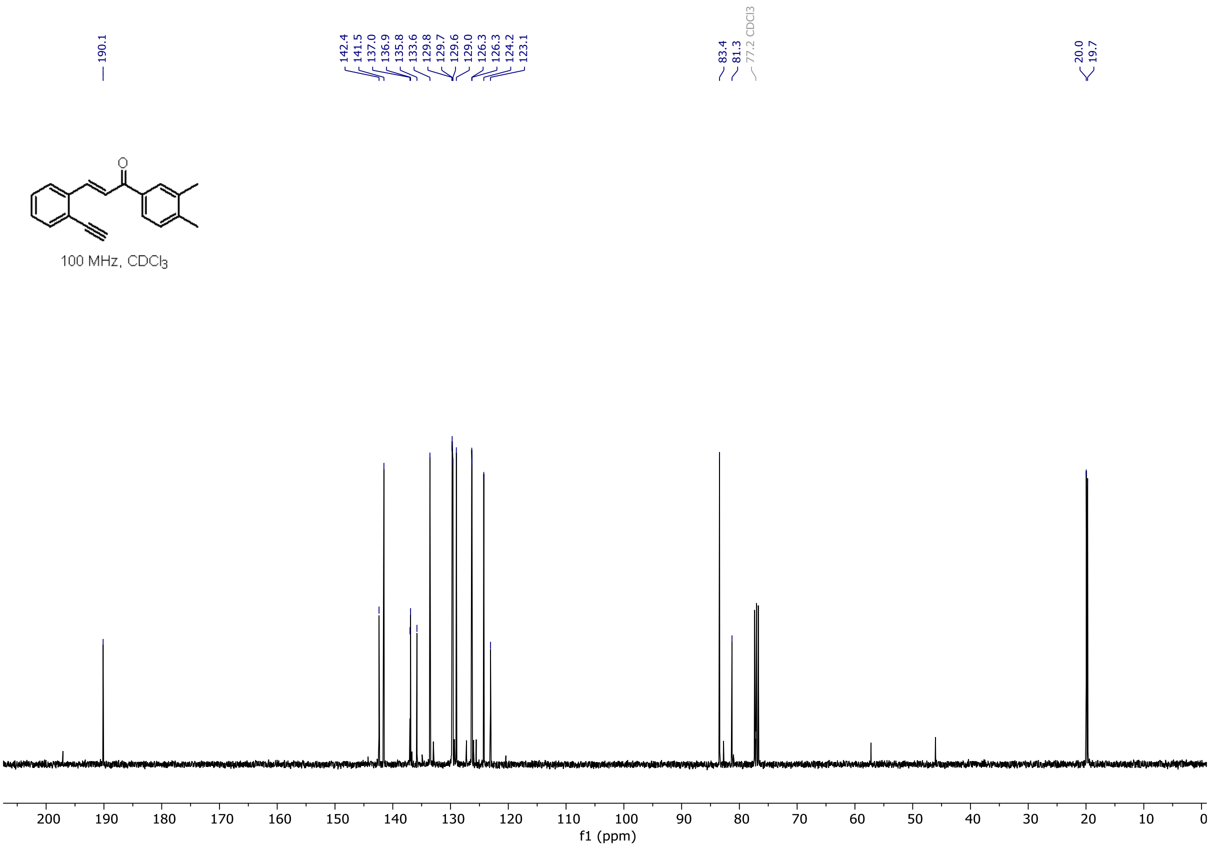

(*E*)-1-(3,5-Difluorophenyl)-3-(2-ethynylphenyl)prop-2-en-1-one (4i)

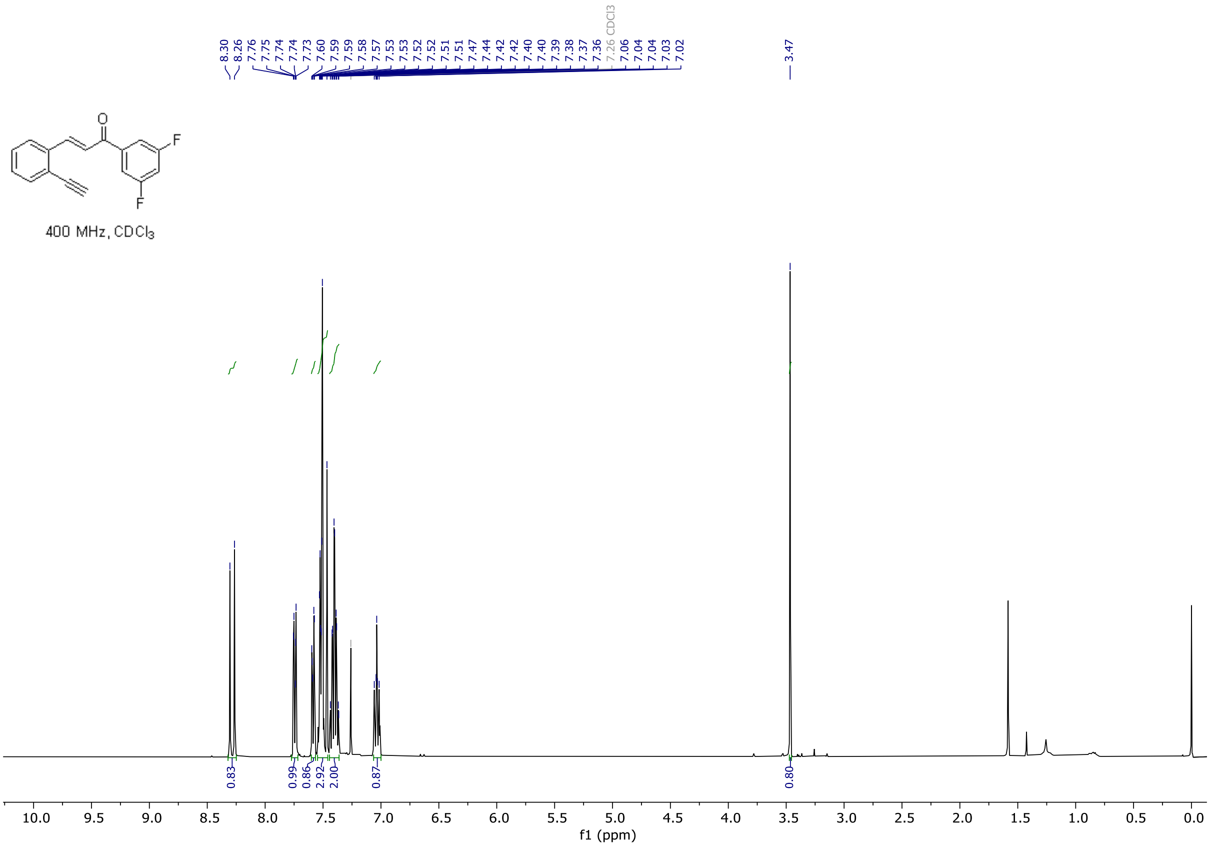

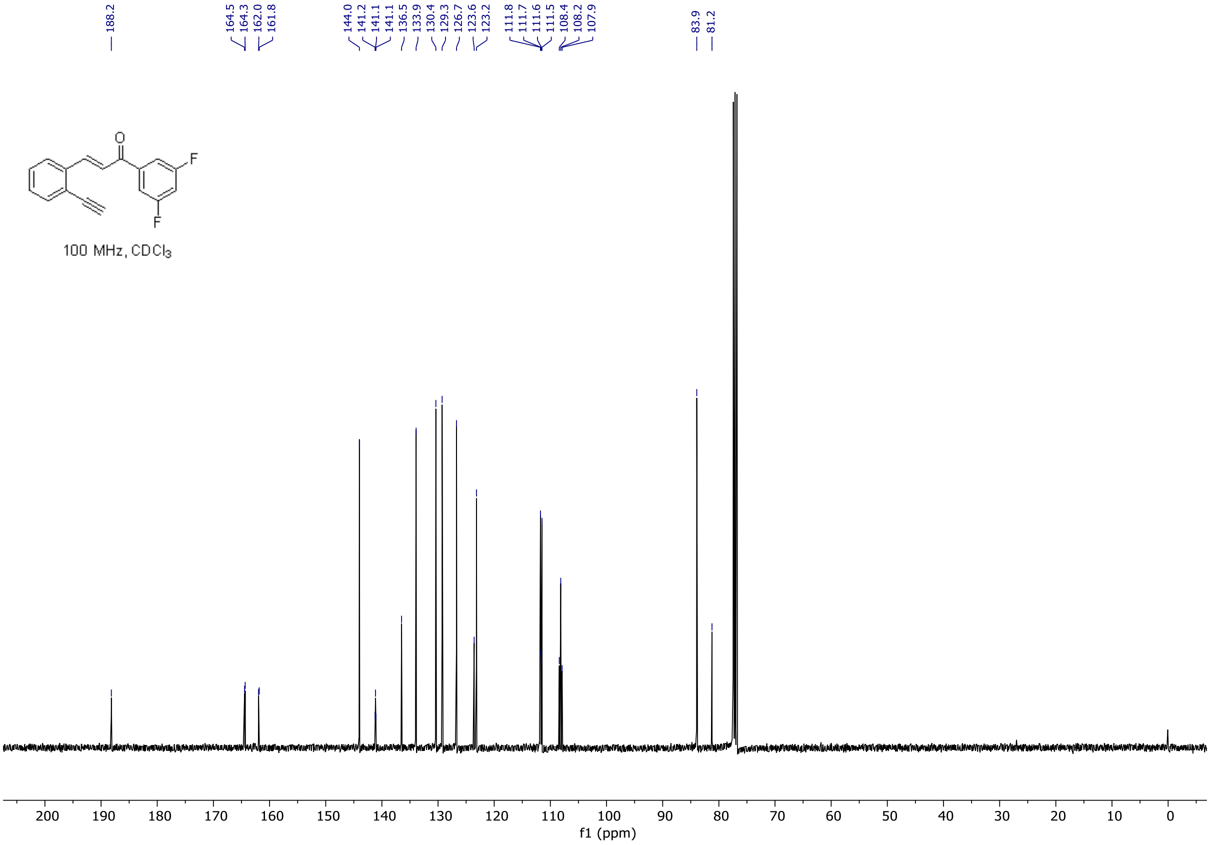

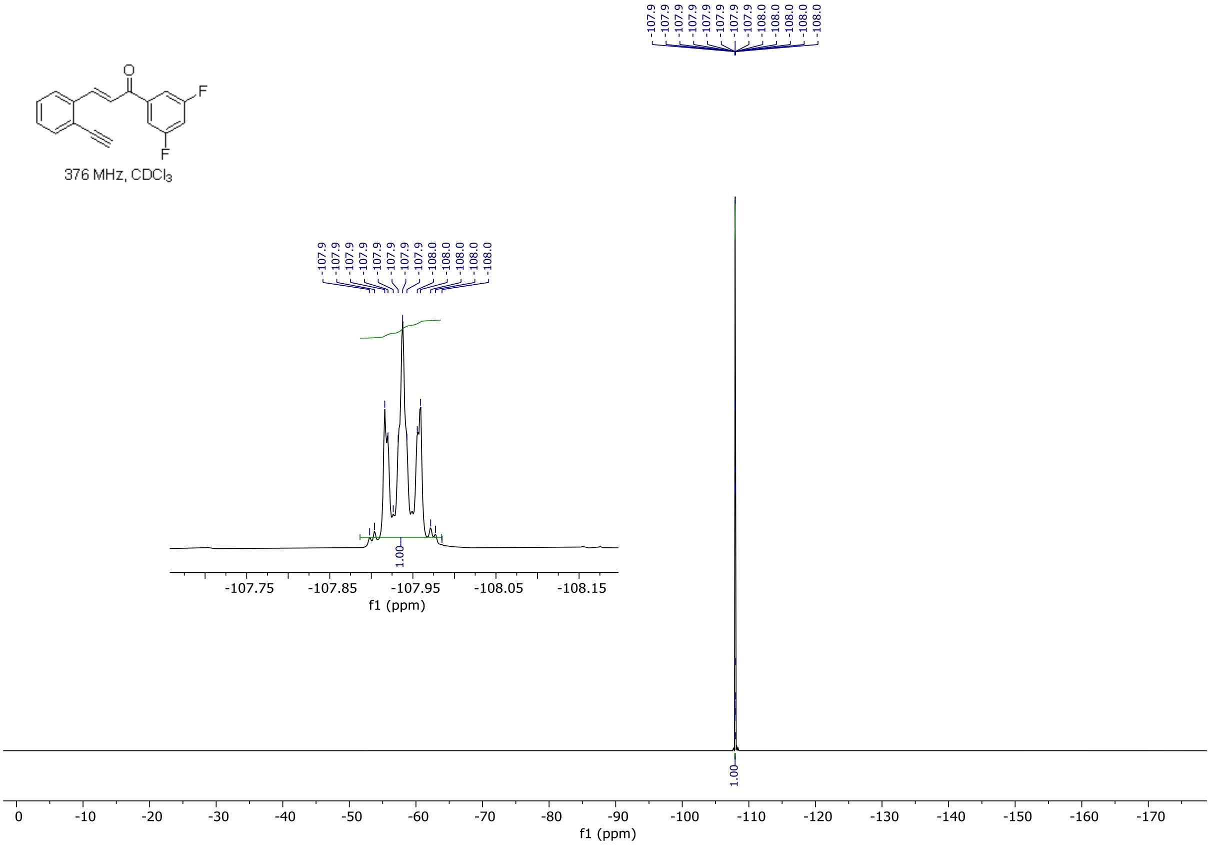

(*E*)-3-(2-Ethynylphenyl)-1-(3-fluorophenyl)prop-2-en-1-one (4j)

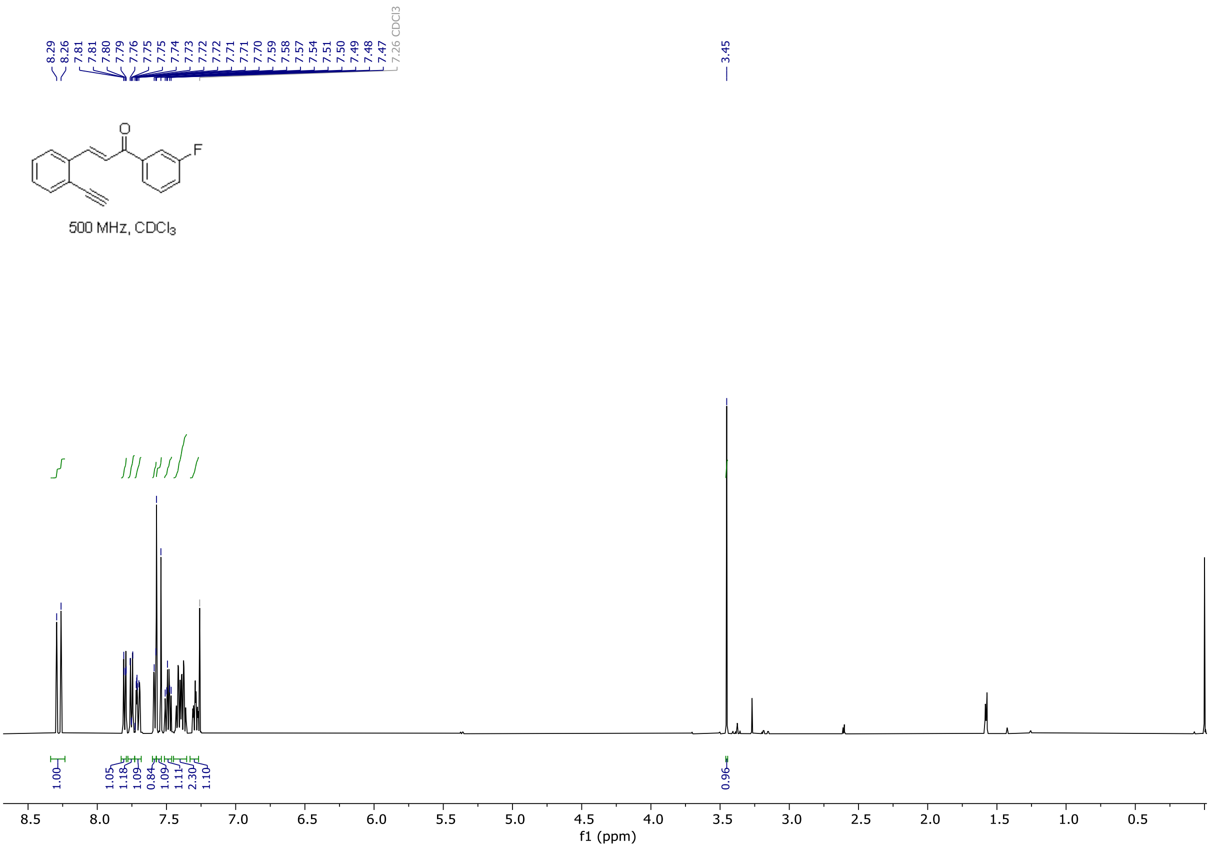

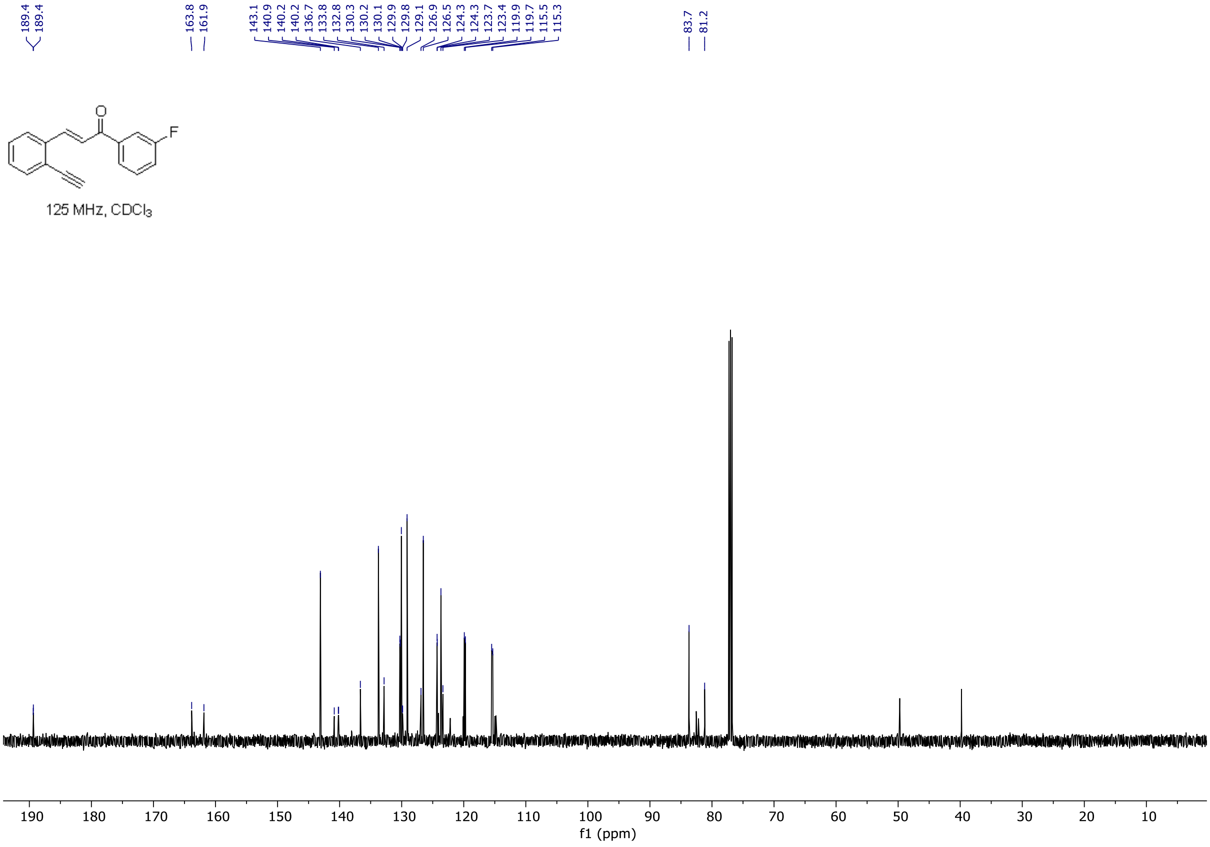
189.4
189.4
163.8
161.9
143.1
140.9
140.2
140.2
136.7
133.8
132.8
130.3
130.2
130.1
129.9
129.8
129.1
126.9
126.5
124.3
124.3
123.7
123.4
119.9
119.7
115.5
115.3
83.7
81.2
O
F
125 MHz, CDCl3
f1 (ppm)

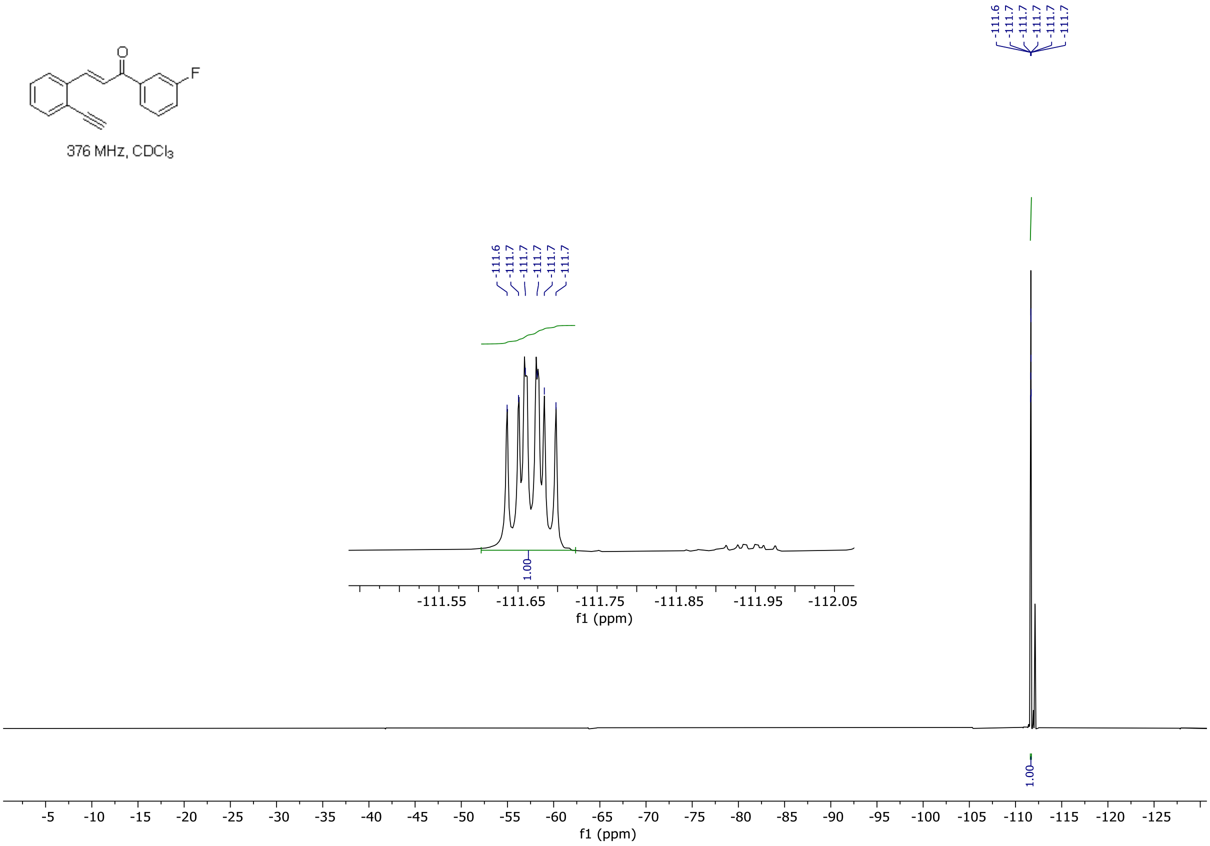
-111.6
-111.7
-111.7
-111.7
-111.7
-111.7
-111.7
O
F
376 MHz, CDCl3
1.00
f1 (ppm)
f1 (ppm)

(E)-3-(2-Ethynylphenyl)-1-(2-fluorophenyl)prop-2-en-1-one (4k)

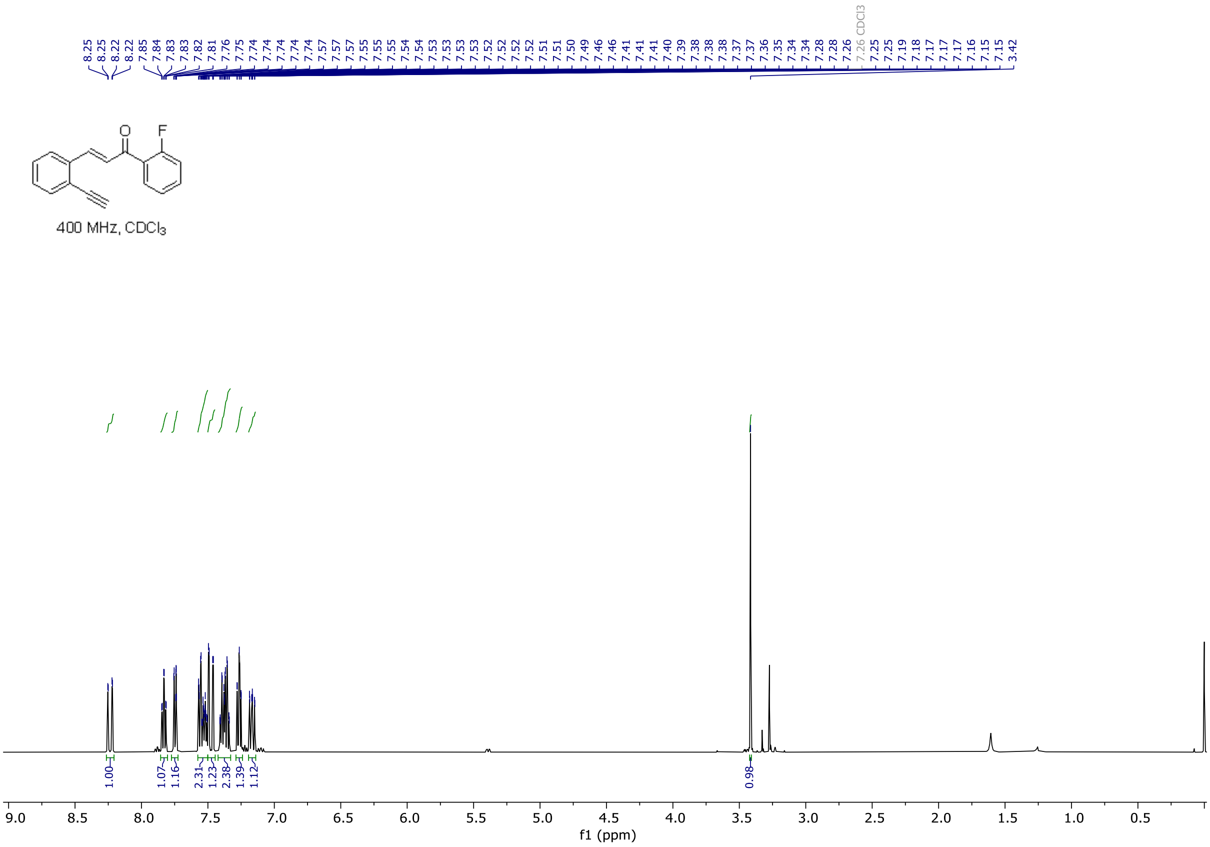

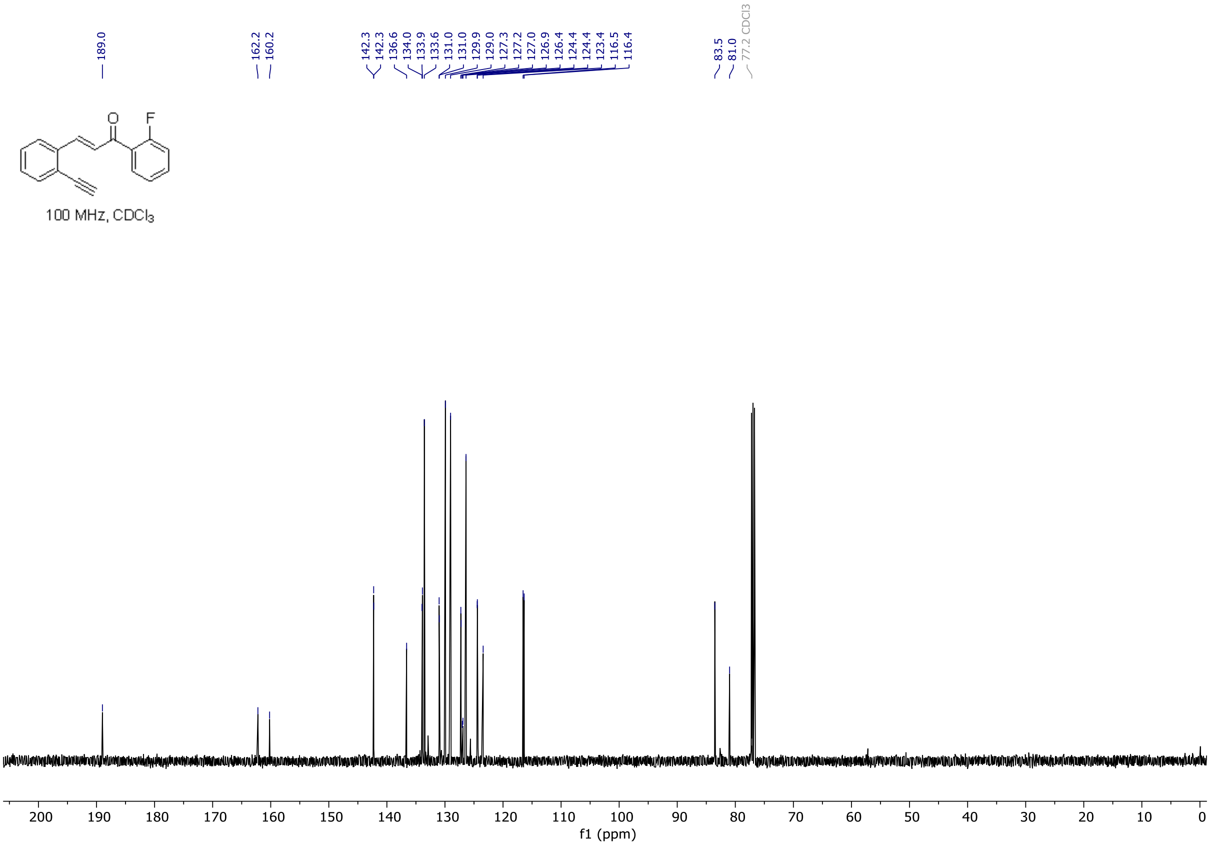

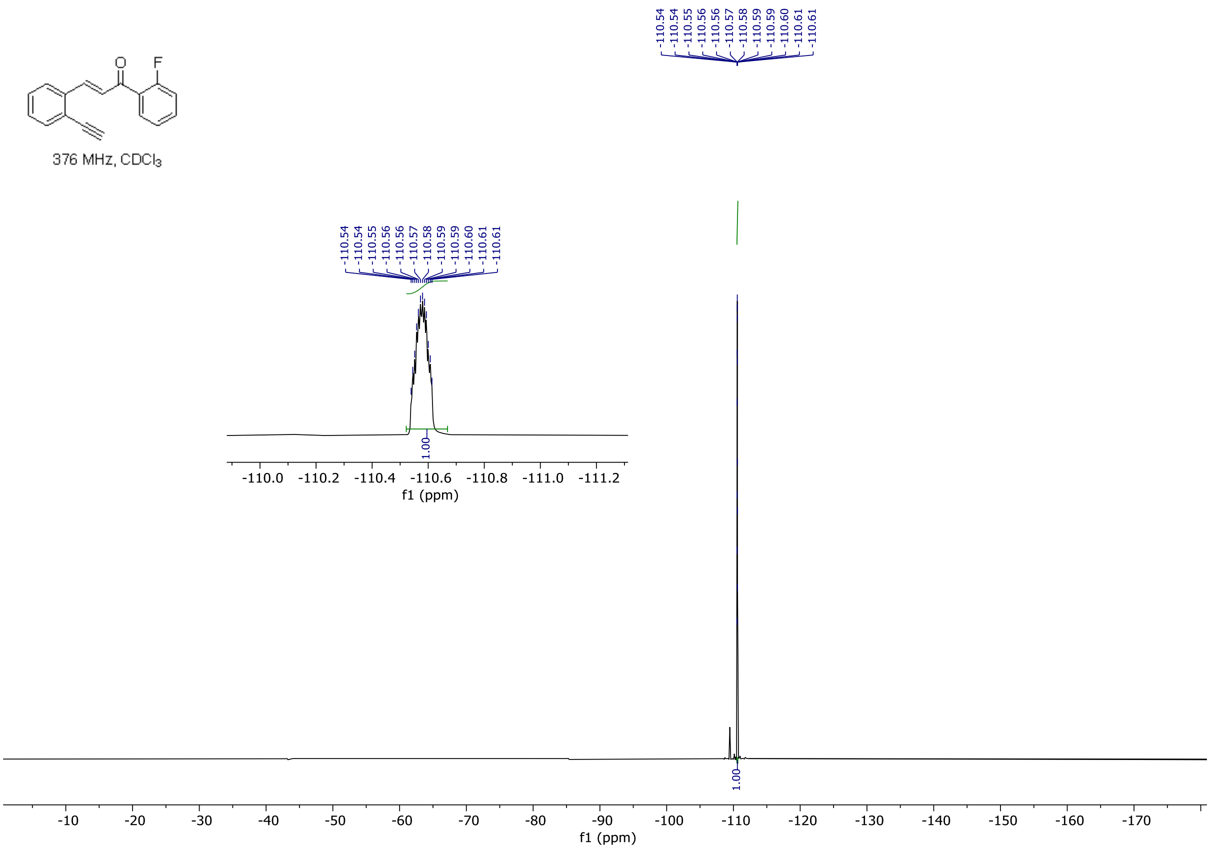

(*E*)-3-(2-Ethynylphenyl)-1-(o-tolyl)prop-2-en-1-one (4l)

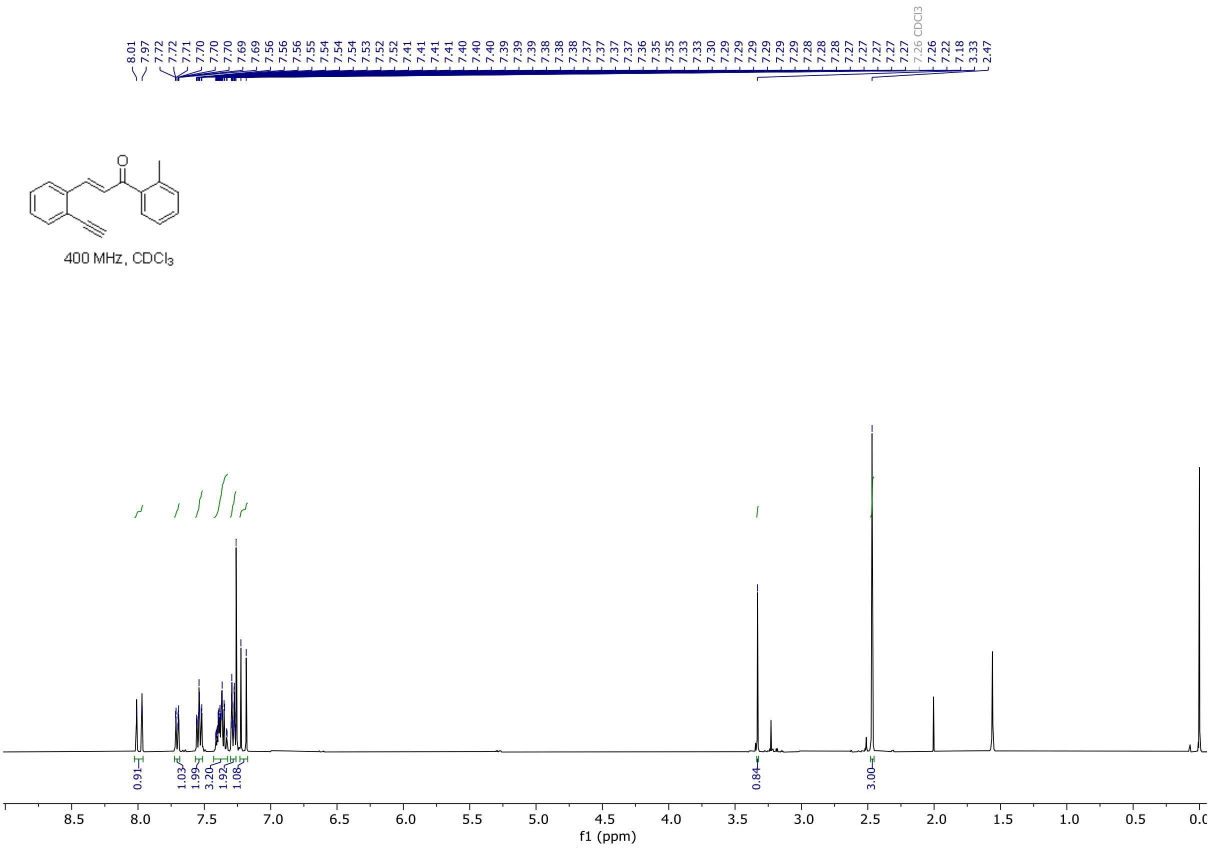

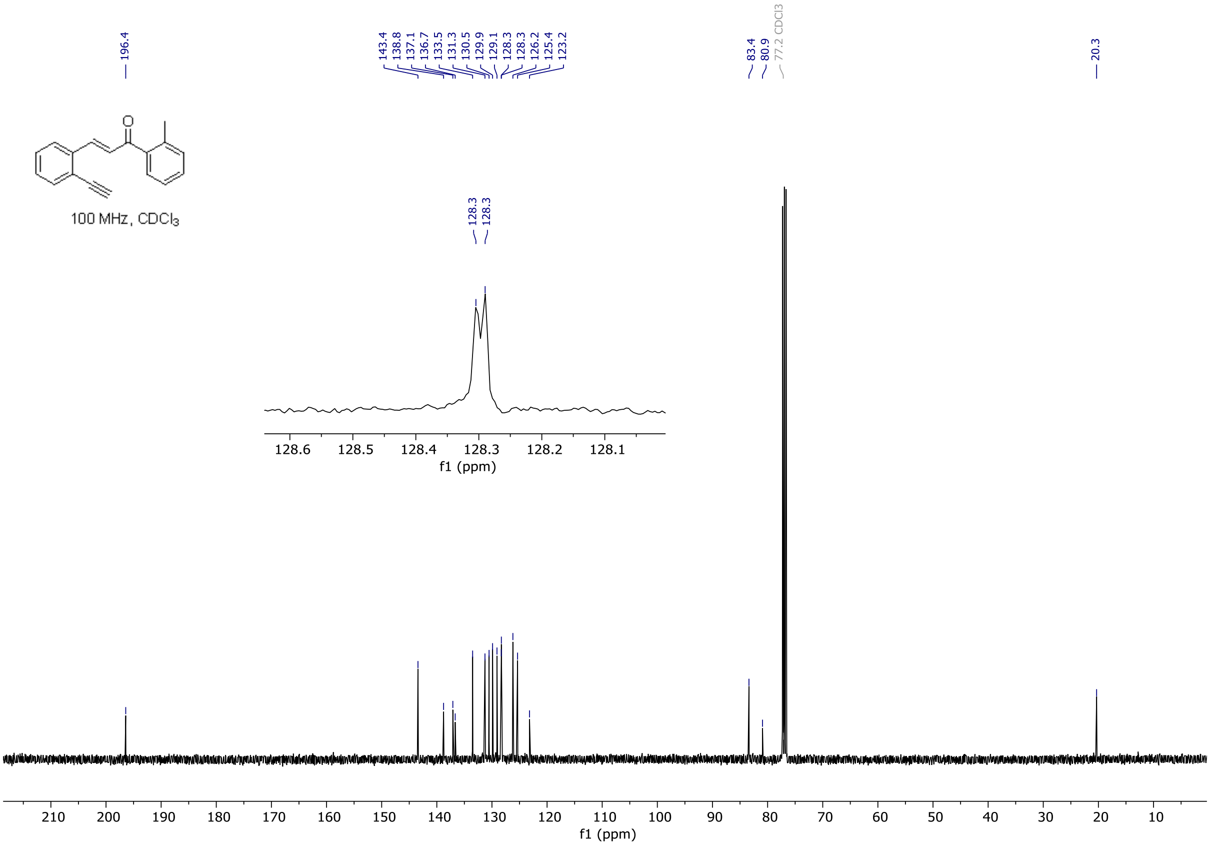

(*E*)-3-(2-Ethynylphenyl)-1-(2-fluoro-4-methoxyphenyl)prop-2-en-1-one (4m)

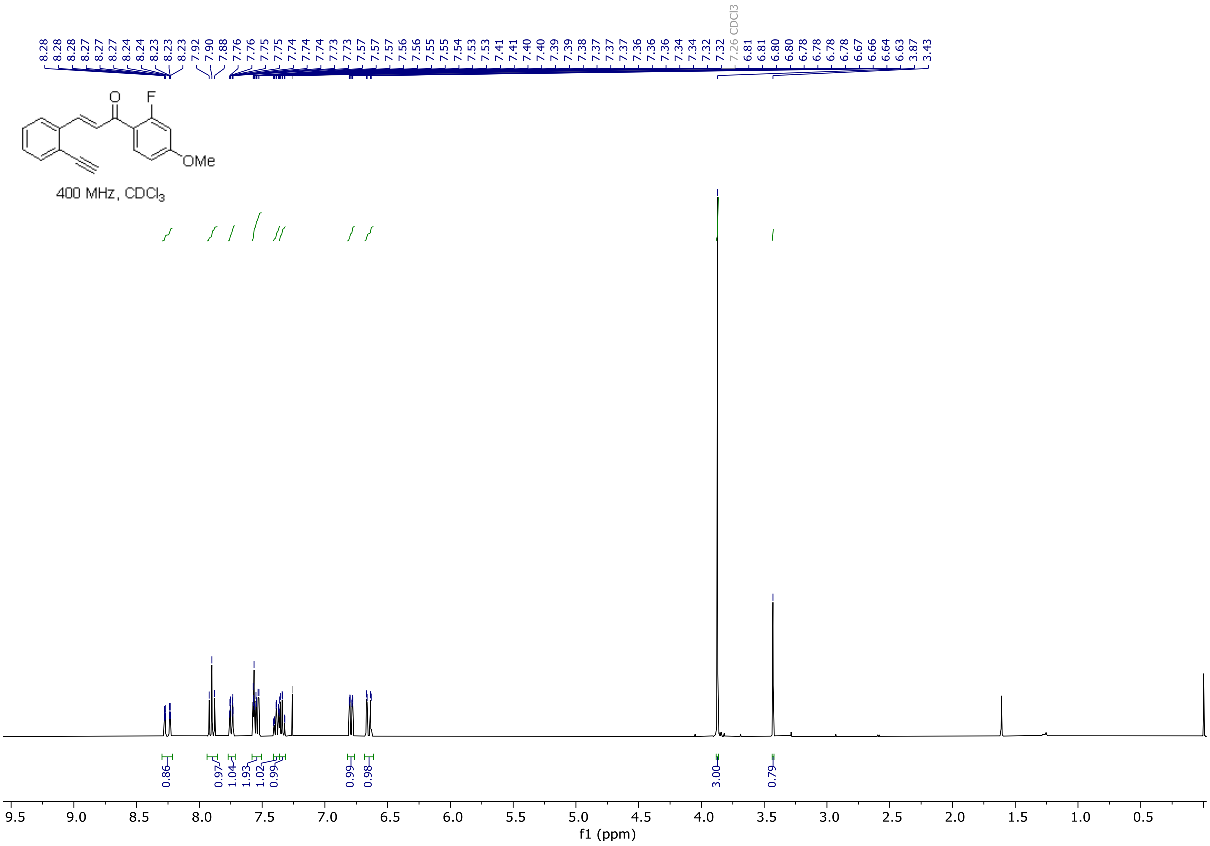

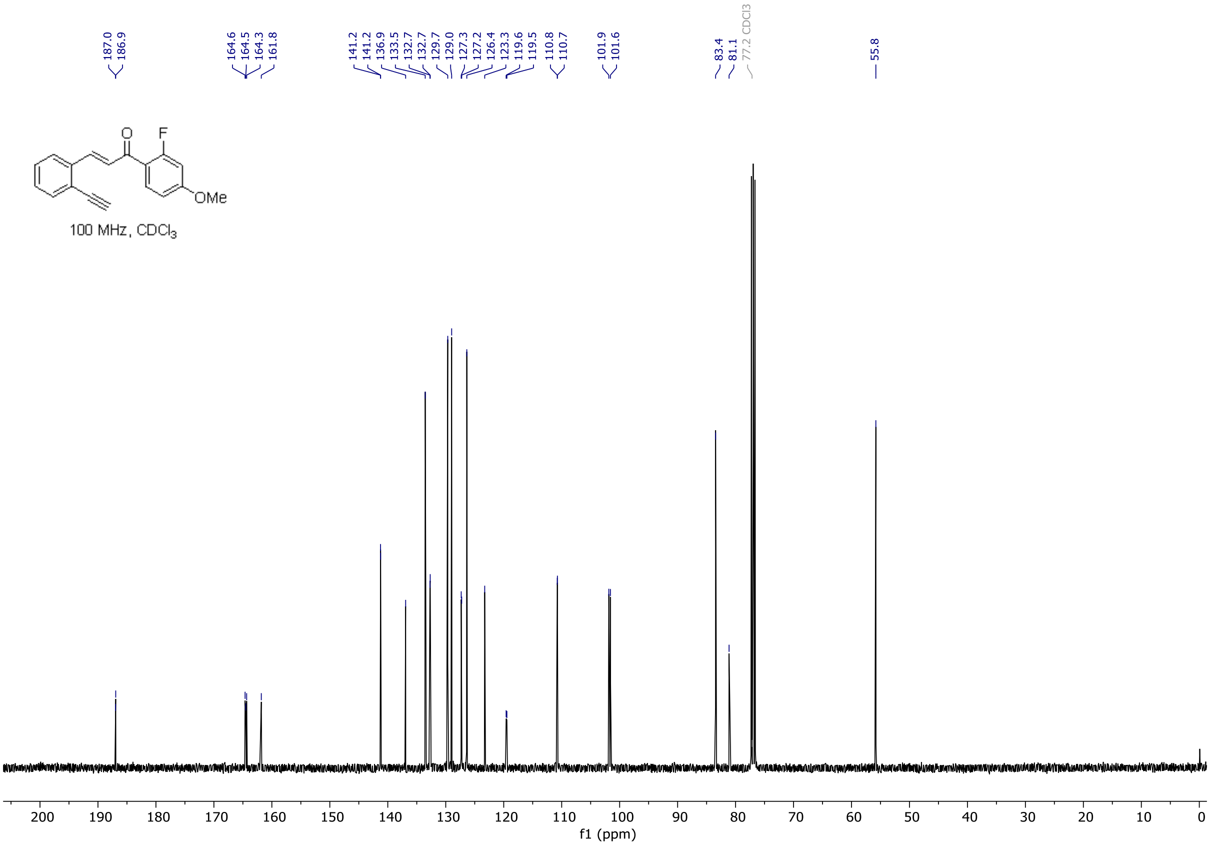
187.0
186.9
164.6
164.5
164.3
161.8
141.2
141.2
136.9
133.5
132.7
132.7
129.7
129.0
127.3
127.2
126.4
123.3
119.6
119.5
110.8
110.7
101.9
101.6
83.4
81.1
77.2 CDCl3
55.8
O
F
OMe
100 MHz, CDCl3
f1 (ppm)

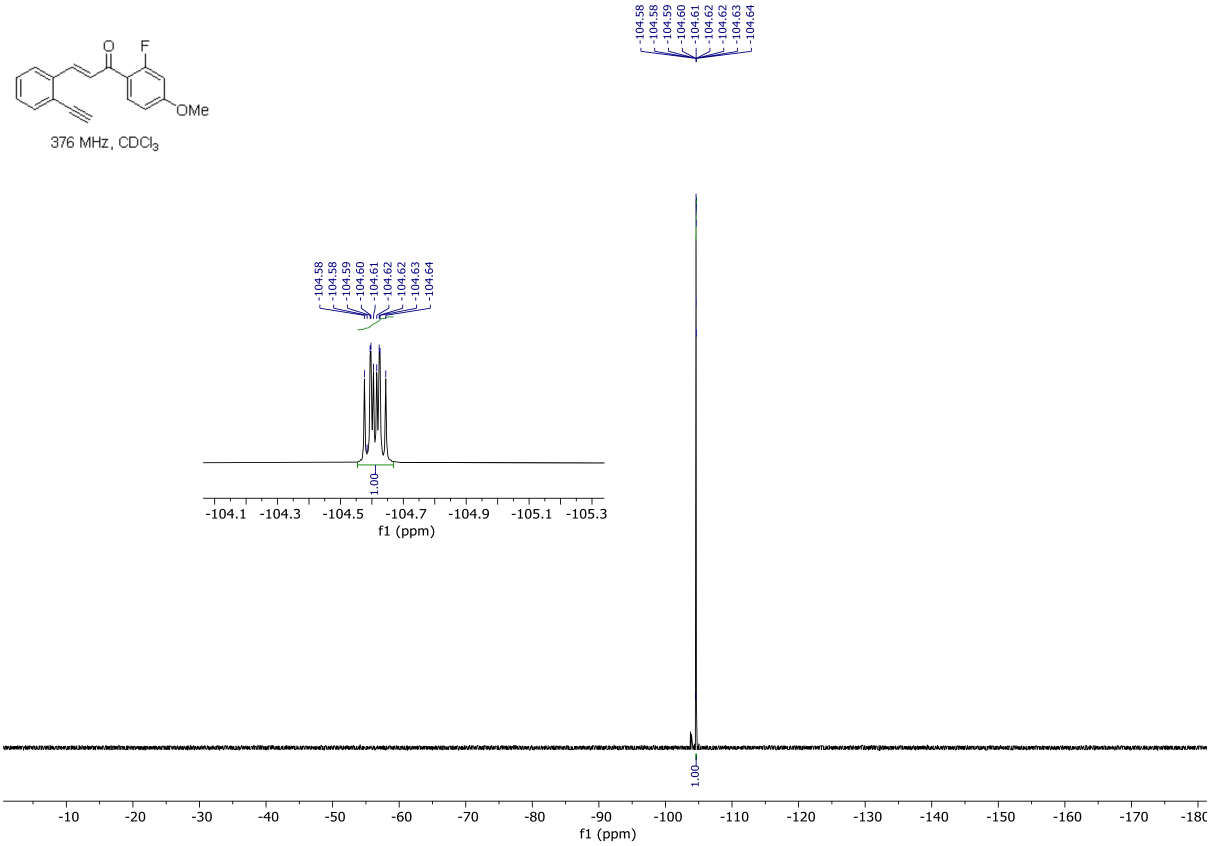
-104.58
-104.58
-104.59
-104.60
-104.61
-104.62
-104.62
-104.63
-104.64
O
F
OMe
376 MHz, CDCl3
1.00
f1 (ppm)

(*E*)-3-(2-Ethynyl-4-methylphenyl)-1-(4-fluorophenyl)prop-2-en-1-one (4n)

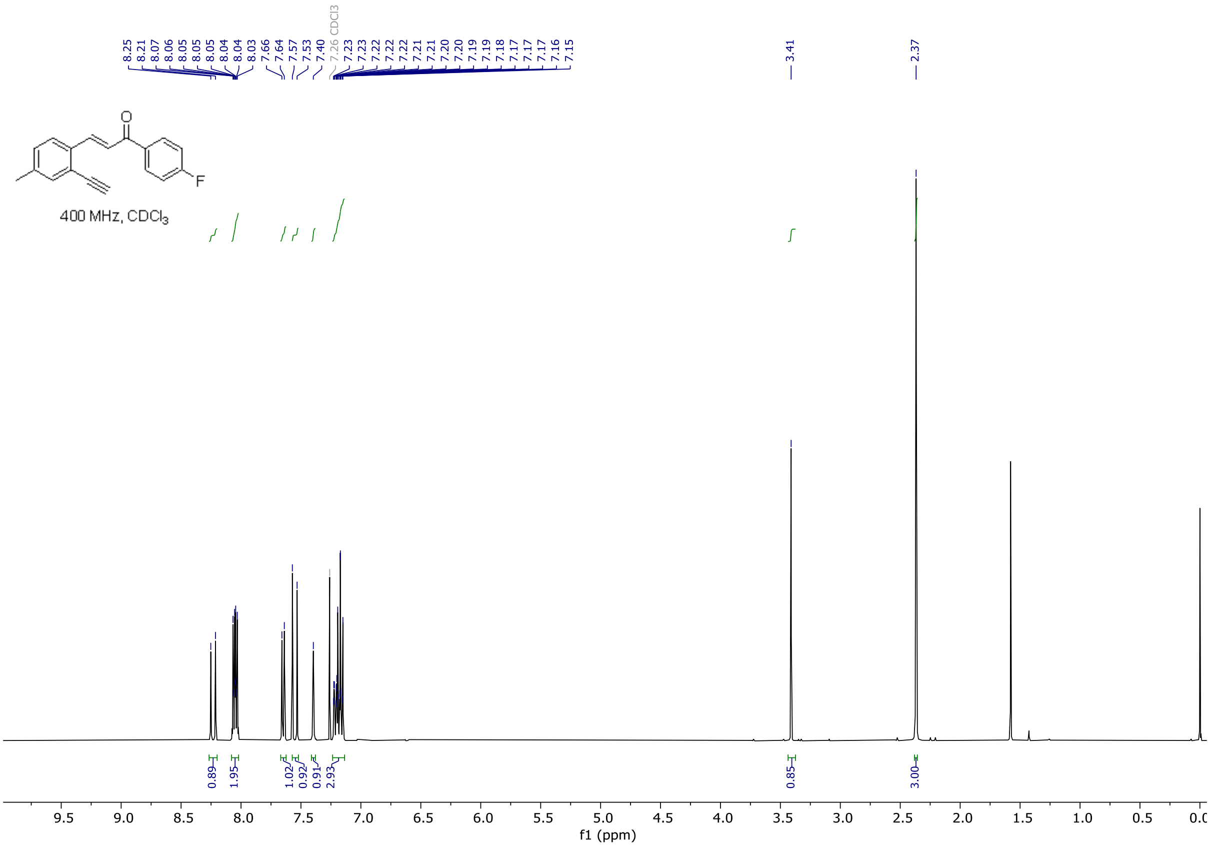

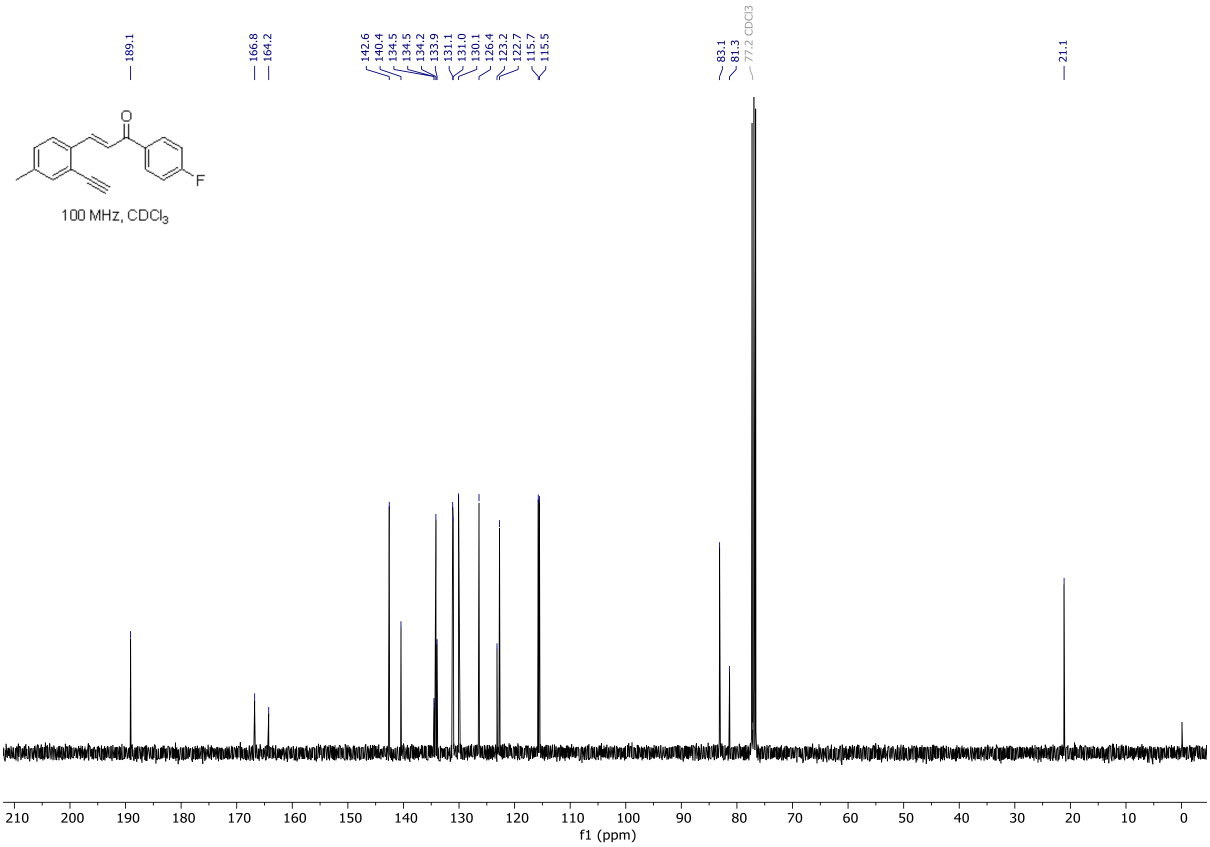

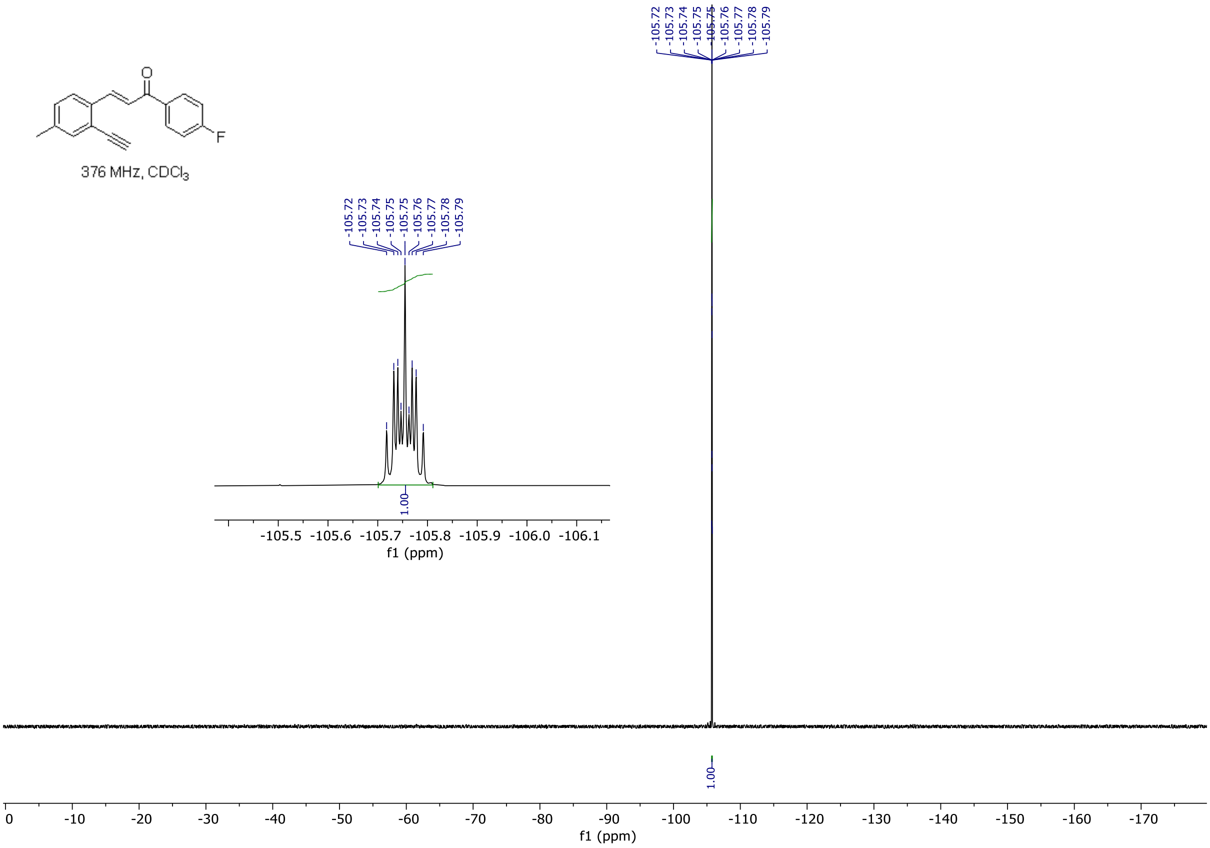

(*E*)-3-(2-Ethynyl-4-fluorophenyl)-1-(p-tolyl)prop-2-en-1-one (4o)

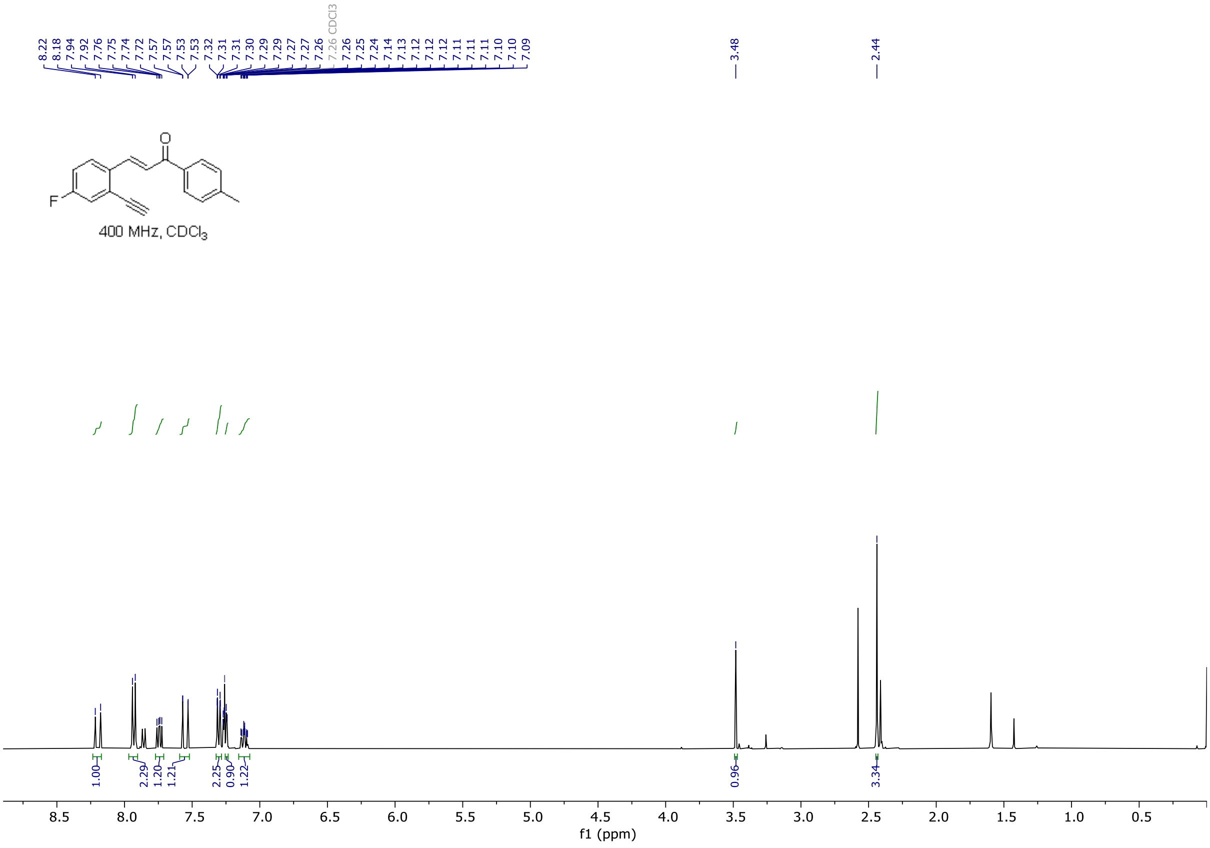

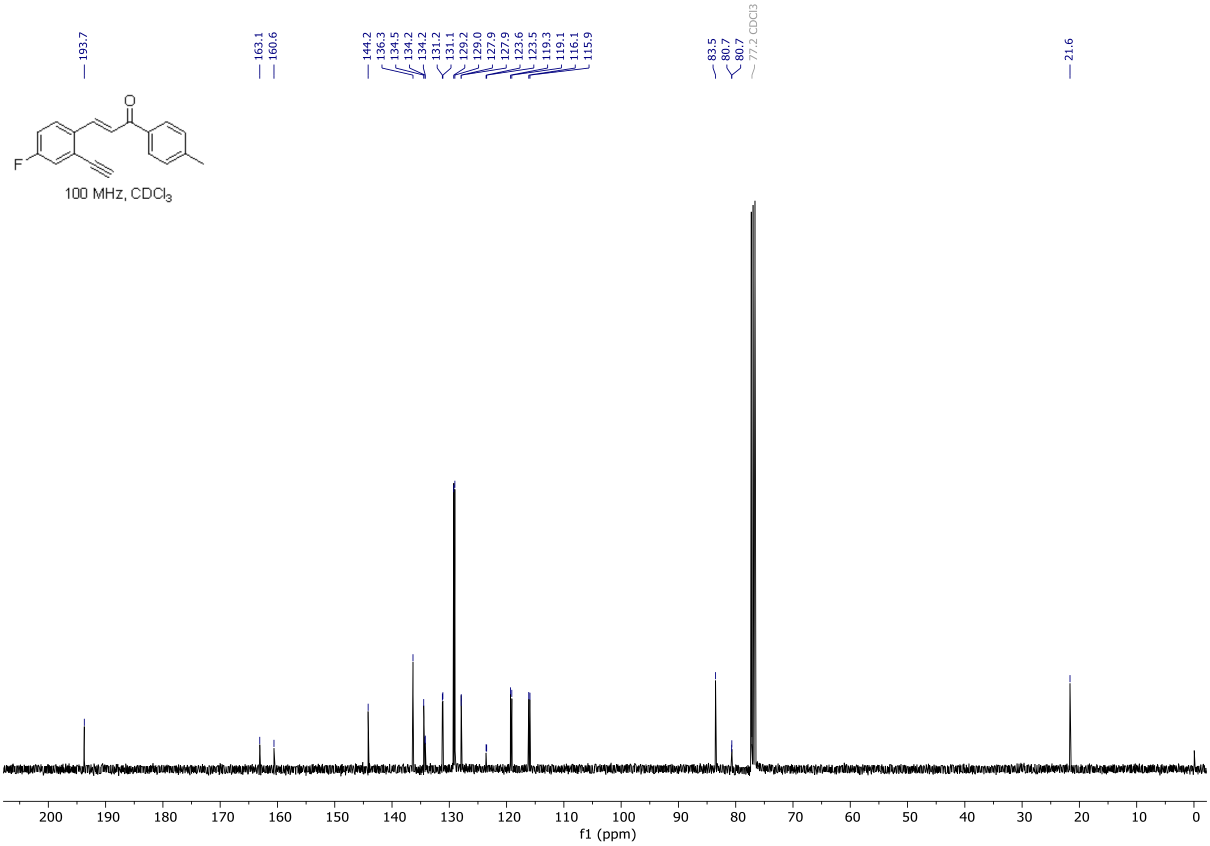
193.7
163.1
160.6
144.2
136.3
134.5
134.2
134.2
131.2
131.1
129.2
129.0
127.9
127.9
123.6
123.5
119.3
119.1
116.1
115.9
83.5
80.7
80.7
77.2 CDCl3
21.6
F
O
100 MHz, $CDCl_3$
f1 (ppm)

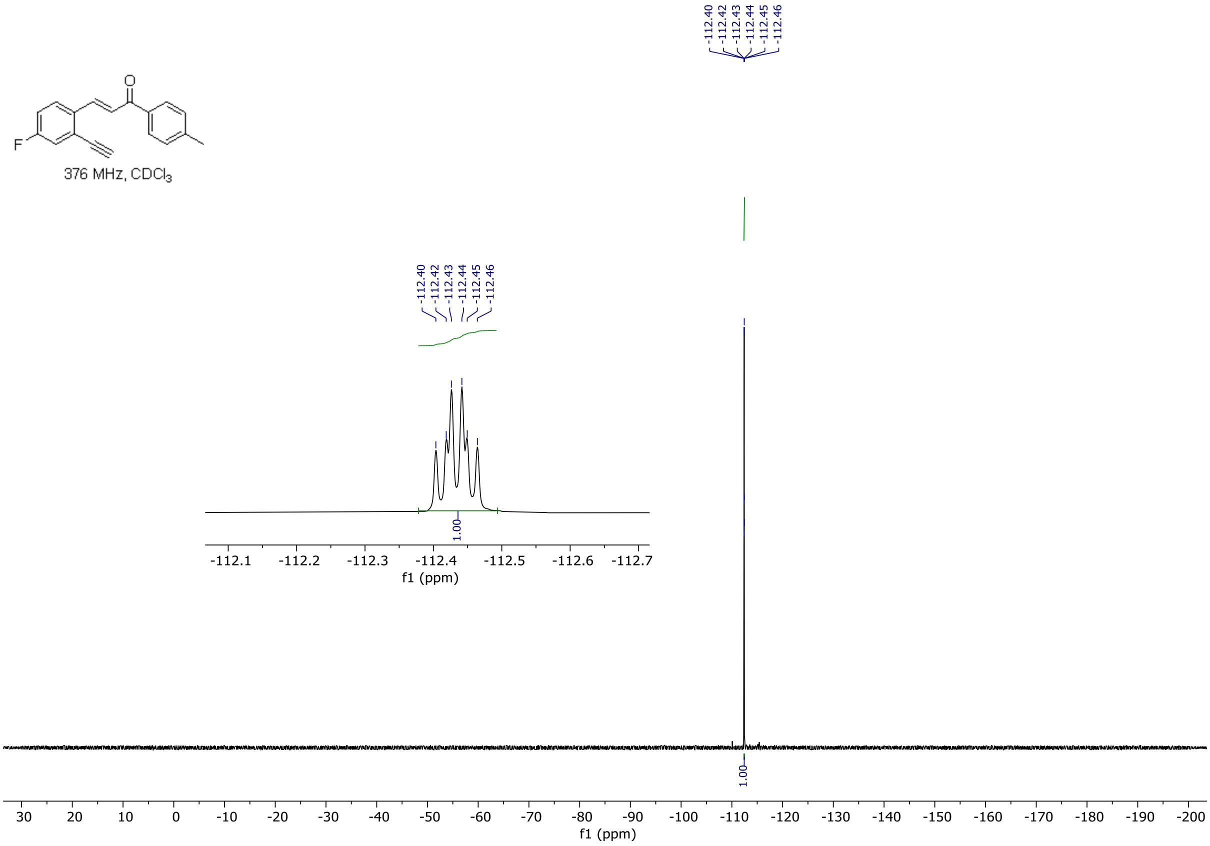
-112.40
-112.42
-112.43
-112.44
-112.45
-112.46
F
O
376 MHz, $CDCl_3$
1.00
f1 (ppm)

(*E*)-3-(2-Ethynylphenyl)-1-phenylprop-2-en-1-one (4p)

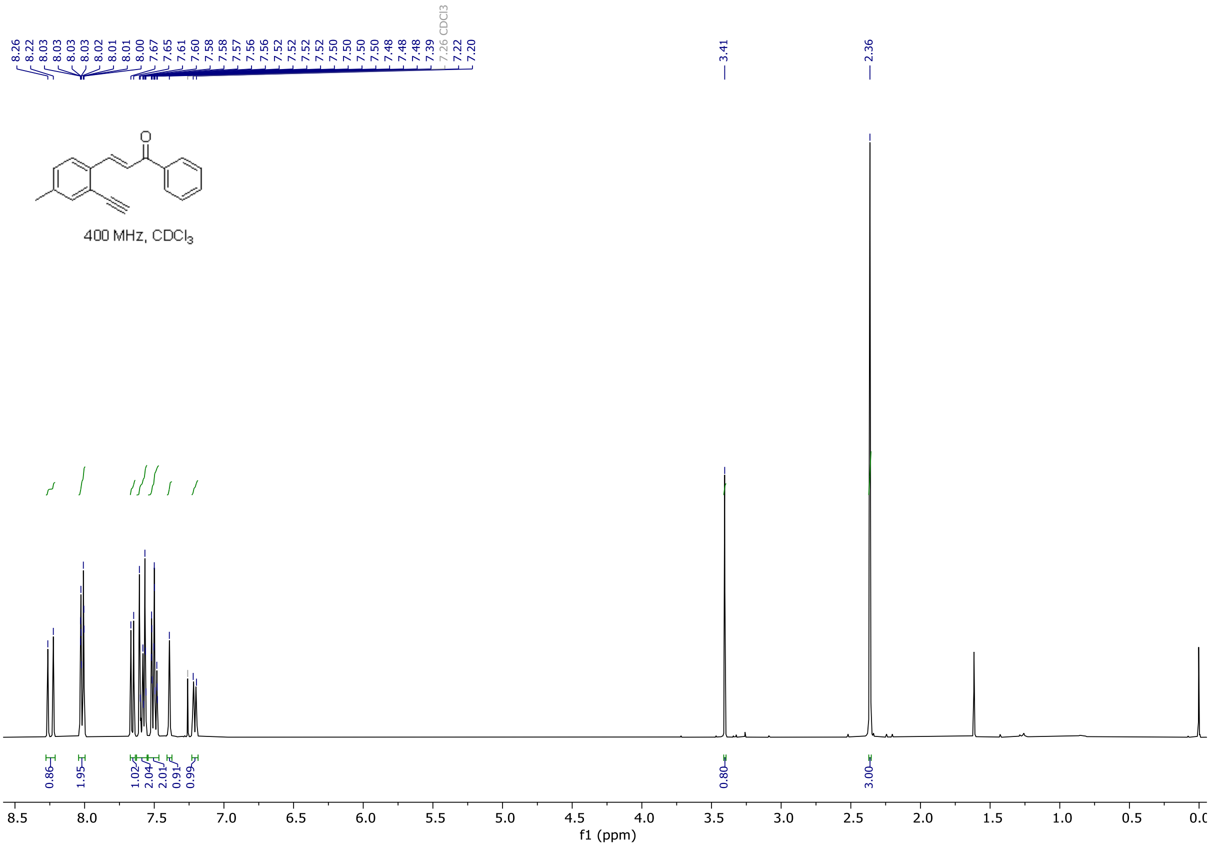

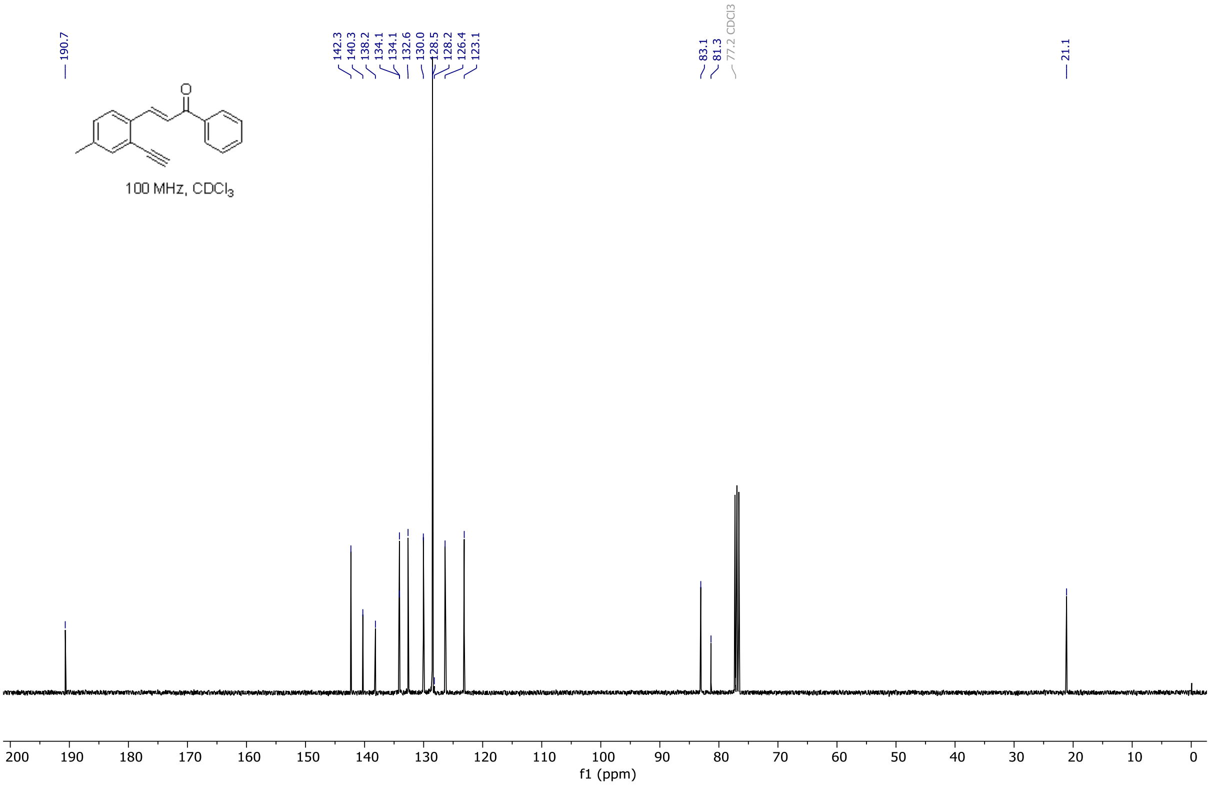

(*E*)-3-(2-Ethynyl-4-fluorophenyl)-1-(4-fluorophenyl)prop-2-en-1-one (4q)

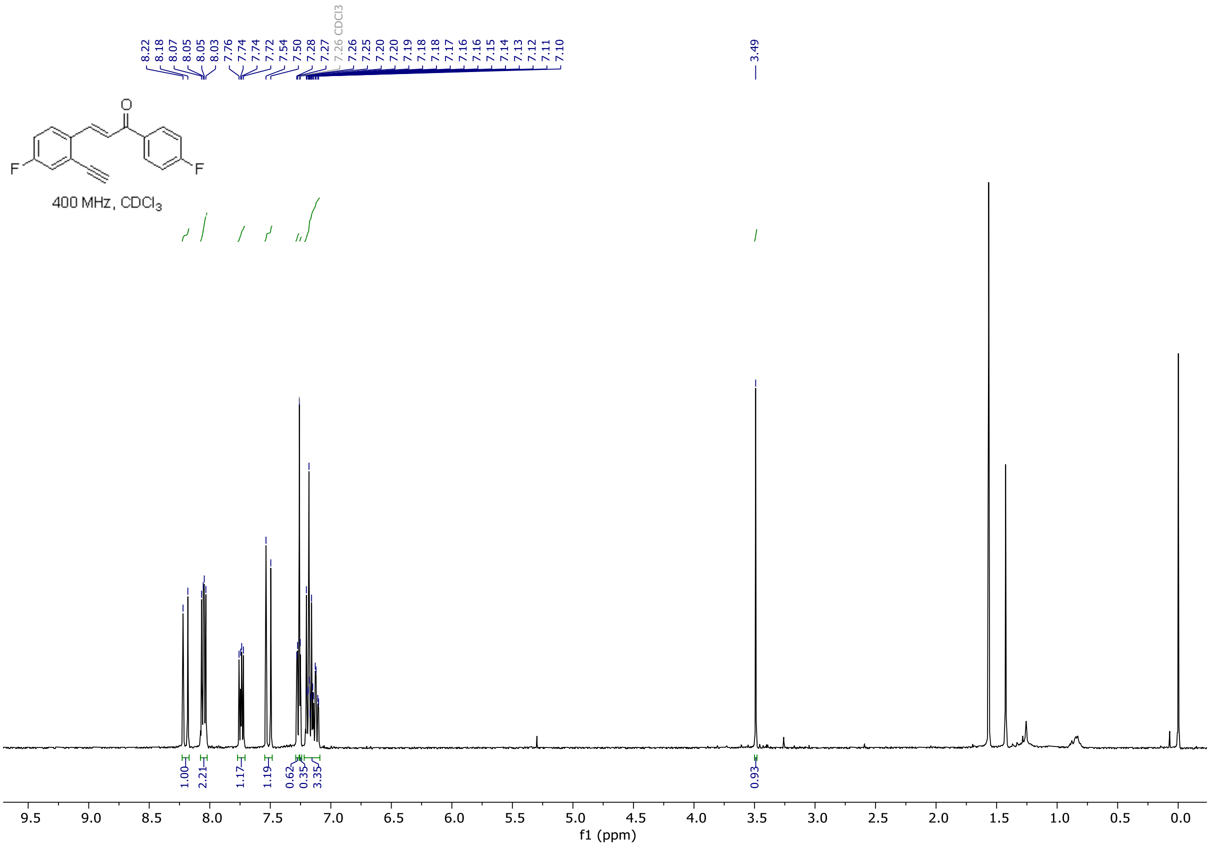

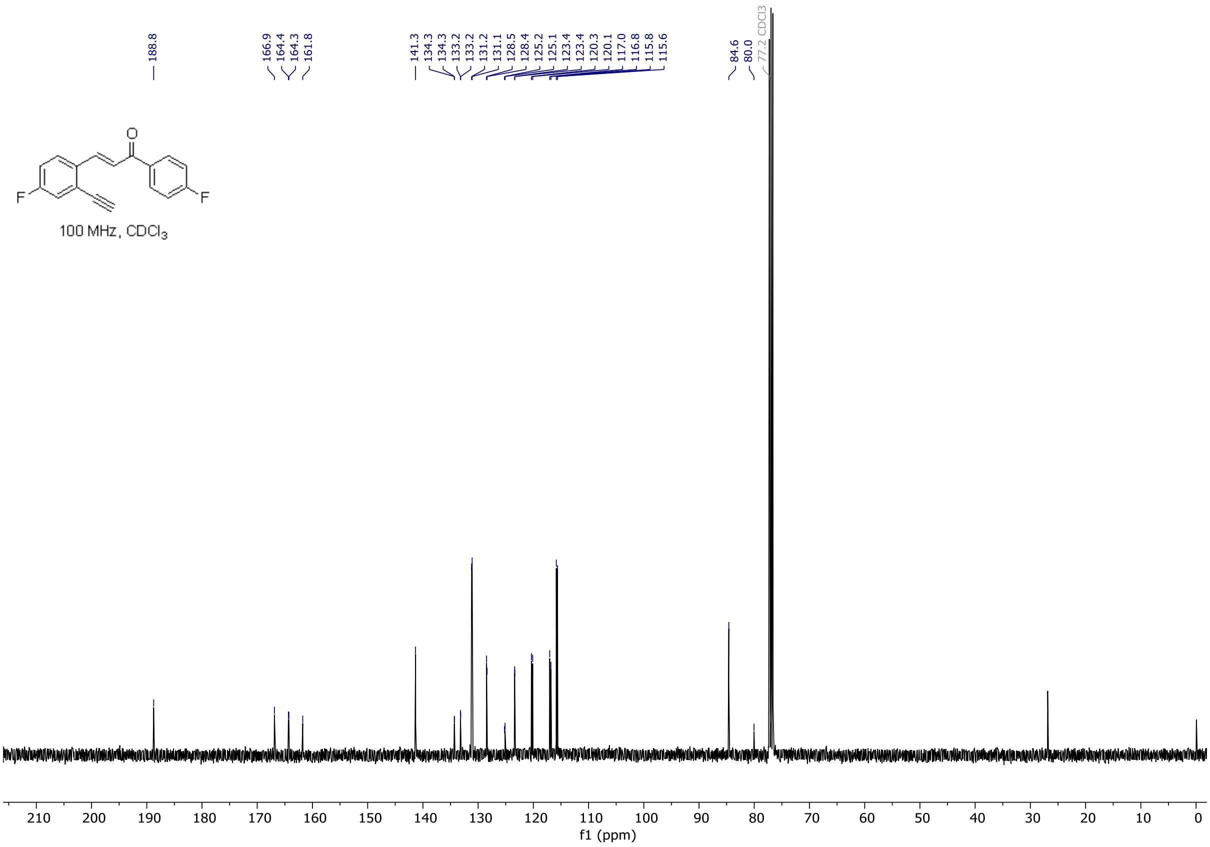

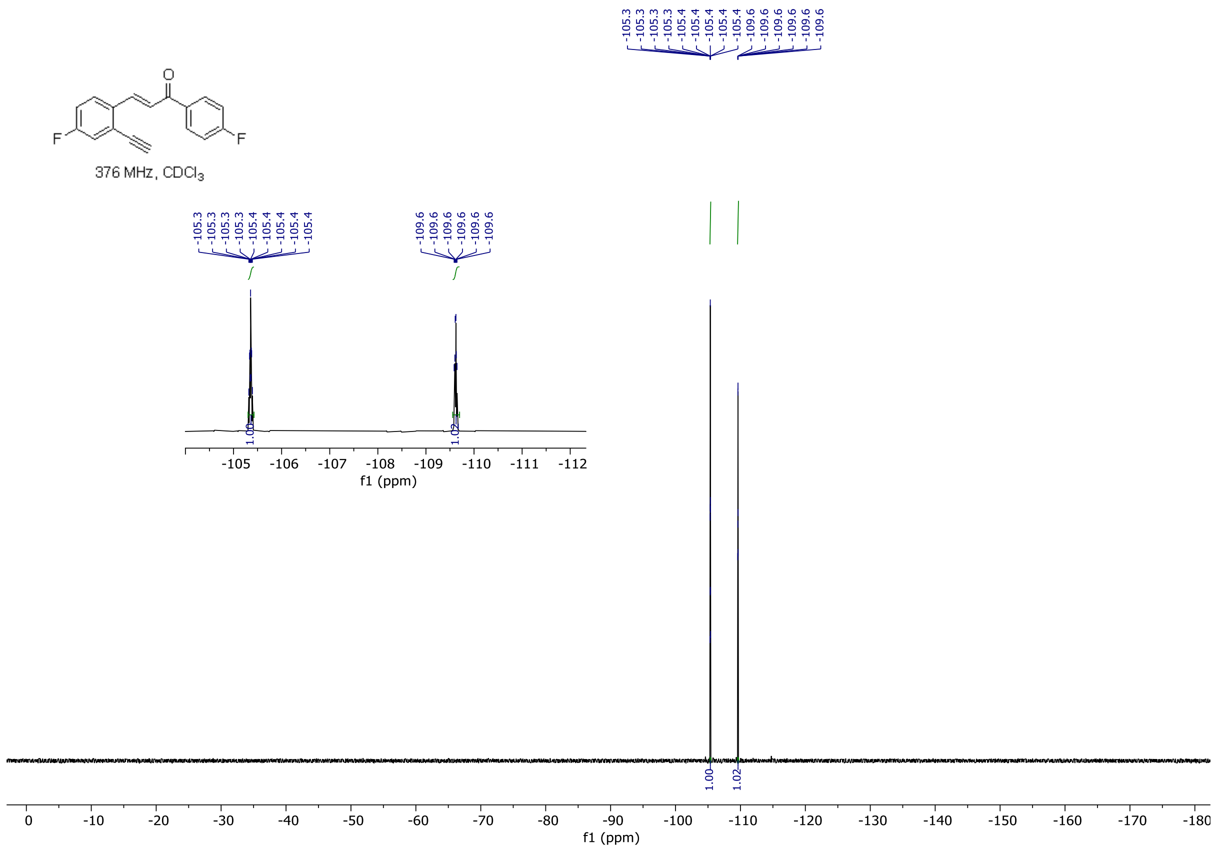

(*E*)-3-(2-Ethynyl-4-methylphenyl)-1-(p-tolyl)prop-2-en-1-one (4r)

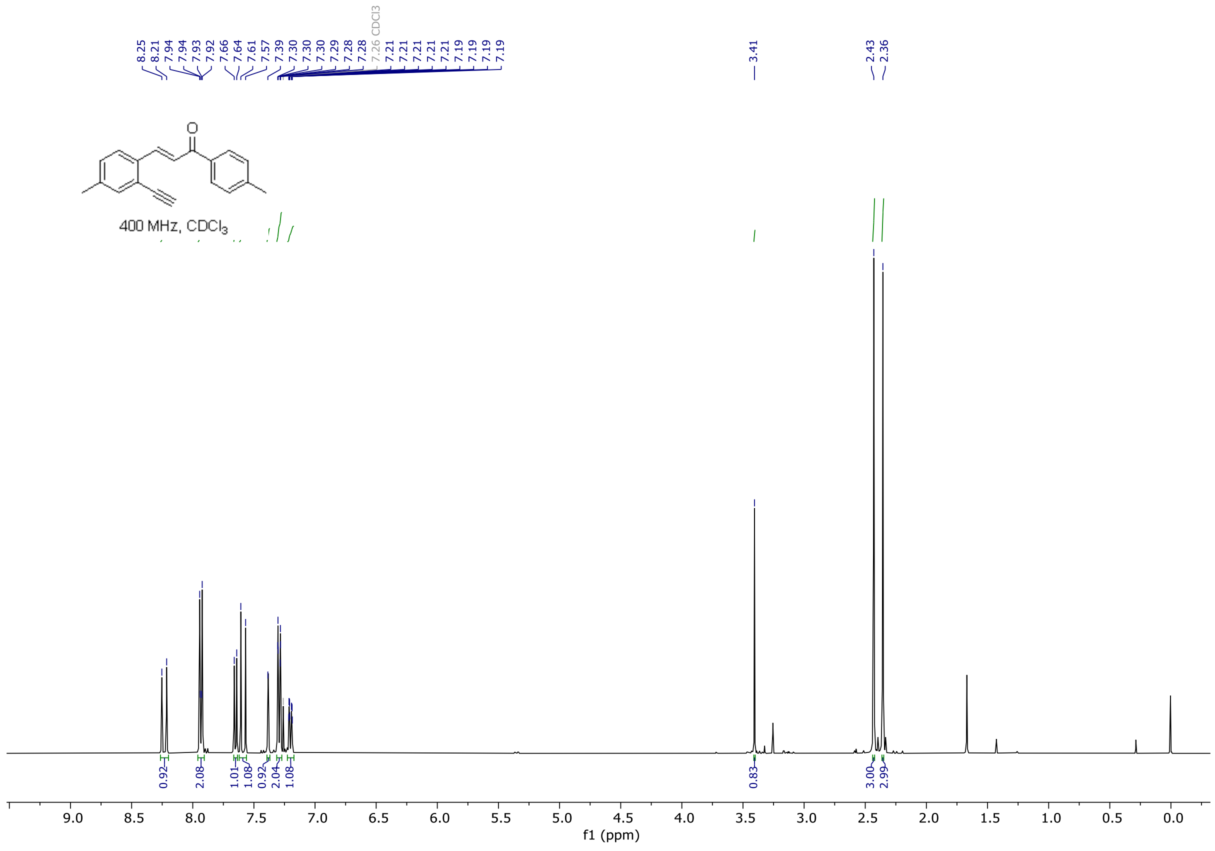

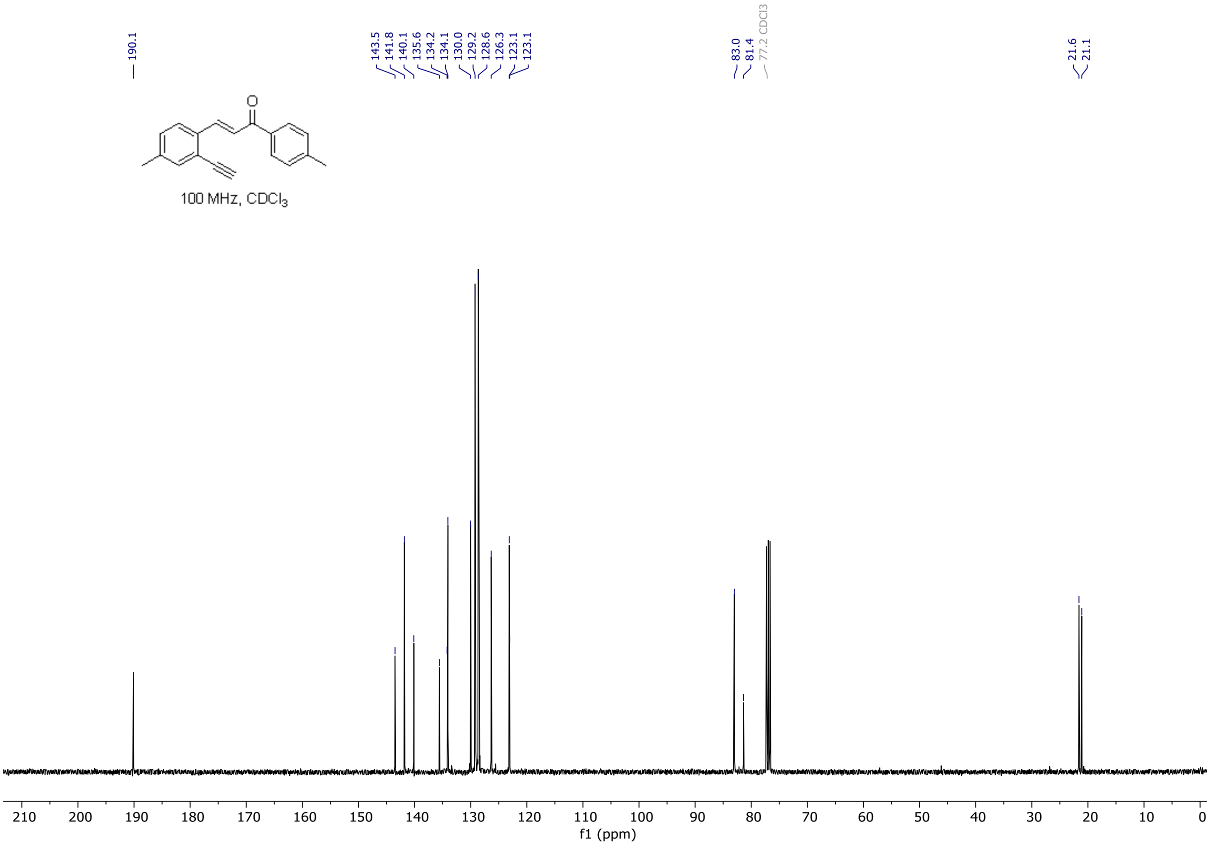

(*E*)-3-(2-Ethynyl-4-fluorophenyl)-1-phenylprop-2-en-1-one (4s)

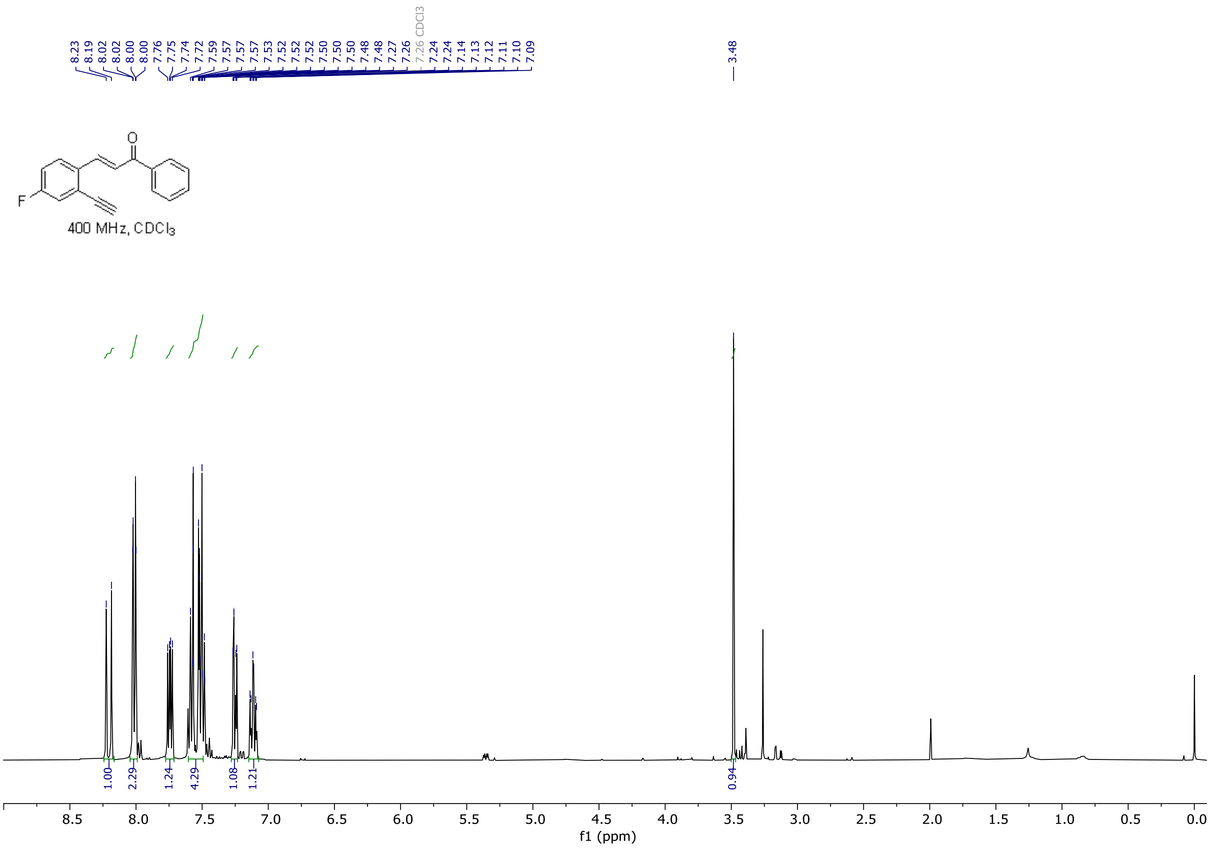

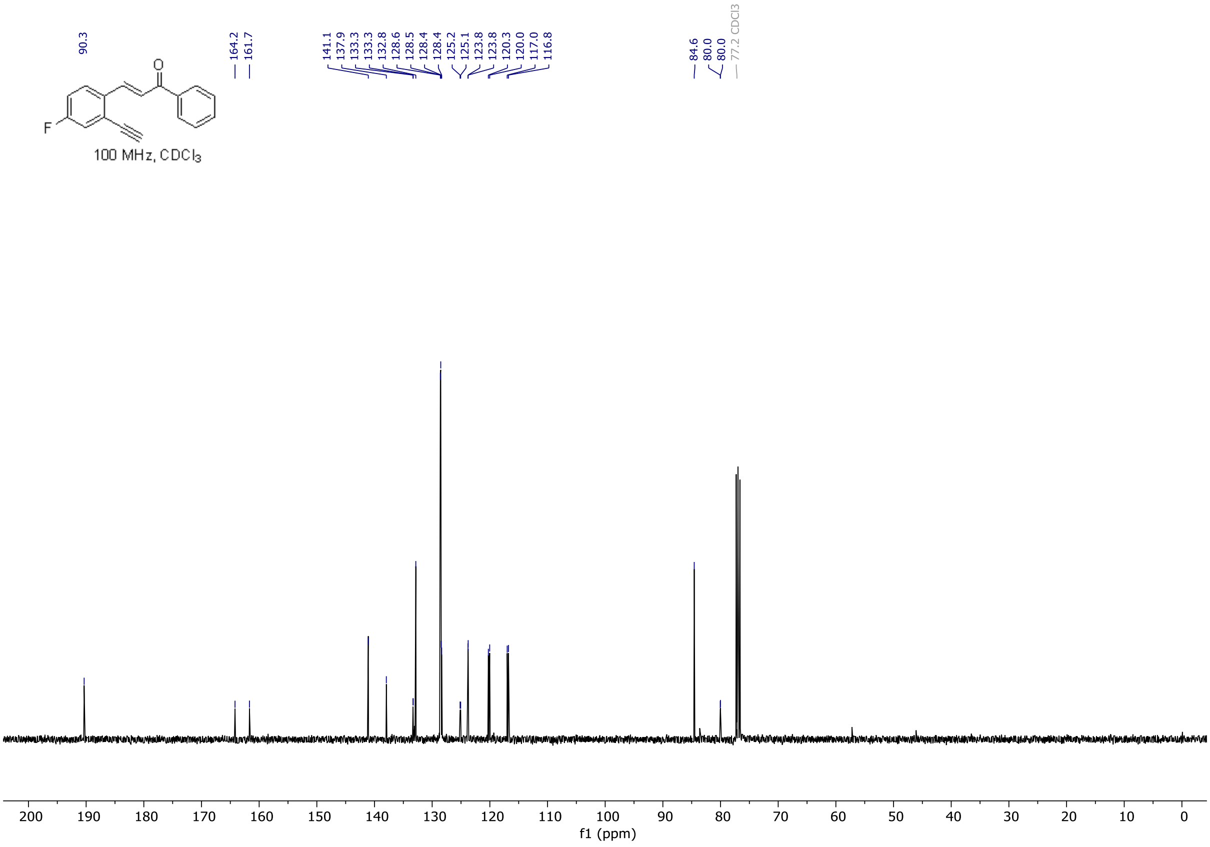

100 MHz, CDCl3
90.3
164.2
161.7
141.1
137.9
133.3
133.3
132.8
128.6
128.5
128.4
128.4
125.2
125.1
123.8
123.8
120.3
120.0
117.0
116.8
84.6
80.0
80.0
77.2 CDCl3
f1 (ppm)

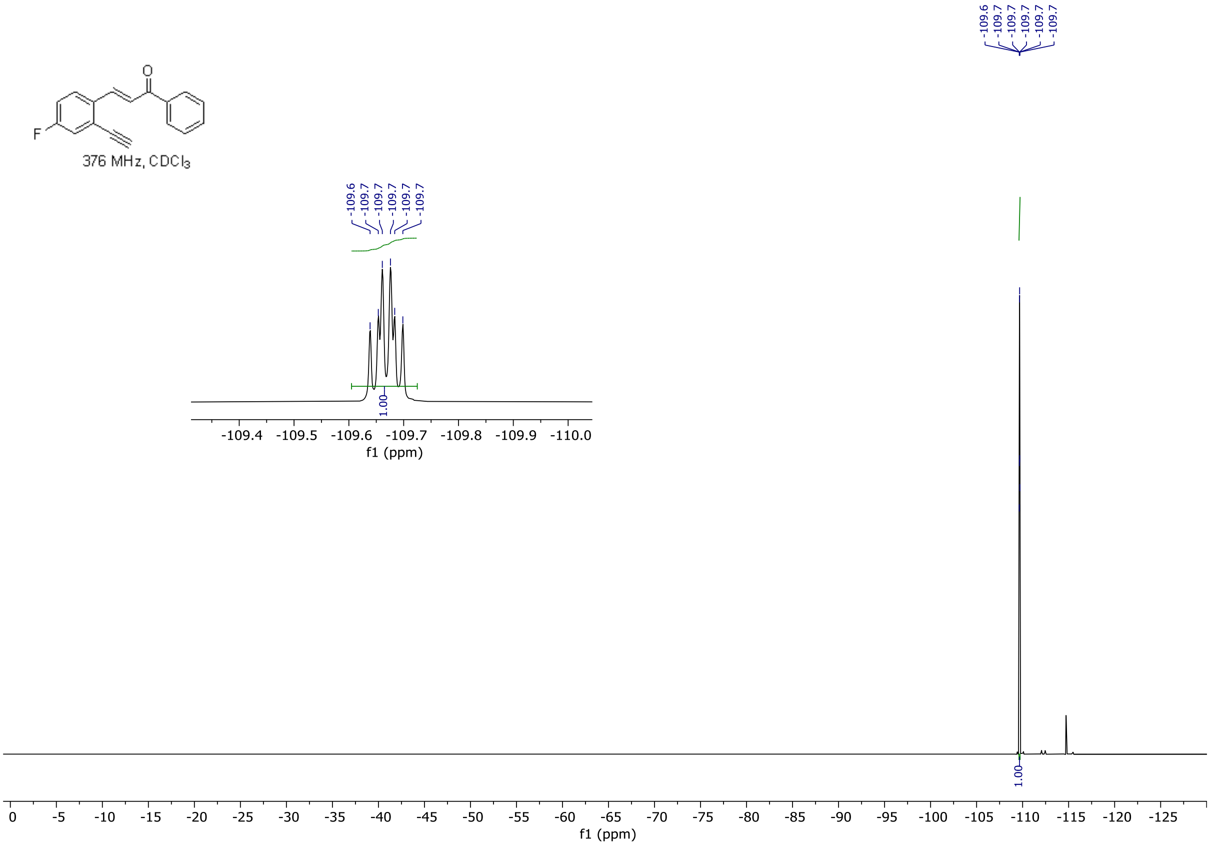

376 MHz, CDCl3
-109.6
-109.7
-109.7
-109.7
-109.7
-109.7
1.00
f1 (ppm)

9-Methoxy-11*H*-benzo[*b*]fluoren-10-ol (5a)

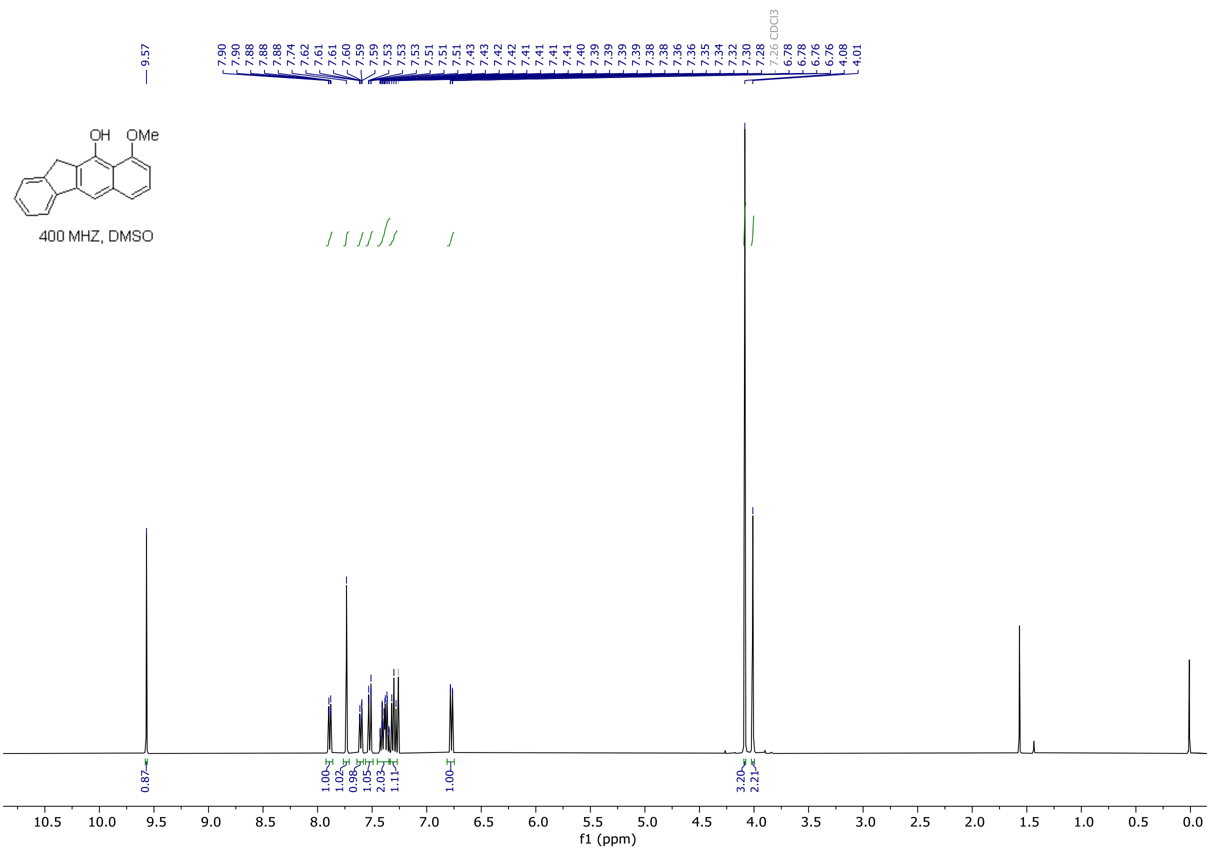

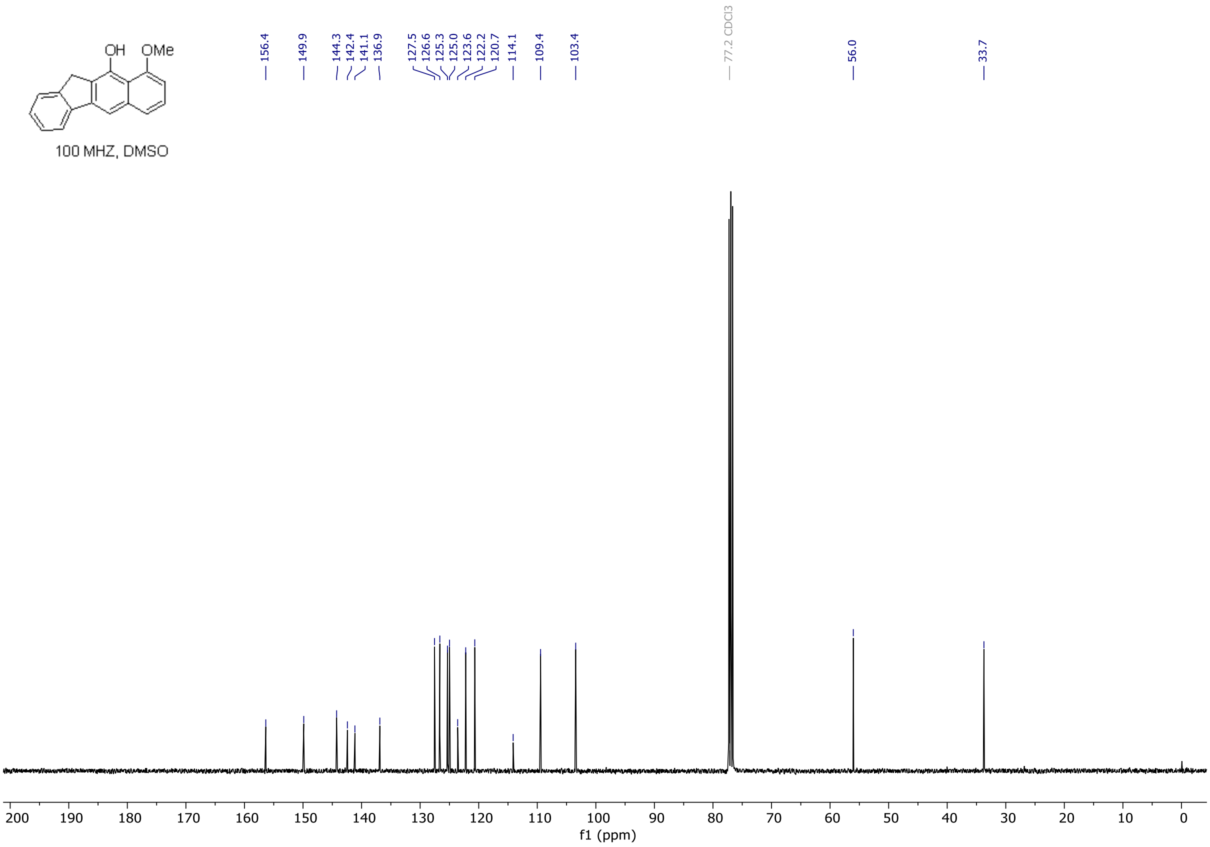

11*H*-Benzo[*b*]fluoren-10-ol (5b)

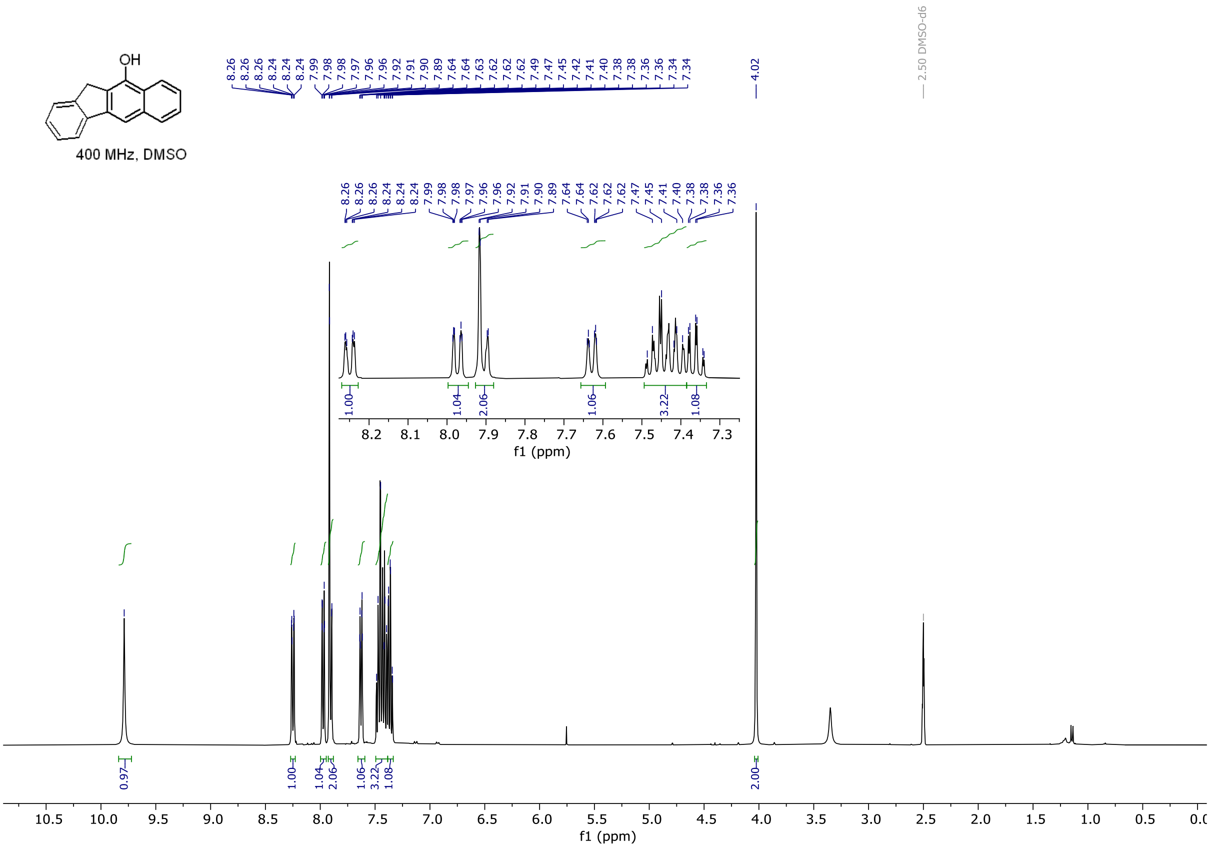

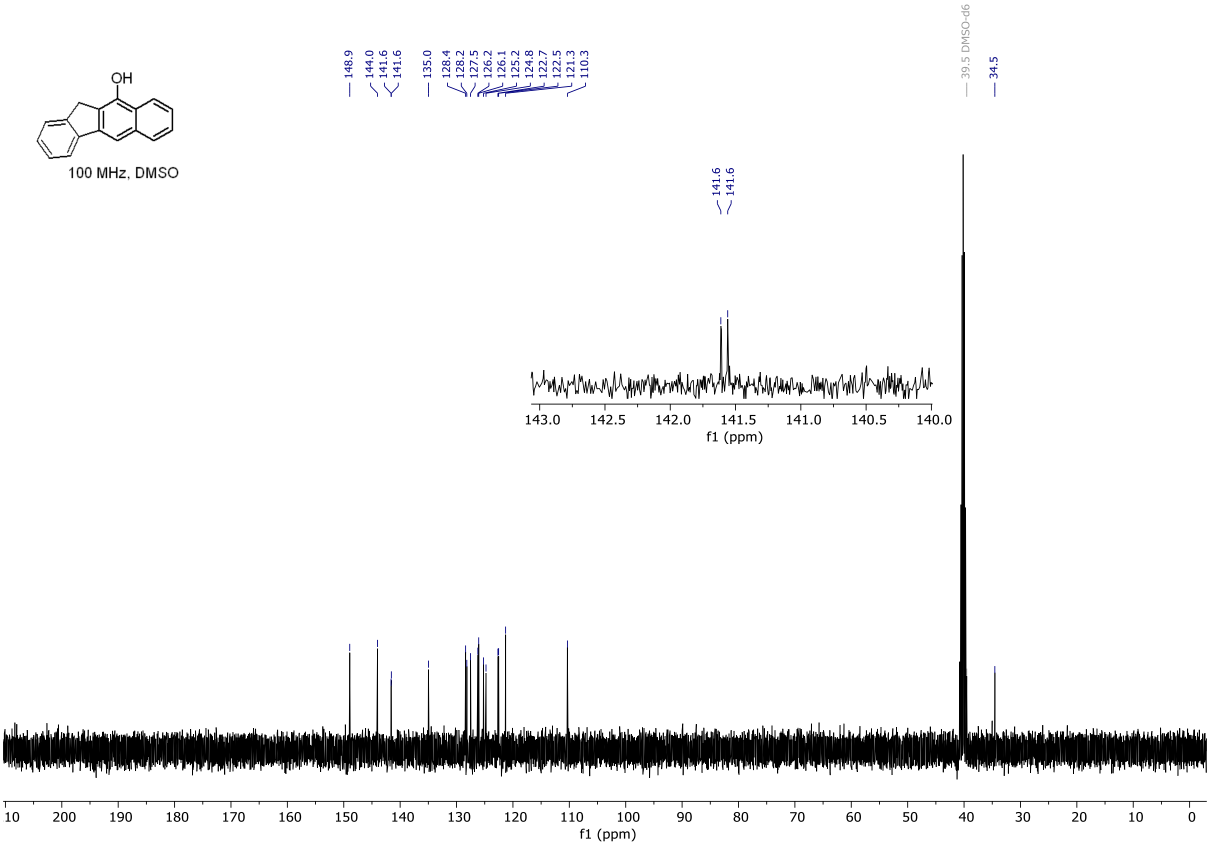

7-(Trifluoromethyl)-11*H*-benzo[*b*]fluoren-10-ol (5c)

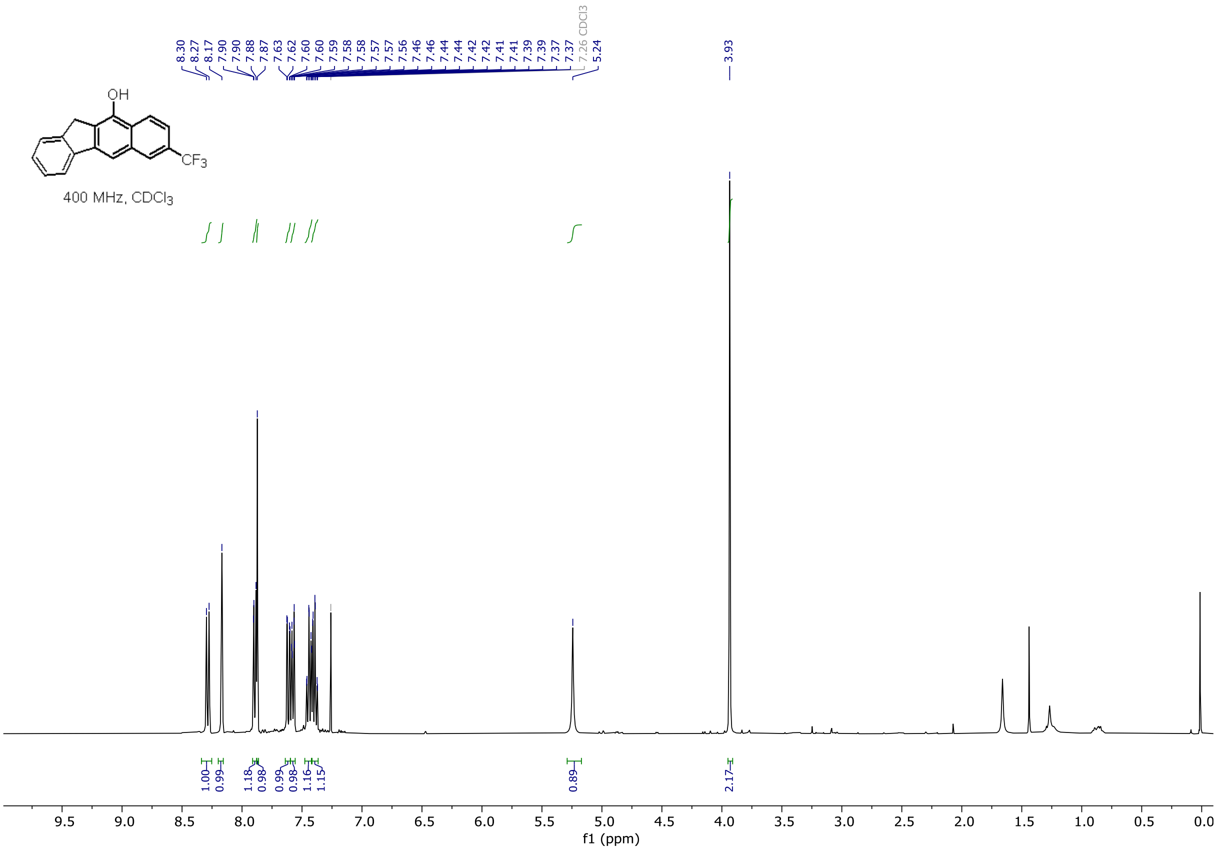

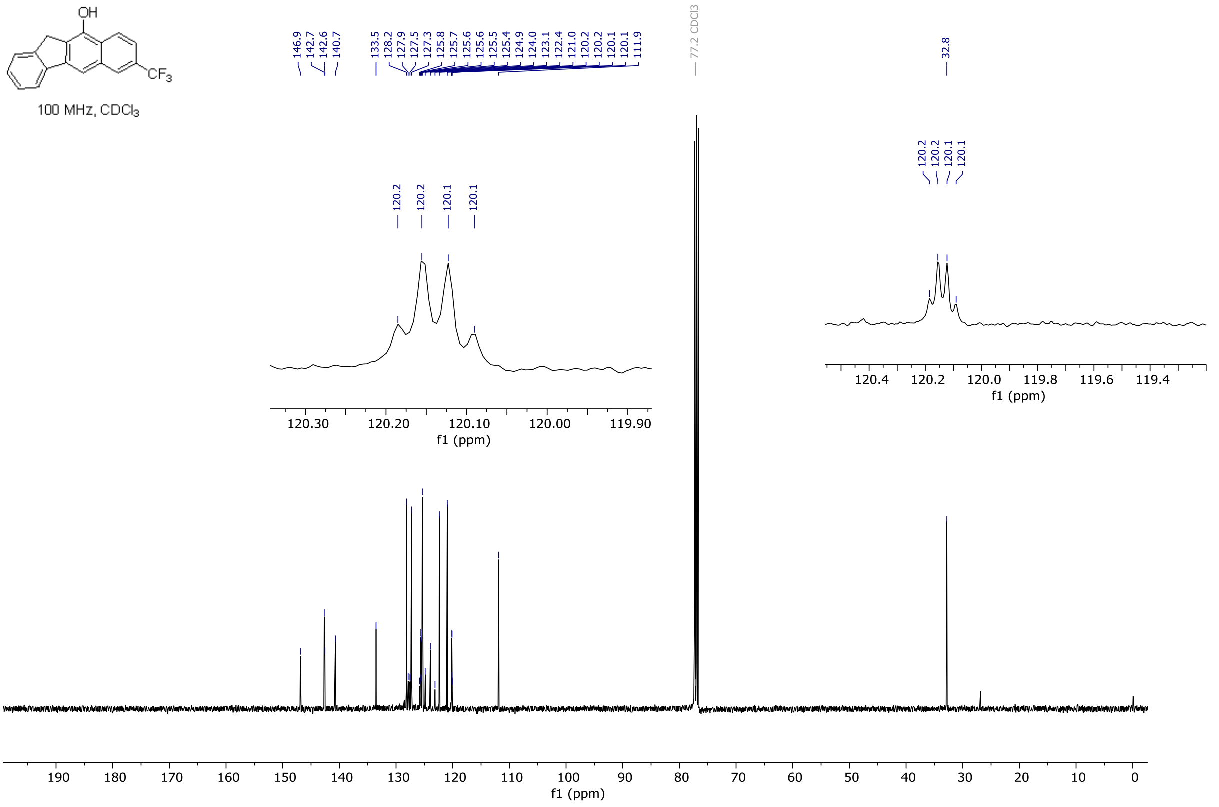

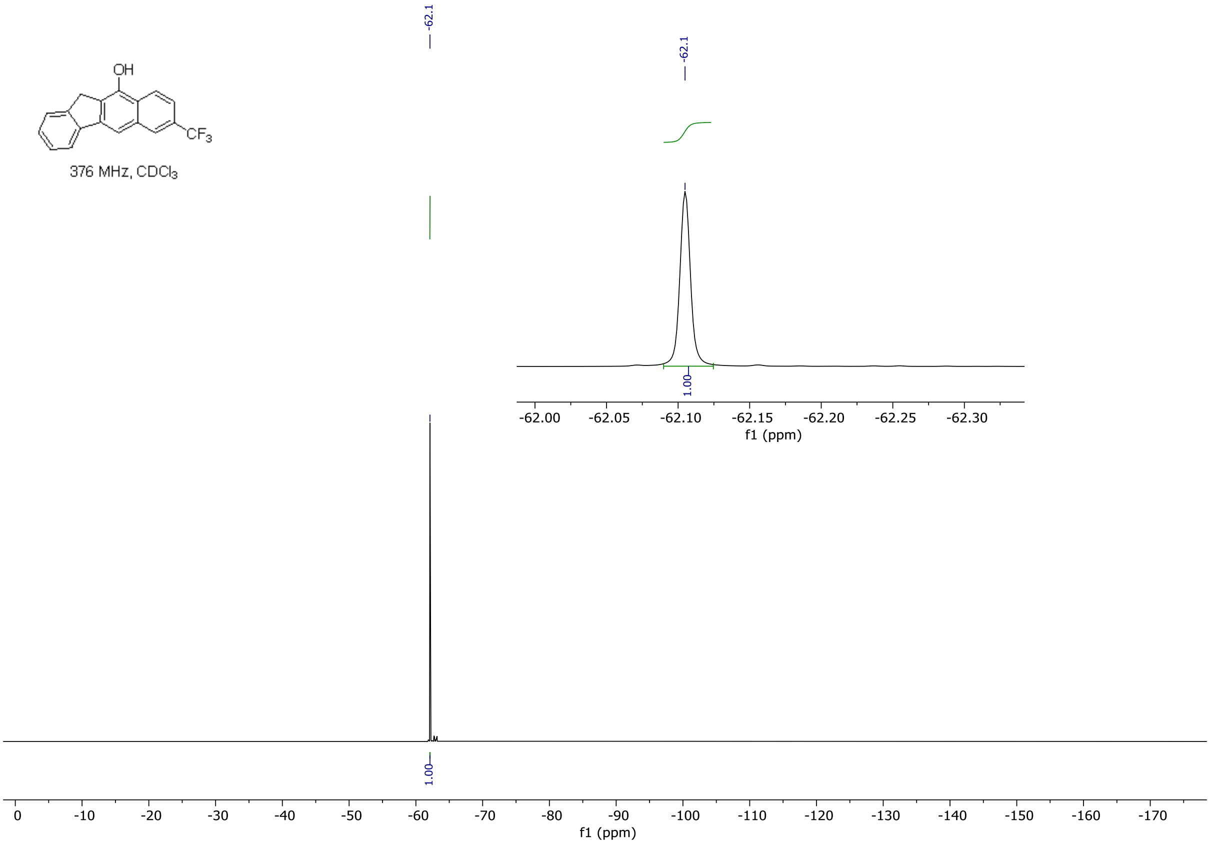

7-Fluoro-11*H*-benzo[*b*]fluoren-10-ol (5d)

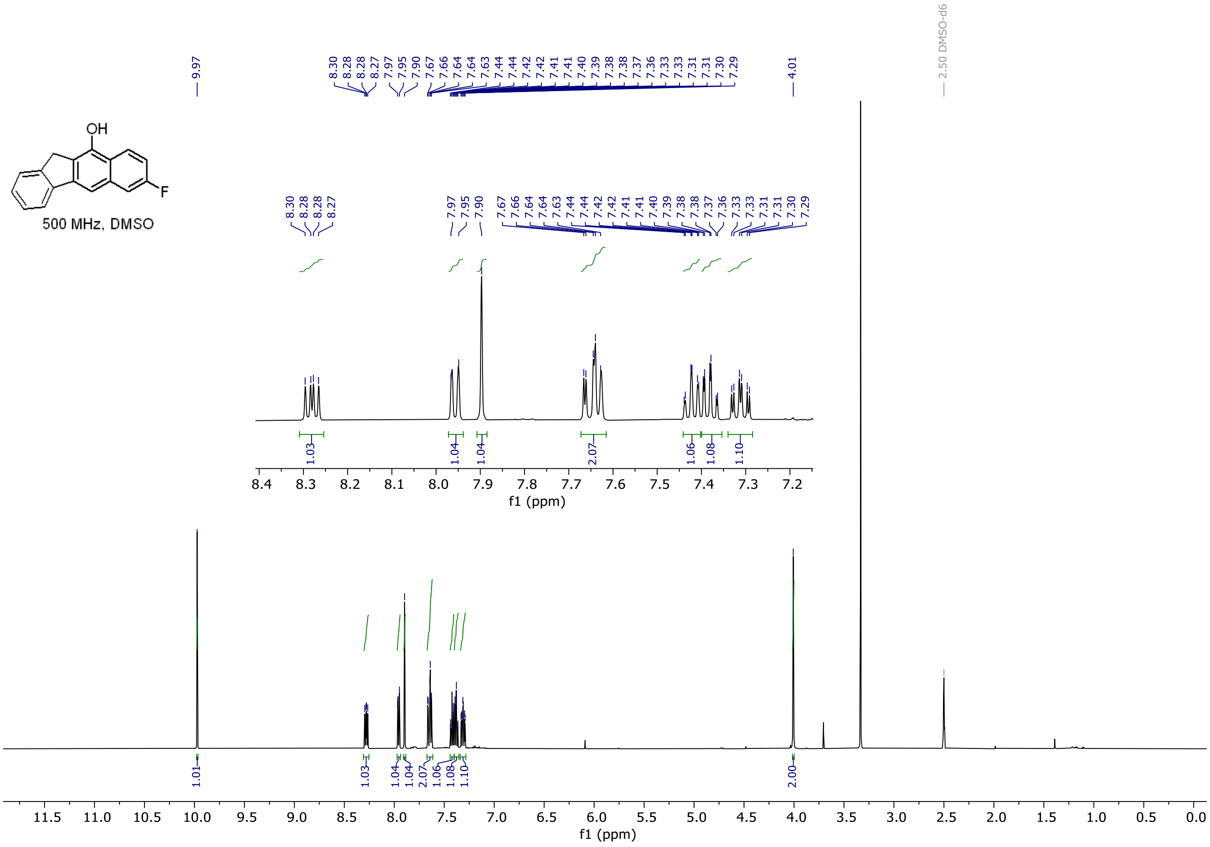

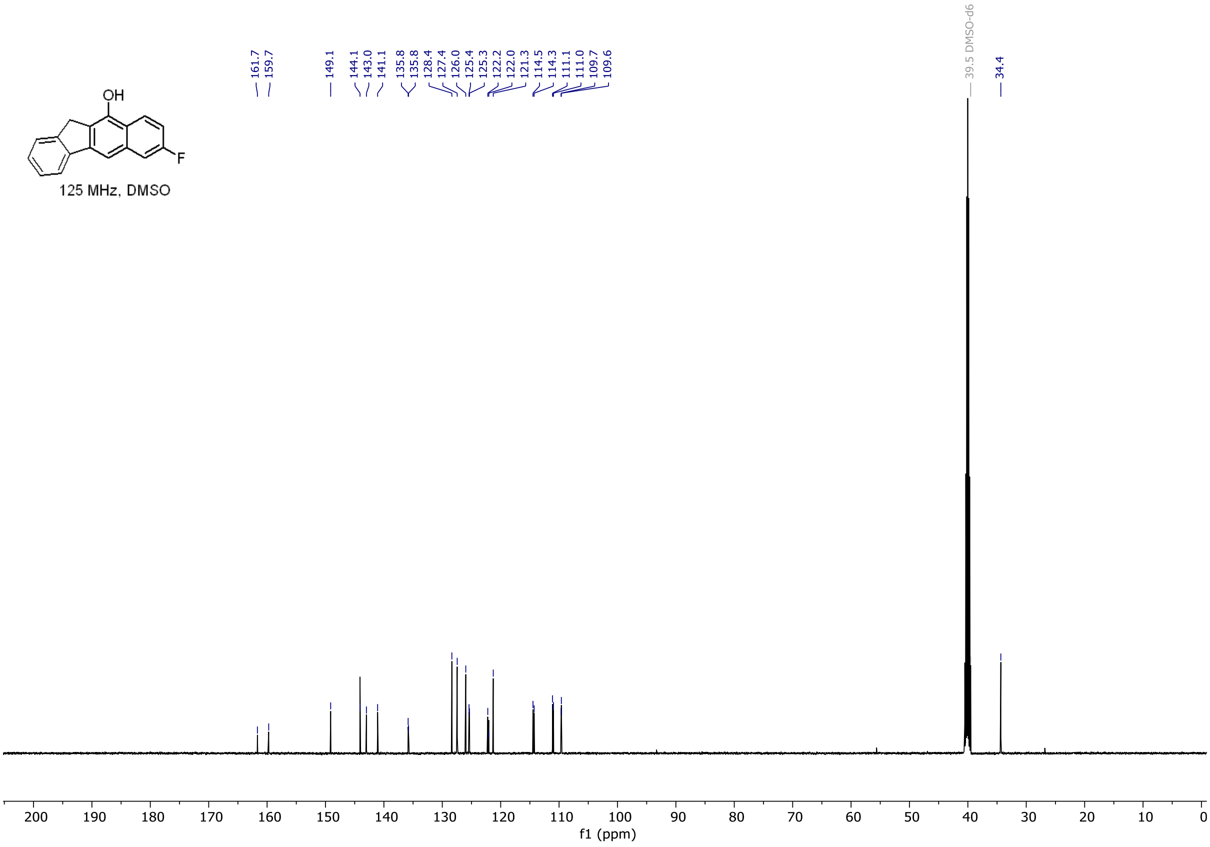
OH
F
125 MHz, DMSO
161.7
159.7
149.1
144.1
143.0
141.1
135.8
135.8
128.4
127.4
126.0
125.4
125.3
122.2
122.0
121.3
114.5
114.3
111.1
111.0
109.7
109.6
39.5 DMSO-d6
34.4
200 190 180 170 160 150 140 130 120 110 100 90 80 70 60 50 40 30 20 10 0
f1 (ppm)

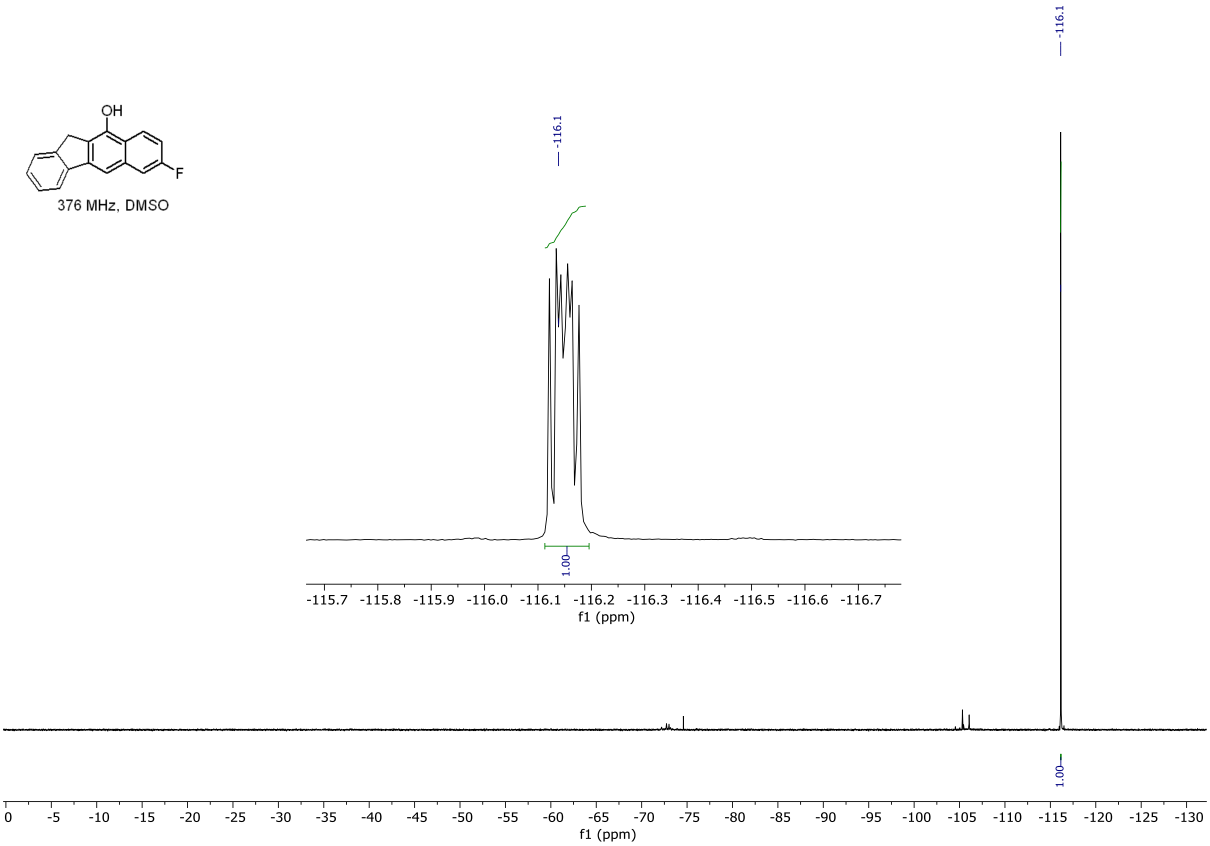
OH
F
376 MHz, DMSO
-116.1
-116.1
1.00
-115.7 -115.8 -115.9 -116.0 -116.1 -116.2 -116.3 -116.4 -116.5 -116.6 -116.7
f1 (ppm)
1.00
0 -5 -10 -15 -20 -25 -30 -35 -40 -45 -50 -55 -60 -65 -70 -75 -80 -85 -90 -95 -100 -105 -110 -115 -120 -125 -130
f1 (ppm)

10-Hydroxy-11*H*-benzo[*b*]fluorene-7-carbonitrile (5e)

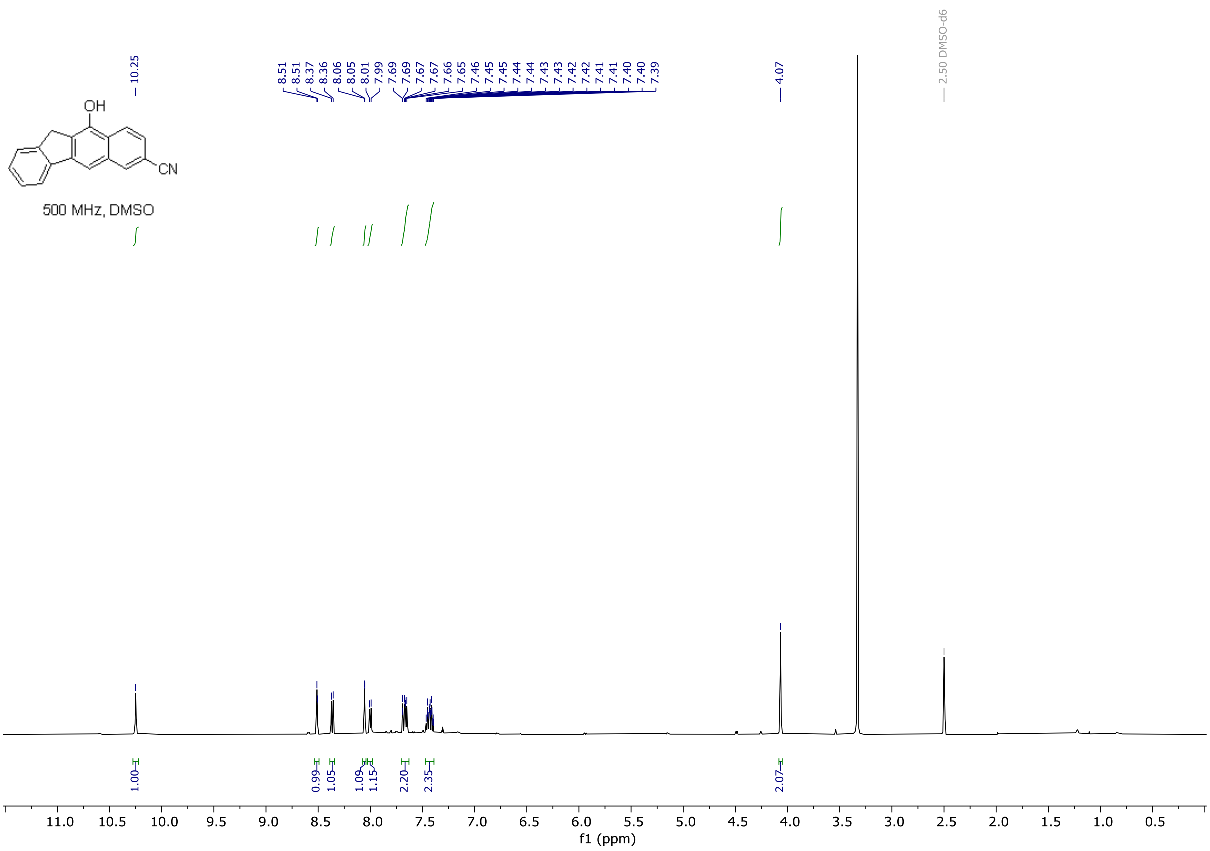

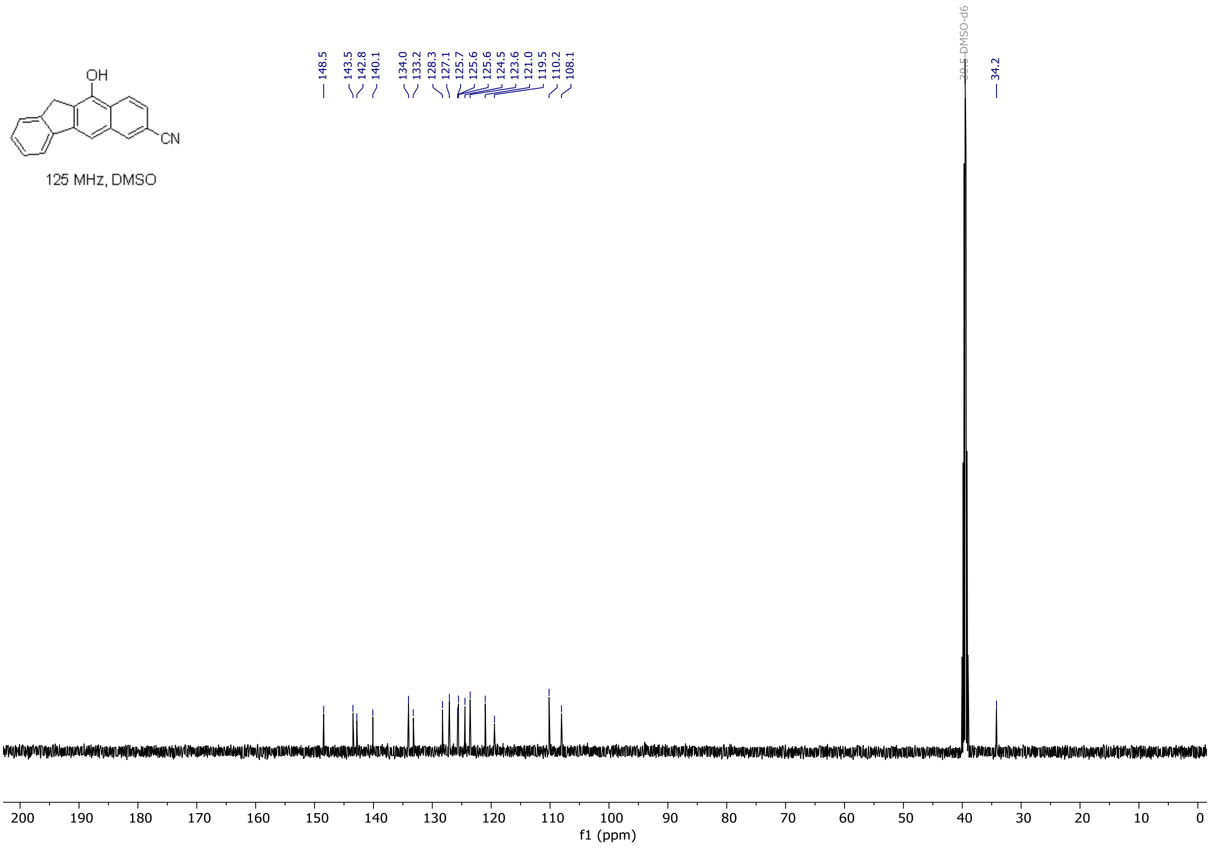

7-Methoxy-11*H*-benzo[*b*]fluoren-10-ol (5f)

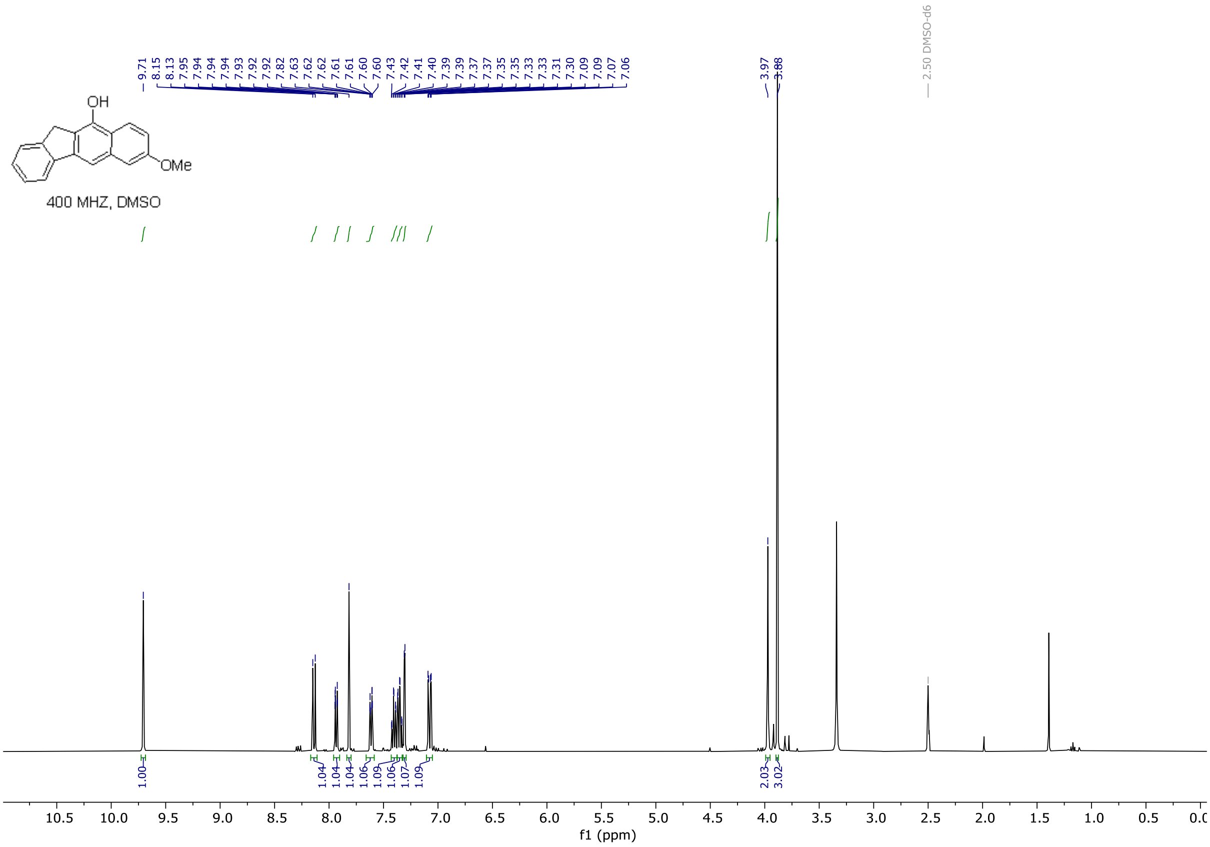

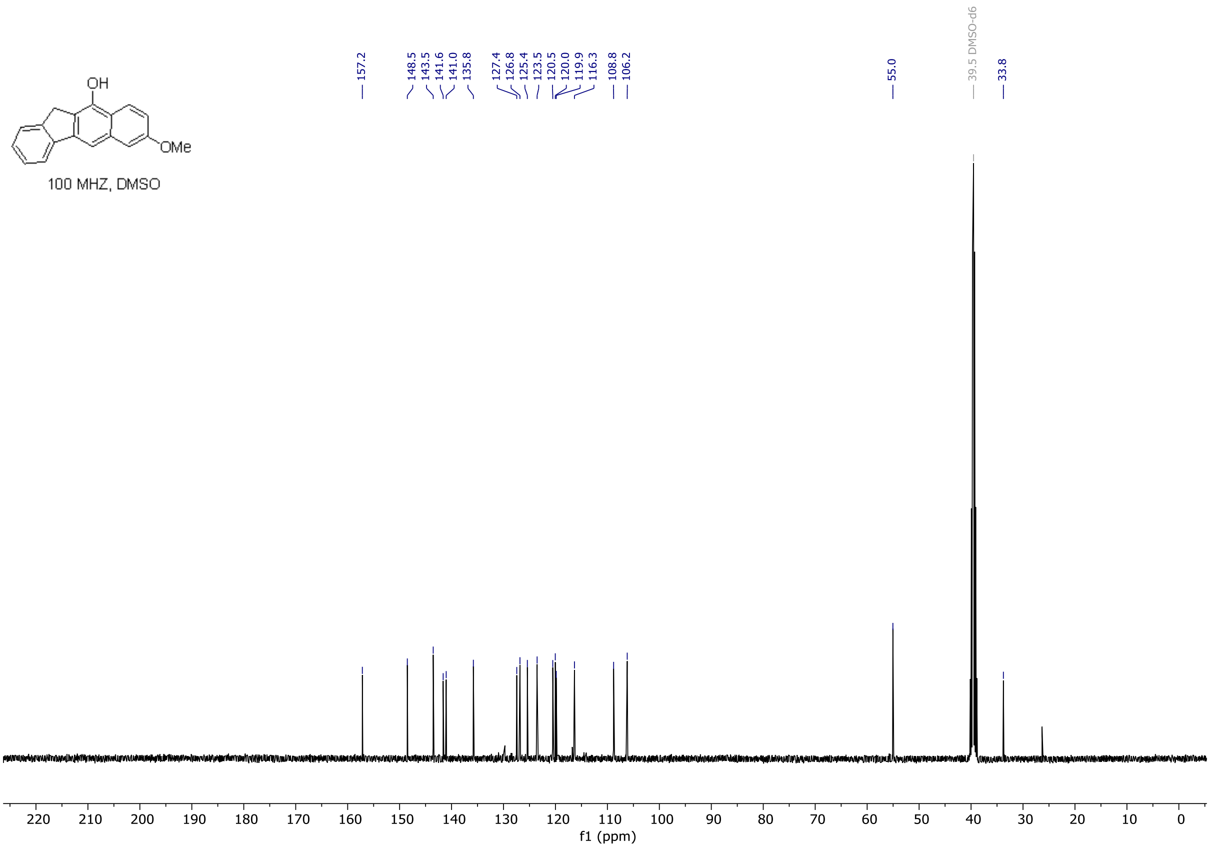

7-Methyl-11*H*-benzo[*b*]fluoren-10-ol (5g)

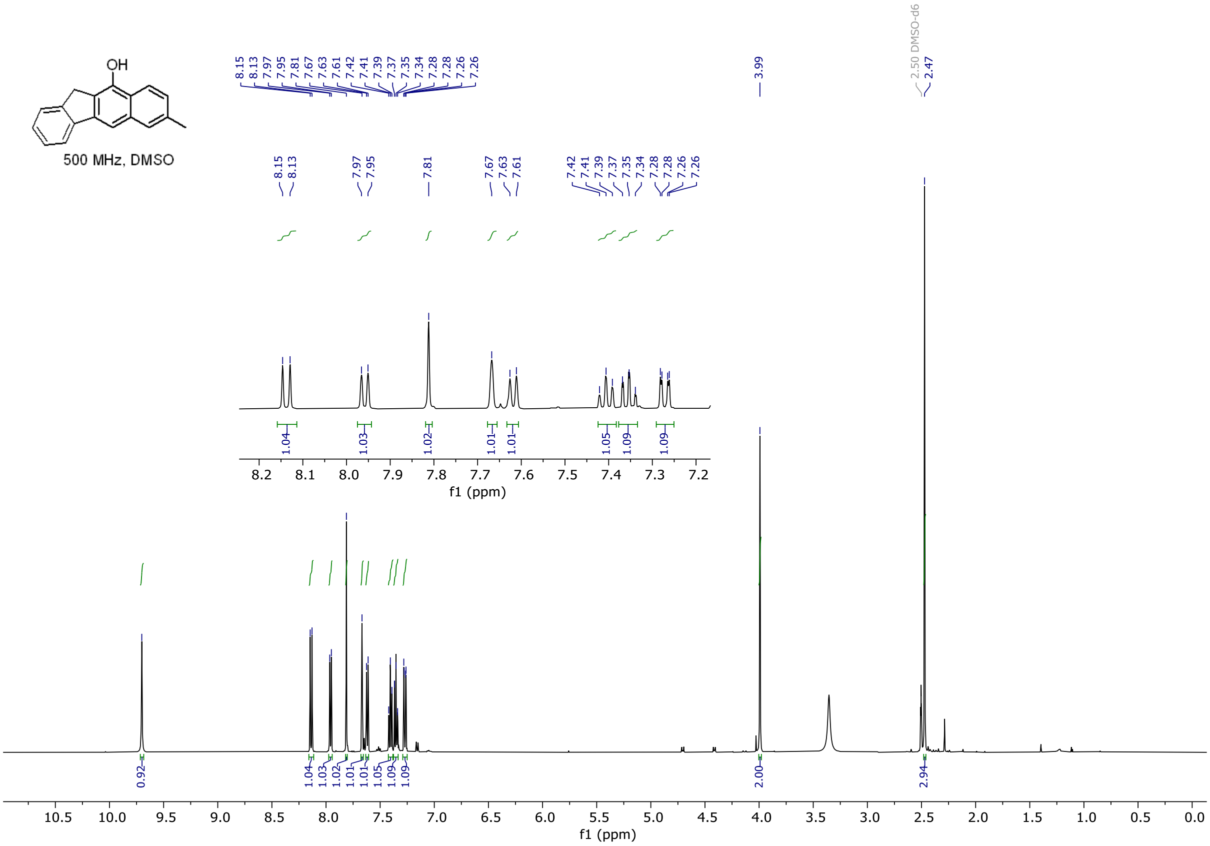

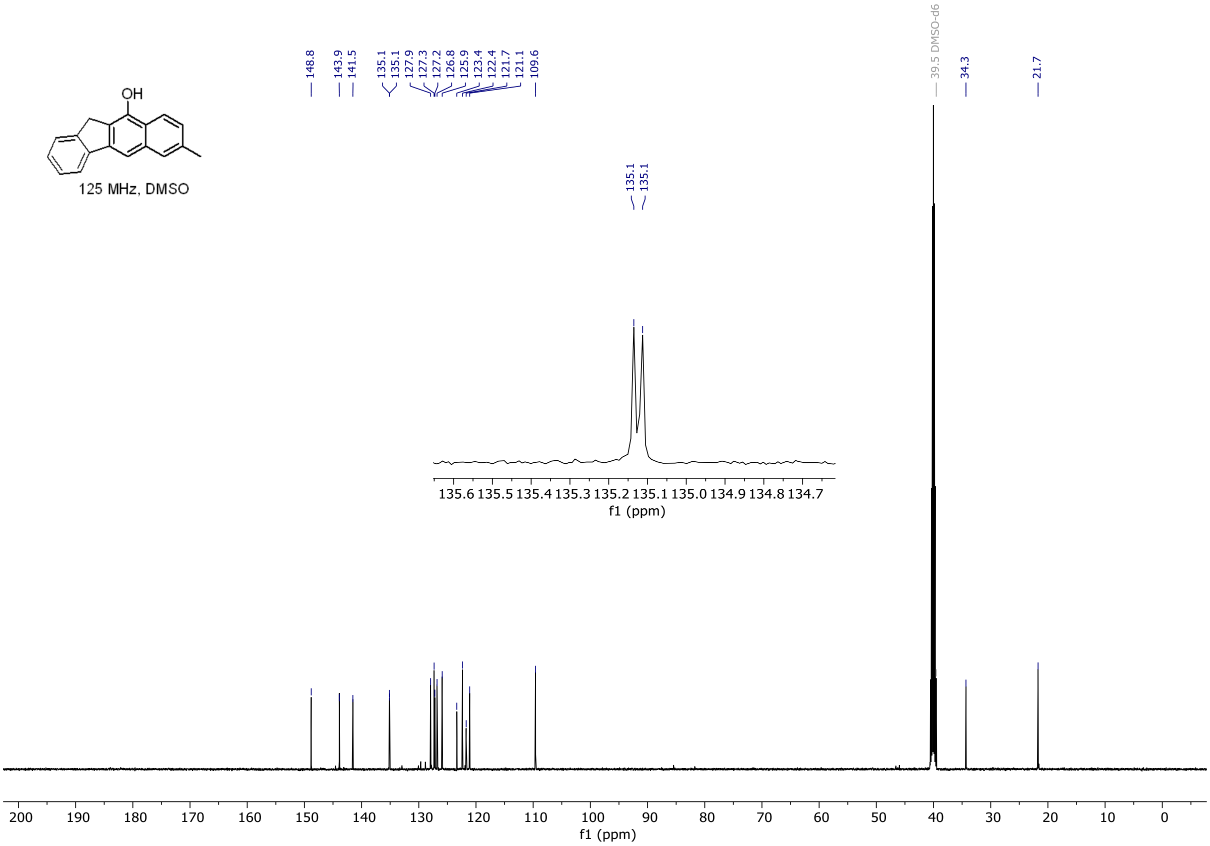

7,8-Dimethyl-11*H*-benzo[*b*]fluoren-10-ol / 6,7-dimethyl-11H-benzo[b]fluoren-10-ol (5h)

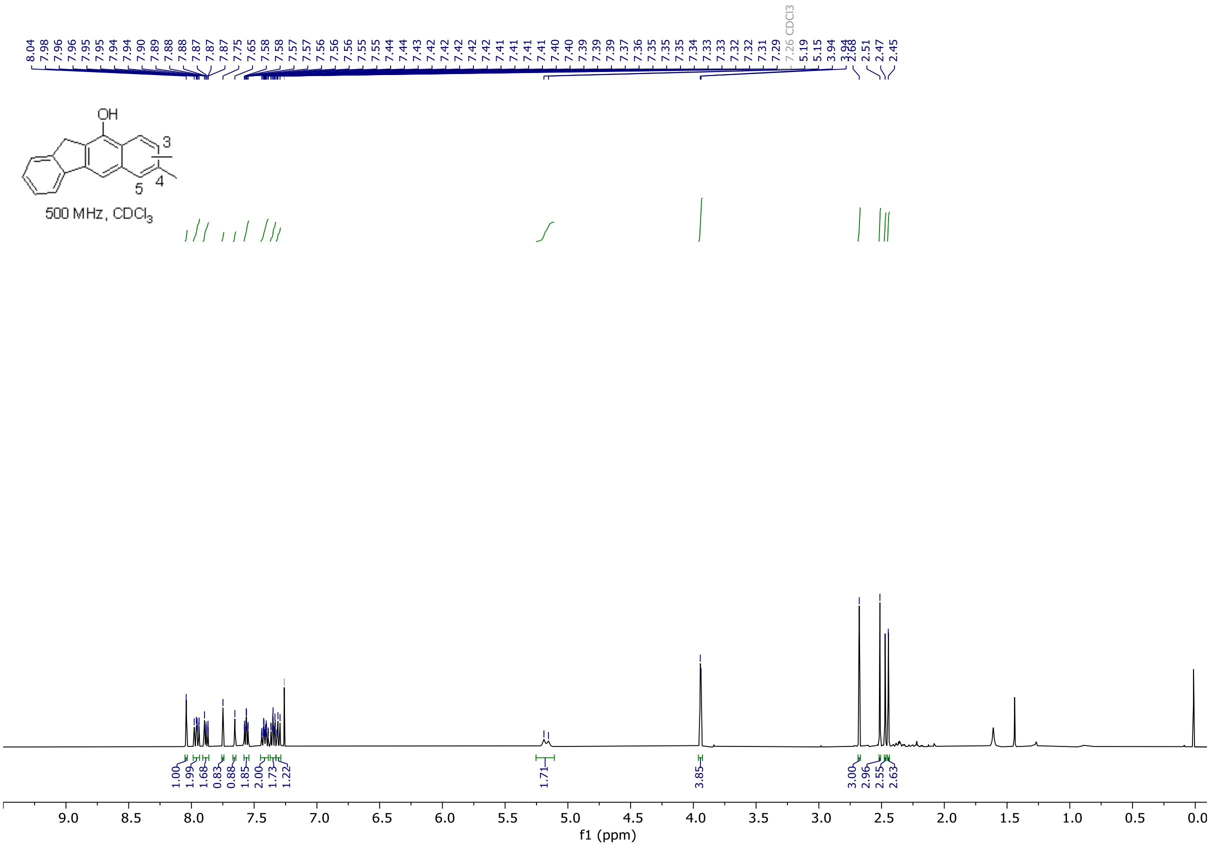

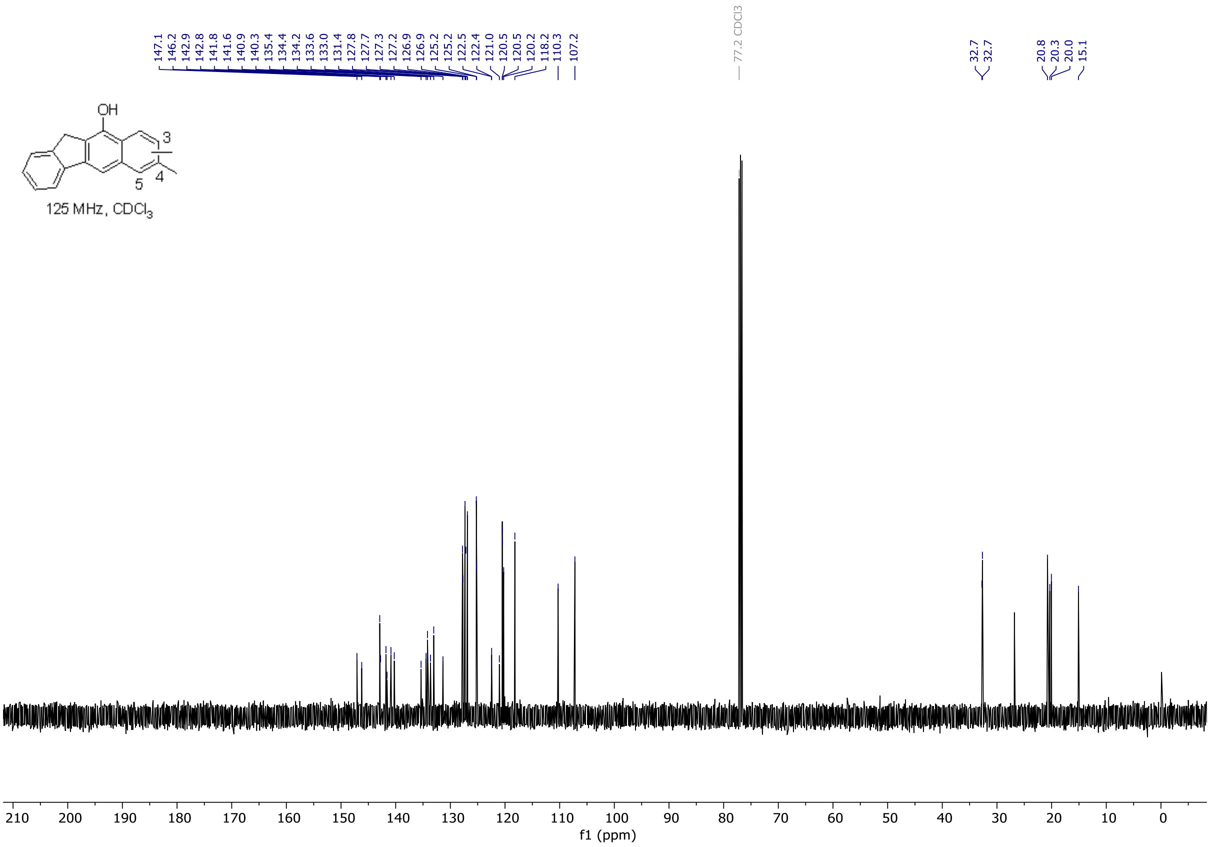

6,8-Difluoro-11*H*-benzo[*b*]fluoren-10-ol (5i)

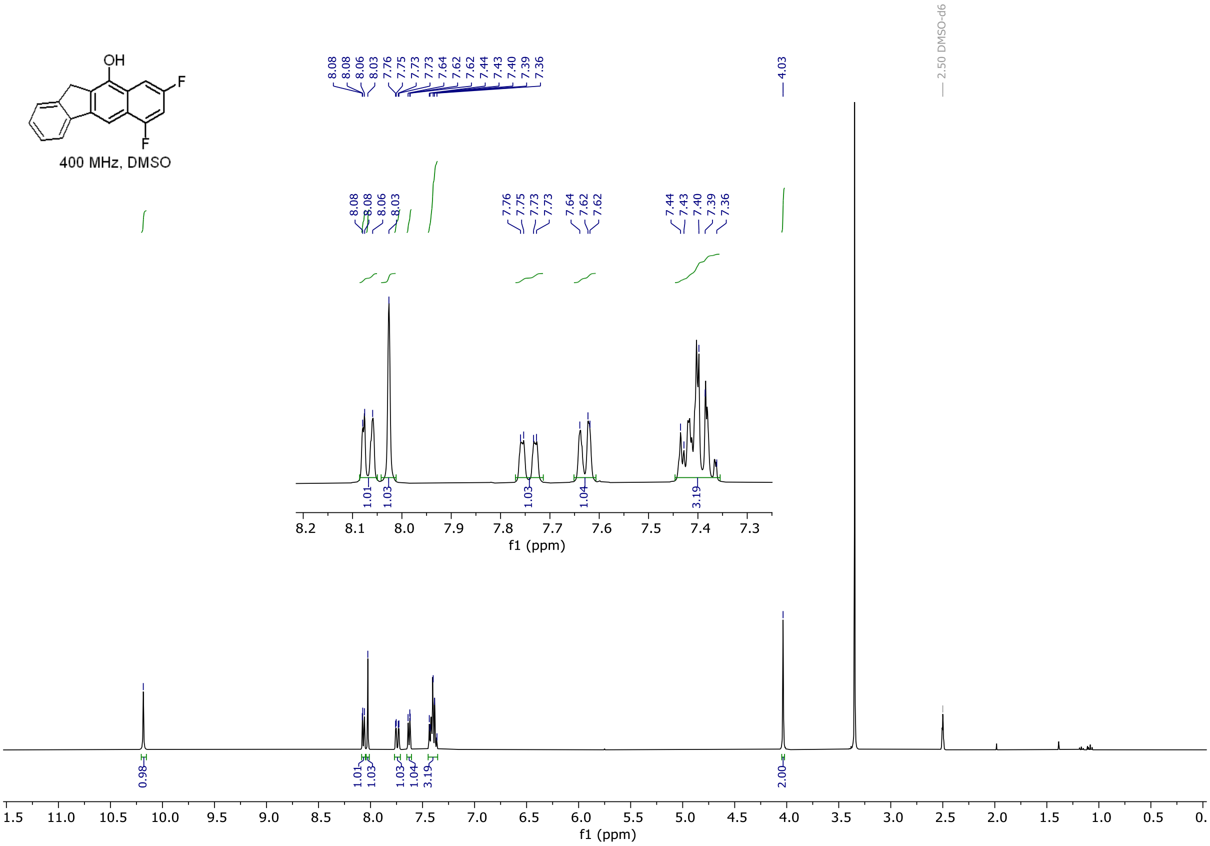

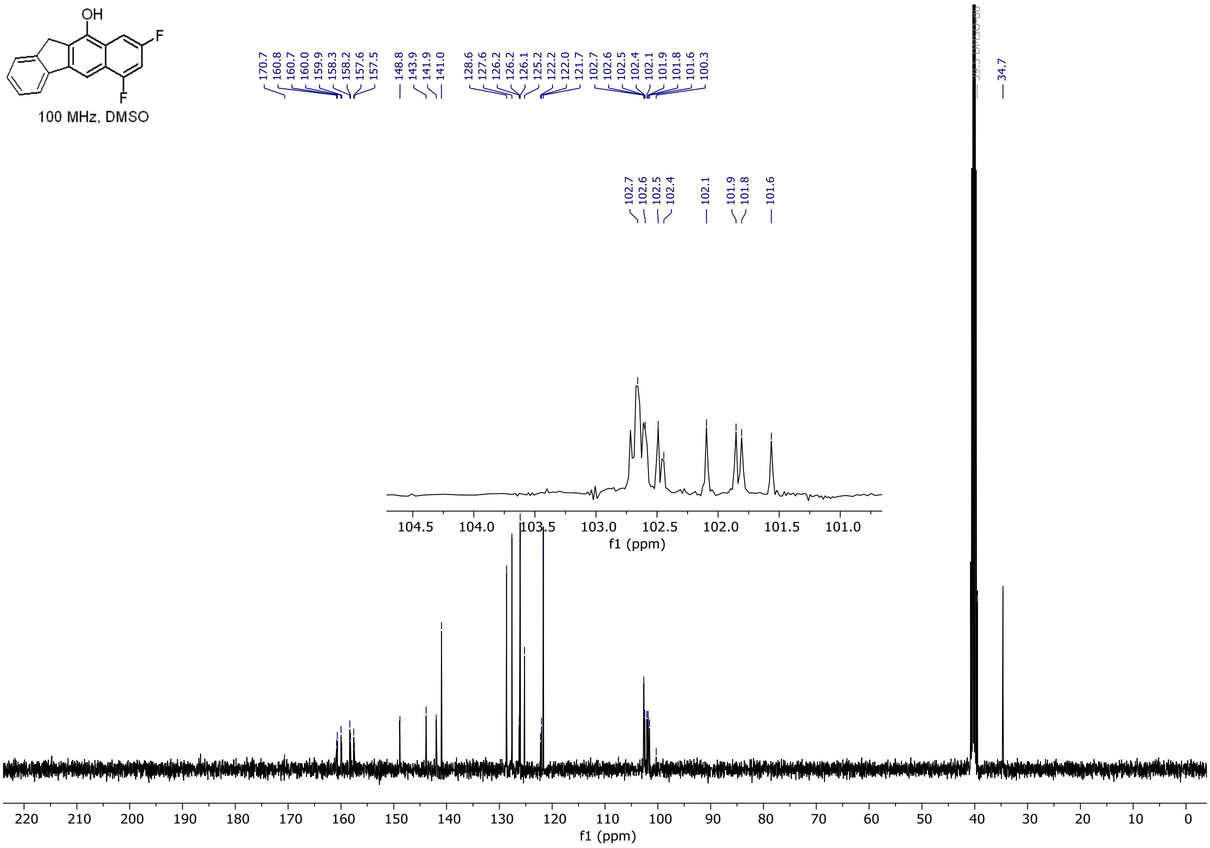

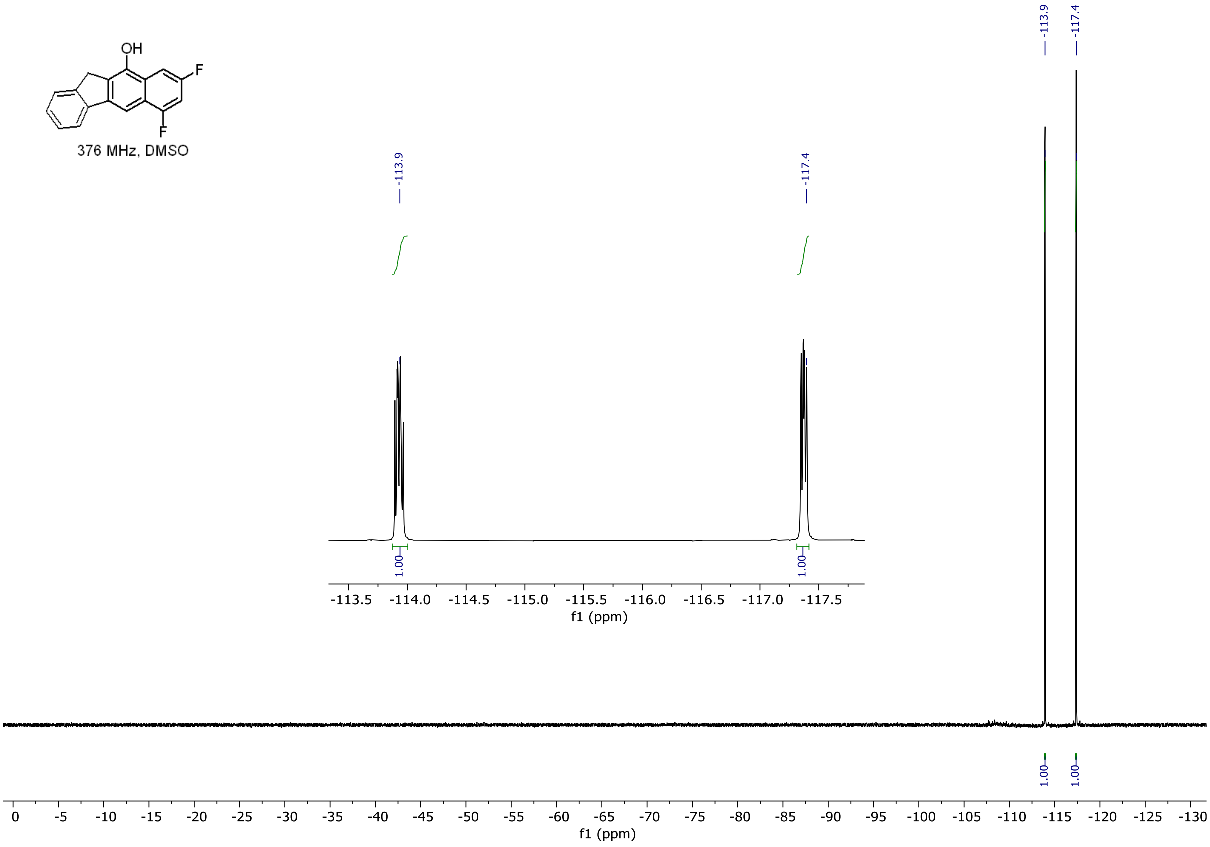

6-Fluoro-11*H*-benzo[*b*]fluoren-10-ol (5j)

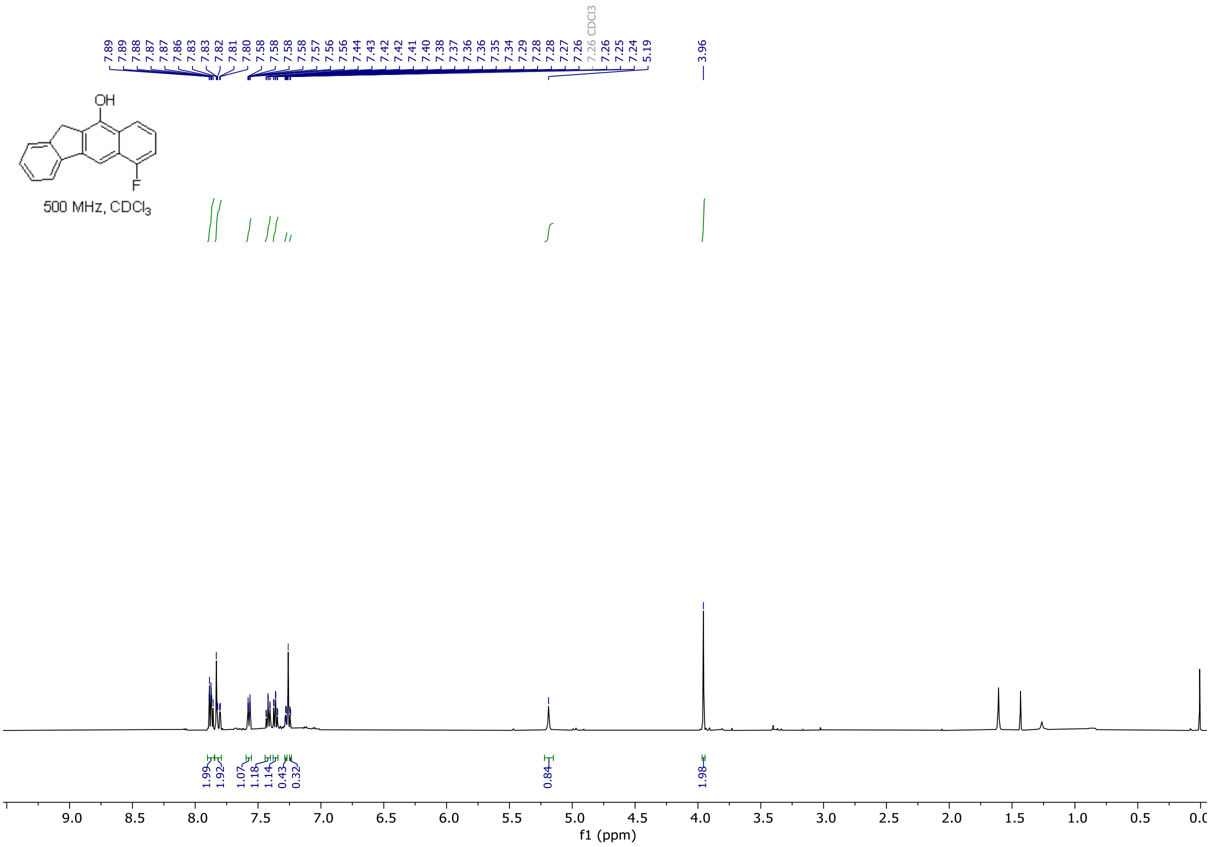

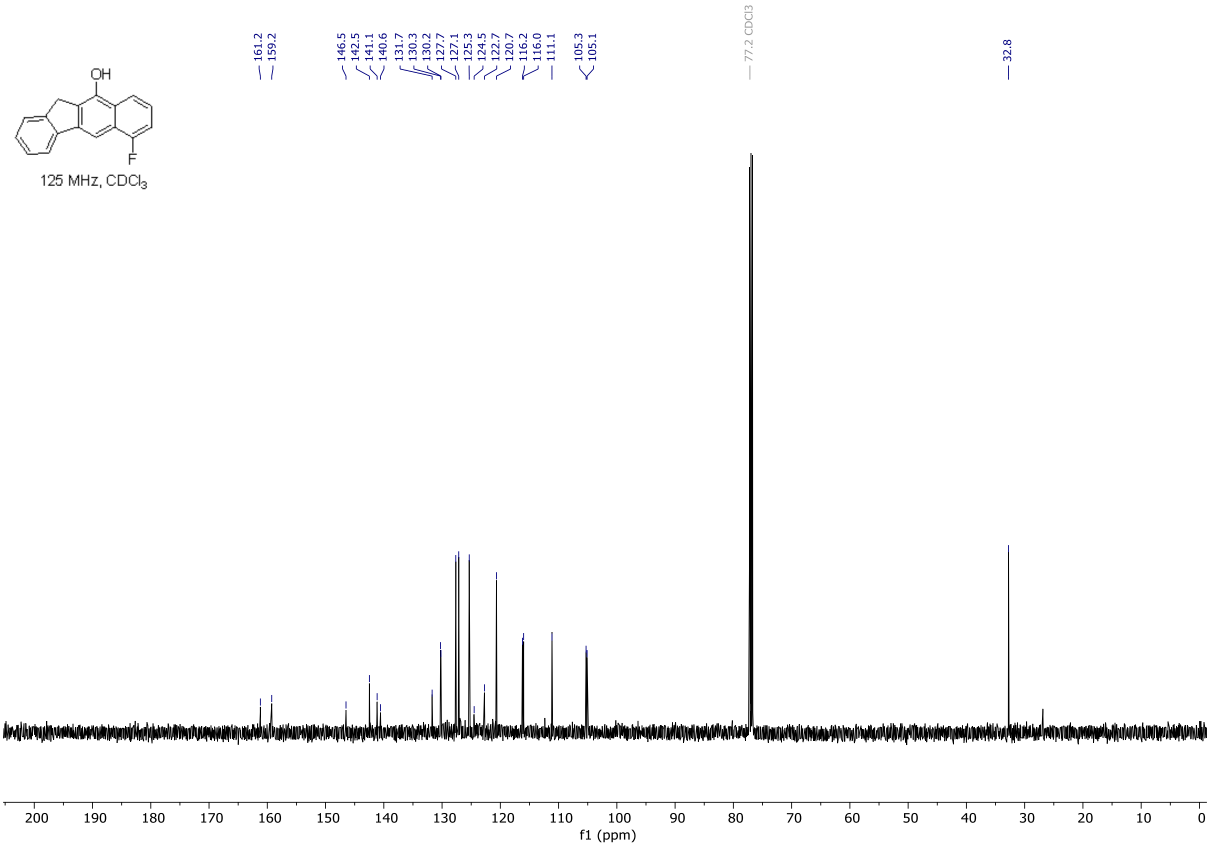
OH
F
125 MHz, CDCl3
161.2
159.2
146.5
142.5
141.1
140.6
131.7
130.3
130.2
127.7
127.1
125.3
124.5
122.7
120.7
116.2
116.0
111.1
105.3
105.1
77.2 CDCl3
32.8
200 190 180 170 160 150 140 130 120 110 100 90 80 70 60 50 40 30 20 10 0
f1 (ppm)

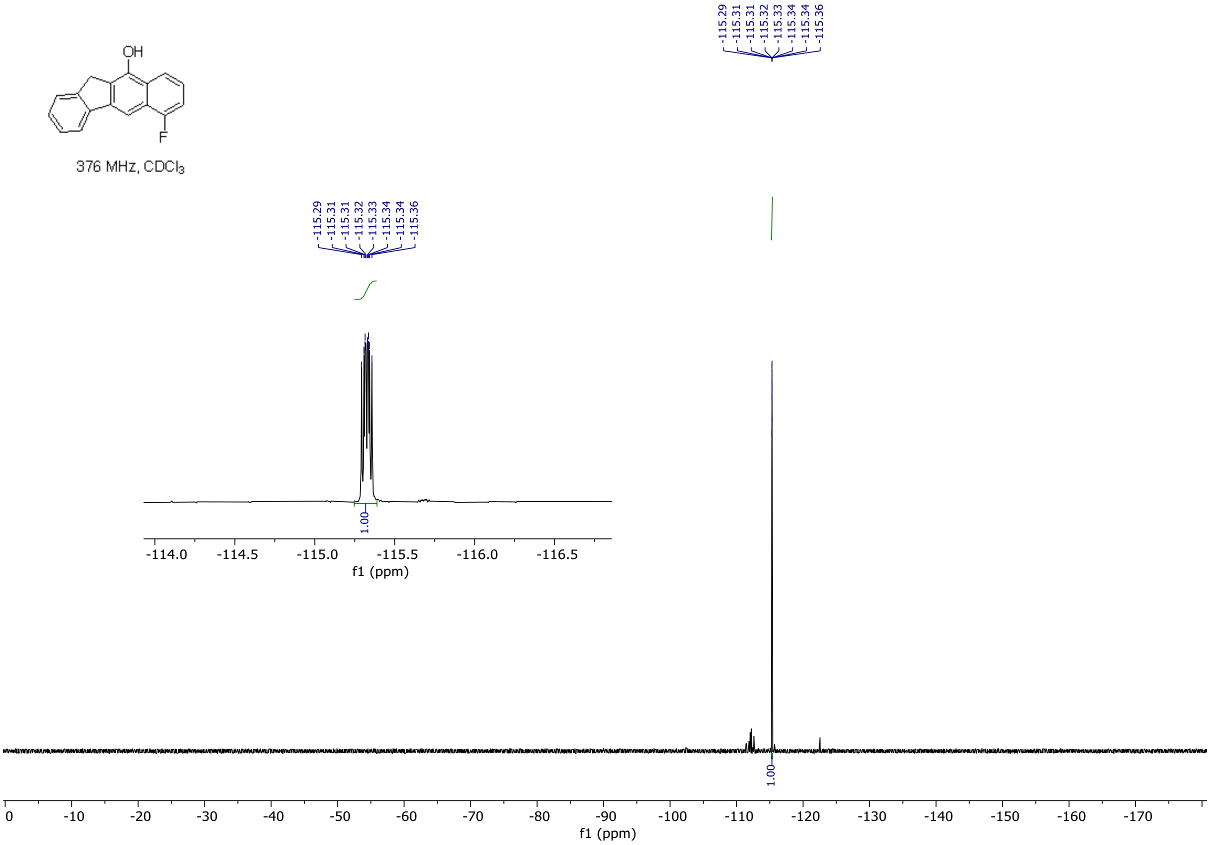
OH
F
376 MHz, CDCl3
-115.29
-115.31
-115.31
-115.32
-115.33
-115.34
-115.34
-115.36
1.00
-114.0 -114.5 -115.0 -115.5 -116.0 -116.5
f1 (ppm)
0 -10 -20 -30 -40 -50 -60 -70 -80 -90 -100 -110 -120 -130 -140 -150 -160 -170
f1 (ppm)

8-Fluoro-11*H*-benzo[*b*]fluoren-10-ol (5j’)

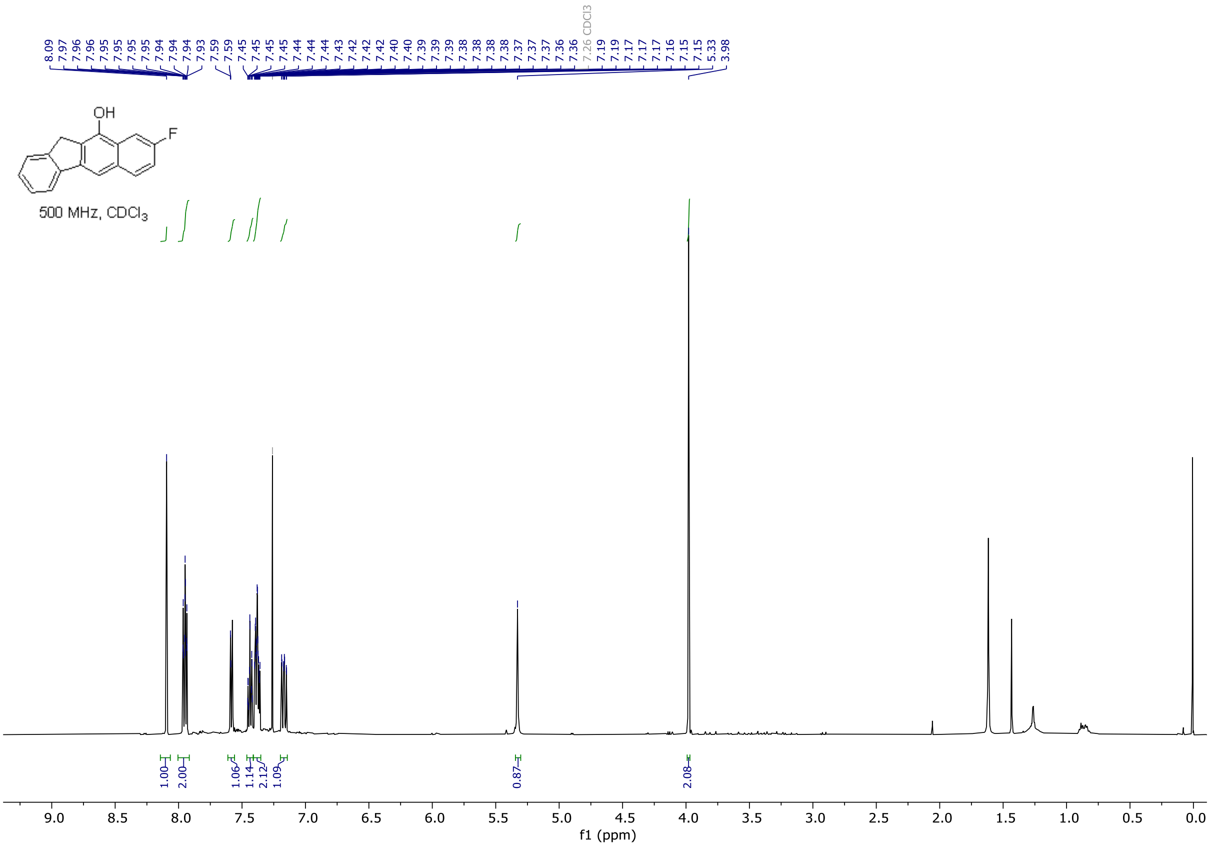

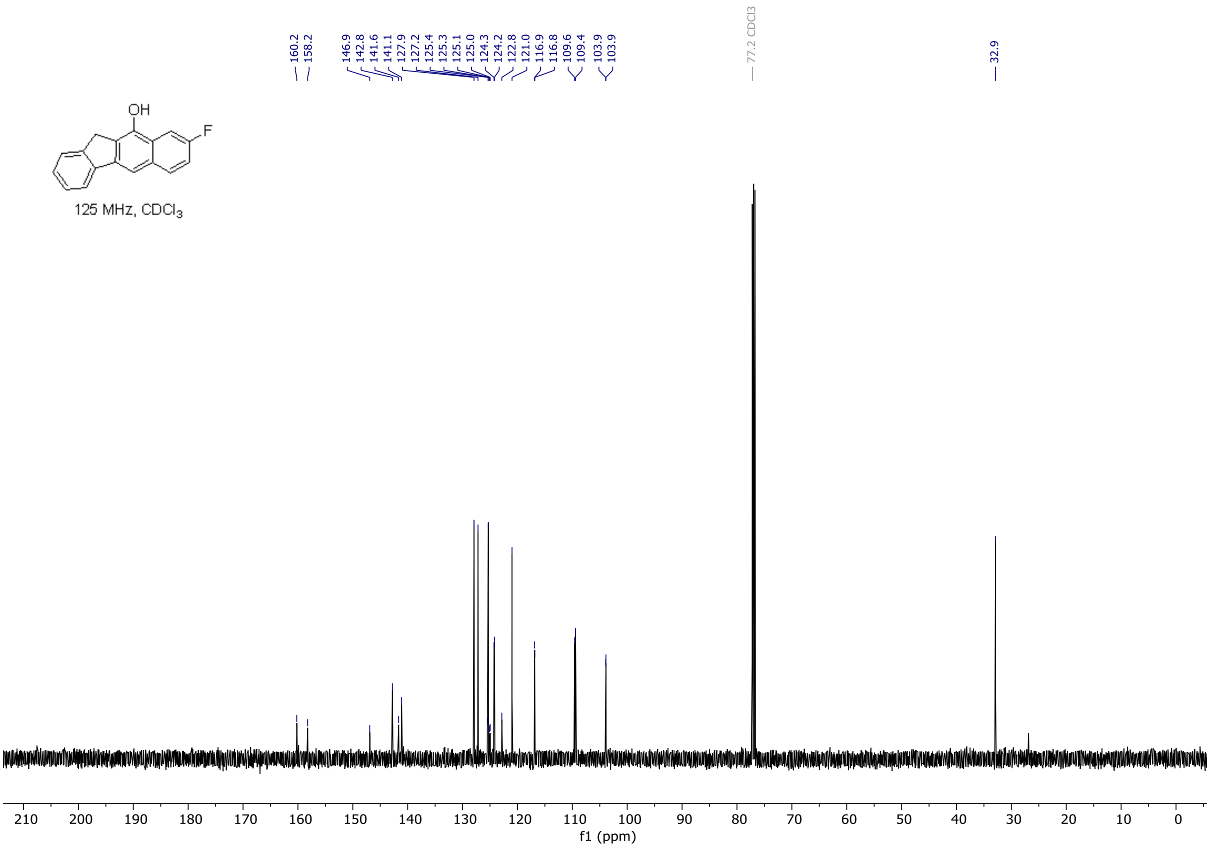

9-Fluoro-11*H*-benzo[*b*]fluoren-10-ol (5k)

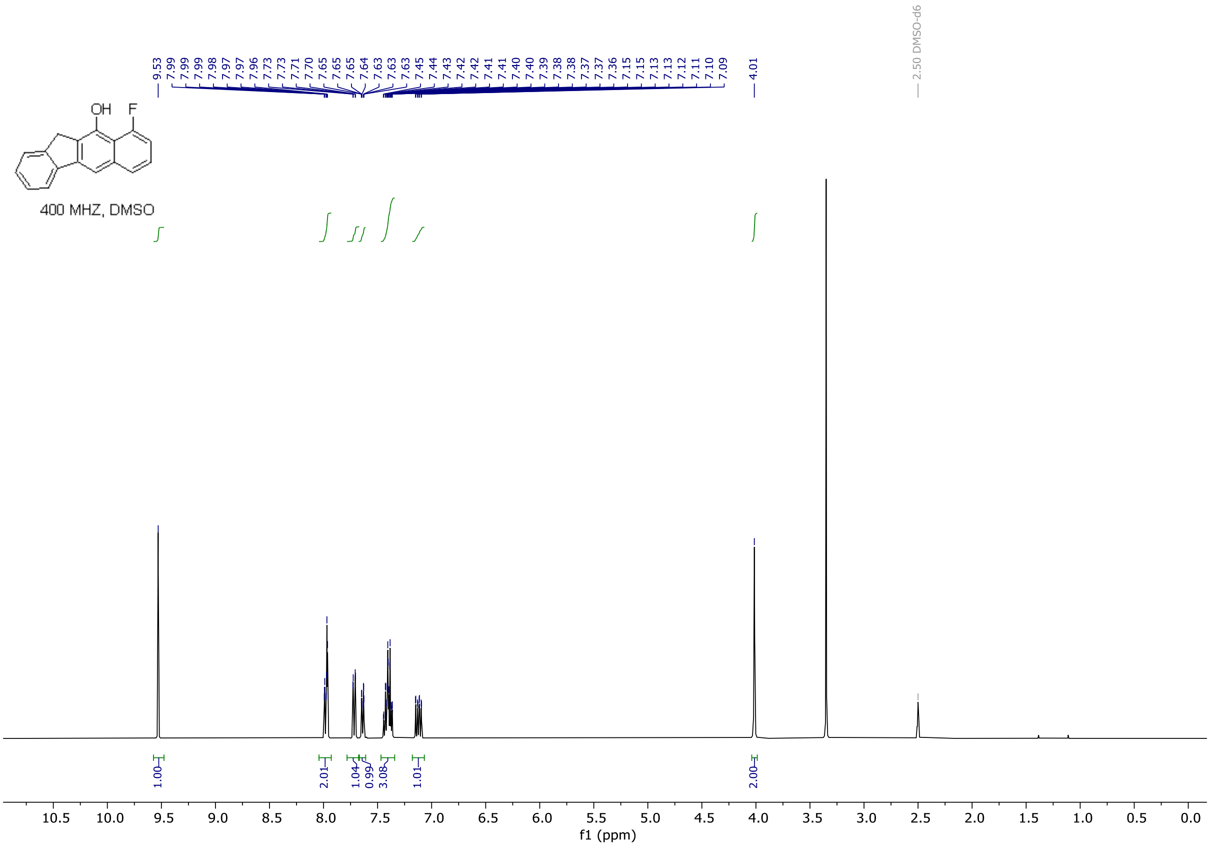

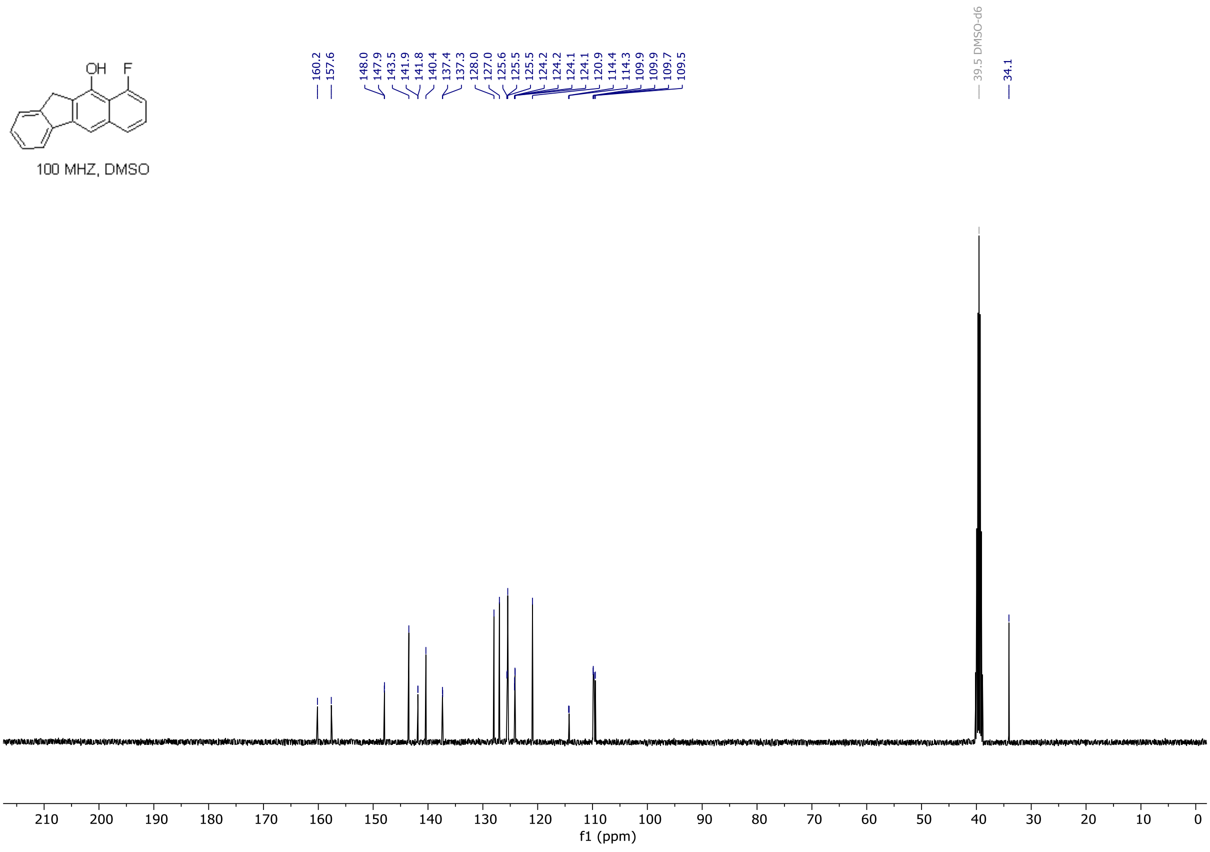

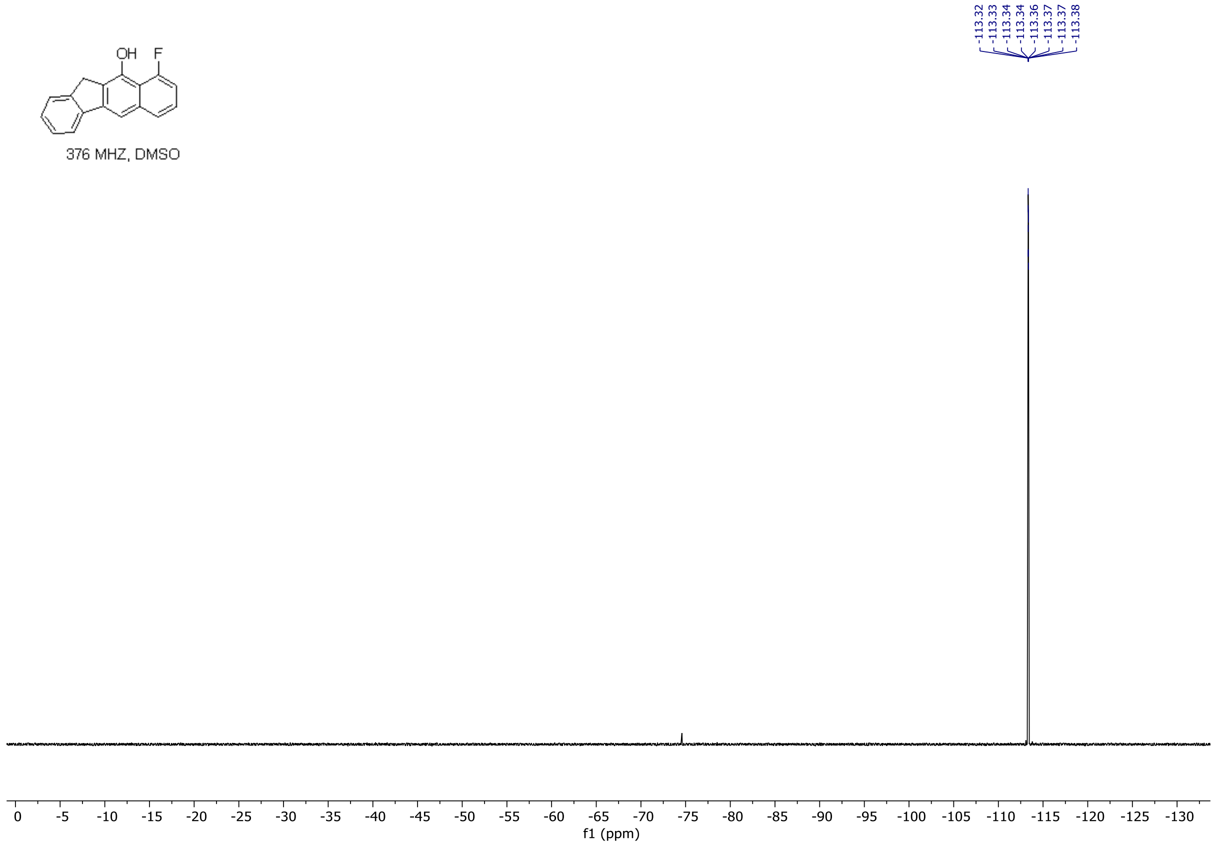

9-Methyl-11*H*-benzo[*b*]fluoren-10-ol (5l)

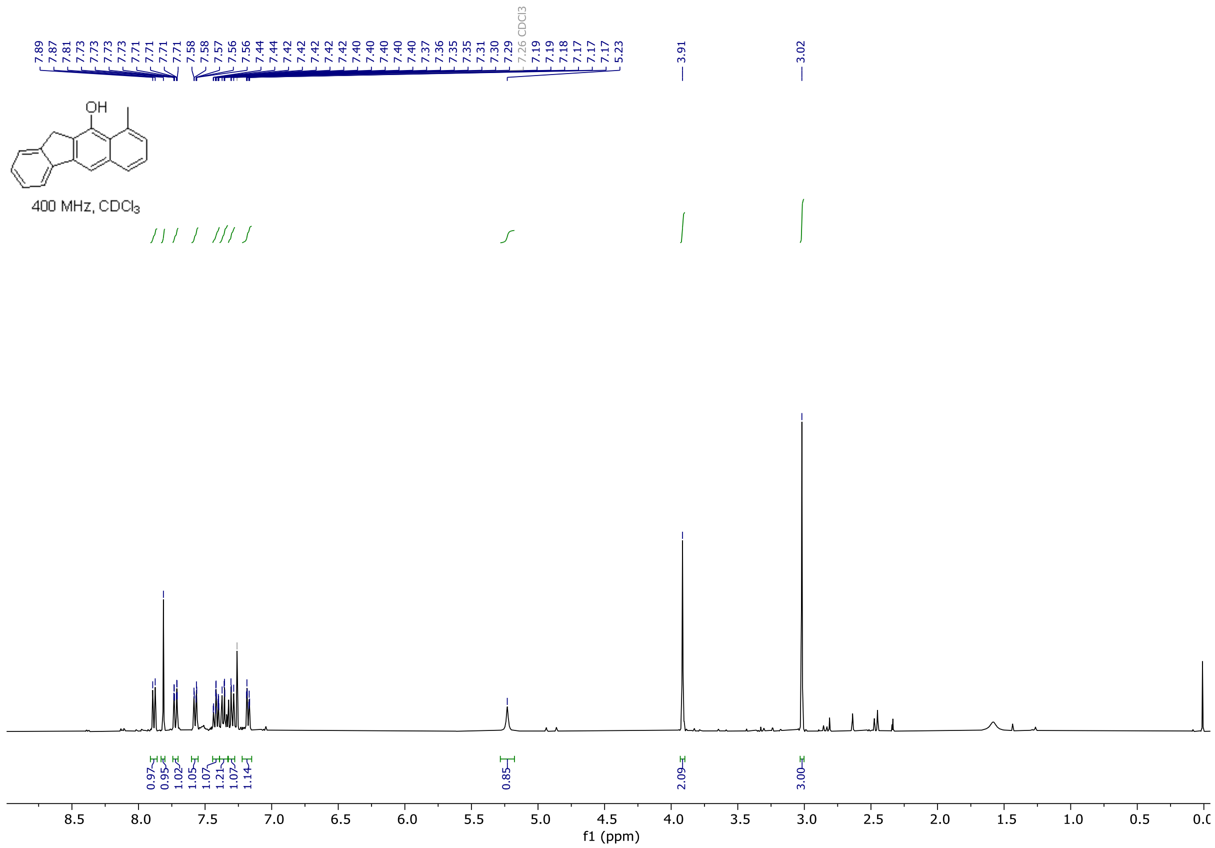

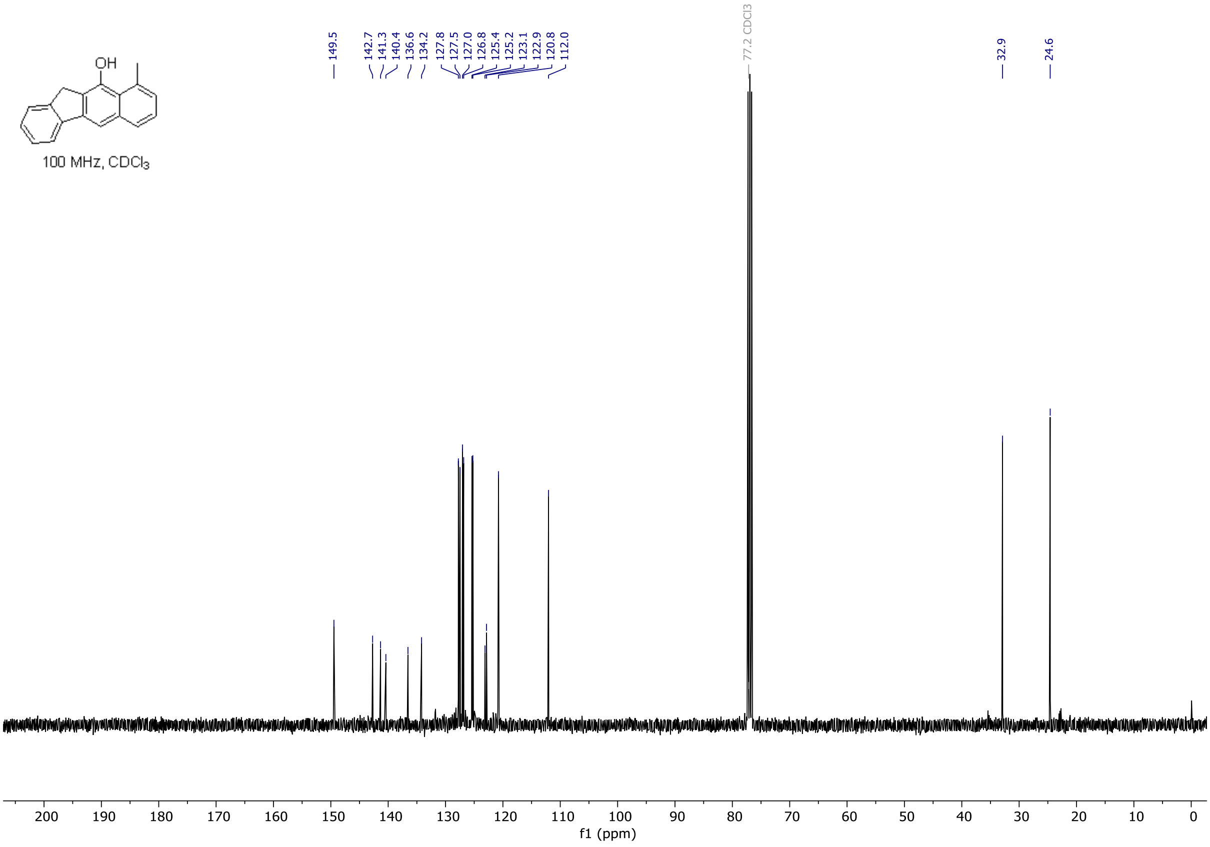

9-Fluoro-7-methoxy-11*H*-benzo[*b*]fluoren-10-ol (5m)

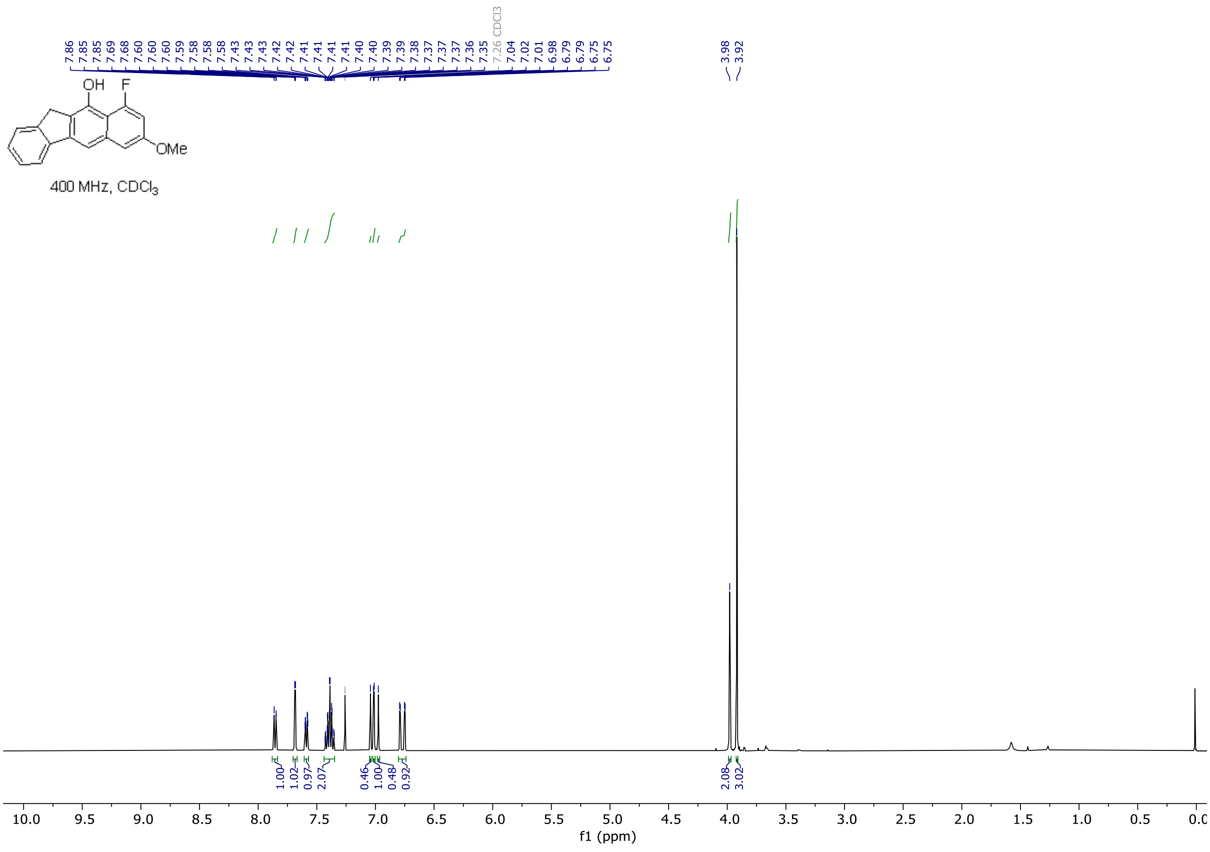

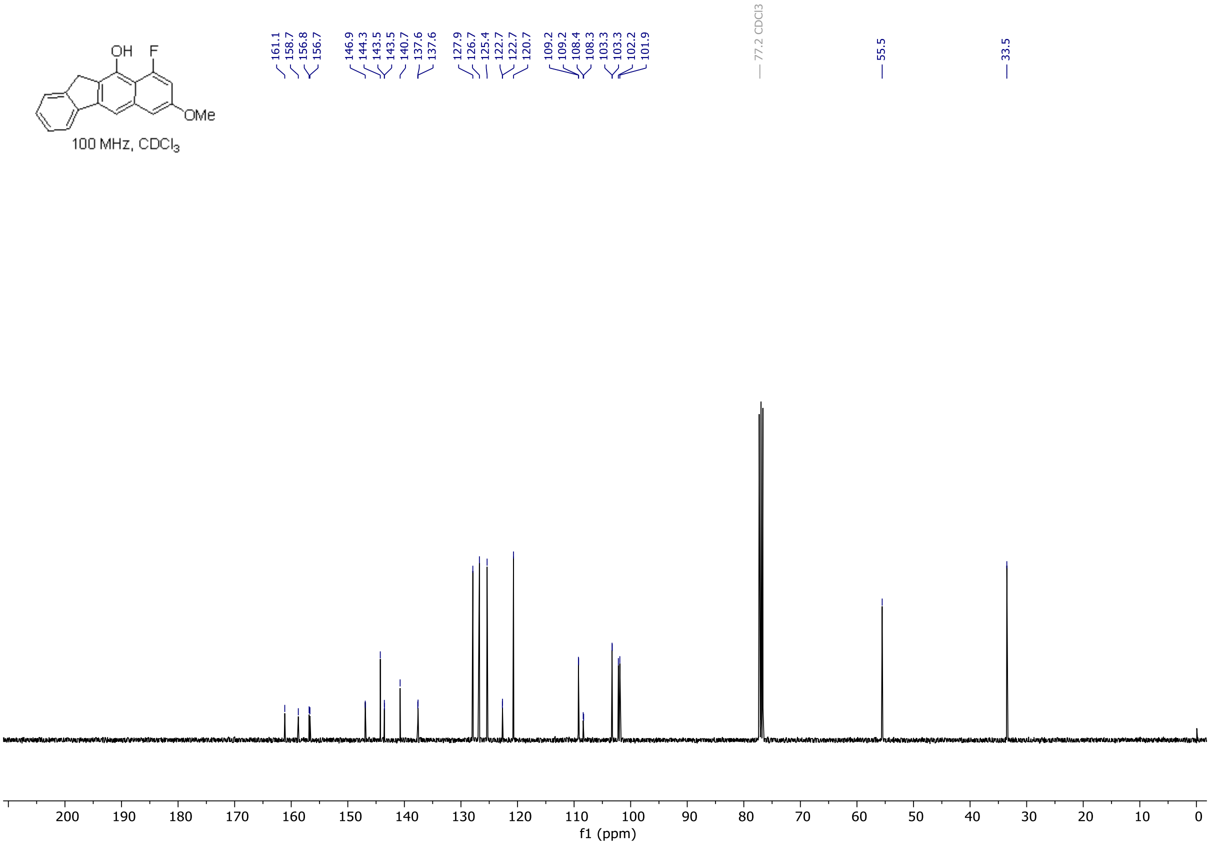
OH
F
OMe
100 MHz, CDCl3
161.1
158.7
156.8
156.7
146.9
144.3
143.5
143.5
140.7
137.6
137.6
127.9
126.7
125.4
122.7
122.7
120.7
109.2
109.2
108.4
108.3
103.3
103.3
102.2
101.9
77.2 CDCl3
55.5
33.5
f1 (ppm)

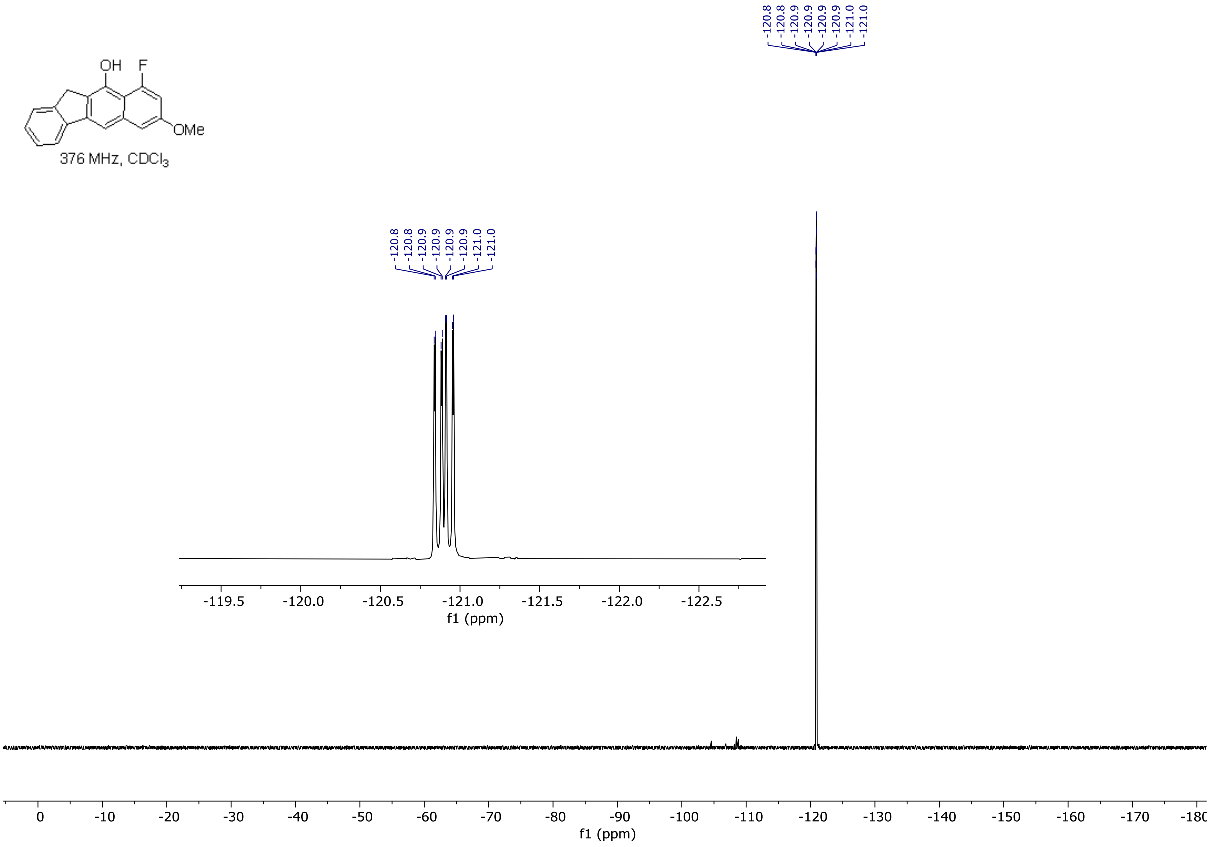
OH
F
OMe
376 MHz, CDCl3
-120.8
-120.8
-120.9
-120.9
-120.9
-120.9
-121.0
-121.0
f1 (ppm)

7-Fluoro-3-methyl-11*H*-benzo[*b*]fluoren-10-ol (5n)

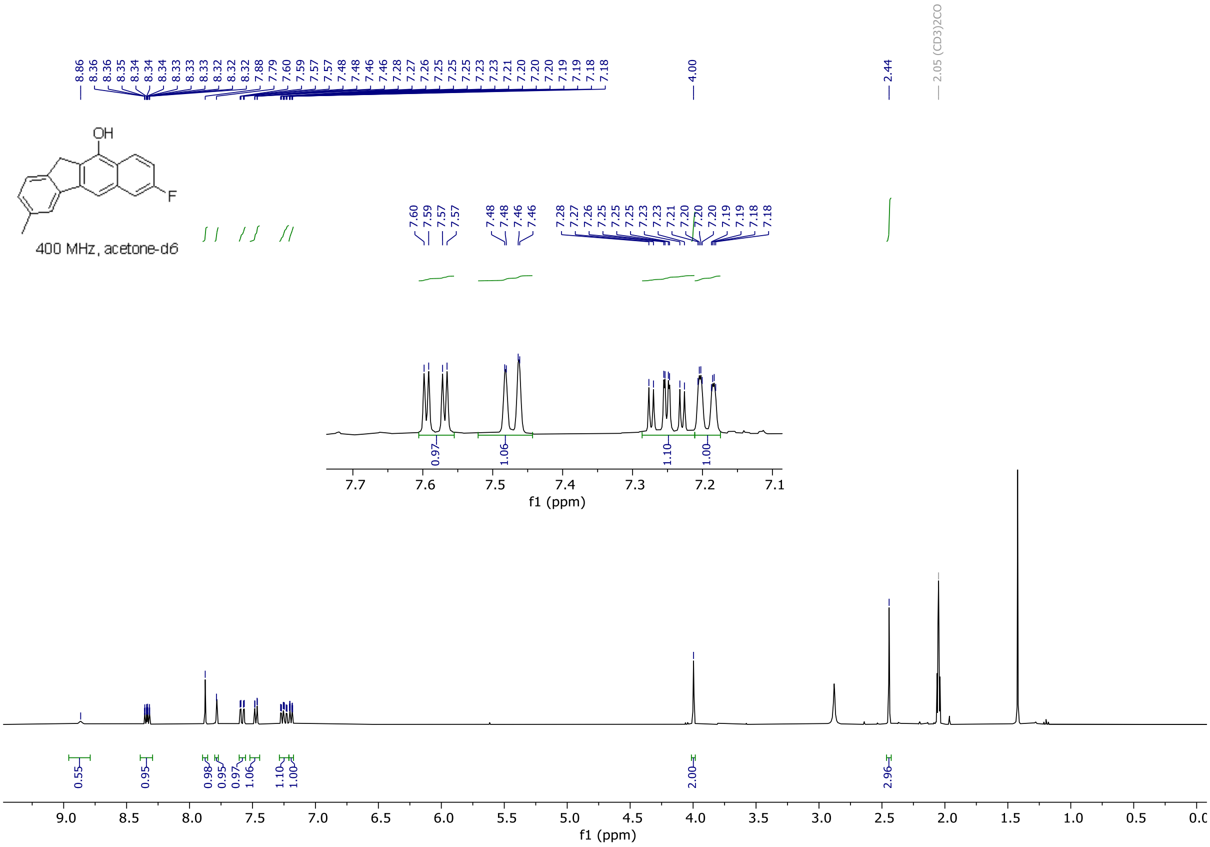

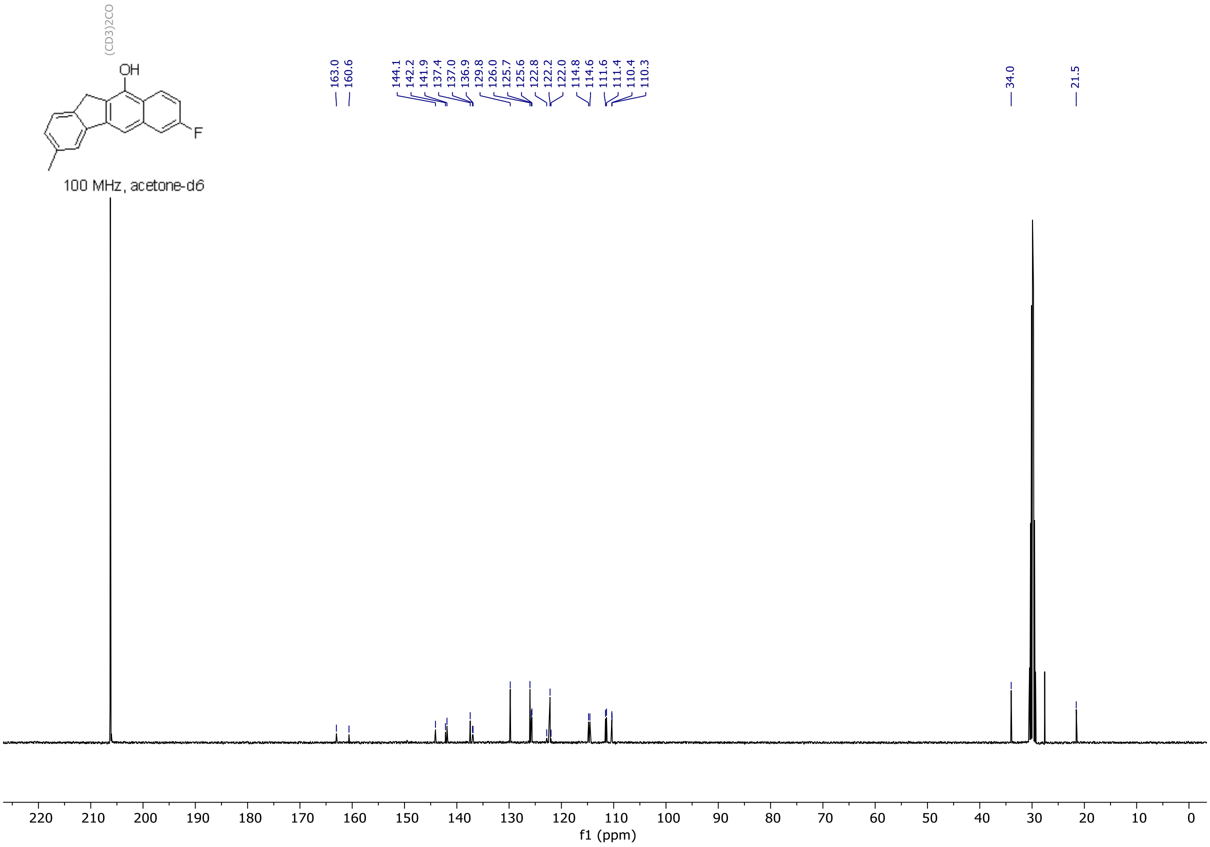

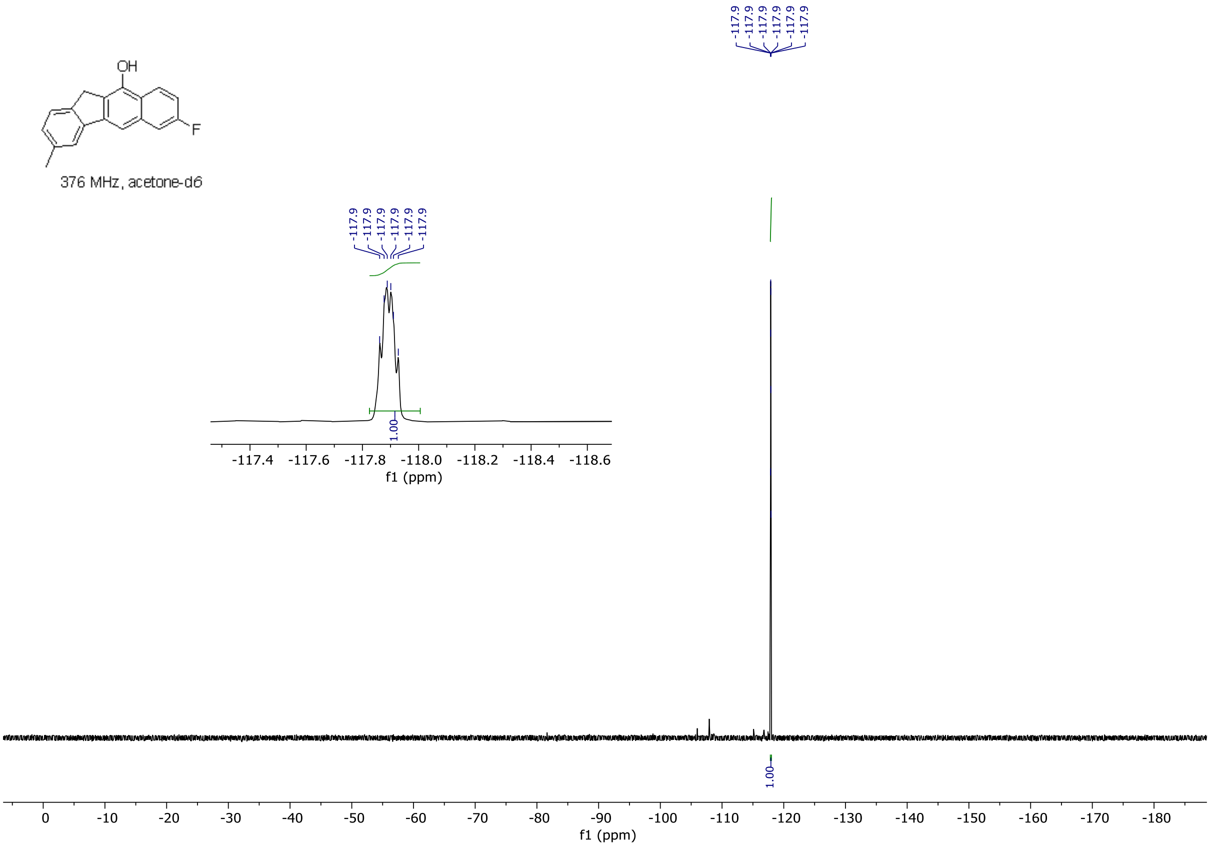

3-Fluoro-7-methyl-11*H*-benzo[*b*]fluoren-10-ol (5o)

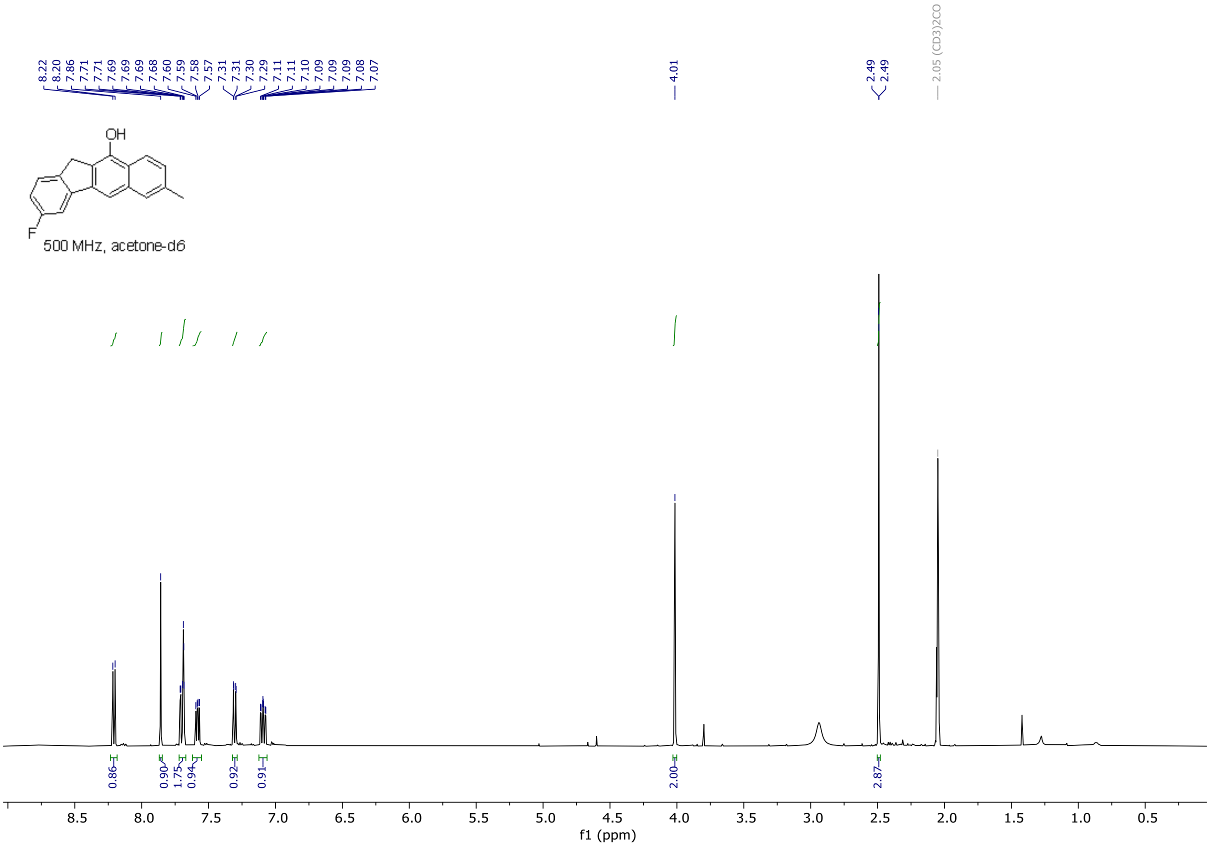

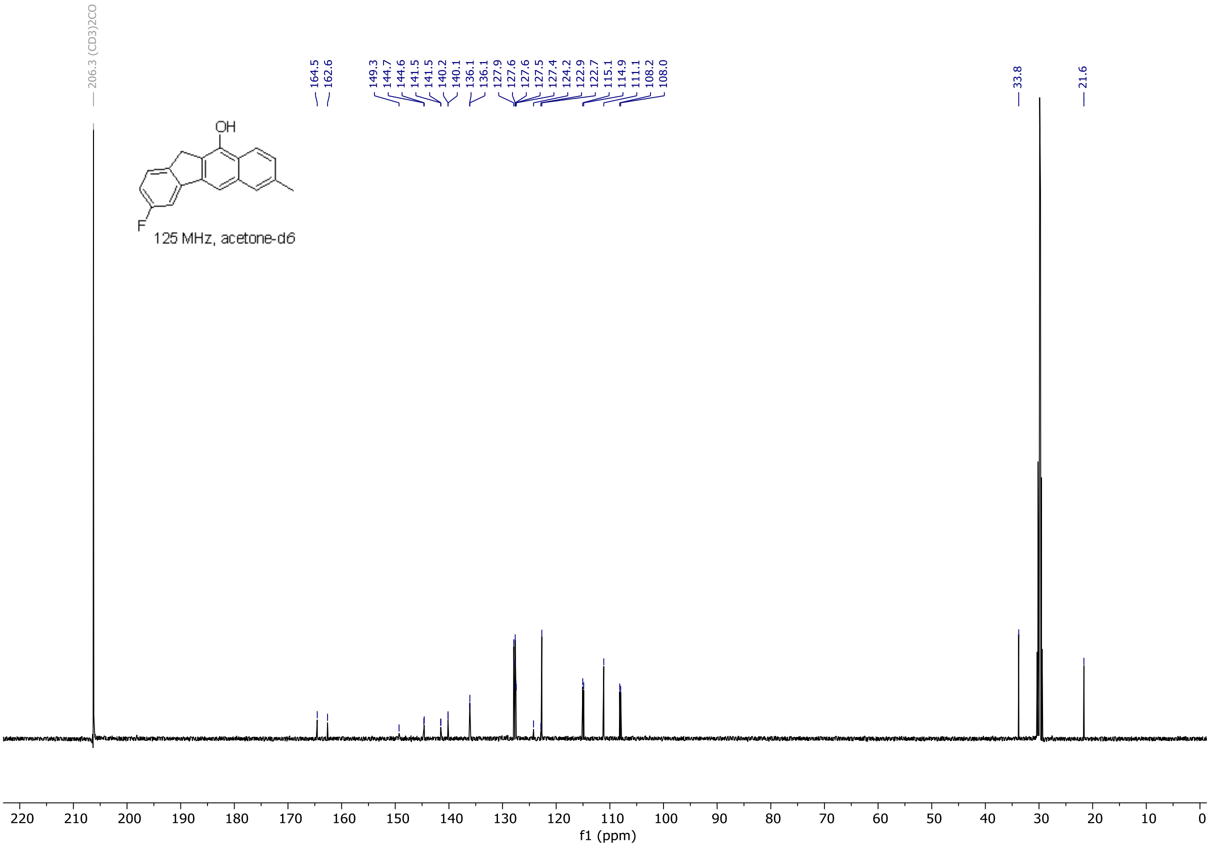
206.3 (CD3)2CO
164.5
162.6
149.3
144.7
144.6
141.5
141.5
140.2
140.1
136.1
136.1
127.9
127.6
127.6
127.5
127.4
124.2
122.9
122.7
115.1
114.9
111.1
108.2
108.0
33.8
21.6
OH
F
125 MHz, acetone-d6
f1 (ppm)

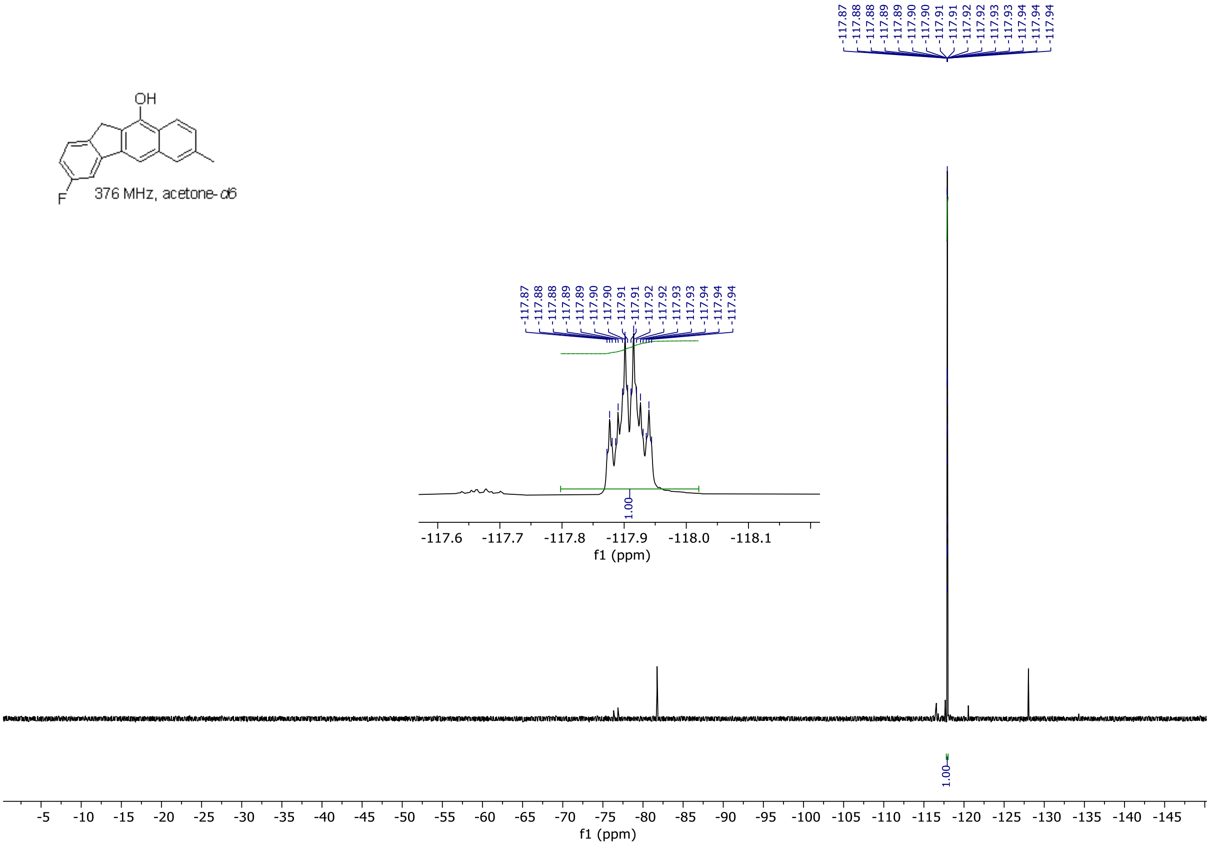
-117.87
-117.88
-117.88
-117.89
-117.89
-117.90
-117.90
-117.91
-117.91
-117.92
-117.92
-117.93
-117.93
-117.94
-117.94
-117.94
OH
F
376 MHz, acetone-d6
1.00
f1 (ppm)

3-Methyl-11*H*-benzo[*b*]fluoren-10-ol (5p)

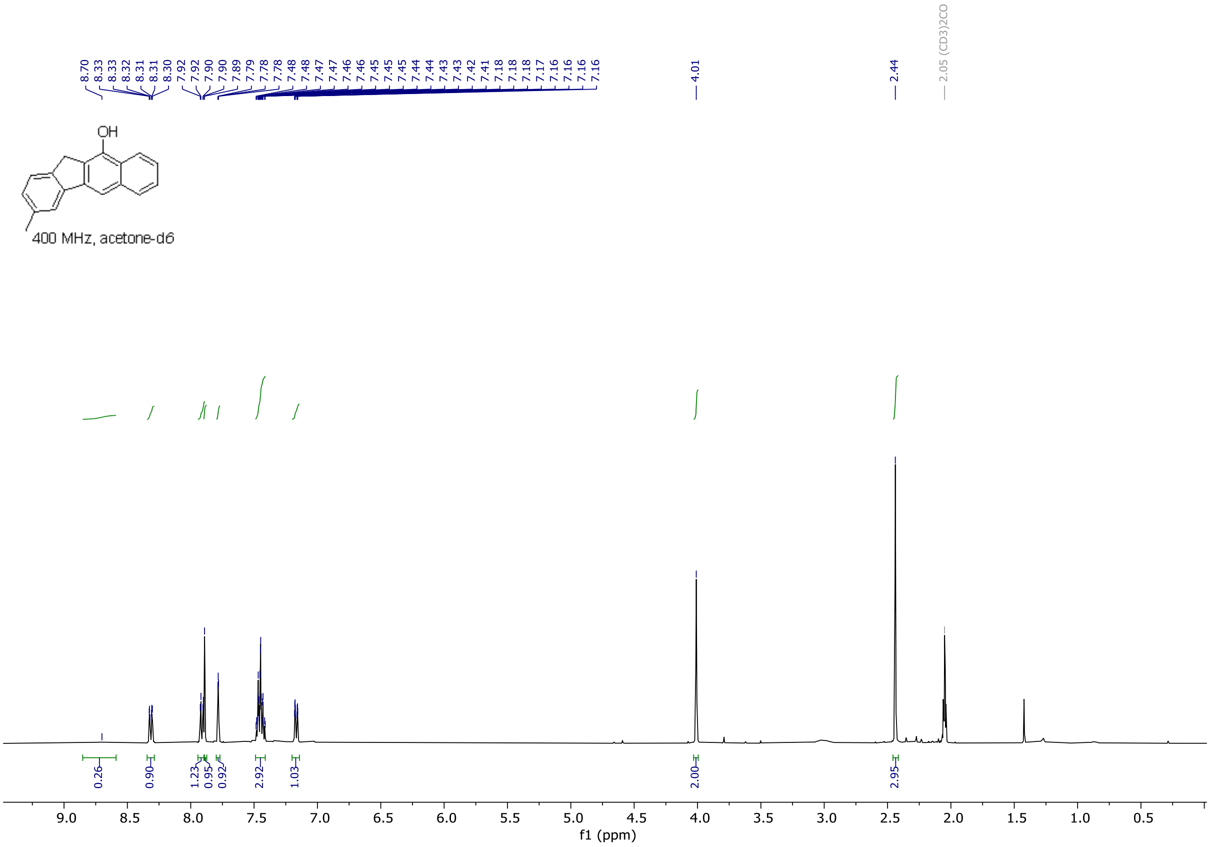

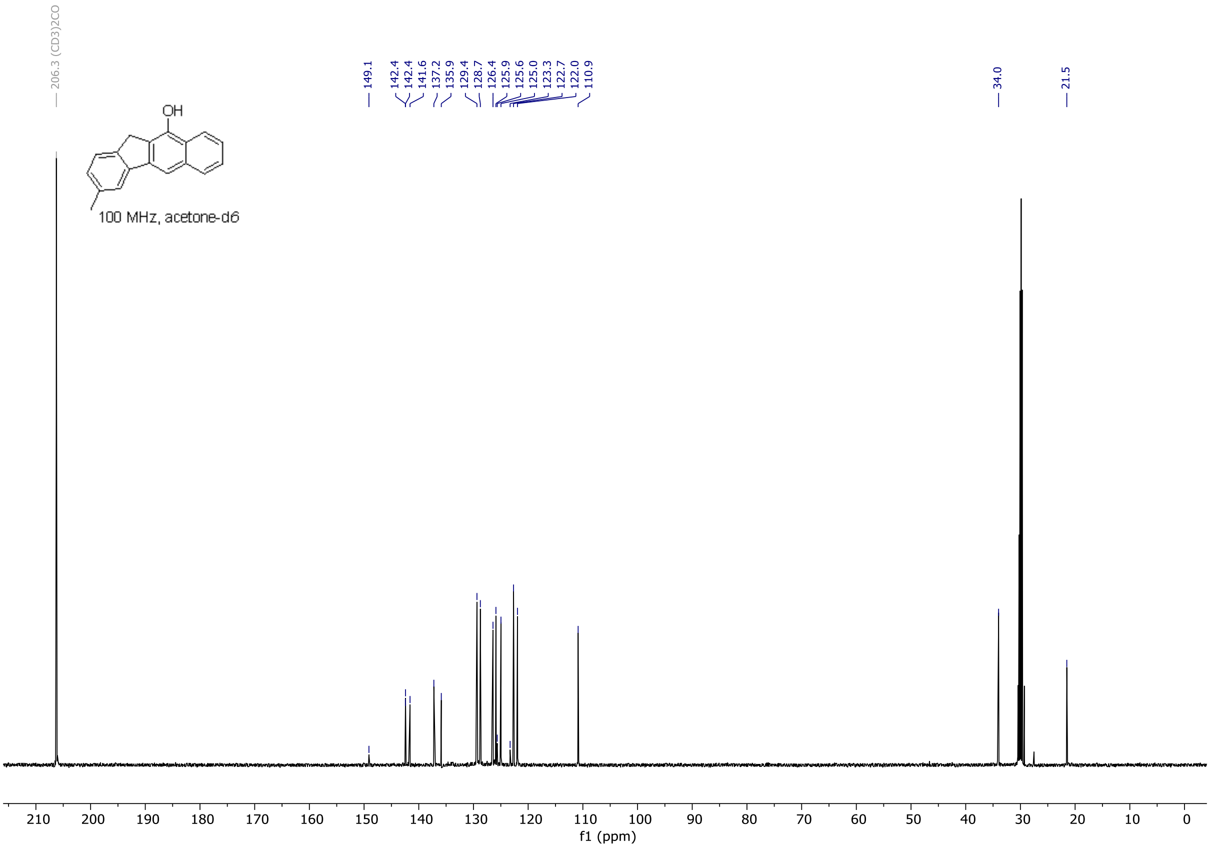

3,7-Difluoro-11*H*-benzo[*b*]fluoren-10-ol (5q)

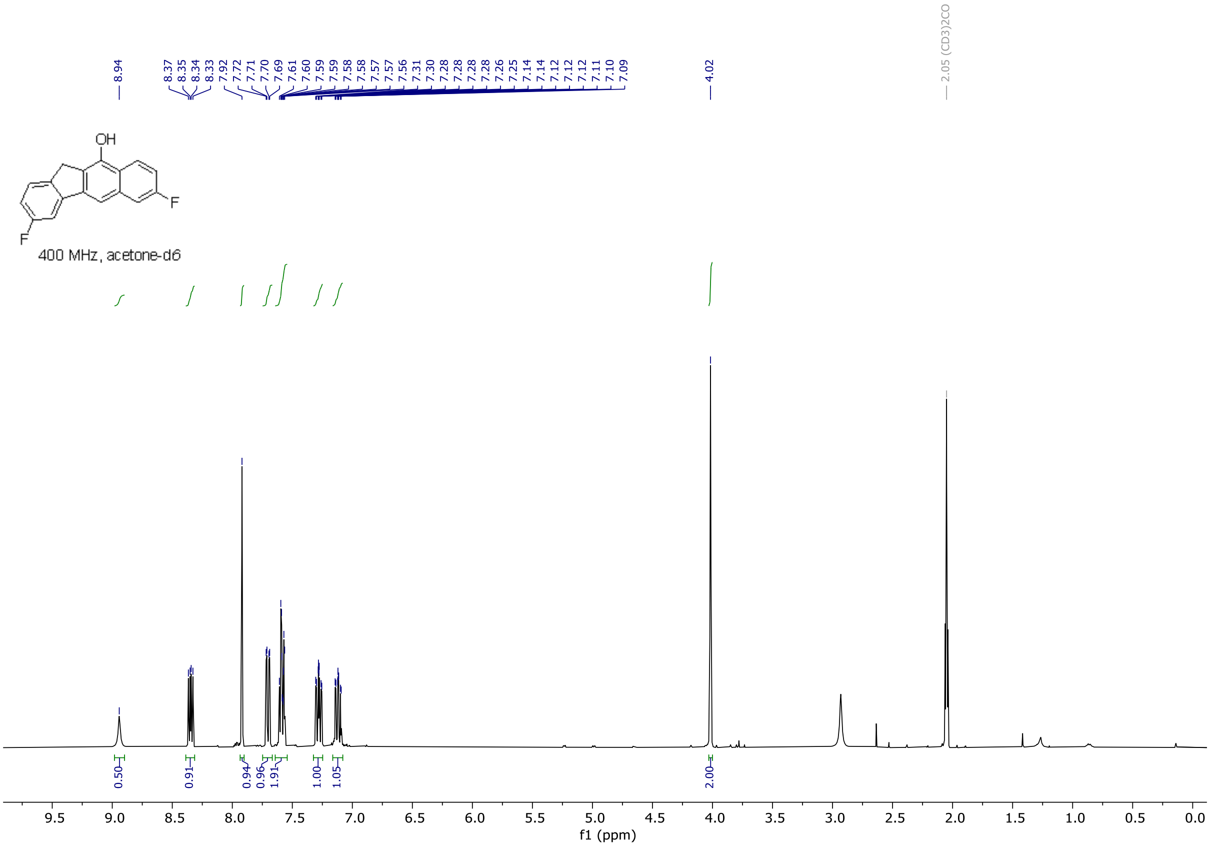

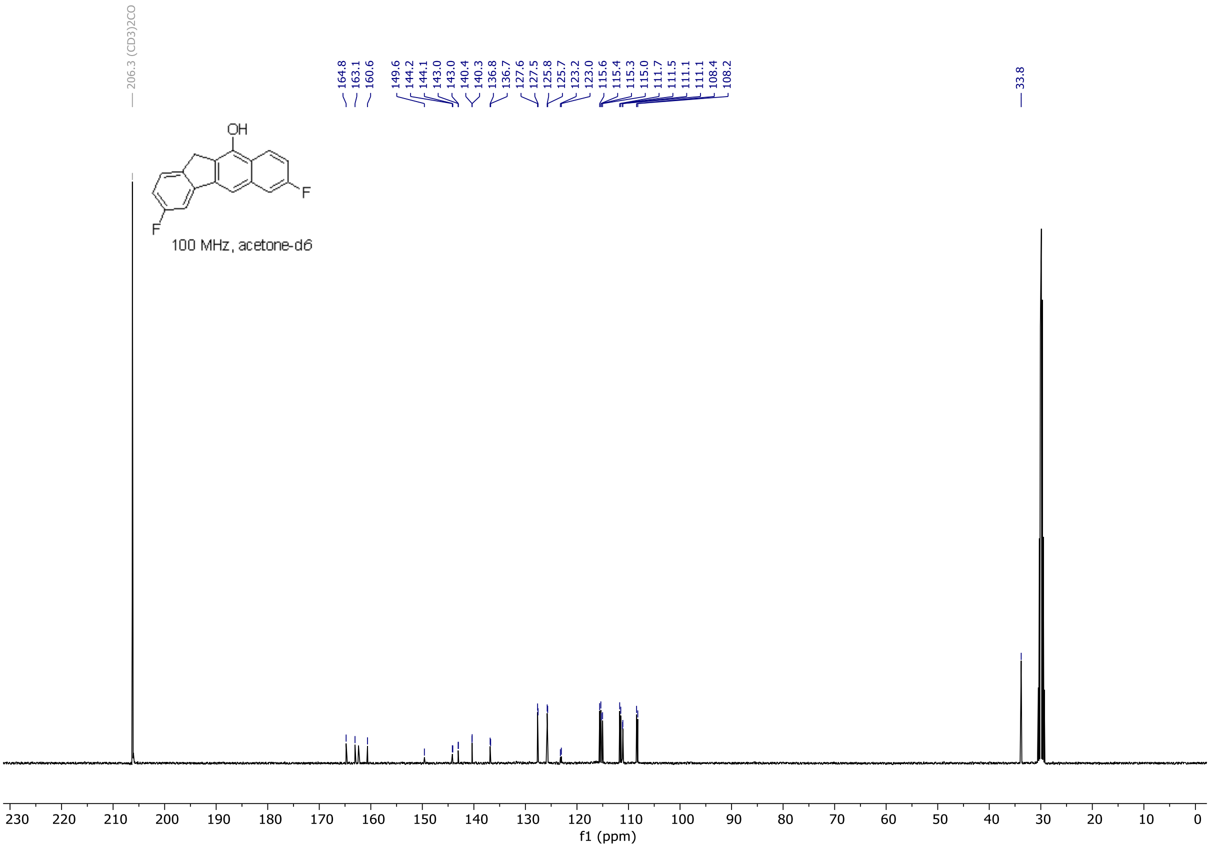

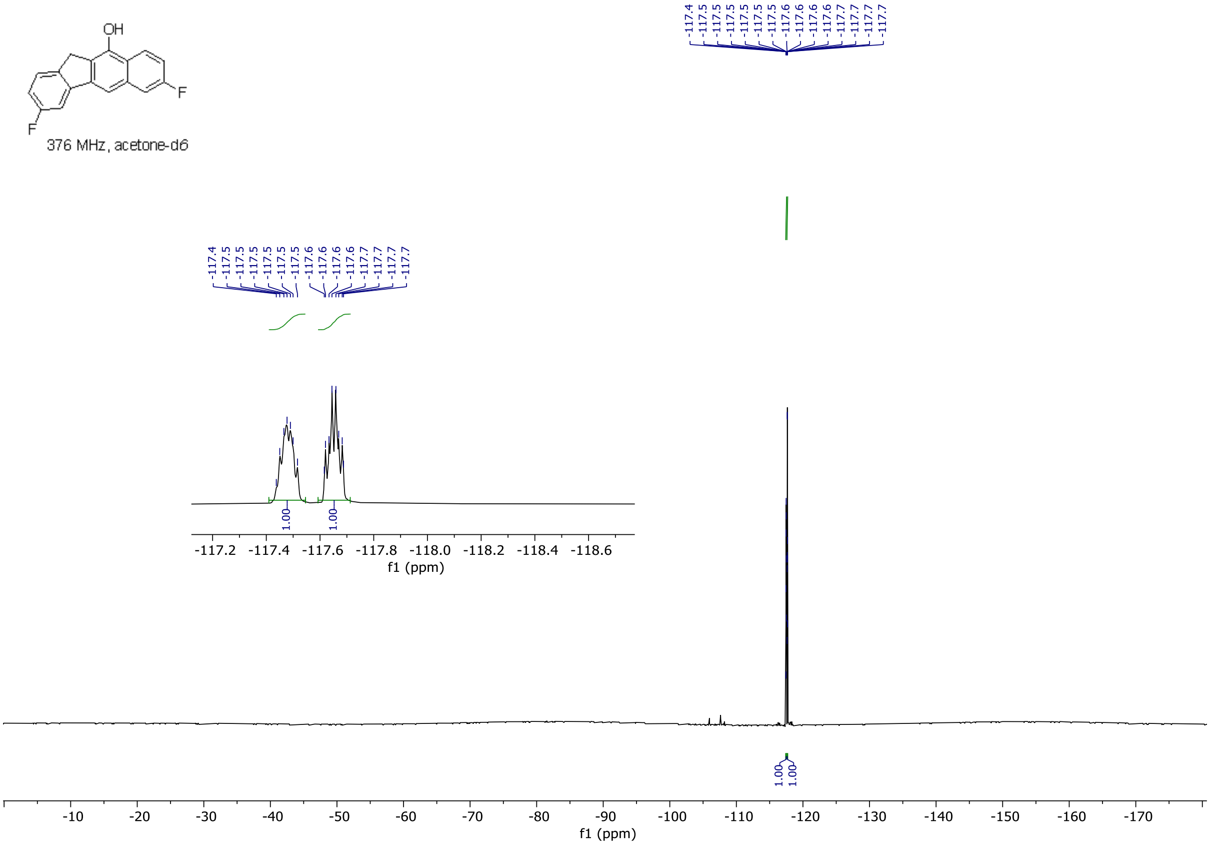

3,7-Dimethyl-11*H*-benzo[*b*]fluoren-10-ol (5r)

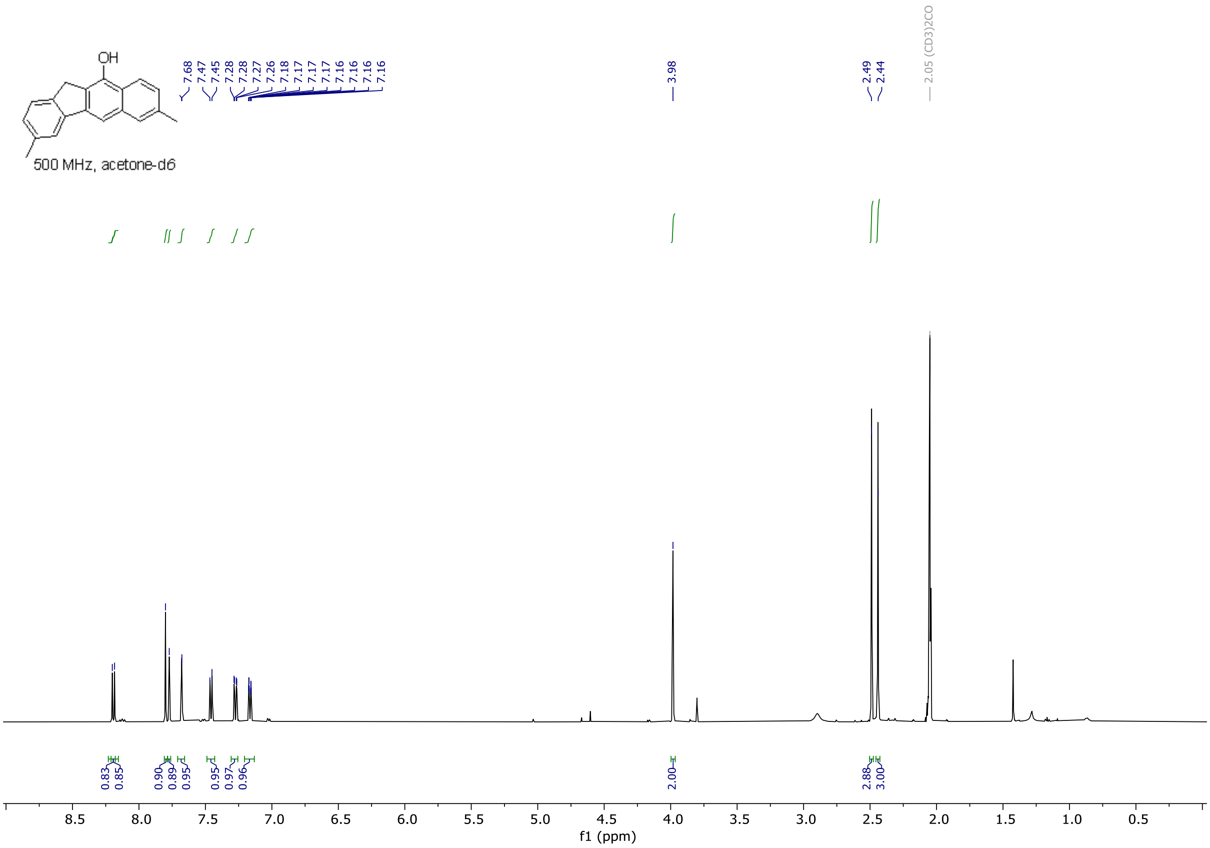

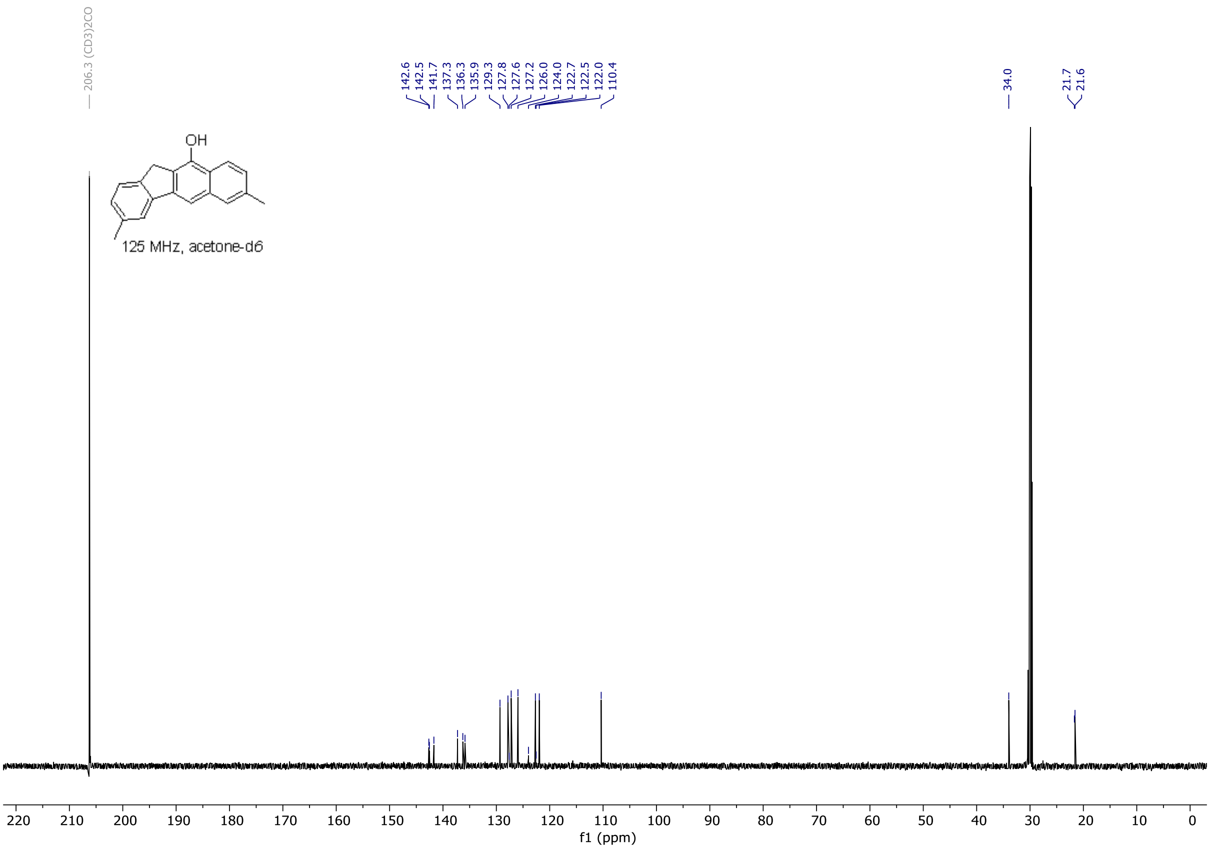

6-Fluoro-11*H*-benzo[*b*]fluoren-10-ol (5s)

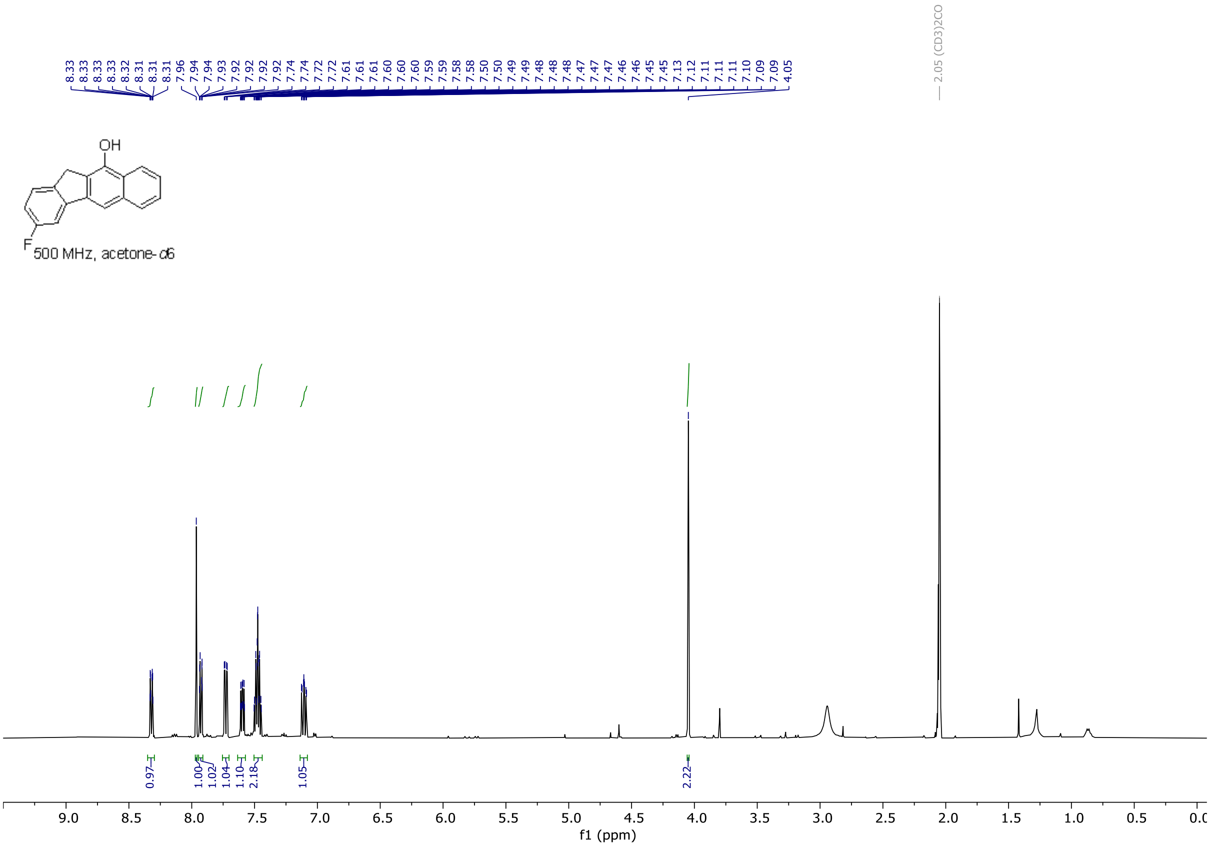

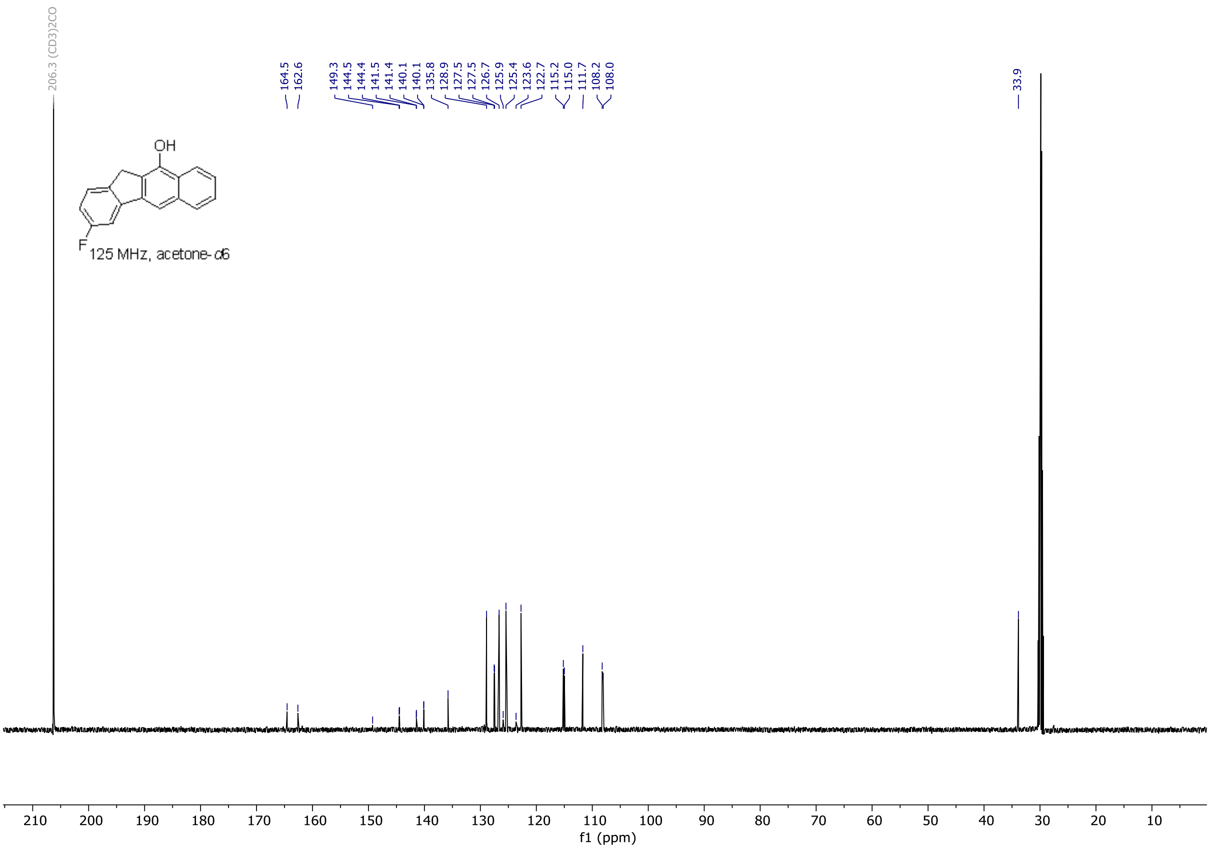
206.3 (CD3)2CO
164.5
162.6
149.3
144.5
144.4
141.5
141.4
140.1
140.1
135.8
128.9
127.5
127.5
126.7
125.9
125.4
123.6
122.7
115.2
115.0
111.7
108.2
108.0
33.9
OH
F
125 MHz, acetone-d6
210 200 190 180 170 160 150 140 130 120 110 100 90 80 70 60 50 40 30 20 10
f1 (ppm)

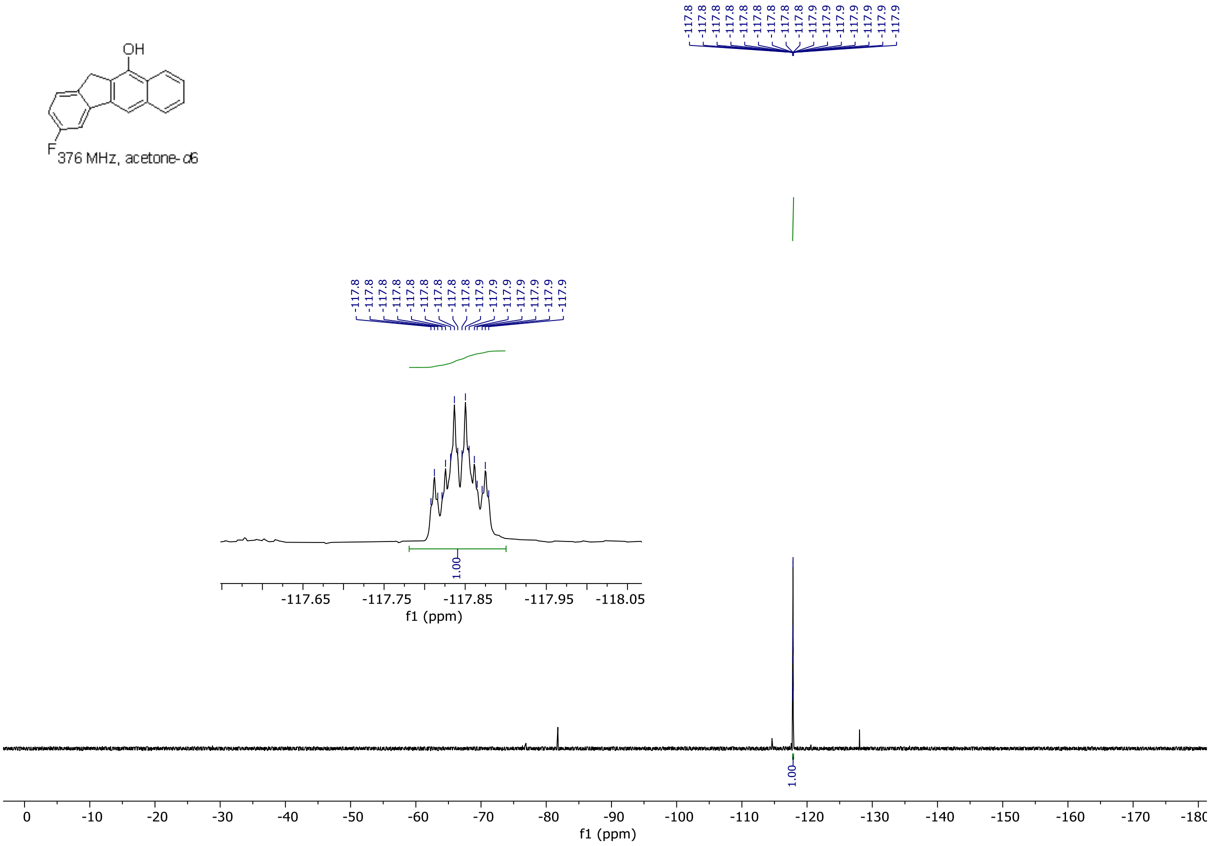
-117.8
-117.8
-117.8
-117.8
-117.8
-117.8
-117.8
-117.8
-117.9
-117.9
-117.9
-117.9
-117.9
-117.9
-117.9
OH
F
376 MHz, acetone-d6
1.00
-117.65 -117.75 -117.85 -117.95 -118.05
f1 (ppm)
1.00
0 -10 -20 -30 -40 -50 -60 -70 -80 -90 -100 -110 -120 -130 -140 -150 -160 -170 -180
f1 (ppm)

5-Bromo-11*H*-benzo[b]fluoren-10-ol (6a)

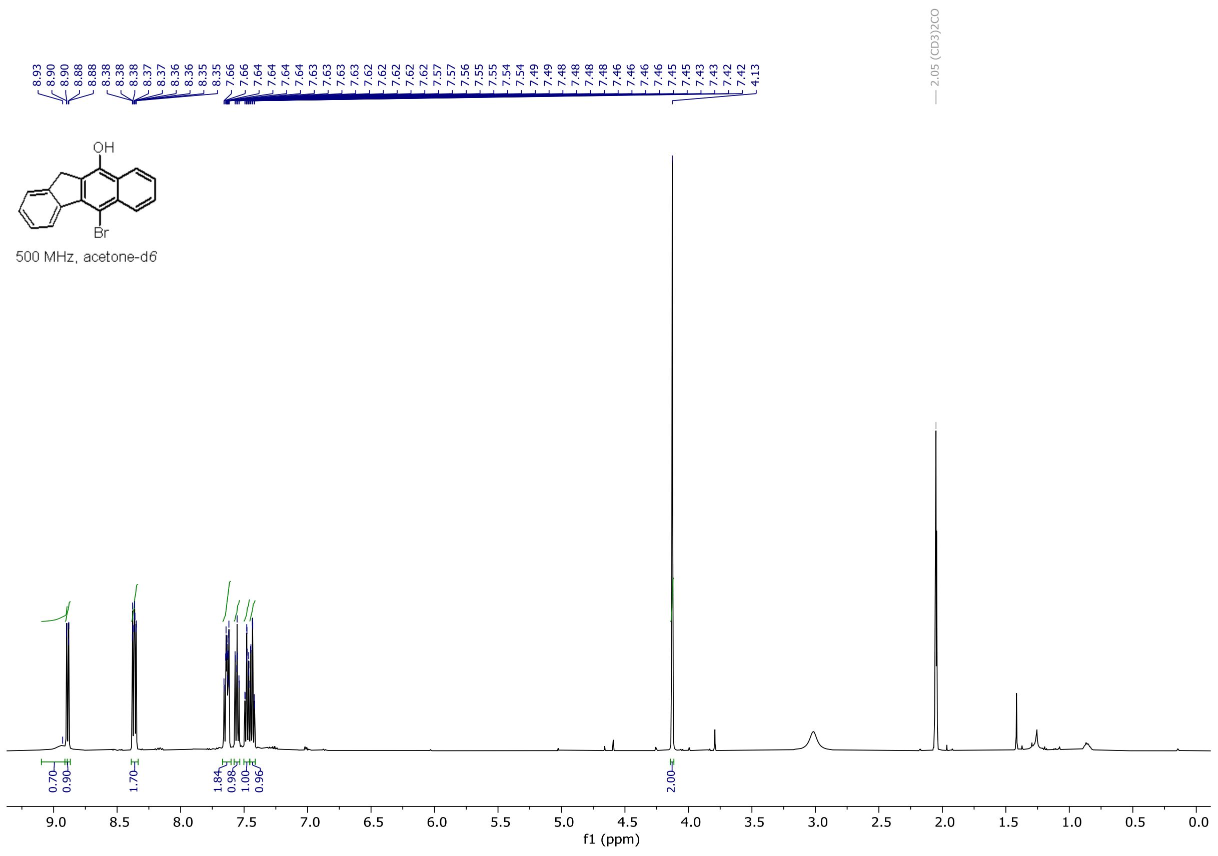

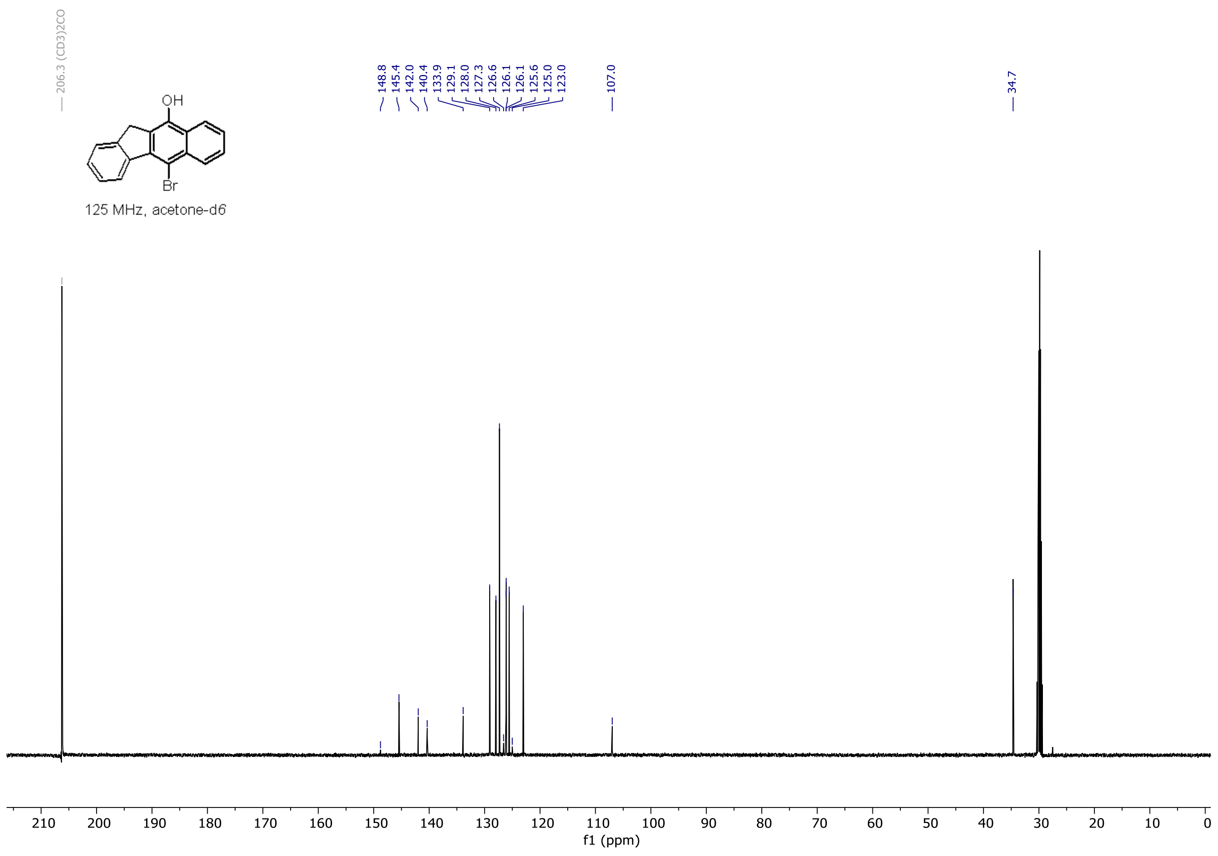

10-Methoxy-11*H*-benzo[*b*]fluorene (6b)

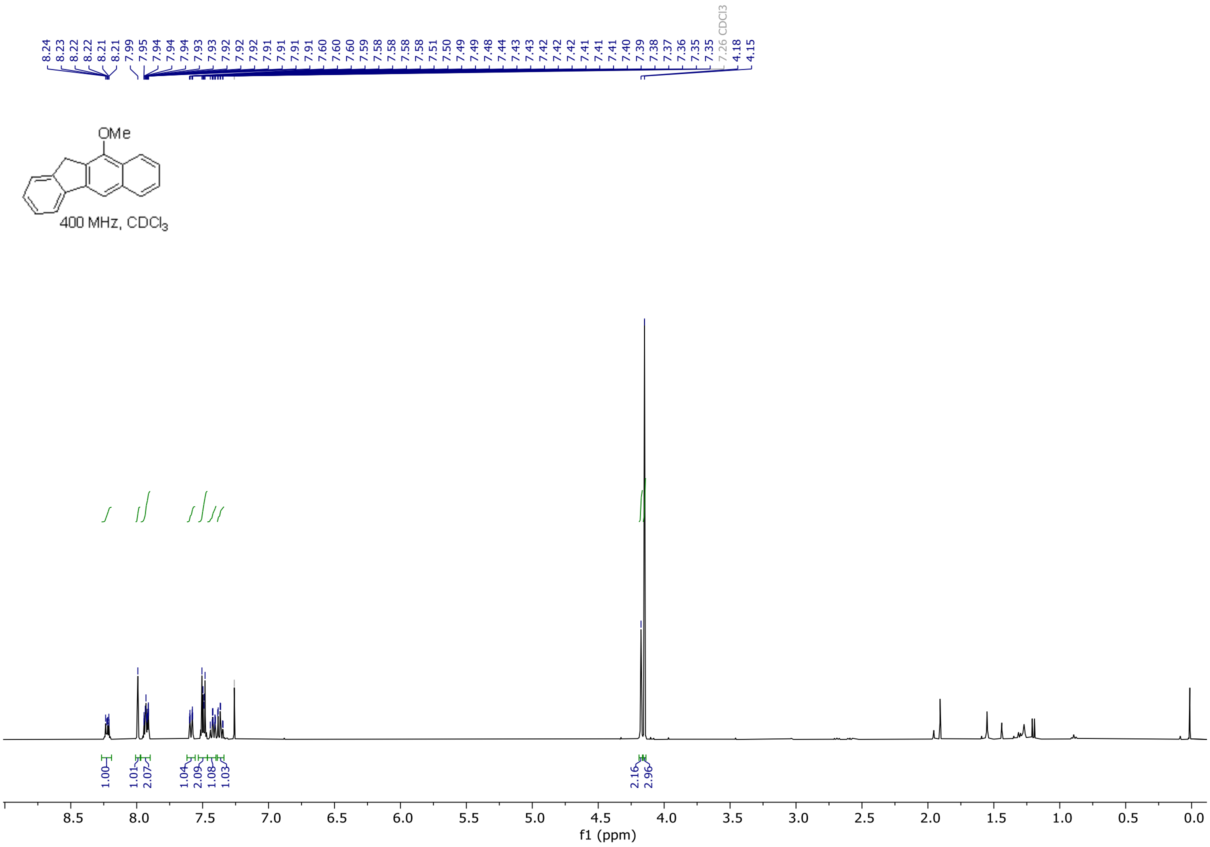

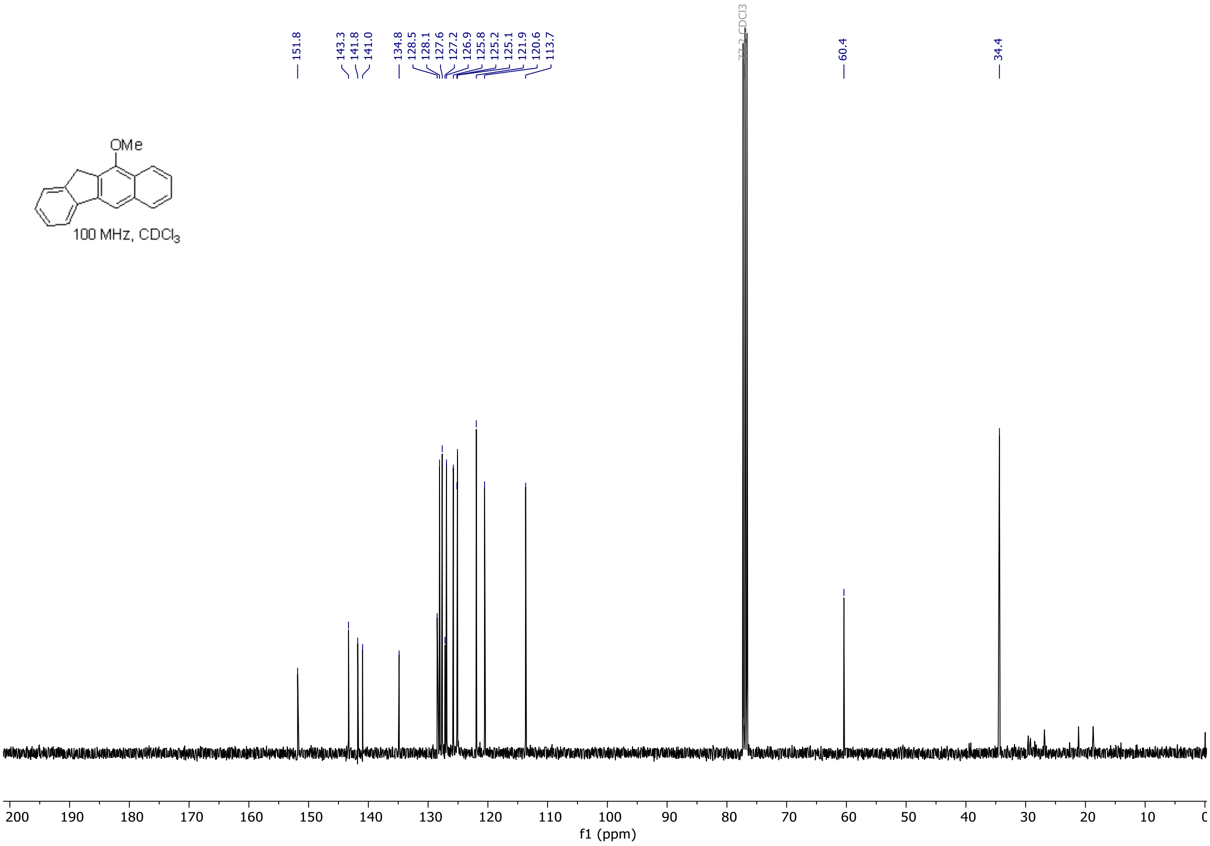

10-Methoxy-11*H*-benzo[b]fluoren-11-one (6c)

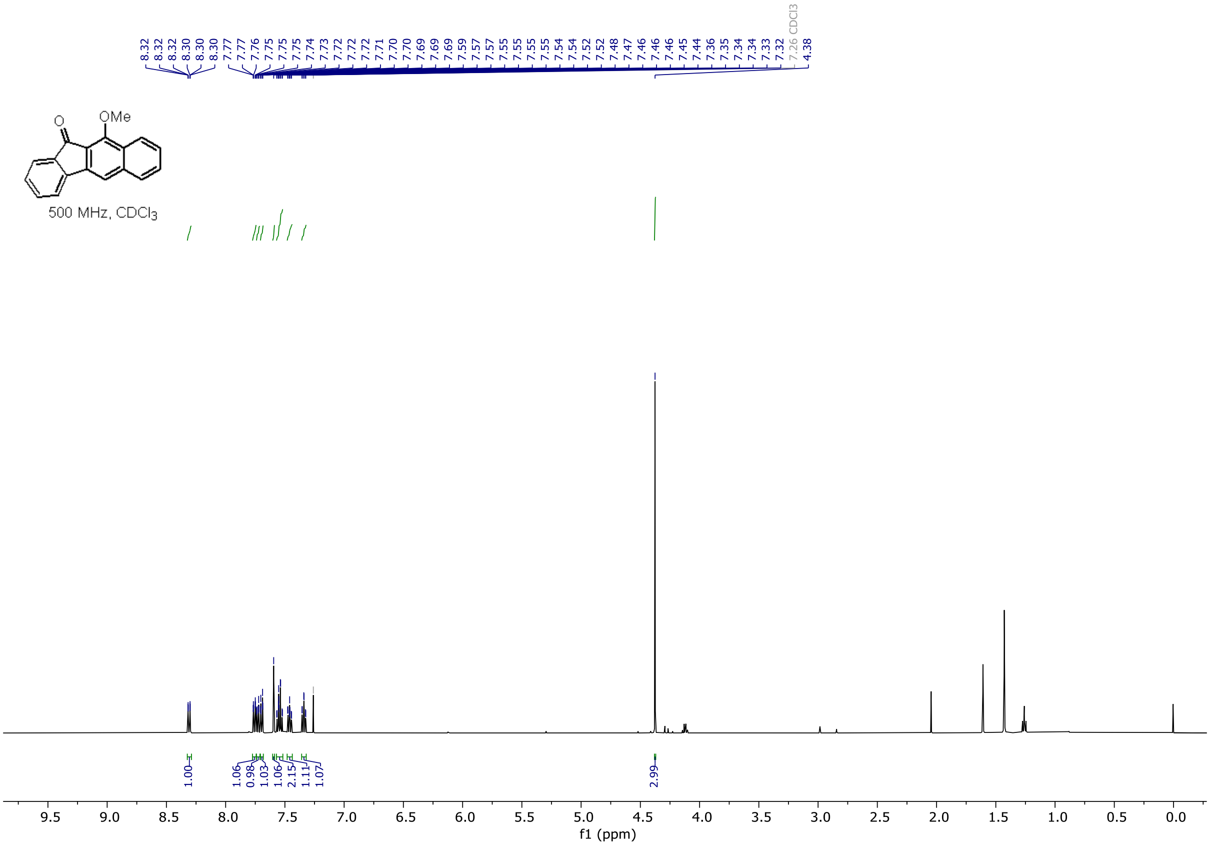

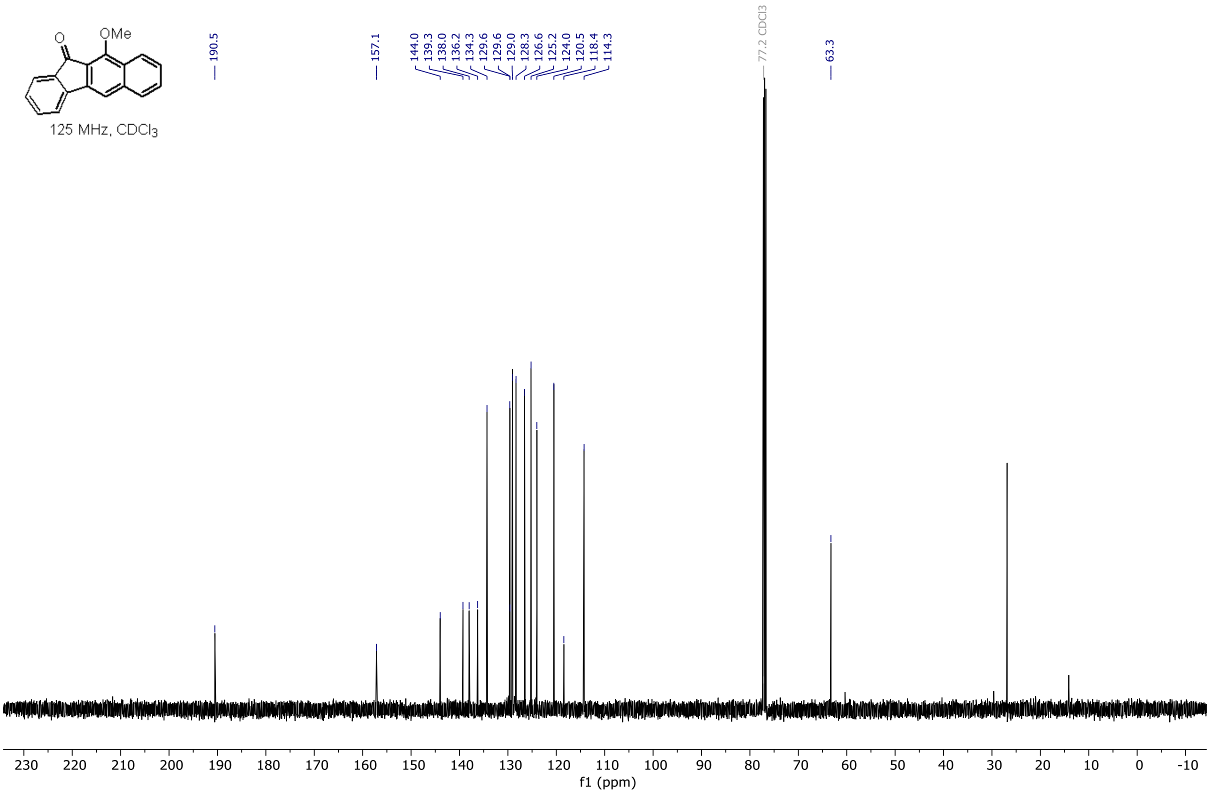

5-Bromo-10-methoxy-11*H*-benzo[*b*]fluorene (6d)

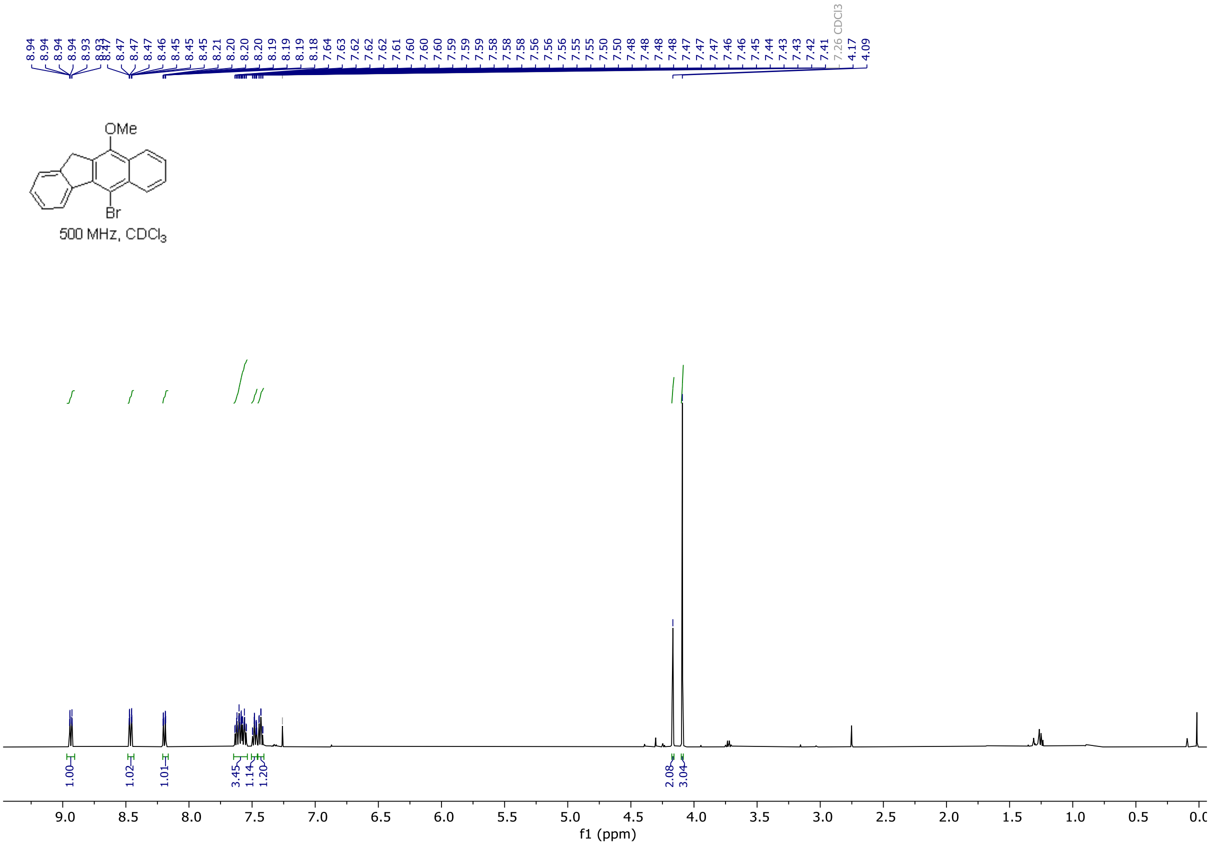

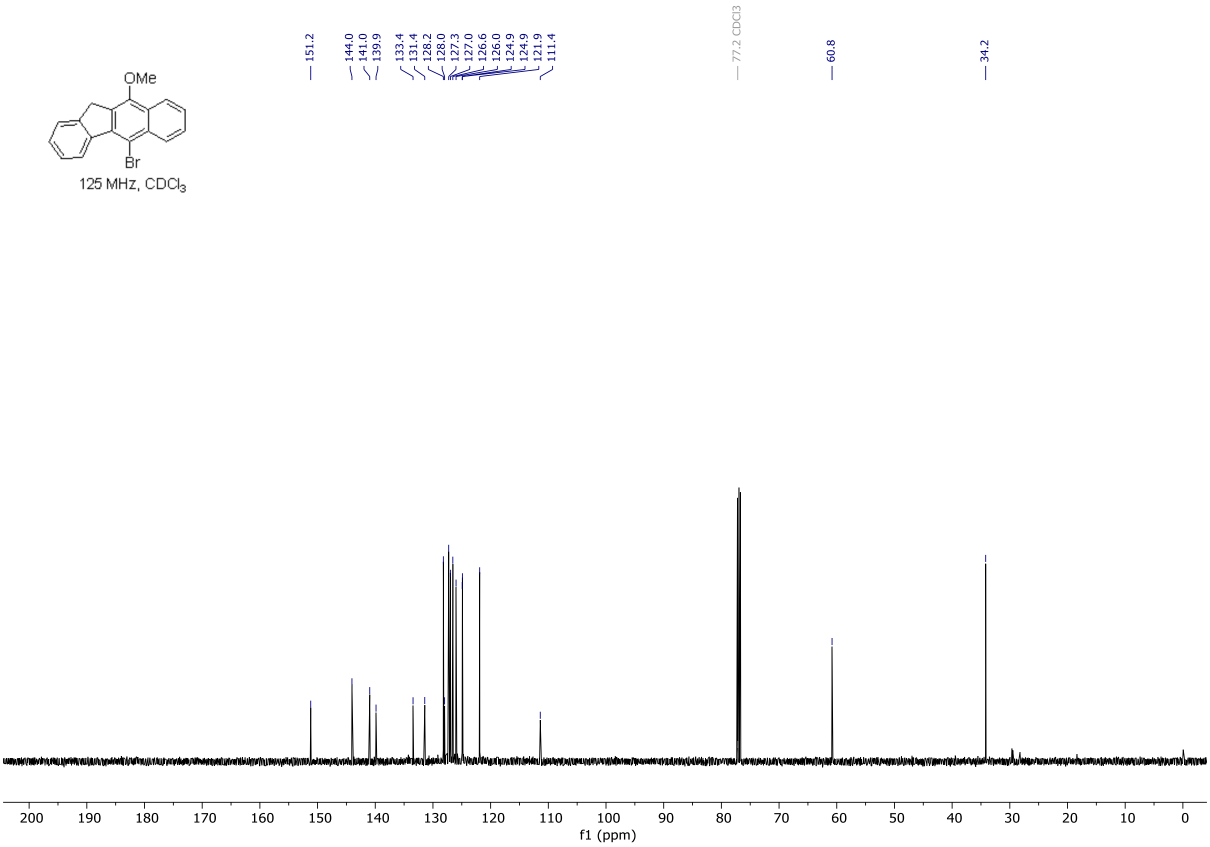

5-Bromo-10-methoxy-11*H*-benzo[*b*]fluoren-11-one (6e)

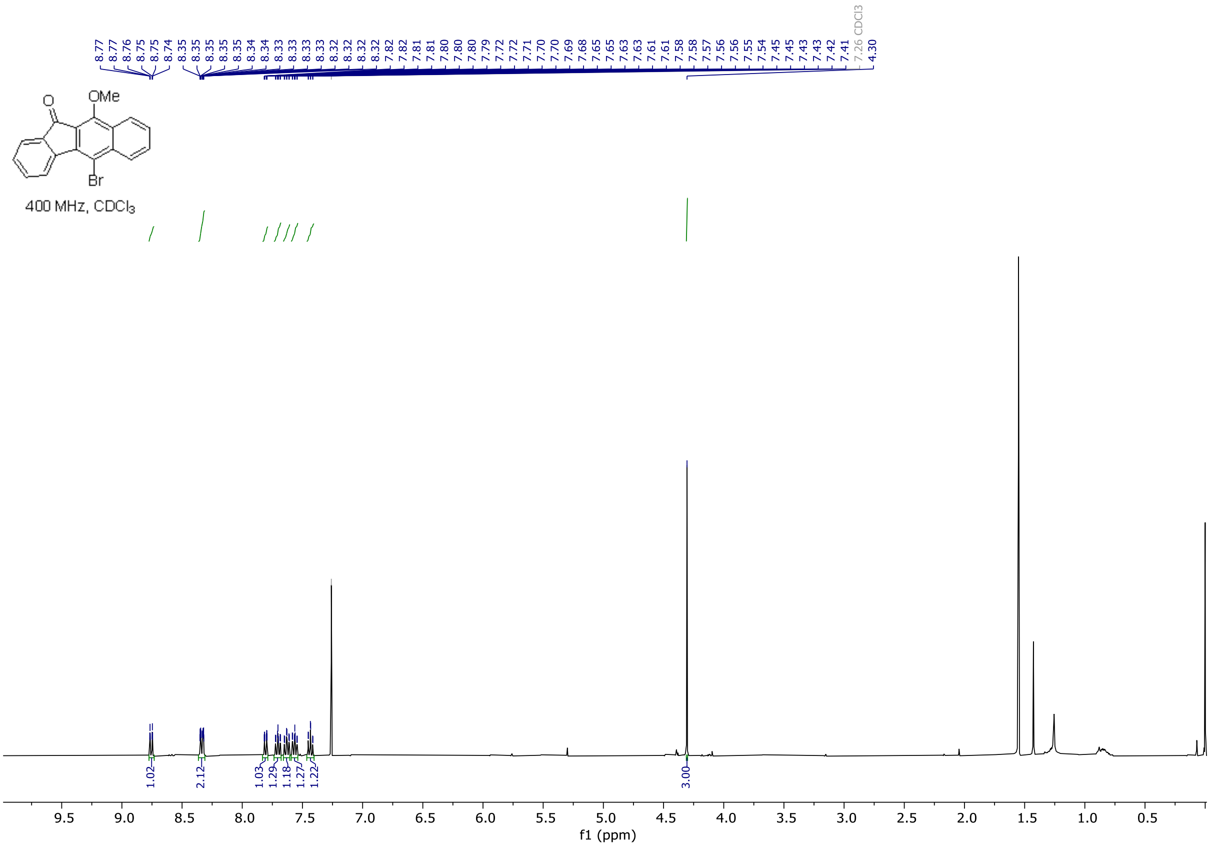

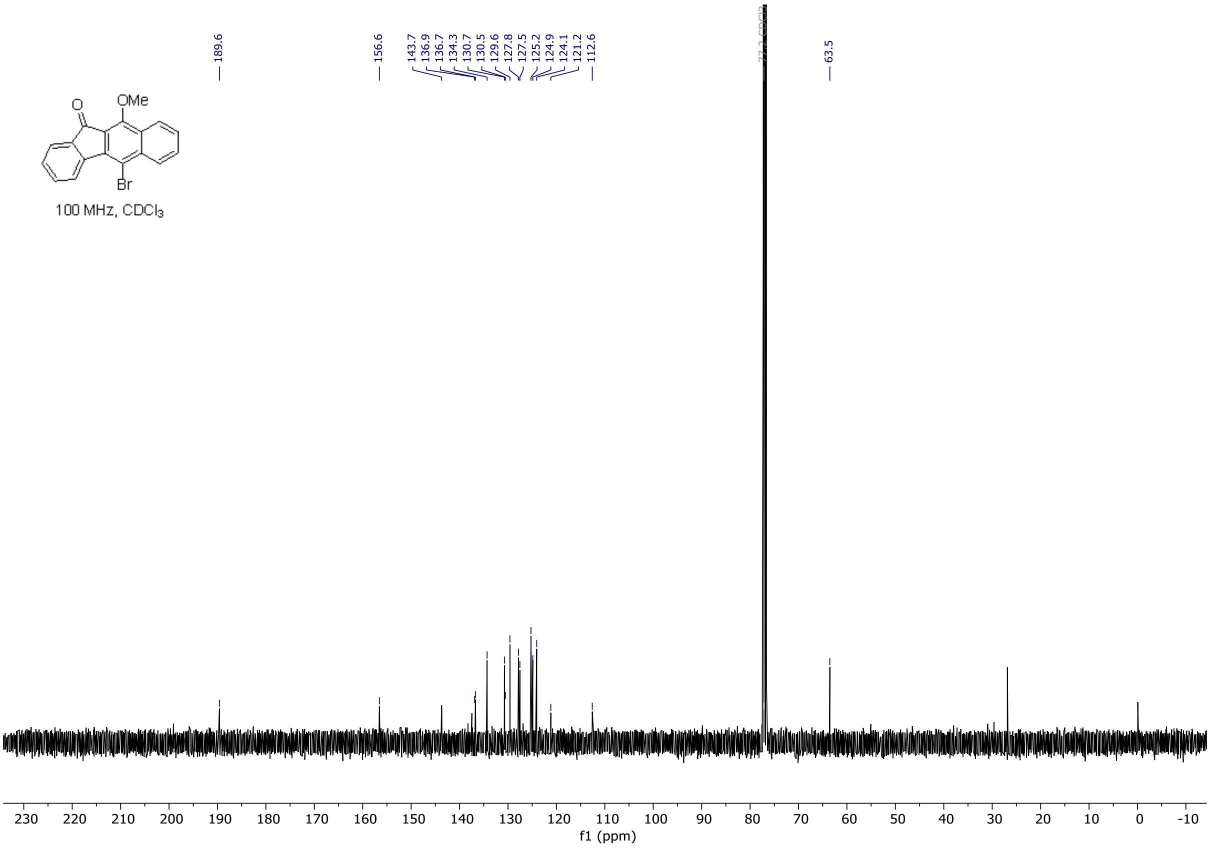

10-Methoxy-5-nitro-11H-benzo[*b*]fluoren-11-one (6f)

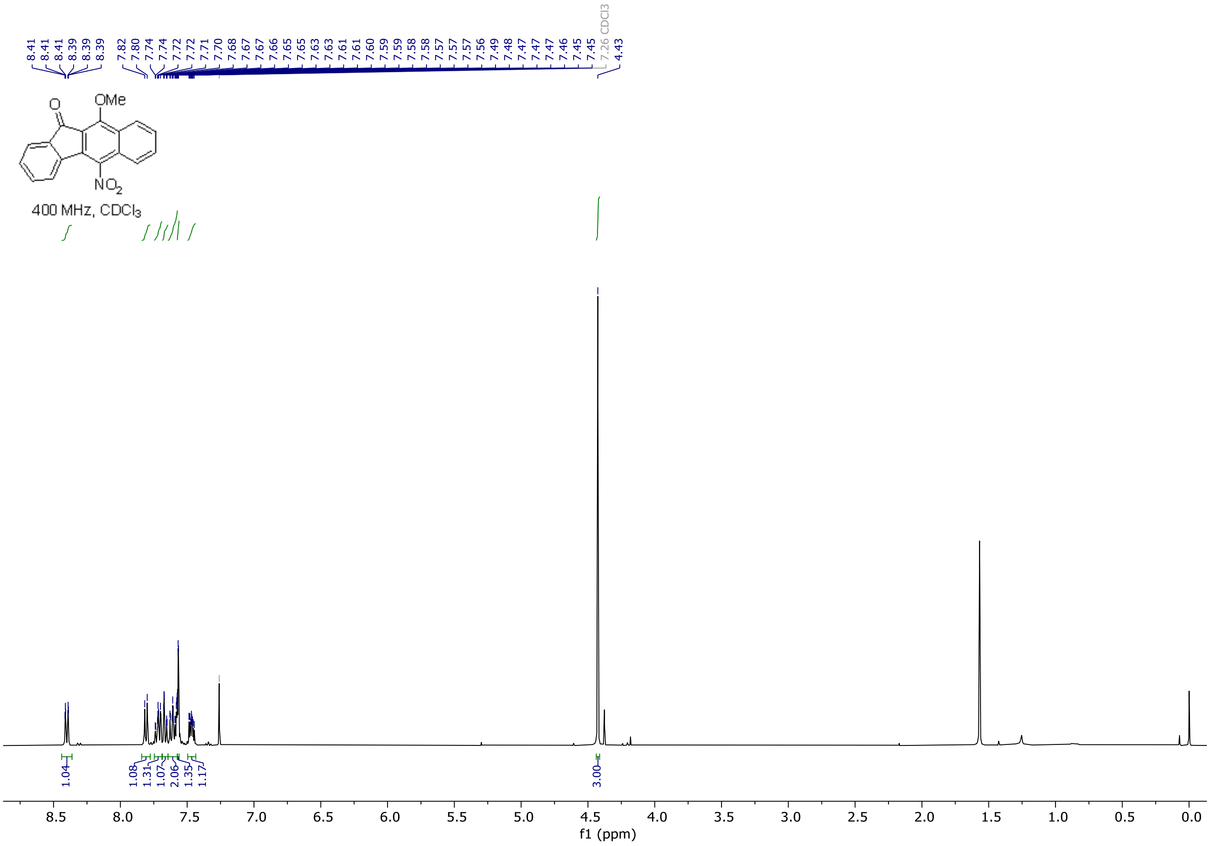

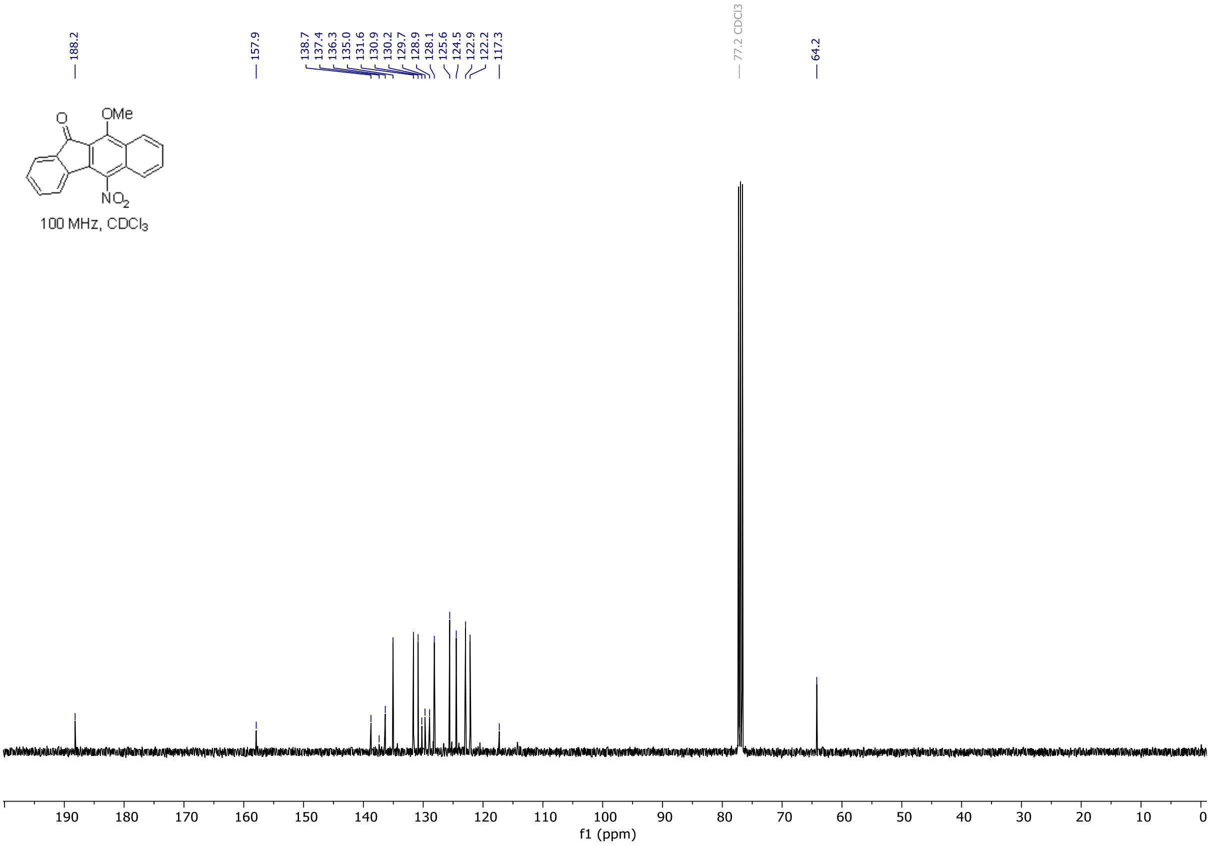

Copy of TEMPO Control HRMS Data

426.1863	426.1830	7.84	51	100.00	100.00	(M+K)+	1

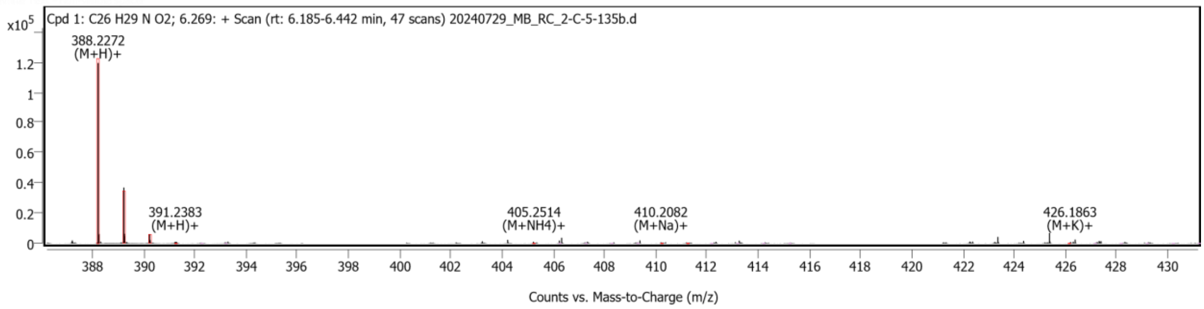

Copy of Deuterated Products HRMS Data

4b-D$_1$

275.0588	275.0604	-5.77	86	1.38	1.47	(M+K)+	1

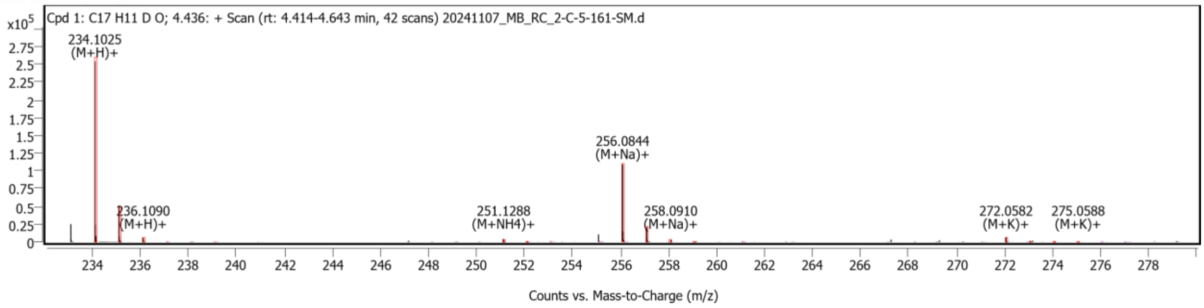

4b-D$_5$

278.0813	278.0832	-6.70	582	12.39	9.04	(M+K)+	1

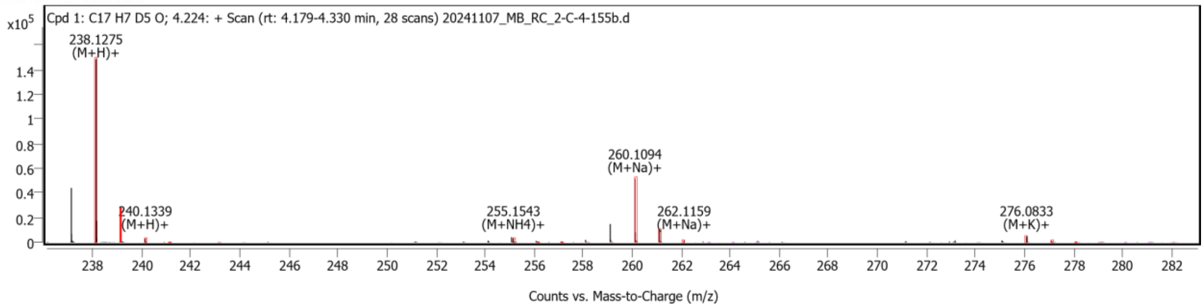

5b-D$_1$

258.0922	258.0908	5.51	69	1.48	1.83	(M+Na)+	1

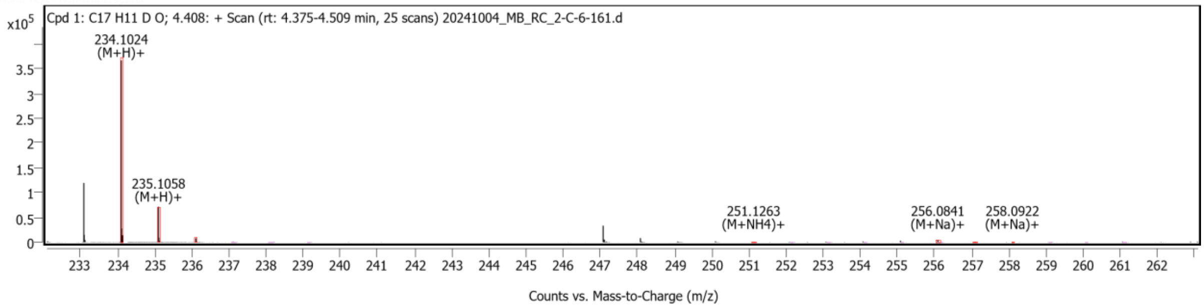

5b-D_5

261.1124	261.1128	-1.57	155	32.42	18.51	(M+Na)+	1

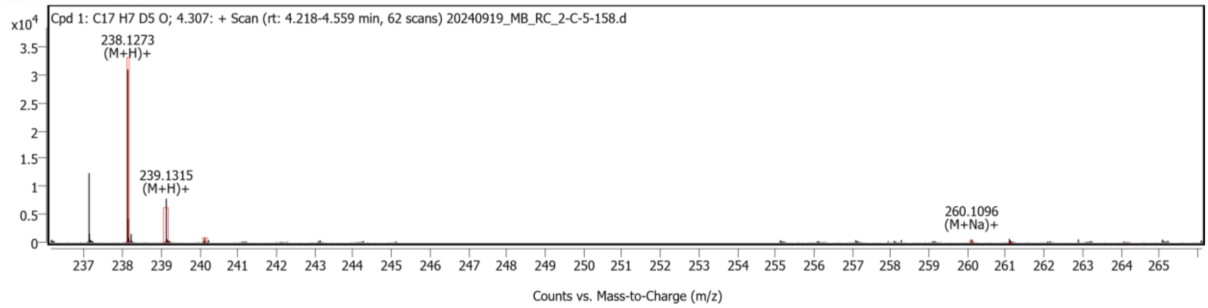

Supplement: Supplementary file 1 — ol4c03978_si_001.pdf [file ol4c03978_si_001.pdf]
