# Supplementary material for: Direct Photochemical Synthesis of Substituted Benzo[b]fluorenes
Source: Org Lett. 2024 Nov 25;26(48):10364–8. doi: 10.1021/acs.orglett.4c03978 (PMC11629382; doi:10.1021/acs.orglett.4c03978)
Supplement: Supplementary file 2 — ol4c03978_si_002.zip [file ol4c03978_si_002.zip › FID for Publication/20240601_RC-2-C-4-88A_01/plots/RC-2-C-4-88A_CARBON_20240601_01_plot01.pdf]

Sample Name:
RC-2-C-4-88A
Data Collected on:
newucd400-vnmrs400
Archive directory:
/home/data/MBaumann/ucd400/RuairiCrawford/2024
Sample directory:
20240601_RC-2-C-4-88A_01
FidFile: RC-2-C-4-88A_CARBON_20240601_01

Pulse Sequence: CARBON (s2pul)
Solvent: cdcl3
Data collected on: Jun 1 2024

Temp. 25.0 C / 298.1 K
Sample #57, Operator: MBaumann

Relax. delay 1.000 sec
Pulse 45.0 degrees
Acq. time 1.000 sec
Width 25000.0 Hz
2000 repetitions
OBSERVE C13, 100.5494845 MHz
DECOUPLE H1, 399.8803230 MHz
Power 41 dB
continuously on
WALTZ-16 modulated
DATA PROCESSING
Line broadening 0.5 Hz
FT size 65536
Total time 1 hr, 10 min

OpenVnmrJ

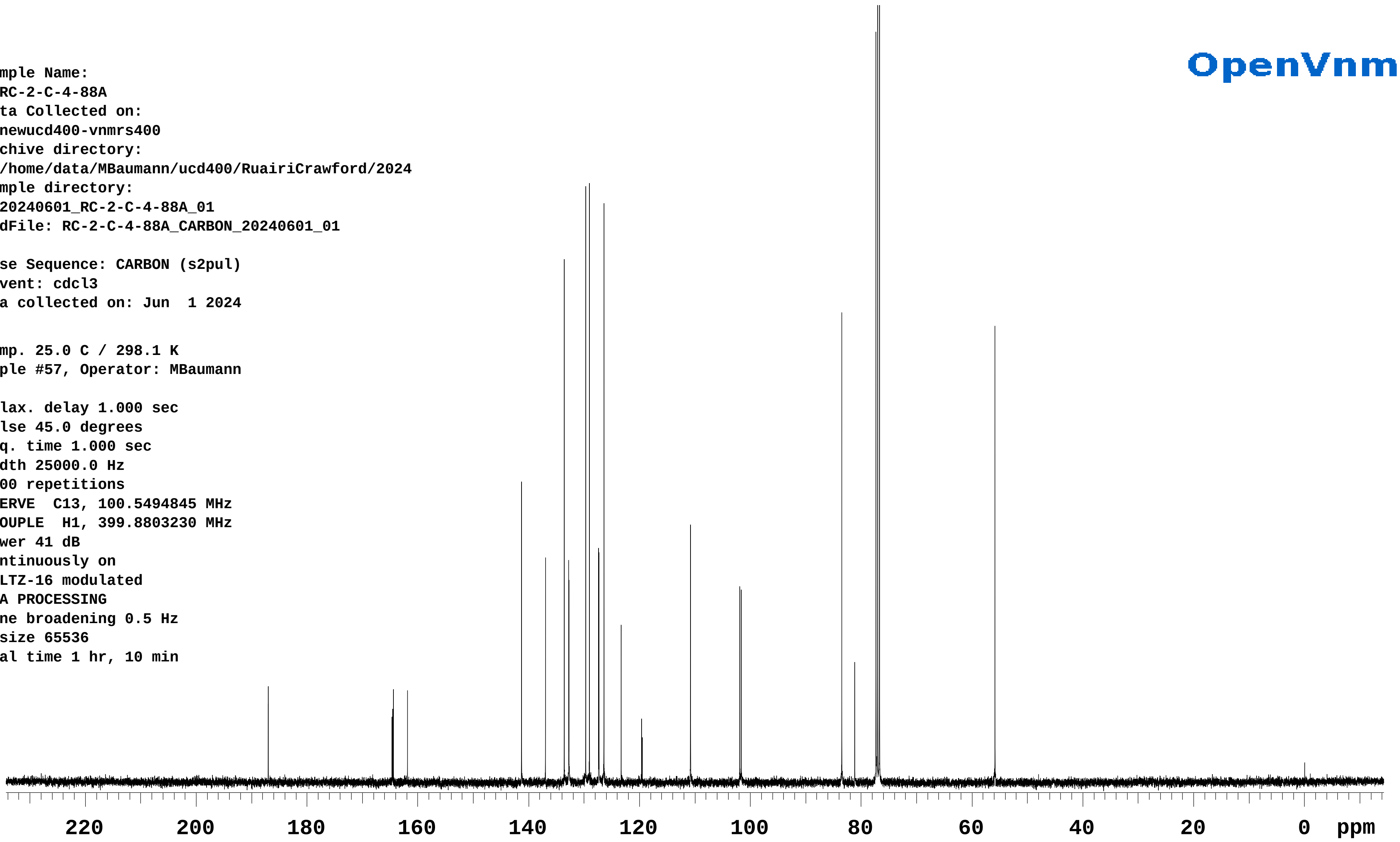

Plotname: RC-2-C-4-88A_CARBON_20240601_01_plot01
